# Barbaloin Alleviates Lung Ischemia-Reperfusion Injury by Dual-Targeting IL-6 and PNP

**DOI:** 10.3390/ijms27125276

**Published:** 2026-06-10

**Authors:** Huanhuan Dong, Niuniu Dong, Jinteng Feng, Yixing Li, Wenyu Peng, Zhiying Wang, Yihan Lin, Bin He, Zixuan Zhao, Xiaopeng Ma, Rongxuan Jiang, Yanpeng Zhang, Mao Sun, Guangjian Zhang

**Affiliations:** 1 Department of Thoracic Surgery, The First Affiliated Hospital, Xi’an Jiaotong University, Xi’an 710032, China; 2 Center of Precision Medicine Research, The First Affiliated Hospital, Xi’an Jiaotong University, Xi’an 710032, China; 3 Department of Biochemistry and Molecular Biology, School of Basic Medicine, Fourth Military Medical University, Xi’an 710032, China; 4 Department of Biomedical Engineering, The University of Melbourne, Melbourne, VIC 3010, Australia

**Keywords:** lung transplantation, ischemia-reperfusion injury, barbaloin, IL-6, PNP, oxidative stress, NF-κB/NLRP3 inflammasome

## Abstract

Ischemia-reperfusion injury (IRI) remains a primary driver of primary graft dysfunction (PGD) following lung transplantation, yet effective therapeutic strategies are currently limited. Early IRI is driven by coordinated oxidative and inflammatory responses, highlighting the need for therapeutic strategies capable of targeting both processes simultaneously. Using integrated human multi-omics analysis, in silico target prediction, and experimental validation, we identified barbaloin as a dual-target lead compound acting on interleukin-6 (IL-6) and purine nucleoside phosphorylase (PNP). In vitro, barbaloin suppressed IL-6 and PNP expression, inhibited PNP activity, reduced reactive oxygen species (ROS) accumulation, and attenuated NF-κB/NLRP3 inflammatory signaling. Crucially, in vivo validation in a C57BL/6J mouse model demonstrated that barbaloin (15 mg/kg) attenuated pulmonary edema and histological injury, partially restored respiratory mechanics, and reduced IL-6 and PNP expression. Collectively, these findings support the IL-6/PNP axis as a critical mediator of early lung IRI and identify barbaloin as a promising dual-target therapeutic candidate for mitigating oxidative and inflammatory injury during lung transplantation.

## 1. Introduction

Ischemia-reperfusion injury (IRI) is characterized by a complex multisystem response to a prolonged interruption of organ perfusion followed by its restoration. Although IRI occurs universally across solid organ transplantation, it is particularly relevant in lung transplantation, as transplanted lungs are especially vulnerable to reperfusion-associated injury [1]. In the transplanted lung, IRI manifests as sterile inflammation, increased microvascular permeability, endothelial dysfunction, and pulmonary edema, ultimately resulting in elevated pulmonary vascular resistance and impaired gas exchange. Clinically, this condition is recognized as primary graft dysfunction (PGD) [2]. Lung IRI (LIRI) and the resulting PGD lead to severe clinical complications, significantly increasing the healthcare burden of lung transplantation. Therefore, elucidating the molecular mechanisms underlying LIRI is critical for improving post-transplant outcomes [3]. Previous studies have demonstrated that ischemia triggers the release of damage-associated molecular patterns (DAMPs), which activate pattern-recognition receptors and promote cytokine upregulation, thereby initiating robust inflammatory responses during reperfusion [4]. Despite extensive research efforts, effective preventive or therapeutic interventions for clinical application remain limited, highlighting the urgent need for novel treatment strategies.

Barbaloin, the major anthraquinone glycoside isolated from *Aloe vera*, is traditionally recognized as a bittering agent [5]. Beyond its traditional use, barbaloin exhibits a broad range of pharmacological activities, including anti-inflammatory, antioxidant, anti-apoptotic, and anti-tumor effects [6,7,8]. Importantly, accumulating evidence suggests that barbaloin exerts protective effects in disease models sharing key pathological features with LIRI. For example, barbaloin attenuates lipopolysaccharide-induced acute lung injury by inhibiting ROS-mediated PI3K/AKT/NF-κB signaling [9]. Additionally, in myocardial ischemia-reperfusion models, barbaloin pre-treatment reduces tissue injury by activating AMPK, improving hemodynamics, and limiting infarct size [10].

Given this promising pharmacological profile and its reported efficacy in inflammatory and ischemic diseases, we hypothesized that barbaloin may exert protective effects against LIRI. To test this hypothesis, we employed an integrated strategy combining human multi-omics analysis, in silico target prediction, and experimental validation. Our analyses identified IL-6 and PNP as central mediators of early post-transplant injury and potential dual targets of barbaloin. Mechanistic studies further demonstrated that barbaloin alleviates oxygen-glucose deprivation/reoxygenation (OGD/R)-induced epithelial injury by suppressing IL-6/PNP-associated oxidative stress and inhibiting the downstream NF-κB/NLRP3 inflammasome pathway. Furthermore, in a C57BL/6J mouse lung ischemia-reperfusion model, barbaloin treatment attenuated reperfusion-induced upregulation of IL-6, PNP, and the downstream inflammatory cytokine IL-1β, reduced pulmonary edema, improved respiratory mechanics, and alleviated histopathological lung injury. Collectively, these findings suggest that the IL-6/PNP axis may play a central role in early LIRI and highlight barbaloin as a promising dual-target therapeutic candidate for mitigating oxidative and inflammatory injury during lung transplantation.

## 2. Results

### 2.1. Integrated Transcriptomic Analysis of Human Lung Transplant Biopsies

To systematically explore the molecular changes associated with early reperfusion in human lung transplantation, we performed integrated transcriptomic analyses using two independent cohorts (GSE127003, 46 paired samples; GSE145989, 67 paired samples). Peripheral lung biopsies were collected at the end of cold ischemia (cit) and 1–2 h post-reperfusion (1–2 h). After batch effect correction and data normalization, 113 matched pairs were retained for analysis (Supplementary Figure S1).

**Figure 1:**
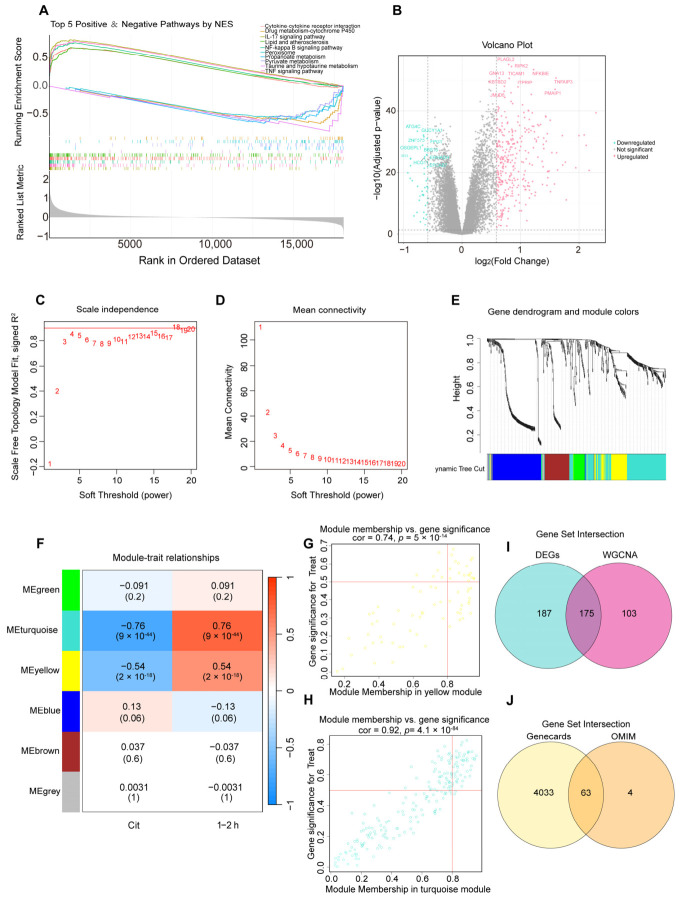
Identification of lung transplantation-related candidate genes. (**A**) Gene set enrichment analysis (GSEA) of the merged datasets GSE127003 and GSE145989. (**B**) Volcano plot of differential expression genes. (**C**) Scale-free fit analysis for selecting the soft-thresholding power (β) in weighted gene co-expression network analysis (WGCNA). Soft-thresholding powers 1–20 (indicated in red) were tested against the scale-free topology model fit (signed R^2^). A power of 4 (β = 4) was selected to meet the criterion of signed R^2^ ≥ 0.85, ensuring a scale-free network topology. (**D**) Mean connectivity analysis for soft threshold power selection in WGCNA. (**E**) Clustering dendrogram of genes and module identification by WGCNA. (**F**) Module-trait relationship heatmap; two lung transplantation associated modules were identified (Yellow: 74 genes; Turquoise: 204 genes), whose union yielded 278 genes. (**G**,**H**) Scatter plots of gene significance (GS) versus module membership (MM) for the yellow (**G**) and turquoise (**H**) modules. (**I**) Venn diagram showing the overlap between the 362 DEGs and the 278 WGCNA derived module genes. (**J**) Venn diagram showing the union of lung transplantation related genes retrieved from the GeneCards database (4096 genes, Gifts ≥ 5) and the OMIM database (67 genes), resulting in 4100 candidate genes.

Gene Set Enrichment Analysis (GSEA) of the combined dataset identified the top 10 differentially regulated pathways between cit and 1–2 h groups (Figure 1A), which mainly segregated into two major functional phenotype, the upregulated pathways of immune-inflammatory activation (including IL-17/TNF/NF-κB signaling, cytokine-cytokine receptor interaction, and lipid and atherosclerosis), and downregulated pathways of metabolic suppression (involving taurine/propanoate/pyruvate metabolism, peroxisome-mediated fatty acid oxidation, and cytochrome P450 metabolism). These results suggest that early reperfusion triggers a pronounced inflammatory response while suppressing metabolic functions in the transplanted lung.

To identify key driver genes, we performed differential expression analysis, yielding 362 significantly altered genes (DEGs; *q* < 0.05, |log_2_FC| ≥ 0.585), including 319 upregulated and 43 downregulated genes (Figure 1B).

We then applied weighted gene co-expression network analysis (WGCNA) to identify modules of functionally coordinated genes, as this method allows detection of groups of genes that act together rather than individually [11]. Using a soft-thresholding power of 4 (β = 4; scale-free topology fit with signed R^2^ ≥ 0.85; Figure 1C,D), six distinct co-expression modules were identified (Figure 1E). The turquoise (204 genes) and yellow (74 genes) modules demonstrated the strongest positive correlation with the transplantation phenotype (Figure 1F). Functional annotation revealed that the yellow module was enriched for acute innate immune effector functions (e.g., neutrophil activation), while the turquoise module was enriched for broad inflammatory pathways (IL-17, TNF, NF-κB), indicating sustained inflammatory responses (Supplementary Figure S2). High correlations between gene significance (GS) and module membership (MM) for both modules (Turquoise: *r* = 0.76, *p* = 9 × 10^−44^; Yellow: *r* = 0.54, *p* = 2 × 10^−18^; Figure 1G,H) confirmed their biological relevance, which establishing 278 high-confidence candidate genes. Together, these analyses indicate that specific co-expression modules underpin the early inflammatory response following reperfusion, providing a focused set of candidate genes for further study.

To further narrow down the screening range of the key driving gene, the overlap of 175 candidate genes between the 362 DEGs and the 278 WGCNA was identified (Figure 1I). Additionally, a reference set of 4100 genes associated with “Lung Transplantation” was compiled from the GeneCards and OMIM databases (Figure 1J). All gene lists are provided in the Supplementary Tables: DEGs and phenotype-linked WGCNA modules (Supplementary Table S1), and the reference gene set derived from GeneCards and OMIM (Supplementary Table S2).

### 2.2. Barbaloin Structure Acquisition and Target Gene Prediction

**Figure 2:**
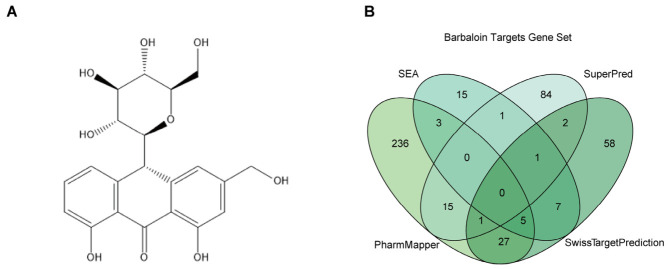
Chemical structure of barbaloin and integrated in silico target prediction. (**A**) Two-dimensional chemical structure of barbaloin. (**B**) Integrated target prediction workflow using PharmMapper, SEA, SuperPred, and SwissTargetPrediction. Predicted targets from individual platforms (290, 32, 104, and 102 targets, respectively) were standardized, merged, and deduplicated to generate 455 nonredundant candidate targets.

To investigate potential molecular targets of barbaloin, its two-dimensional (2D) structure was generated using ChemBio3D (version 14.0), and SMILES notation was obtained (Figure 2A). Target prediction was performed using four independent platforms: PharmMapper, the Similarity Ensemble Approach (SEA), SuperPred, and SwissTargetPrediction. These platforms integrate complementary ligand-based and pharmacophore-based prediction algorithms to comprehensively capture putative targets. Initial predictions yielded 290, 32, 104, and 102 targets, respectively. All predicted targets were standardized using UniProt identifiers, merged, and deduplicated, resulting in a total of 455 unique genes as the predicted target pool for barbaloin (Supplementary Table S3). These results provide a comprehensive set of candidate targets for subsequent integration with transcriptomic and disease-associated gene datasets.

### 2.3. Identification and Functional Characterization of Key Target Genes Linking Barbaloin to Lung Transplantation

**Figure 3:**
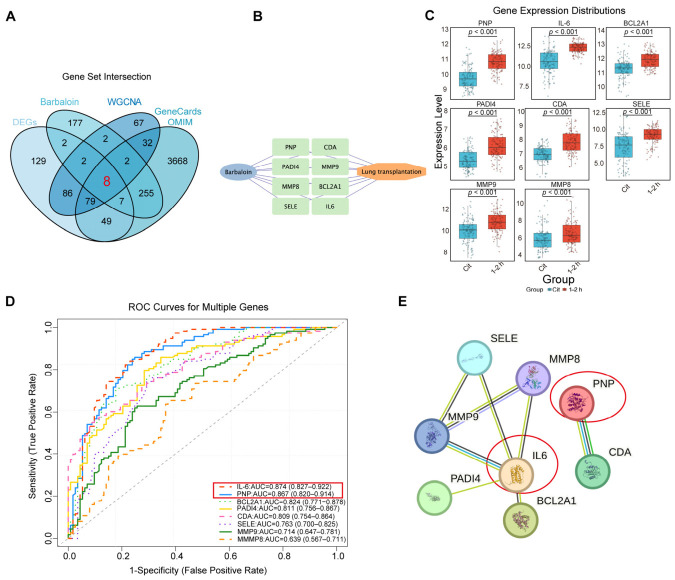
Identification of IL-6 and PNP as core barbaloin-associated targets in lung transplantation. (**A**) Venn diagram showing the intersection of four gene sets: differentially expressed genes (DEGs), WGCNA-derived module genes, lung transplantation-associated genes (GeneCards and OMIM), and predicted barbaloin targets, resulting in eight overlapping candidate genes. (**B**) Regulatory network of barbaloin-key target genes-lung transplantation. (**C**) Differential expression analysis of the eight candidate genes in the integrated transcriptomic dataset, showing significant upregulation after reperfusion compared with the cold ischemia (cit) group. (**D**) Receiver operating characteristic (ROC) curve analysis evaluating the diagnostic performance of candidate genes for reperfusion injury, highlighting IL-6 and PNP as top-performing targets. (**E**) Protein-protein interaction (PPI) network of candidate genes, identifying IL-6 and PNP as central hub nodes.

To identify barbaloin targets relevant to lung transplantation pathology, we intersected four gene sets: the 362 DEGs, the 278 WGCNA-derived genes, 4100 lung transplantation-associated genes, and the 455 predicted barbaloin targets. This integrative screening identified eight key genes: PNP, IL-6, BCL2A1, PADI4, CDA, SELE, MMP9, and MMP8 (Figure 3A). A regulatory network visualizing interactions between barbaloin and these genes was constructed using Cytoscape (version 3.9.0; Figure 3B). All eight genes were significantly upregulated at 1–2 h post-reperfusion compared to the cit group (*p* < 0.001, Figure 3C). Receiver operating characteristic (ROC) curve analysis indicated that IL-6 and PNP exhibited high diagnostic accuracy for reperfusion injury (IL-6: AUC = 0.874, 95% CI: 0.827–0.922; PNP: AUC = 0.867, 95% CI: 0.820–0.914; Figure 3D). Moreover, protein-protein interaction (PPI) analysis highlighted IL-6 and PNP as central nodes (score ≥ 0.4; Figure 3E). Together, these findings indicate that IL-6 and PNP are central mediators of early reperfusion injury and key candidate targets for barbaloin.

### 2.4. Immunological Characterization and Pathway Functional Annotation of Core Hub Genes

**Figure 4:**
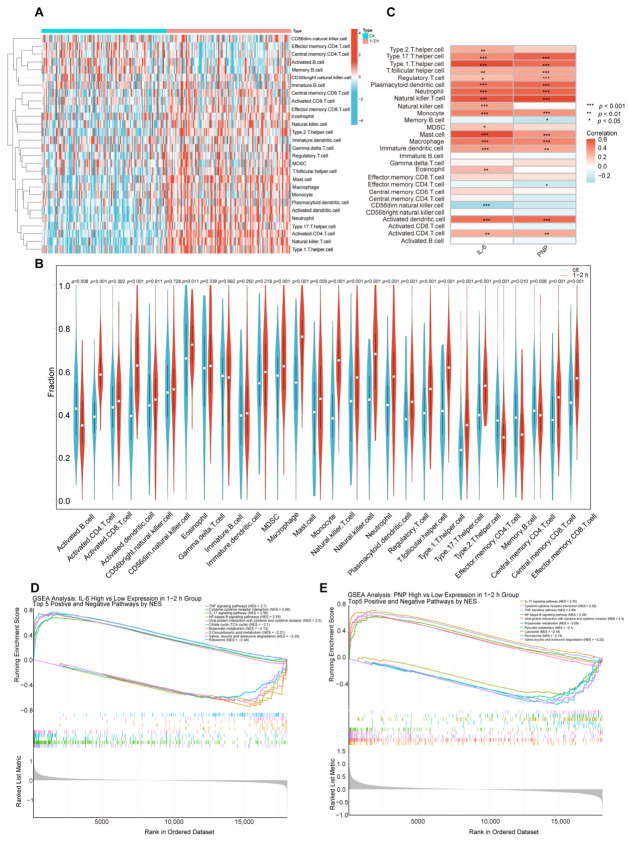
Immunological characterization and pathway annotation of the core hub genes IL-6 and PNP. (**A**) Heatmap showing single-sample gene set enrichment analysis (ssGSEA) scores of 28 immune cell populations in cold ischemia (cit) and 1–2 h reperfusion samples, illustrating immune microenvironment remodeling after reperfusion. (**B**) Differential immune infiltration analysis comparing immune cell enrichment scores between cit and reperfusion groups, highlighting significantly altered immune cell populations (Wilcoxon test). (**C**) Spearman correlation heatmap showing associations between IL-6/PNP expression and immune cell infiltration levels, demonstrating positive correlations with key innate and adaptive immune cell subsets. (**D**,**E**) Gene set enrichment analysis (GSEA) of IL-6 (**D**) and PNP (**E**), showing enrichment of immune- and inflammation-related pathways, including IL-17, TNF, and NF-κB signaling. Data are presented as enrichment scores or correlation coefficients as indicated. * *p* < 0.05, ** *p* < 0.01, *** *p* < 0.001.

To elucidate the immunological functions of the core hub genes IL-6 and PNP during early post-reperfusion injury, we performed single-sample gene set enrichment analysis (ssGSEA) followed by correlation analysis [12]. ssGSEA revealed profound remodeling of the immune microenvironment after reperfusion, with significant changes in the infiltration levels of 21 out of 28 immune cell subsets compared to the cit group (Figure 4A,B). Notably, the CD4^+^ T-cell compartment was broadly and strongly activated (*p* < 0.001), including both pro-inflammatory (Th1, Th17) and regulatory (Treg) subsets, reflecting disrupted immune homeostasis alongside active counter-regulation. Spearman correlation analysis revealed that both two core hub genes are positively correlated with the activation of key innate and adaptive immune cells, including neutrophils, Th1 cells, and dendritic cells (Figure 4C). Furthermore, IL-6 and PNP were significantly enriched in major pro-inflammatory cascades, such as IL-17, TNF and NF-κB signaling, suggesting them as important mediators of the dysregulated immune response and inflammatory amplification in early lung allograft injury (Figure 4D,E). These results suggest that IL-6 and PNP serve as central drivers of immune dysregulation and inflammatory amplification during early lung allograft injury.

### 2.5. Cellular Mapping of Core Hub Genes by Single-Cell Transcriptomics

**Figure 5:**
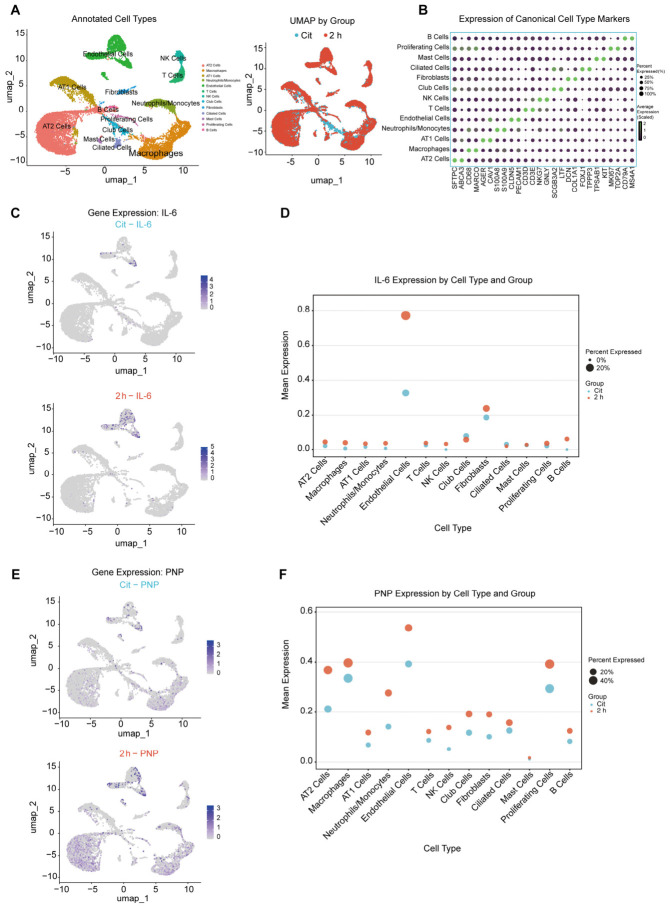
Single-cell transcriptomic characterization of IL-6 and PNP expression during early lung reperfusion injury. (**A**) Uniform manifold approximation and projection (UMAP) visualization of 104,127 cells from 12 lung transplant samples, colored by annotated cell type (left) and by experimental condition (right: cold ischemia [cit] and 2 h reperfusion). (**B**) Dot plot showing the expression of canonical lineage-specific markers used for cell type annotation across 14 identified cell populations. (**C**,**E**) UMAP feature plots illustrating the spatial distribution of IL-6 (**C**) and PNP (**E**) expression under cit (top) and reperfusion (bottom) conditions. (**D**,**F**) Dot plots showing cell type-specific expression patterns of IL-6 (**D**) and PNP (**F**) across experimental conditions. IL-6 exhibited inducible enrichment predominantly in endothelial and myeloid populations following reperfusion, whereas PNP displayed broader expression across both structural and immune cell compartments.

To precisely delineate the cellular sources and dynamics of IL-6 and PNP during early graft injury, we analyzed longitudinal single-cell RNA-seq data from six lung transplant recipients (GSE220797), encompassing matched samples from cold preservation (cit) and 2-hour (2 h) reperfusion time points (Supplementary Figure S3). Unsupervised clustering of 104,127 high-quality cells identified 14 distinct cell populations, which were annotated using canonical lineage-specific markers (Figure 5A,B). Analysis revealed distinct cellular expression patterns for IL-6 and PNP after reperfusion (Figure 5C–F). IL-6 was highly inducible and predominantly expressed in endothelial and myeloid cells post-reperfusion, whereas PNP showed broad constitutive expression across structural and immune cells, with moderate but widespread upregulation. These single-cell results indicate that IL-6 and PNP coordinately contribute to the early cellular response to reperfusion injury, with IL-6 acting in a cell type-specific manner and PNP providing a ubiquitous modulatory role.

### 2.6. Molecular Docking and Molecular Dynamics Simulations of Barbaloin with Core Hub Targets

**Figure 6:**
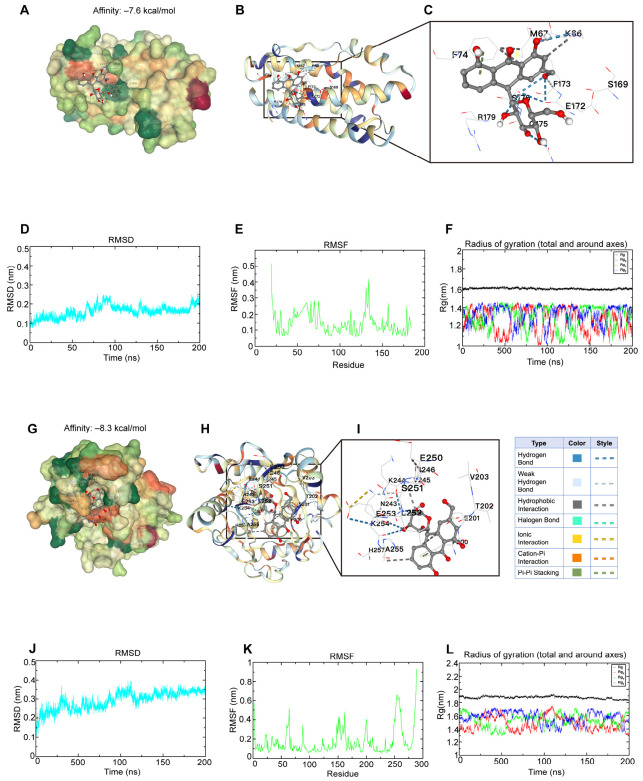
Molecular docking (MD) and molecular dynamics simulations (MDS) analyses of barbaloin with IL-6 and PNP. (**A**–**C**) MD analysis of the IL-6-barbaloin complex, showing the predicted binding cavity, docking pose, and key residue interactions. (**D**–**F**) MDS analysis of the IL-6-barbaloin complex, including root-mean-square-deviation (RMSD), root-mean-square fluctuation (RMSF), and radius of gyration (Rg), demonstrating structural stability during the 200 ns simulation. (**G**–**I**) MD analysis of the PNP-barbaloin complex, showing the catalytic binding pocket, docking pose, and key molecular interactions. (**J**–**L**) MDS analysis of the PNP-barbaloin complex, including RMSD, RMSF, and Rg, indicating stable binding and structural compactness throughout the simulation.

To explore potential direct interactions between barbaloin and the core hub genes, we performed molecular docking (MD) and molecular dynamics simulations (MDS) for IL-6 and PNP [13,14]. Computational simulations were chosen to provide structural and energetic insights into ligand-target interactions prior to experimental validation. Barbaloin formed a complex with IL-6 with a calculated binding affinity of −7.6 kcal/mol (Figure 6A,B). The ligand occupied a functional domain of IL-6, engaging key interfacial residues including LYS66, MET67, PHE74, SER169, GLU172, PHE173, GLN175, SER176, and ARG179 (Figure 6C). Notably, residues SER169, GLU172, PHE173, and GLN175 lie within a critical region (residues 128–175) essential for IL-6 biological activity, highlighting the potential functional relevance of this interaction [15]. For PNP, docking affinity was −8.3 kcal/mol (Figure 6G,H), with the ligand localizing to the active site and interacting with residues PHE200, GLU201, THR202, VAL203, ASN243, LYS244, VAL245, ILE246, GLU250, SER251, GLU253, LYS254, ALA255, ASN256, and HIS257 (Figure 6I). Key residues PHE200, GLU201, and ASN243 constitute the catalytic and substrate-binding pocket of PNP [16], suggesting that barbaloin may modulate enzymatic function via competitive or allosteric mechanisms. Subsequent molecular dynamics simulations further confirmed the structural stability of both complexes. The IL-6-barbaloin complex reached equilibrium rapidly, with backbone root-mean-square-deviation (RMSD) stabilizing at 0.1–0.25 nm within 50 ns (Figure 6D). Root-mean-square fluctuation (RMSF) analysis showed minimal fluctuations (<0.2 nm) in active site residues (Figure 6E), and the radius of gyration (Rg) remained constant at ~1.6 nm (Figure 6F), indicating a stable, well-folded structure. For the PNP-barbaloin complex, backbone RMSD stabilized at 0.2–0.4 nm after 100 ns (Figure 6J). Terminal regions showed higher flexibility, while binding interface residues exhibited reduced fluctuations (Figure 6K). Rg remained stable at ~1.9 nm (Figure 6L), confirming structural compactness. Together, these computational results demonstrate that barbaloin can stably and energetically favorably interact with IL-6 and PNP, providing a structural rationale for its potential modulation of these core hub targets in LIRI.

### 2.7. The Protective Mechanism of Barbaloin in the OGD/R Cell Model

To investigate whether barbaloin directly protects lung epithelial cells from ischemia-reperfusion-like stress, we established an in vitro OGD/R model in BEAS-2B bronchial epithelial cells, which recapitulates key epithelial features of early LIRI, including oxidative stress and inflammatory activation [17]. We first performed CCK-8 assays to evaluate the cytotoxicity and protective concentration range of barbaloin under OGD/R conditions.

**Figure 7:**
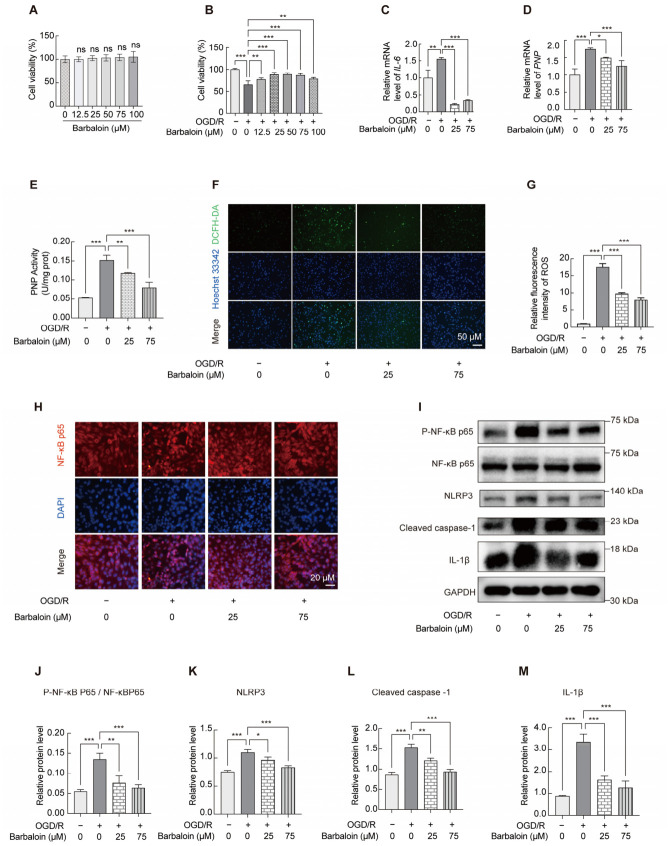
Barbaloin protects BEAS-2B cells against oxygen-glucose deprivation/reoxygenation (OGD/R) injury by mitigating oxidative stress and inhibiting the NF-κB/NLRP3 pathway. (**A**) CCK-8 assay showing cell viability after 24 h barbaloin treatment (12.5–100 µM) under normoxic conditions, confirming lack of cytotoxicity. (**B**) CCK-8 assay demonstrating recovery of BEAS-2B cell viability in OGD/R model after barbaloin pretreatment at 12.5–100 µM. (**C**,**D**) QRT-PCR analysis of IL-6 (**C**) and PNP (**D**) mRNA in OGD/R cells, showing barbaloin effectively reverses OGD/R-induced upregulation. (**E**) PNP enzyme activity assay, showing barbaloin inhibits OGD/R-induced enzymatic activation. (**F**,**G**) Intracellular reactive oxygen species (ROS) levels assessed by DCFH-DA staining (**F**) and quantitative analysis (**G**), indicating dose-dependent ROS suppression by barbaloin. (**H**) Immunofluorescence staining of NF-κB p65 (red) and DAPI (blue), showing barbaloin prevents nuclear translocation induced by OGD/R. (**I**–**M**) Western blot analysis of P-NF-κB p65, NF-κB p65, NLRP3, Cleaved caspase-1, and IL-1β protein expression. Data are presented as mean ± SD. (**B**) unpaired two-tailed Student’s *t*-test; all other panels: one-way ANOVA with Tukey’s test. * *p* < 0.05, ** *p* < 0.01, *** *p* < 0.001; ns = not significant.

CCK-8 assays demonstrated that barbaloin exhibited no significant cytotoxicity after 24 h of treatment at concentrations ranging from 12.5 to 100 μM. (Figure 7A). OGD/R markedly reduced cell viability, whereas barbaloin pretreatment dose-dependently improved cell survival at concentrations of 25–75 μM (*p* < 0.001; Figure 7B). To determine whether these protective effects were associated with modulation of the predicted targets, IL-6 and PNP expression levels were subsequently assessed. Barbaloin significantly attenuated the OGD/R-induced upregulation of IL-6 and PNP mRNA (Figure 7C,D) and consistently inhibited OGD/R-triggered PNP enzymatic activity (Figure 7E). These findings suggest that barbaloin alleviates epithelial injury during OGD/R partly through suppression of the IL-6/PNP axis.

Because oxidative stress is a central driver of reperfusion-associated inflammatory injury, we next evaluated intracellular ROS accumulation and downstream NF-κB/NLRP3 signaling [18]. Barbaloin markedly reduced OGD/R-induced ROS generation in a dose-dependent manner (Figure 7F,G). In parallel, barbaloin inhibited NF-κB p65 phosphorylation and nuclear translocation (Figure 7H–J), accompanied by reduced expression of key inflammasome-related proteins, including NLRP3, cleaved caspase-1, and IL-1β (Figure 7I,K–M). Collectively, these results indicate that barbaloin attenuates OGD/R-induced epithelial injury by suppressing ROS-mediated NF-κB/NLRP3 inflammatory activation downstream of the IL-6/PNP axis.

### 2.8. Barbaloin Alleviates Acute Lung Ischemia-Reperfusion Injury in Mice

**Figure 8:**
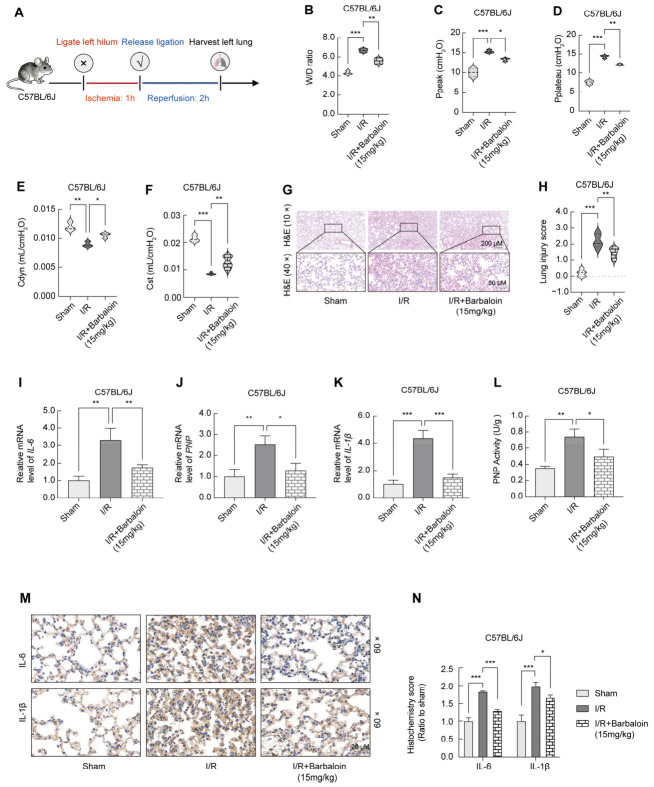
Barbaloin alleviates acute lung ischemia-reperfusion injury in mice by suppressing the IL-6/PNP axis. (**A**) Schematic diagram of the mouse lung ischemia-reperfusion (I/R) model. Barbaloin (15 mg/kg) was administered intraperitoneally 30 min before ischemia. (**B**) Lung wet-to-dry (W/D) ratio as an indicator of pulmonary edema. Barbaloin significantly attenuated the increase in lung water content induced by I/R injury. (**C**–**F**) Pulmonary function parameters: peak airway pressure (Ppeak, (**C**)), plateau pressure (Pplateau, (**D**)), dynamic compliance (Cdyn, (**E**)), and static compliance (Cst, (**F**)). I/R-induced elevations in airway pressures and reductions in lung compliance were partially restored by barbaloin. (**G**) Representative hematoxylin and eosin (H&E) staining of lung tissues (scale bar: 200 μm for ×10 magnification, 50 μm for ×40 magnification). I/R injury induced alveolar edema, inflammatory cell infiltration, hemorrhage, and structural disruption, which were alleviated by barbaloin. (**H**) Semi-quantitative lung injury score based on H&E staining. (**I**–**K**) Relative mRNA expression levels of (**I**) IL-6, (**J**) PNP, and (**K**) IL-1β in lung tissues measured by qRT-PCR. Barbaloin suppressed reperfusion-induced upregulation of these inflammatory mediators. (**L**) PNP enzymatic activity in lung tissues. (**M**) Representative immunohistochemical staining images of IL-6 and IL-1β in lung tissues (scale bar: 50 μm) (Data are presented as mean ± SD. (**N**) Quantitative analysis of immunohistochemical staining intensity, normalized to the Sham group. All data are presented as mean ± SD (*n* = 6 per group). * *p* < 0.05, ** *p* < 0.01, *** *p* < 0.001.

To evaluate the protective effect of barbaloin against LIRI, mice were subjected to 1 h of ischemia followed by 2 h of reperfusion [19], with barbaloin (15 mg/kg) administered intraperitoneally 30 min before ischemia (Figure 8A). Ischemia/reperfusion (I/R) injury significantly increased the lung wet-to-dry (W/D) ratio, a hallmark of pulmonary edema, which was markedly attenuated by barbaloin treatment (Figure 8B). Functional assessment via mechanical ventilation further revealed that barbaloin partially reversed I/R-induced elevations in peak airway pressure (Ppeak) and plateau pressure (Pplateau) while restoring dynamic lung compliance (Cdyn) and static lung compliance (Cst); Figure 8C–F, demonstrating preserved pulmonary mechanics.

Histopathology revealed alveolar edema, inflammatory infiltration, hemorrhage, and hyaline membrane formation after I/R, which were markedly ameliorated by barbaloin treatment, as confirmed by semi-quantitative lung injury scores (Figure 8G,H).

Molecular validation via qRT-PCR and enzymatic assays confirmed that barbaloin effectively suppressed the I/R-induced upregulation of *IL-6*, *PNP*, and *IL-1β* mRNA, while inhibiting elevated PNP catalytic activity (Figure 8I–L). Consistently, immunohistochemical staining demonstrated reduced expression of IL-6 and IL-1β in the lung parenchyma following barbaloin administration (Figure 8M,N). Collectively, these in vivo results demonstrate that barbaloin attenuates IL-6/PNP-mediated inflammatory-metabolic crosstalk and ROS-dependent signaling, thereby ameliorating structural injury and preserving pulmonary mechanics. These findings support barbaloin as a promising dual-target therapeutic candidate for mitigating acute lung IRI in the context of transplantation.

### 2.9. ADMET and Drug-likeness Profiling of Barbaloin

To evaluate the pharmacokinetic and safety profile of barbaloin, we conducted in silico ADMET profiling and drug-likeness assessment (Table 1, Table 2 and Table 3), providing early predictions of bioavailability, metabolic stability, and potential toxicity to guide future preclinical development.

ADMET analysis showed that barbaloin complies with Lipinski’s rule of five, with high predicted intestinal absorption (96.3%) and low CYP450 interaction risk. Despite high plasma protein binding (93.54%) and a short, predicted half-life (0.635 h), its overall drug-likeness was supported by favorable synthetic accessibility and MCE-18 scores. However, predicted risks of drug-induced liver injury and mutagenicity warrant careful preclinical safety evaluation. Together, these results indicate that barbaloin possesses promising drug-like properties while underscoring the need for targeted optimization before therapeutic application.

## 3. Discussion

Our translational strategy utilized a top-down, human-to-rodent flow. By first mapping key regulatory hubs in human clinical LIRI specimens, we ensured clinical relevance. Subsequent functional validation in our murine model confirmed that targeting this human-derived IL-6/PNP axis yields significant physiological and structural preservation in vivo.

Early LIRI is characterized by the concurrent activation of inflammatory cascades and disruption of metabolic homeostasis. However, therapeutic strategies capable of simultaneously modulating these interconnected processes remain limited. By integrating human bulk and single-cell transcriptomics with network pharmacology, molecular modeling, and experimental validation, the present study identifies the IL-6/PNP axis as a key regulatory axis involved in early reperfusion injury and supports barbaloin as a dual-target modulator capable of suppressing both inflammatory signaling and ROS-associated metabolic stress.

Transcriptomic profiling of reperfused human lung allografts revealed broad activation of inflammatory pathways, including IL-17, TNF, and NF-κB signaling, together with suppression of multiple metabolic programs, underscoring the substantial bioenergetic burden imposed by acute reperfusion [20,21]. Integrative screening across DEGs, phenotype-associated co-expression modules, lung transplantation-related genes, and predicted barbaloin targets converged on IL-6 and PNP as core hub genes with high network centrality and strong diagnostic performance. The simultaneous emergence of these two molecules is mechanistically notable because it links canonical cytokine-driven inflammation with purine metabolic reprogramming, suggesting that oxidative stress during reperfusion is sustained through coordinated inflammatory-metabolic crosstalk rather than isolated signaling events [22]. This dual-hit signature provided the rationale for further mechanistic dissection and therapeutic targeting.

IL-6 and PNP appear to converge mechanistically on the amplification of oxidative stress during early reperfusion. PNP, a key enzyme in purine salvage, is sharply upregulated during ischemia; its catabolic activity generates hypoxanthine, which is rapidly oxidized by xanthine oxidase upon reperfusion, producing a substantial ROS burst [23]. In parallel, IL-6 enhances oxidative stress through mitochondrial dysfunction and NADPH oxidase activation [24,25]. These complementary processes establish a feed-forward oxidative-inflammatory circuit that drives NF-κB/NLRP3 inflammasome activation and downstream tissue injury [26]. Single-cell transcriptomic mapping further highlighted distinct cellular contributions to this process, with IL-6 predominantly enriched in endothelial and myeloid populations, whereas PNP showed broader expression across both structural and immune cell compartments [27]. Together, these observations support a model in which inflammatory signaling and metabolic ROS production cooperate to orchestrate the early phase of graft reperfusion injury.

To establish this therapeutic efficacy, molecular docking and 200 ns molecular dynamics simulations revealed stable interactions between barbaloin and both IL-6 and PNP, providing computational evidence for its dual-target engagement [28]. In BEAS-2B cells subjected to oxygen-glucose deprivation/reoxygenation (OGD/R), barbaloin treatment not only improved cell viability but also suppressed IL-6 and PNP expression, reduced intracellular ROS accumulation, and attenuated NF-κB/NLRP3 inflammasome activation. These in vitro observations were further validated in a mouse model of LIRI. Specifically, barbaloin administered prior to ischemia significantly blunted reperfusion-induced upregulation of IL-6, PNP, and IL-1β, alleviated pulmonary edema, and ameliorated histopathological lung injury. Collectively, these findings suggest that simultaneous modulation of inflammatory and metabolic ROS-generating pathways may confer broader protection against acute reperfusion injury compared to single-pathway intervention alone.

Several limitations warrant consideration. First, the current mouse lung IRI model and BEAS-2B OGD/R system represent hyper-acute experimental settings designed to capture immediate early transcriptional responses and acute target engagement. However, they do not fully reproduce the prolonged cold ischemia, alloimmune activation, microvascular thrombosis, or systemic inflammatory complexity characteristic of clinical PGD [29,30]. Second, although docking simulations support barbaloin’s dual-target engagement, direct biophysical validation assays, such as surface plasmon resonance (SPR) and isothermal titration calorimetry (ITC), are still required to definitively characterize binding affinity and kinetics [31,32].

Translation toward clinical application requires addressing several pharmacokinetic and safety constraints. ADMET predictions for barbaloin indicate potential risks of DILI and mutagenicity. However, these in silico alerts are preliminary screening indicators rather than definitive evidence of toxicity and therefore require experimental validation. [33]. Additionally, drug-drug interactions with post-transplant antimetabolites (e.g., mycophenolate mofetil or azathioprine) warrant careful consideration due to overlapping purine-pathway modulation [34]. Localized delivery via EVLP or nebulization offers a practical strategy: restricting barbaloin exposure to the lung graft achieves high regional bioavailability at the site of injury while minimizing systemic exposure, thereby reducing both systemic toxicity and the risk of drug-drug interactions [35,36]. Beyond these pharmacokinetic considerations, comprehensive safety evaluation remains necessary for future translation. Since the current study focused on acute target engagement and early functional protection, longer-term studies incorporating repeated dosing, multi-organ toxicity assessment, immune profiling, and standard mutagenicity testing will be required to fully define the safety profile of barbaloin in the transplantation setting.

Compared to existing strategies, such as tocilizumab (IL-6 blockade) or emerging single-target PNP inhibitors, barbaloin’s dual-target capacity offers a more holistic intervention [16,37,38]. By concurrently targeting the metabolic source of ROS (PNP) and the inflammatory amplifier (IL-6), barbaloin interrupts a self-sustaining injury circuit. As a small molecule, it may also provide superior tissue penetration into the ischemic graft compared to larger biological agents [39].

Taken together, these findings position the IL-6/PNP axis as a potential regulatory hub in early LIRI and support simultaneous inflammatory-metabolic modulation as a rational therapeutic paradigm for reperfusion injury. By suppressing ROS accumulation and downstream NF-κB/NLRP3 inflammasome activation, barbaloin supports protective effects across both cellular and in vivo models of lung reperfusion injury. Future efforts focused on scaffold optimization, long-term safety evaluation, physiologically relevant transplantation models, and EVLP-based delivery strategies will be important for advancing this dual-target approach toward clinical translations.

## 4. Materials and Methods

### 4.1. Integrated Bulk Transcriptomic Analysis

This study integrated transcriptomic data from two independent datasets on human lung transplantation (GSE127003 with 46 sample pairs and GSE145989 with 67 sample pairs) [40,41]. Peripheral lung biopsies were collected at the end of cold ischemia and after 1–2 h reperfusion, yielding a total of 113 matched sample pairs. Raw expression matrices were merged and normalized, followed by batch effect correction using the “sva” package (version 3.54.0) to minimize inter-dataset variability before downstream analyses.

### 4.2. Identification of Differentially Expressed Genes (DEGs) in Lung Transplantation

To identify transcriptional alterations associated with early reperfusion injury, differential expression analysis was performed between cold ischemia and 1–2 h reperfusion samples using the “limma” package (version 3.62.2). Genes with |log2 fold change| ≥ 0.585 and adjusted *p*-value (q value) < 0.05 were considered significantly differentially expressed.

### 4.3. Weighted Gene Co-Expression Network Analysis (WGCNA)

WGCNA was performed using the “WGCNA” package (version 1.73). A scale-free network was constructed by selecting the optimal soft-thresholding power (β) based on the scale-free topology fit index (R^2^ > 0.85). Module identification was performed using hierarchical clustering and the dynamic tree-cutting algorithm (minimum module size = 30). Modules significantly associated with lung transplantation status were selected based on module membership (MM) and gene significance (GS). Genes from phenotype-associated modules were merged for downstream analysis.

### 4.4. Retrieval of Lung Transplantation-Related Genes from Public Databases

Lung transplantation-related genes were retrieved by querying the GeneCards (https://www.genecards.org/, accessed on 15 June 2025) [42] and OMIM databases (https://www.omim.org/, accessed on 15 June 2025) [43] using the keyword “Lung transplantation”, where results from GeneCards were filtered with a relevance score threshold of ≥5, followed by taking the union of the identified gene sets from both sources to generate a comprehensive reference list.

### 4.5. Collection of Barbaloin Targets

To comprehensively predict the putative targets of barbaloin, canonical SMILES and SDF structures were obtained from PubChem (https://pubchem.ncbi.nlm.nih.gov/, accessed on 15 June 2025) [44]. Target prediction was performed using four independent platforms, including PharmMapper (https://www.lilab-ecust.cn/pharmmapper/, accessed on 15 June 2025) [45], SEA (https://sea.bkslab.org/, accessed on 15 June 2025) [46], SuperPred (https://prediction.charite.de/) [47], and SwissTargetPrediction (http://swisstargetprediction.ch/, accessed on 15 June 2025) [48], to integrate complementary ligand-based and pharmacophore-based prediction algorithms. Predicted targets from all platforms were standardized using UniProt identifiers, merged, and deduplicated to generate a comprehensive candidate target set.

### 4.6. Four-Set Intersection Analysis for Screening Key Target Genes

Candidate therapeutic targets were screened by intersecting four datasets: (1) DEGs identified from reperfusion samples, (2) WGCNA-derived module genes, (3) lung transplantation-related genes from public databases, and (4) predicted barbaloin targets. Overlapping genes were visualized using Venn analysis and subsequently imported into Cytoscape for construction of the barbaloin-target-lung transplantation interaction network [49].

### 4.7. Characterization of Key Targets: Expression, Diagnostic Evaluation, and PPI Network

Expression differences of candidate genes were visualized using the “ggplot2” package (version 3.5.2). Receiver operating characteristic (ROC) analysis was performed using the “pROC” package (version 1.18.5) to evaluate diagnostic performance, and area under the curve (AUC) values were calculated. Protein-protein interaction (PPI) analysis was performed using the STRING database (https://cn.string-db.org, accessed on 15 June 2025) with a minimum interaction score threshold of 0.4.

### 4.8. Integrative Screening for Core Hub Genes

Core hub genes were prioritized using an integrative strategy combining network topology and diagnostic performance. Genes were retained if they simultaneously exhibited centrality within the PPI network and strong discriminatory ability in ROC analysis (AUC > 0.85). Based on these criteria, IL-6 and PNP were selected for subsequent validation.

### 4.9. ssGSEA and GSEA Analyses of Core Hub Genes

ssGSEA was performed using the “GSVA” package (version 1.50.0) to estimate the enrichment scores of 28 immune cell types in each sample, using the immune cell gene set from the Molecular Signatures Database (MSigDB, version 7.5.1). Spearman correlation analysis was conducted to assess the relationship between the expression levels of core hub genes and immune cell infiltration scores in the 1–2 h reperfusion group, with a significance threshold of *p* < 0.05. GSEA was performed using the “clusterProfiler” package (version 4.10.0) to identify biological pathways associated with each core hub gene. Samples were divided into high-expression and low-expression groups based on the median expression of each hub gene. GSEA was run with 1000 permutations, and pathways with an FDR < 0.25 and normalized enrichment score (NES) > 1.5 were considered significantly enriched [50].

### 4.10. Single-Cell Sequencing Analysis

Single-cell RNA sequencing (scRNA-seq) was performed on lung biopsies from six lung transplant recipients collected after cold preservation and 2-h reperfusion (GSE220797) [27]. Data were processed using the “Seurat” package (version 5.2.1). Quality filtering retained genes detected in ≥3 cells and cells expressing 200–6000 genes with <20% mitochondrial reads. Data were normalized, and the top 2000 variable genes were selected for downstream analysis. Dimensional reduction (PCA), graph-based clustering (resolution = 0.5), and cell-type annotation using established marker genes were subsequently performed.

### 4.11. Molecular Docking

Molecular docking was performed to evaluate the potential binding interactions between barbaloin and candidate hub proteins. Barbaloin was energy-minimized using the MM2 force field in ChemBio3D (version 14.0). Crystal structures of IL-6 (PDB: 1ALU) and PNP (PDB: 1M73) were obtained from the RCSB Protein Data Bank. Docking simulations were carried out using CB-Dock2 [51,52], which automatically detects potential binding cavities and estimates binding affinity. The docking conformation with the lowest predicted binding energy was selected for visualization and interaction analysis.

### 4.12. Molecular Dynamics Simulation

To further assess binding stability, a 200 ns molecular dynamics MD simulation was performed using GROMACS (version 2023.2) with the CHARMM36 force field to assess the stability of the PNP-barbaloin and IL-6-barbaloin complexes. Systems were prepared using the CHARMM-GUI server [53], solvated in a TIP3P water box, neutralized with NaCl, and simulated under NPT conditions (1 atm, 310 K) with a 2 fs time step. Structural stability and conformational dynamics were evaluated by RMSD, RMSF, and Rg analyses [54,55].

### 4.13. Cell Culture and Hypoxia/Reoxygenation Model

To simulate LIRI in vitro, an OGD/R model was established. Human bronchial epithelial cells (BEAS-2B, ATCC CRL-9609, Manassas, VA, USA) were pretreated with barbaloin (0–100 μM, MedChemExpress, HY-N0123, Monmouth Junction, NJ, USA) and then subjected to 12 h of hypoxia (1% O_2_, 5% CO_2_, balanced with N_2_) in glucose-free, serum-free DMEM containing the same concentrations of barbaloin, followed by 8 h of reoxygenation in complete DMEM supplemented with 10% fetal bovine serum (FBS, 10099141, Gibco, Grand Island, NY, USA) under normoxic conditions (21% O_2_, 5% CO_2_), with barbaloin also present throughout the reoxygenation period [56].

### 4.14. Lung Ischemia-Reperfusion Mouse Model

A murine warm ischemia-reperfusion (I/R) model was established to evaluate the in vivo protective effect of barbaloin against acute lung ischemia-reperfusion injury (LIRI).

Male C57BL/6J mice (8 weeks old, 20–22 g) were obtained from The Jackson Laboratory and acclimatized for 7 days before experimentation. A total of 18 mice were allocated into three groups (*n* = 6 per group): Sham, I/R, and Barbaloin (15 mg/kg, intraperitoneal injection administered 30 min before ischemia).

Mice were housed under specific pathogen-free conditions and anesthetized with pentobarbital sodium (40 mg/kg, i.p.), followed by maintenance with 1% isoflurane. Body temperature was maintained throughout the procedure using a thermostatically controlled surgical table. After endotracheal intubation, mice were mechanically ventilated with room air at a respiratory rate of 120 breaths/min, an inspiratory-to-expiratory (I:E) ratio of 1:2, tidal volume (TV) of 6 mL/kg, and positive end-expiratory pressure (PEEP) of 2 cm H_2_O. Heparin sodium (100 U/kg) was administered via tail vein injection 5–10 min before surgery.

Following left thoracotomy, the left pulmonary hilum was reversibly ligated using a 6–0 silk suture to induce 1 h of warm ischemia, confirmed by lung darkening, collapse, and cessation of ventilation and pulsation. Reperfusion was initiated by releasing the ligature and maintained for 2 h [57]. At the end of reperfusion, mice were euthanized by pentobarbital overdose and left lung tissues were harvested for subsequent analyses.

### 4.15. In Vitro and In Vivo Functional Assays

Cell viability, intracellular ROS levels, and PNP enzymatic activity were assessed using the CCK-8 assay (Beyotime Biotechnology, C0037, Shanghai, China), DCFH-DA fluorescence staining (Beyotime Biotechnology, S1105S, Shanghai, China), and a commercial PNP activity kit (Solarbio Life Sciences, BC6350, Beijing, China), respectively, all following the manufacturer’s instructions. For ROS measurement, cells were incubated with DCFH-DA for 20 min at 37 °C, and fluorescence was analyzed using ImageJ (version 1.54i).

For gene expression analysis, qRT-PCR was performed using a SYBR Green Fast qPCR Mix (Abclonal, RK21203, Wuhan, China) following the manufacturer’s instructions. Total RNA was extracted with TRIzol reagent (Thermo Fisher Scientific, 15596026, Waltham, MA, USA), and cDNA was synthesized using a reverse transcription kit (Abclonal, RK20428, Wuhan, China). GAPDH served as the endogenous control, and relative expression was calculated via the 2^−ΔΔCt^ method [58]. Protein expression and localization were evaluated via western blot and IF assays, with targets including key components of the NF-κB/NLRP3 pathway (P-NF-κB p65, NF-κB p65, NLRP3, IL-1β, and cleaved caspase-1). Band intensities from western blot were quantified using Quantity One (version 4.62), and IF fluorescence intensity was quantified using ImageJ (version 1.54i). Detailed experimental procedures were performed as previously described [59]. Detailed antibody information and primer sequences are provided in Supplementary Tables S4 and S5.

For histopathological and immunohistochemical evaluation, lung tissues were fixed, paraffin-embedded, and sectioned (4 μm). Sections were stained with hematoxylin-eosin (H&E) to assess general tissue morphology and with immunohistochemistry (IHC) to detect IL-6 and IL-1β protein expression. Lung injury on H&E sections was scored blindly by two pathologists based on alveolar edema, inflammatory cell infiltration, hemorrhage, and hyaline membrane formation (each 0–4), with the final score calculated as the average of the four parameters. IHC staining was quantified using the Histochemistry-score method (SlideViewer, version 2.4), calculated as ∑(pi × i), where i indicates staining intensity (0–3: negative, weak, moderate, strong) and pi is the corresponding percentage of positive cells. The final score (0–300) integrates both intensity and extent of staining, providing a semi-quantitative measure of protein expression [60].

Pulmonary edema was assessed by the lung wet-to-dry (W/D) ratio. The left lung was excised after reperfusion, weighed to obtain the wet weight, and dried at 60 °C until a constant weight was reached to determine the dry weight. Pulmonary function was evaluated under mechanical ventilation at the end of reperfusion using a small-animal ventilator, including measurement of Ppeak, Pplateau, Cdyn, and Cst. After stabilization of the respiratory waveform, a 1 s inspiratory hold maneuver was applied to induce a brief breath hold. Ppeak was recorded as the maximal airway pressure during ventilation, whereas Pplateau was determined from the stabilized pressure during the inspiratory hold. Lung compliance was calculated as follows: Cst = TV/(Pplateau − PEEP) and Cdyn = TV/(Ppeak − PEEP) [61].

### 4.16. ADMET Property Prediction

To preliminarily evaluate the translational potential and safety profile of barbaloin, ADMET properties were predicted using the ADMETlab 2.0 platform (https://admetmesh.scbdd.com/, accessed on 20 June 2025) [62]. Parameters related to absorption, distribution, metabolism, excretion, and toxicity were calculated using the default prediction models based on the SMILES structure of barbaloin.

### 4.17. Statistical Analysis

Data analysis was performed using R (version 4.4.0) and GraphPad Prism (version 9.0). Experimental data are presented as mean ± standard deviation (SD). For in vitro experiments, values represent three independent biological replicates. For in vivo experiments, the exact sample size for each group is indicated in the corresponding figure legends. Normality and homogeneity of variance were assessed prior to statistical analysis. When assumptions were satisfied, comparisons among three or more groups were performed using one-way analysis of variance (ANOVA) followed by Tukey’s multiple comparisons test. Statistical significance was defined as * *p* < 0.05. Symbols indicate significance levels as follows: * *p* < 0.05, ** *p* < 0.01, *** *p* < 0.001; ns, not significant.

## 5. Conclusions

In conclusion, this study identifies the IL-6/PNP axis as a key inflammatory-metabolic axis involved in the early phase of lung ischemia-reperfusion injury. By integrating multi-omic screening with experimental validation, our findings support barbaloin as a promising dual-target lead compound capable of simultaneously modulating purine metabolic disruption and inflammatory signaling. Mechanistically, barbaloin attenuated ROS accumulation and downstream NF-κB/NLRP3 inflammasome activation, providing protective effects in both cellular and murine models of lung ischemia-reperfusion injury. Although further optimization and long-term safety evaluation are required, localized delivery strategies such as EVLP may enhance its translational potential for reducing primary graft dysfunction after lung transplantation.

## Figures and Tables

**Table 1:** Prediction of Toxicity Parameters of Barbaloin.

Propetry	Parameter	Values	Unit
Absorption	Caco-2 Permeability	−5.892	(Log Papp)
	MDCK Permeability	4 × 10^−^^6^	cm/s
	P-glycoprotein inhibitor	NO	categorical
	P-glycoprotein substrate	NO	categorical
	Human Intestinal Absorption	96.3	(% Absorbed)
Distribution	Volume Distribution	0.902	(L/kg)
	Plasma Protein Binding	93.54	(%)
	Blood-Brain Barrier Penetration	NO	categorical
	The fraction unbound in plasms	4.162	(%)
Metabolism	CYP1A2 inhibitor	NO	categorical
	CYP1A2 substrate	NO	categorical
	CYP2C19 inhibitor	NO	categorical
	CYP2C19 substrate	NO	categorical
	CYP2C9 inhibitor	NO	categorical
	CYP2C9 substrate	NO	categorical
	CYP2D6 inhibitor	NO	categorical
	CYP2D6 substrate	NO	categorical
	CYP3A4 inhibitor	NO	categorical
	CYP3A4 substrate	NO	categorical
Excretion	Clearance	6.166	(mL/min/kg)
	Half-life	0.635	(hour)
Toxicity	hERG Blockers	NO	categorical
	hERG Blockers (10 μm)	NO	categorical
	Drug Induced Liver Injuy	Yes	categorical
	AMES Toxicity	Yes	categorical
	Rat Oral Acute Toxicity	NO	categorical
	FDA Maximum (Recommended) Daily Dose	NO	categorical
	Skin Sensitization	Yes	categorical
	Respiratory Toxicity	NO	categorical

**Table 2:** Physicochemical Properties of Barbaloin.

Compounds	Formula	Mol Weight	Volume	nHA	nHD	nRot	nRing	nHet	fChar	nRig	TPSA
Barbaloin	C_21_H_22_O_9_	418.13	398.203	9	7	3	4	9	0	23	167.91

Note. The general recommended ranges are as follows: (a) Molecular weight, 100 to 600. (b) nHA-Number of H bond acceptors, <12. (c) nHD-Number of rotatable bonds, <7. (d) nRot-Number of rotatable bonds, <11. (e) nRing-Number of rings, <6. (f) nHet-Number of heteroatoms, 1 to 15. (g) fChar-Formal charge, −4 to 4. (h) nRig-Number of rigid bonds, <30. (i) TPSA-Topological Polar Surface Area, <140.

**Table 3:** Drug-likeliness Properties of Barbaloin.

Compounds	logP	logS	Lipinski Rule	Pfizer Rule	GSK Rule	Golden Triangle	PAINS	SAscore	MCE-18	Fsp3	GASA
Barbaloin	0.623	−1.916	Accepted	Accepted	Rejected	Accepted	0	4.173	88.759	0.381	1

Note. logP-Lipophilicity, logS-Water solubility, SAscore-Synthetic accessibility score, Fsp3-sp3 hybridized carbons.

## Data Availability

The data presented in this study are openly available in NCBI Gene Expression Omnibus (GEO) at the following links: https://www.ncbi.nlm.nih.gov/geo/query/acc.cgi?acc=GSE127003, accessed on 1 June 2025 (reference number GSE127003), https://www.ncbi.nlm.nih.gov/geo/query/acc.cgi?acc=GSE145989, accessed on 1 June 2025 (reference number GSE145989), https://www.ncbi.nlm.nih.gov/geo/query/acc.cgi?acc=GSE220797, accessed on 1 June 2025 (reference number GSE220797). [NCBI GEO] [https://www.ncbi.nlm.nih.gov/geo/query/acc.cgi?, accessed on 1 June 2025] [GSE127003; GSE145989; GSE220797].

## Supplementary Materials

The following supporting information can be downloaded at: https://www.mdpi.com/article/10.3390/ijms27125276/s1.

## Abbreviations

The following abbreviations are used in this manuscript:

IRI	Ischemia-reperfusion injury
ROS	Reactive oxygen species
IL-6	Interleukin-6
PNP	Purine nucleoside phosphorylase
PGD	Primary graft dysfunction
H/R	hypoxia/reoxygenation
GSEA	Gene set enrichment analysis
DEGs	Differentially expressed genes
WGCNA	Weighted gene co-expression network
GS	Gene significance
MM	Module membership
2D	Two-dimensional
SEA	Similarity Ensemble Approach
AUC	Area under the curve
PPI	Protein-protein interaction
ssGSEA	Single-sample gene set enrichment analysis
MD	Molecular docking
MDS	Molecular dynamics simulations
BEAS-2B	Human bronchial epithelial cells
DILI	Drug-induced liver injury
CI	Confidence interval
EVLP	Ex vivo lung perfusion
SPR	Surface plasmon resonance
TV	Tidal volume
PEEP	Positive end-expiratory pressure
Ppeak	Peak airway pressure
Pplateau	Plateau pressure
Cst	Static lung compliance
Cdyn	Dynamic lung compliance
CCK-8	Cell counting kit-8
H&E	Hematoxylin-eosin
IHC	Immunohistochemistry
W/D	Wet-to-dry
ADMET	Absorption, Distribution, Metabolism, Excretion, Toxicity
ROC	Receiver operating characteristic
scRNA-seq	Single-cell RNA sequencing
RMSD	Root-mean-square deviation
RMSF	Root-mean-square fluctuation
Rg	Radius of gyration
FBS	Fetal bovine serum
qRT-PCR	Quantitative real-time polymerase chain reaction
IF	Immunofluorescence
GAPDH	Glyceraldehyde-3-phosphate dehydrogenase
