# Supplementary material for: Barbaloin Alleviates Lung Ischemia-Reperfusion Injury by Dual-Targeting IL-6 and PNP
Source: Int J Mol Sci. 2026 Jun 10;27(12):5276. doi: 10.3390/ijms27125276 (PMC13300021; doi:10.3390/ijms27125276)
Supplement: Supplementary file 1 [file ijms-27-05276-s001.zip › Supplementary Material-Original Images for Blots or Gels or Microscopy.pdf]

Supplementary material-uncropped western blots:

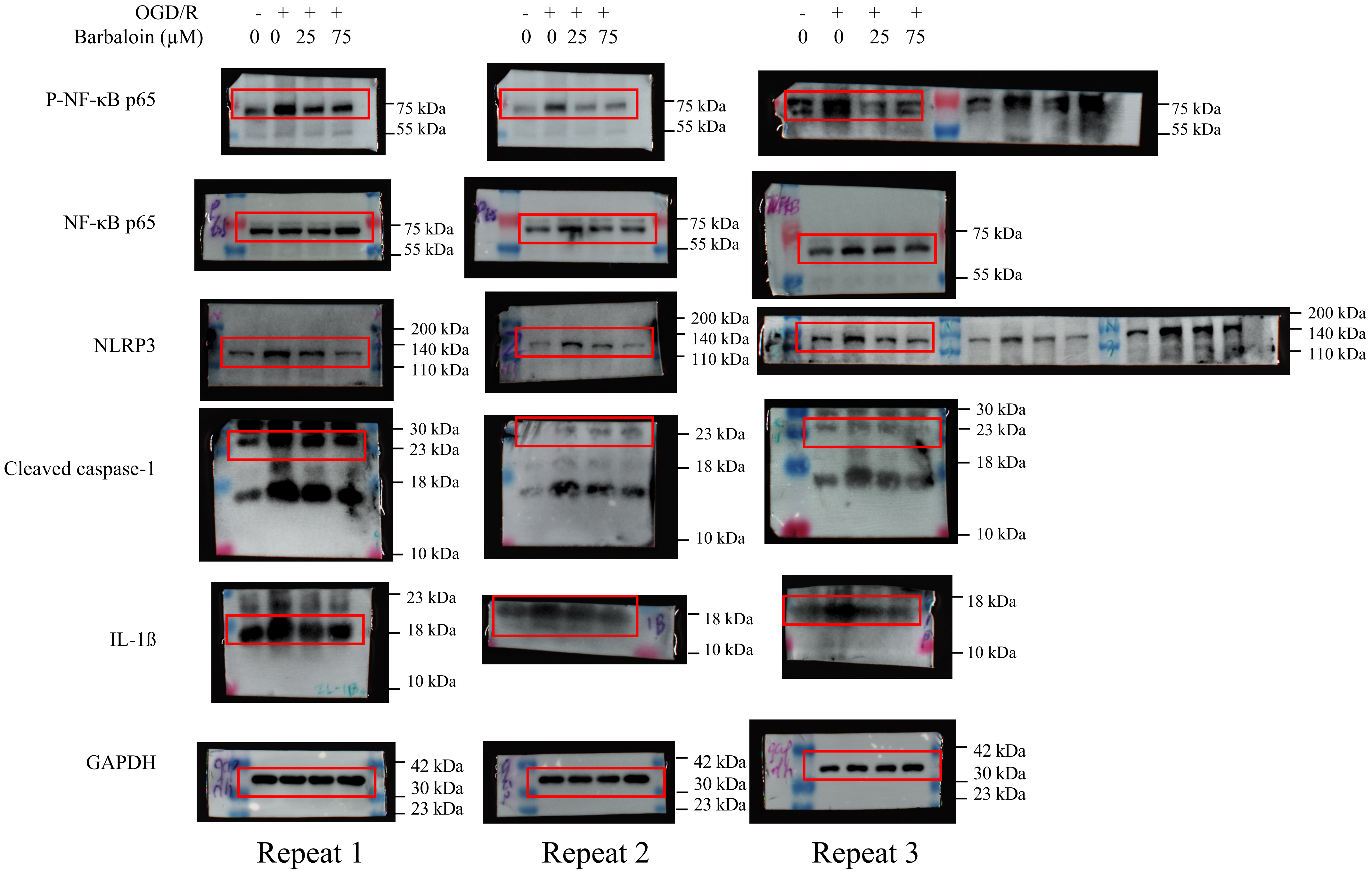

Supplementary Material - unadjusted images:

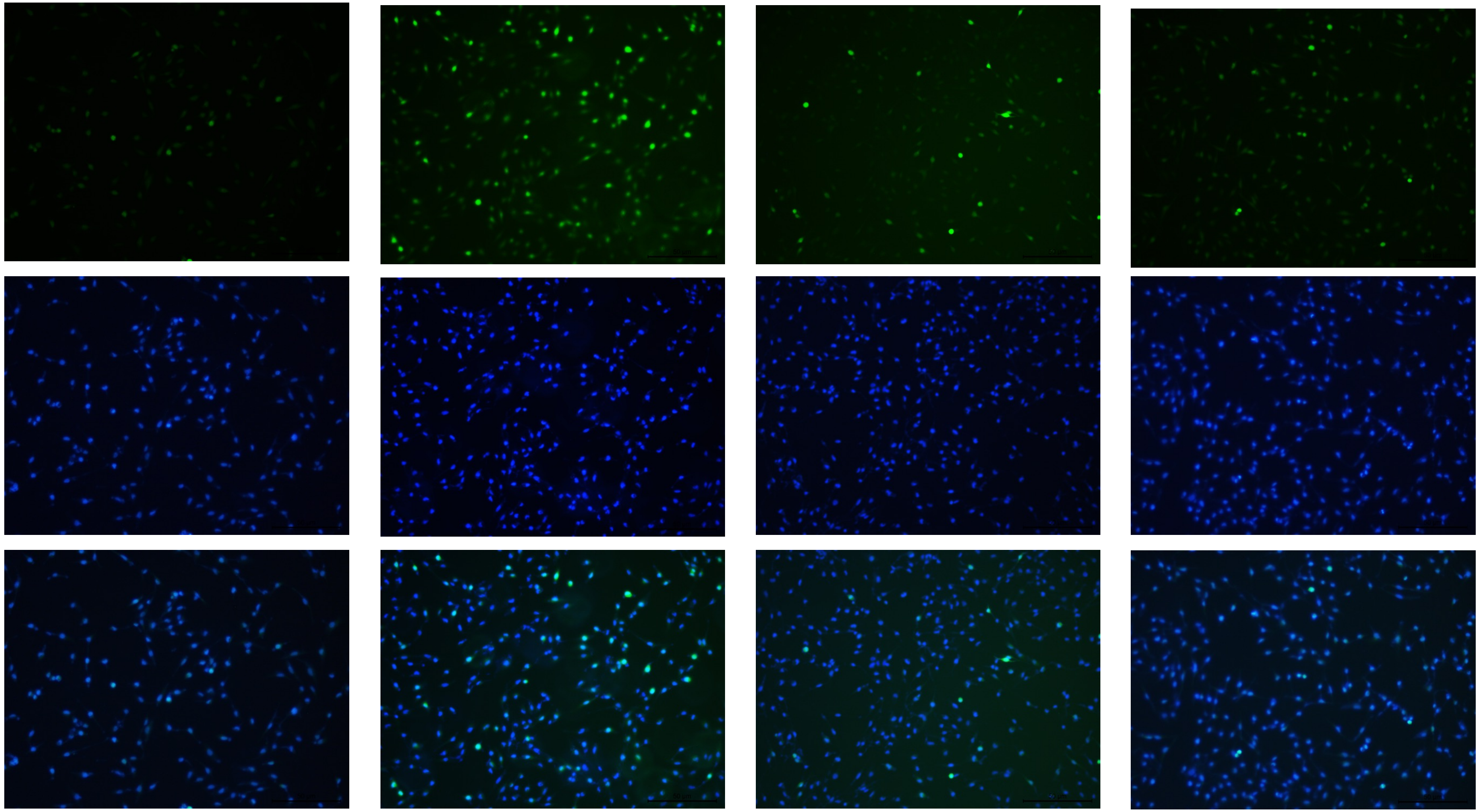

Supplementary Material - Original immunofluorescence images :

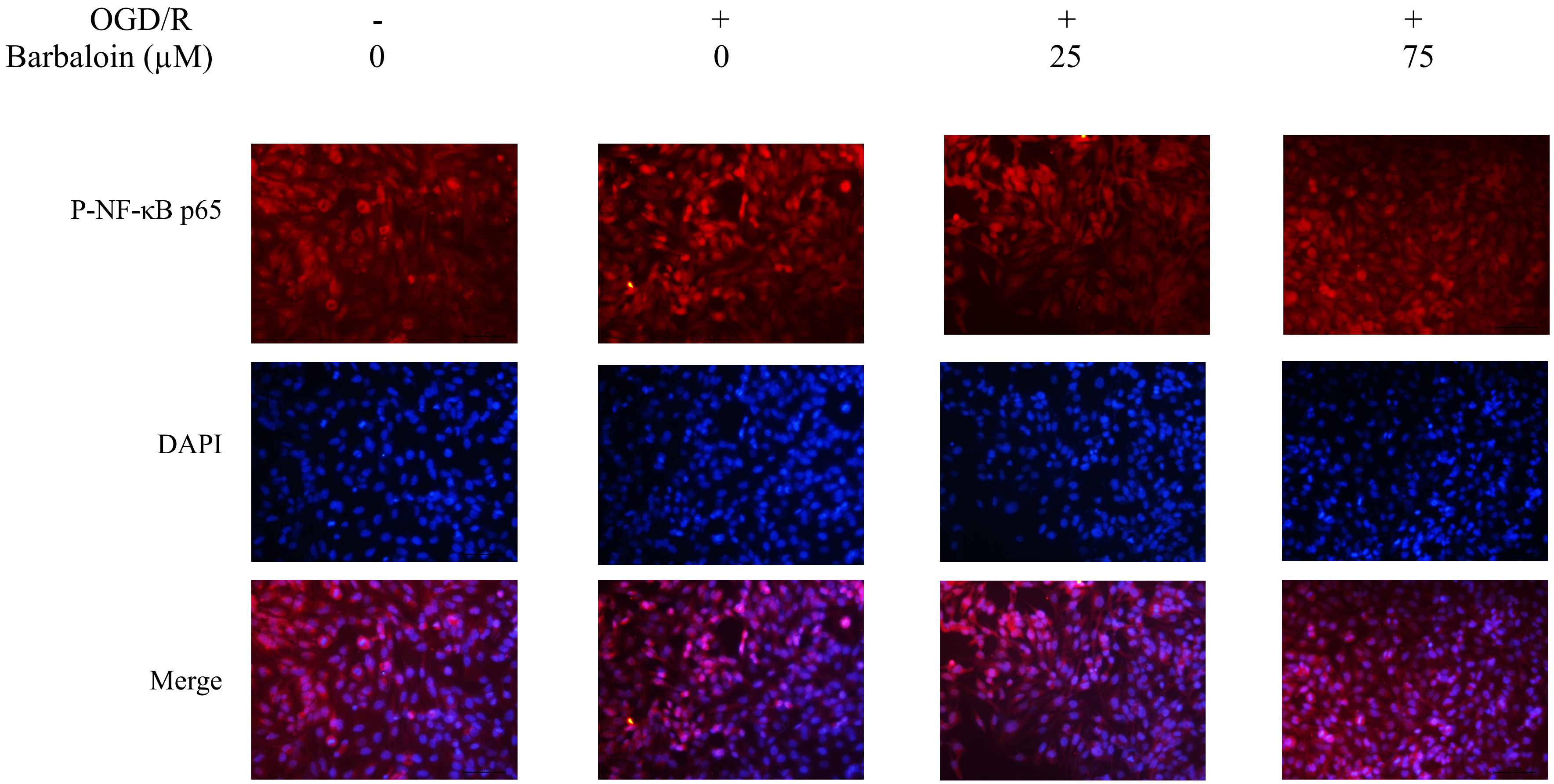

Supplementary Material - Original hematoxylin-eosin (H&E) staining images :

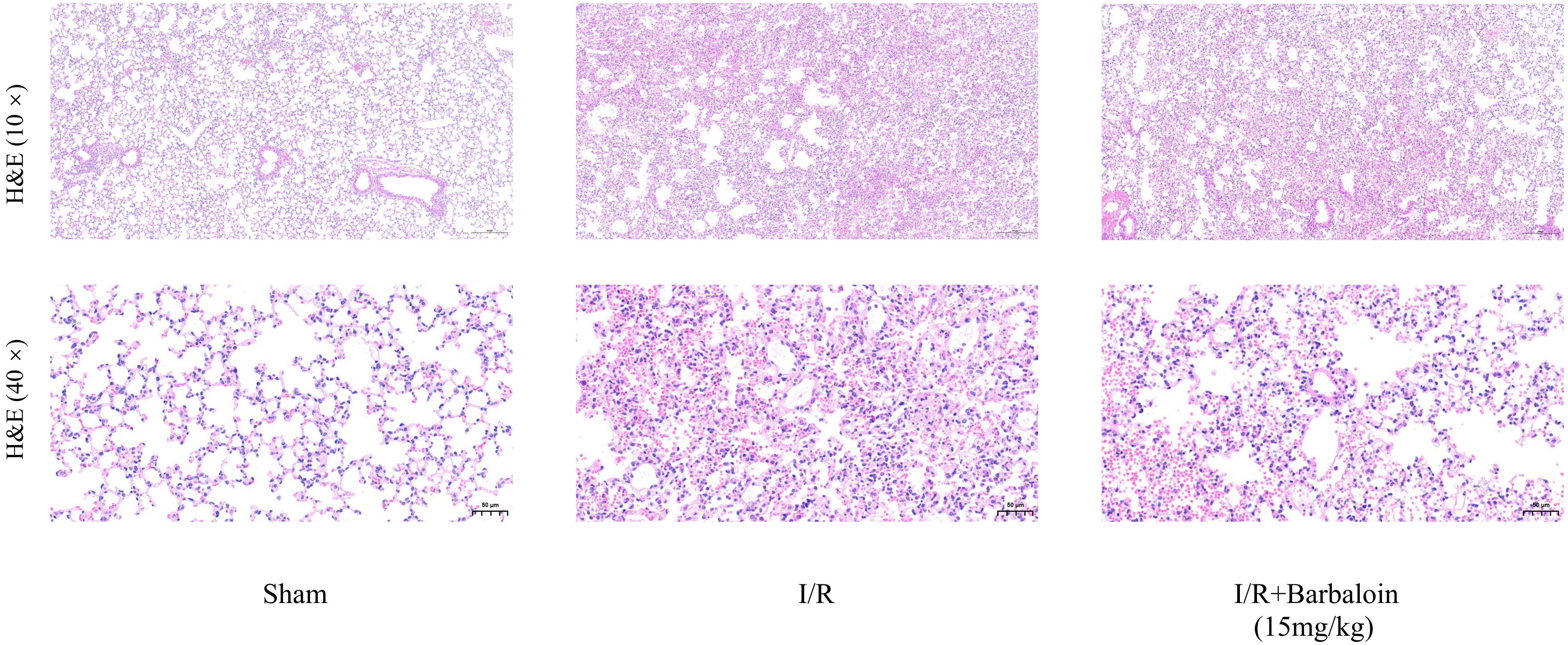

Supplementary Material – Original immunohistochemistry (IHC) staining images :

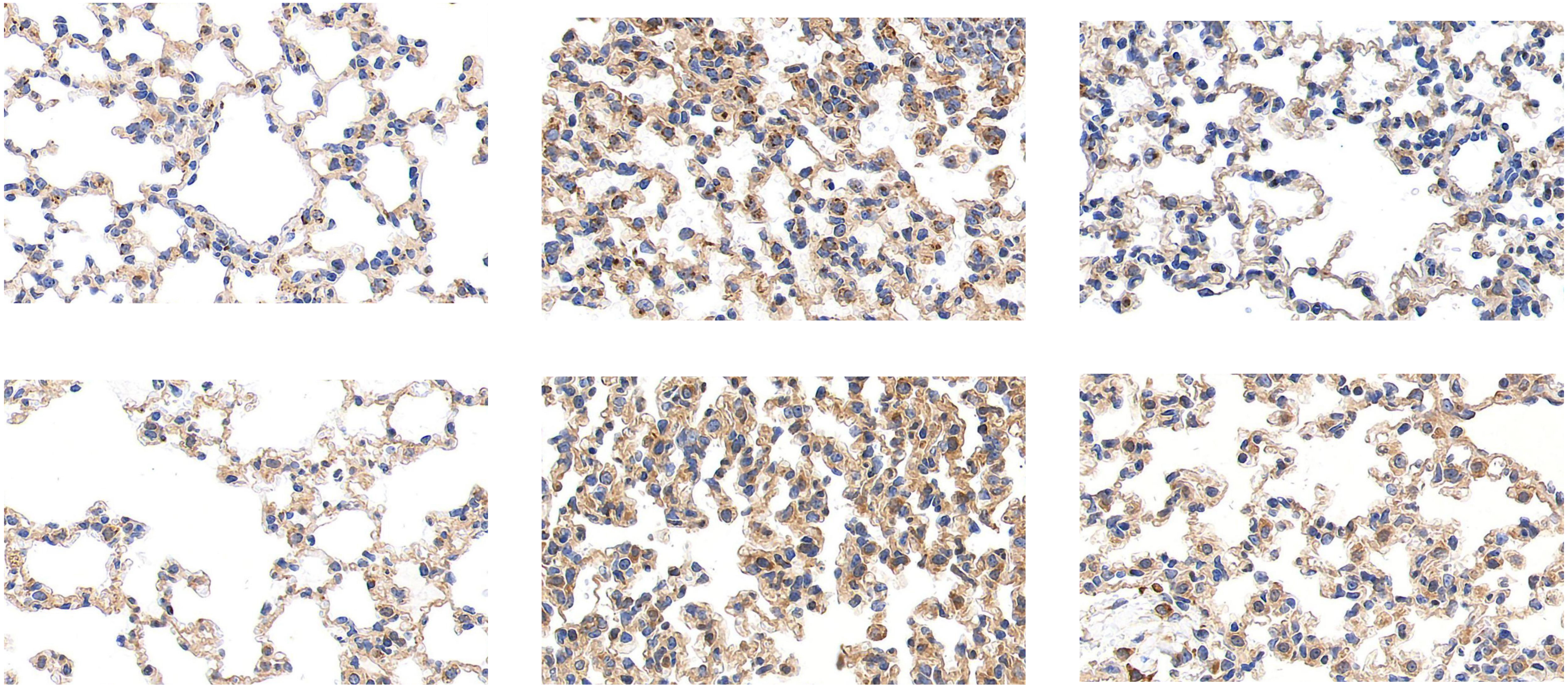

IL-6

IL-1β

Sham

I/R

I/R+Barbaloin
(15mg/kg)
