# Supplementary material for: Barbaloin Alleviates Lung Ischemia-Reperfusion Injury by Dual-Targeting IL-6 and PNP
Source: Int J Mol Sci. 2026 Jun 10;27(12):5276. doi: 10.3390/ijms27125276 (PMC13300021; doi:10.3390/ijms27125276)
Supplement: Supplementary file 1 [file ijms-27-05276-s001.zip › Supplementary Table S1.pdf]

Supplementary Table S1. Differentially Expressed Genes (DEGs) and Phenotype-Linked WGCNA Module Genes (MEyellow & MEturquoise)

DEGs	MEyellow	MEturquoise
PLAGL2	ADGRG3	ACKR1
RIPK2	ANKRD22	ADAMTS9-AS1
NFKBIE	AQP9	ADM
TNFAIP3	BCL2A1	AGTR2
GNA13	BTNL8	AMPD1
TICAM1	CAMP	ANGPTL4
KBTBD2	CCDC178	ANKRD1
ITPRIP	CD177	APOBEC3B
PMAIP1	CDA	ARC
JMJD6	CLC	ARID5A
RASGEF1B	CLEC4D	ATF3
PPP1R15A	CMTM2	ATP13A4-AS1
ZBTB43	CRISP3	B3GALT2
B3GNT5	CST7	BABAM2-AS1
IER5	CX3CR1	BAG3
NFKB2	CXCR1	BDKRB2
ARL5B	CXCR2	BIRC3
RLIM	CYTL1	C15orf48
ELL	DHRS9	C2CD4A
RLF	ENPP2	C9orf152
RNF148	FCAR	CA3
GADD45B	FCN1	CCK
SSH1	FFAR2	CCL11
ALMS1-IT1	FGFBP2	CCL2
GCH1	FOLR3	CCL20
MCL1	FPR2	CCL4
BAG3	GJB6	CCL5
CSF3	GLT1D1	CCL7
EPOP	GNLY	CCL8
IRF1	GPR84	CCN4
PELI1	GZMB	CD274
DNAJB1	GZMH	CD69
AGO2	IL18R1	CD83
MSX1	IL18RAP	CD8A
ZBTB2	IL1R2	CH25H
IER3	IL1RL1	CHI3L1
CCNH	KLB	CLDN1
SLC30A1	KLRF1	CSF3
SLC2A3	KRT23	CX3CL1
CCNL1	LCN2	CXCL1

SLC25A33
HSPB8
RELB
MT1F
CNKSR3
RRAD
KLHL15
ATF3
CD83
RNF19B
YOD1
ELF3
TIPARP
MT1HL1
DNAJA1
STX11
NFKBIZ
CHIC2
DUSP5
ABL2
UGCG
ATG4C
IRAK2
ICAM1
HOXD1
ARID5A
ZNF267
KDM6B
BCL10
MT1M
GABPB1
FOSL1
GUCY1A1
CXCL2
MT1G
CXCL8
SESN2
SOCS2
ZNF573
BIRC3
PPIF
ARC
FPGT

LMNB1
LRG1
LY6H
MGAM
MMP8
MMP9
MSLN
MYRIP
NFE2
NKG7
NTM
OLAH
ORM1
OSM
PADI4
PF4
PI3
PPBP
PRF1
PROK2
RGL4
RGS18
RIPOR2
RNASE2
RUBCNL
S100A12
S100P
SAMSN1
SELL
TMEM71
TOX3
TRIM58
VNN1
VNN2

CXCL10
CXCL11
CXCL3
CXCL5
CXCL8
CXCL9
DACT1
DKK1
DNAJA4
DNAJB1
DUSP2
DUSP5
EGR1
EGR2
EGR3
ELF3
EREG
ESM1
F8
FGA
FGFBP1
FGG
FNDC1
FOS
FOSB
FOSL1
G0S2
GADD45B
GALNT15
GCH1
GCLM
GDF10
GEM
GFPT2
GJB2
GPA33
GPIHBP1
GPR171
GPR34
GZMK
HAS1
HAS2
HBB

REL
ITPKC
CLCF1
KBTBD8
DUSP2
MT1X
PIM2
SERPINE1
SBNO2
SAMSN1
NOCT
IL1A
MAP3K8
PLK3
MAFF
ZFP36
MXD1
B4GALT5
ASH1L-AS1
CDKN1A
SOCS3
FOSL2
C3orf52
PPRC1
CXCL3
LIMK2
OSGEPL1
SPRY4
HSPA6
UPP1
VPS37B
SERTAD1
PHF13
TLNRD1
NFKB1
VMP1
EPHA2
PTGS2
NAMPT
GJB6
SLC25A25
IL1RAP
ZBTB21

HBD
HMOX1
HOXA5
HOXC6
HRCT1
HSD17B2
HSPA6
HSPH1
HTATSF1P2
ICAM1
ICAM4
IDO1
IER3
IFNG
IGHD
IGHM
IGLL3P
IL1A
IL1B
IL24
IL6
IRF1
ITGA2
ITLN1
ITPKC
JCHAIN
KCNN2
KDM6B
KRT80
LIF
LINC00844
LIPG
LRRC32
LRRN3
MEDAG
MEOX2
MIR3682
MIR3945HG
MMP10
MS4A15
MSX1
MT1F
MT1G

ZNF165
SPSB1
DCUN1D3
AREG
TIFA
CXCL1
HBB
GPR84
TMEM217
RALGDS
BBS10
DDIT3
EHHADH
PHLDA2
STK17A
DEDD2
PFKFB3
PHLDA1
HCG11
EREG
PANX1
PER2
NLRP3
BHLHE40
CHSY1
FCAR
IRX3
EFCAB7
PNP
PPP1R3C
ETS1
TOP1
PIM1
TP53INP2
BMP2
ST7-AS1
EID3
SLC25A37
HIVEP2
CLEC4D
SERPINB9
SPHK1
GADD45A
MT1M
MUC15
MZB1
NABP1
NFKBIE
NFKBIZ
NIM1K
NPY1R
NR4A2
OMD
P2RY12
PI15
PMAIP1
PNP
POU2AF1
PPIF
PPP1R15A
PRSS21
PTGS2
PTX3
RASGRP1
RASSF10
RELB
RND1
RNF148
RRAD
RTKN2
S100A3
SELE
SERPINB2
SERPINE1
SERTAD1
SERTM1
SFN
SGPP2
SHISA2
SLC10A4
SLC16A6
SLC30A1
SLC6A14
SLC7A11
SLC7A5
SMCO3

PLAUR
IL6
RHOH
TNF
SGMS2
MAP3K14
ZFAND2A
FSTL3
RUBCNL
EMP1
IL1B
GJB2
HOXA5
BAZ1A
STC2
CYTIP
TNFRSF10D
LRRK2
LRG1
PIGA
BABAM2-AS1
CRY1
CCL20
TNFRSF12A
DISP1
SLC20A1
CCDC71L
MIR3682
LIF
SERPINB2
TREML2
FFAR2
STC1
XIRP1
SERPINB1
RND1
TNFAIP6
SLC7A5
RGS16
RND3
GPR34
BATF3
TNFSF9

SOCS2
SOX7
SPHK1
SPINK1
SPSB1
STC1
STC2
STEAP4
TAC1
TDO2
TENM2
TFPI2
TIMP4
TMEM178A
TMEM217
TNC
TNF
TNFAIP3
TNFAIP6
TNFRSF11B
TNFRSF12A
TNFRSF17
TNFSF9
TNIP3
TSLP
UGT2B4
UTS2
VCAM1
YOD1
ZFAND2A
ZNF165
ZNF331
ZNF404
ZNF750
ZNF880

FAM222A
DLGAP1-AS2
GIMAP2
TUBB2A
G0S2
FPR2
CHORDC1
ADM
OSM
MYC
HAS2
EZH2
SOX7
B3GALT2
MIR3945HG
FAM13C
CX3CL1
BATF
CD274
SNAI1
SGPP2
BCL2A1
IL24
GPR183
ADGRG3
CSRNP1
C9orf152
IRX5
HSPH1
PLEK
CH25H
HRH1
PDE4B
RDH10
MSC
JUNB
IL32
FAM110C
PADI4
GZMB
CDA
LMNB1
PTX3

PLEKHG1
CRISP3
DUSP14
IL1RN
SH3RF3-AS1
CCL2
ANGPTL4
IL18R1
IL18RAP
CCL4
TSLP
RGL4
MEOX2
SAMD13
S100P
ADAMTS9
CXCR1
IFNG
SPIN4
LRRC32
RBMS3-AS3
DNAJA4
CMTM2
HMOX1
THBD
DACT1
VCAM1
BYSL
FPR1
RASGRP1
FIBIN
PANK1
CCL11
LIPG
GCLM
SLC19A2
SLC6A14
HBD
ADORA3
STEAP4
ZNF404
C2CD4A
HEY1

SFN
NABP1
CLC
TNFRSF10C
ICAM4
GNLY
ZNF750
ANKRD22
TMEM71
RIPOR2
C15orf48
GEM
ART4
SLC16A6
AQP9
SELE
PRF1
SHISA2
CITED1
IL1R2
CLDN1
TRIL
ADAMTS9-AS1
SLC7A11
FOSB
CNTN3
CXCR2
FCN1
NFE2
SELL
GFPT2
APOBEC3B
TNIP3
CXCL5
VNN1
MMP9
CCL7
CCDC178
TNFRSF17
FOS
TFPI2
VNN2
PI3

RARRES1
PF4
RUNDC3B
S100A12
RGS18
IL1RL1
FGFBP2
SLITRK6
PPBP
TMEM100
MGAM
PROK2
MMP1
SRGAP3-AS2
SOSTDC1
MMP8
TAC1
CEACAM8
OLFM4
ANKRD1
SNTN
