# Supplementary material for: Barbaloin Alleviates Lung Ischemia-Reperfusion Injury by Dual-Targeting IL-6 and PNP
Source: Int J Mol Sci. 2026 Jun 10;27(12):5276. doi: 10.3390/ijms27125276 (PMC13300021; doi:10.3390/ijms27125276)
Supplement: Supplementary file 1 [file ijms-27-05276-s001.zip › Supplementary Table S2.pdf]

Supplementary Table S2. Lung Transplantation-Related Genes Curated from GeneCards and OMIM Databases

Only GeneCards	Only OMIM	GeneCards and OMIM
EGFR	FMO2	EGFR
TERT	FASLG	TERT
TP53	KCNK3	TP53
KRAS	SFTPB	KRAS
BRAF	CASP8	BRAF
TSC2	BMPR2	TSC2
ABCA3	CPS1	ABCA3
NKX2-1	FARSB	NKX2-1
ERBB2	DLEC1	ERBB2
CDKN2A	CCR2	CDKN2A
SFTPC	DBR1	SFTPC
ALK	TERC	ALK
DICER1	PIK3CA	DICER1
RB1	EIF4G1	RB1
TGFB1	ATP13A3	TGFB1
IL6	SLC34A2	IL6
PIK3CA	IGFBP7	PIK3CA
MALAT1	NAF1	MALAT1
TNF	TERT	TNF
PTEN	LUCAT1	PTEN
TSC1	IRF1	TSC1
AKT1	PRKN	AKT1
COPA	EGFR	COPA
IL10	CAV1	IL10
MARS1	POT1	MARS1
SFTPB	BRAF	SFTPB
H19	SFTPC	H19
MYC	SOX17	MYC
SFTPA1	MAP3K8	SFTPA1
ITGA3	ERCC6	ITGA3
MET	SFTPA2	MET
FAS	SFTPA1	FAS
MIR21	SFTPD	MIR21
CTNNB1	MUC5B	CTNNB1
IFNG	SLC67A1	IFNG
STK11	FAM111B	STK11
STAT3	MEN1	STAT3
FASLG	MALAT1	FASLG
FGFR1	PPP2R1B	FGFR1
CCND1	KRAS	CCND1

ATM	MARS1	ATM
MUC5B	OAS1	MUC5B
CASP8	ZCCHC8	CASP8
HRAS	SMAD9	HRAS
PVT1	RB1	PVT1
NFE2L2	NSMCE3	NFE2L2
ERCC6	NOP10	ERCC6
CFTR	EIF2AK4	CFTR
FGFR3	CHRNA3	FGFR3
CSF3	ABCA3	CSF3
KIT	PARN	KIT
NOTCH1	FOXF1	NOTCH1
CXCL8	RPA1	CXCL8
MAP2K1	ERBB2	MAP2K1
SMAD4	ITGA3	SMAD4
ABCB1	MPO	ABCB1
FGFR2	TBX4	FGFR2
PRKN	FARSA	PRKN
ROS1	CAPNS1	ROS1
SERPINA1	SARS2	SERPINA1
LOC110806263	CYP2A6	LOC110806263
RET	TGFB1	RET
SFTPA2	HCK	SFTPA2
GAS5	RTEL1	GAS5
MIR125A	HMOX1	MIR125A
BRCA2	CSF2RB	BRCA2
PDGFRA	CSF2RA	PDGFRA
NF1		NF1
MIR155		MIR155
MLH1		MLH1
IL1B		IL1B
HMOX1		HMOX1
VEGFA		VEGFA
FGF10		FGF10
PPARG		PPARG
MDM2		MDM2
NEAT1		NEAT1
NRAS		NRAS
PIK3CG		PIK3CG
RARB		RARB
SFTPD		SFTPD
ACTA2		ACTA2
LUCAT1		LUCAT1

RAF1
CD274
SMARCA4
CHEK2
MIR17
RTEL1
MIR126
CAV1
BRCA1
RASSF1
BCL2
DDR2
KEAP1
KMT2D
MIR146A
LINC02605
HLA-DRB1
MIR145
APC
MIR30A
ESR1
CTLA4
NRG1
IL4
TUG1
MIRLET7C
SFTA3
IL2RA
IGF1R
IL2
MBL2
MIR140
MUC1
IRF1
TLR4
FOXF1
DLEC1
TERC
ERBB4
HLA-B
ELN
GSTM1
CYP2A6

RAF1
CD274
SMARCA4
CHEK2
MIR17
RTEL1
MIR126
CAV1
BRCA1
RASSF1
BCL2
DDR2
KEAP1
KMT2D
MIR146A
LINC02605
HLA-DRB1
MIR145
APC
MIR30A
ESR1
CTLA4
NRG1
IL4
TUG1
MIRLET7C
SFTA3
IL2RA
IGF1R
IL2
MBL2
MIR140
MUC1
IRF1
TLR4
FOXF1
DLEC1
TERC
ERBB4
HLA-B
ELN
GSTM1
CYP2A6

ACE
SNHG1
MIR143
NTRK1
MIR29A
CSF2
MIR221
MIR146B
BAP1
MIR200B
MIR98
FLNA
MIRLET7B
MMP9
ERBB3
CCL2
AXL
CDKN2B-AS1
SRC
MIRLET7D
ALB
MIR205
NTRK3
FBXW7
MIR31
CYP1A1
ICAM1
NTRK2
MMP2
PTGS2
SOX2
MIR199A1
CCR2
MIR214
HGF
DSP
ATR
MIR182
LINC00511
PPP2R1B
TP73
CYP3A5
PKHD1

ACE
SNHG1
MIR143
NTRK1
MIR29A
CSF2
MIR221
MIR146B
BAP1
MIR200B
MIR98
FLNA
MIRLET7B
MMP9
ERBB3
CCL2
AXL
CDKN2B-AS1
SRC
MIRLET7D
ALB
MIR205
NTRK3
FBXW7
MIR31
CYP1A1
ICAM1
NTRK2
MMP2
PTGS2
SOX2
MIR199A1
CCR2
MIR214
HGF
DSP
ATR
MIR182
LINC00511
PPP2R1B
TP73
CYP3A5
PKHD1

SLC19A1
CXCR4
MTOR
FLCN
MIR150
MIR22
CFH
CRP
MIR93
HIF1A
CDH1
UCA1
CCAT1
MMP1
PCAT1
MIR210
MIR34A
HLA-A
MIR338
MIR20A
GSTP1
SOD2
IDH1
HOTAIRM1
ENG
MIR195
MIR141
EDN1
MIR200C
GATA3
MIR18A
CHEK1
ANXA2
MIR133B
NFKB1
MIR335
SNHG16
MTHFR
MIR130A
MIR148A
CASP3
MIR19A
ELANE

SLC19A1
CXCR4
MTOR
FLCN
MIR150
MIR22
CFH
CRP
MIR93
HIF1A
CDH1
UCA1
CCAT1
MMP1
PCAT1
MIR210
MIR34A
HLA-A
MIR338
MIR20A
GSTP1
SOD2
IDH1
HOTAIRM1
ENG
MIR195
MIR141
EDN1
MIR200C
GATA3
MIR18A
CHEK1
ANXA2
MIR133B
NFKB1
MIR335
SNHG16
MTHFR
MIR130A
MIR148A
CASP3
MIR19A
ELANE

MIR183
SCGB1A1
MIR216A
MUC16
KDR
IDH2
MIR99A
CDKN1A
SMAD3
IL13
GNAS
BDNF-AS
FOXO1
MIR200A
PALB2
OIP5-AS1
TGFBR2
MIR101-1
MIR34C
DANCR
MIR139
EGF
XIST
ADAMTS9-AS2
MPO
TMPO-AS1
FGF7
MIR451A
MIR26A1
BMPR2
TP63
IGF1
BAX
TBX4
FGF2
ERCC2
FENDRR
NSD2
MIR30E
NORAD
POLD1
HOTAIR
CDKN2B

MIR183
SCGB1A1
MIR216A
MUC16
KDR
IDH2
MIR99A
CDKN1A
SMAD3
IL13
GNAS
BDNF-AS
FOXO1
MIR200A
PALB2
OIP5-AS1
TGFBR2
MIR101-1
MIR34C
DANCR
MIR139
EGF
XIST
ADAMTS9-AS2
MPO
TMPO-AS1
FGF7
MIR451A
MIR26A1
BMPR2
TP63
IGF1
BAX
TBX4
FGF2
ERCC2
FENDRR
NSD2
MIR30E
NORAD
POLD1
HOTAIR
CDKN2B

IL1A
GFOD3P
MIR191
PARN
MIR324
SPP1
MIR29C
LINC01089
WT1
ERCC1
SMPD1
MIR486-1
FOXP3
MIR204
MAPK1
CCN2
IL17A
CYP3A4
POLE
MIR124-1
MIR212
U2AF1
PDCD1
FN1
MCM3AP-AS1
CASC11
MIR192
SERPINE1
FARSB
FAM13A
NKILA
MIR4435-2HG
MIRLET7G
AFAP1-AS1
SHH
HLA-G
MIR106A
ENO2
MIF
MIR203A
MIR497
IL1RN
MIR224

IL1A
GFOD3P
MIR191
PARN
MIR324
SPP1
MIR29C
LINC01089
WT1
ERCC1
SMPD1
MIR486-1
FOXP3
MIR204
MAPK1
CCN2
IL17A
CYP3A4
POLE
MIR124-1
MIR212
U2AF1
PDCD1
FN1
MCM3AP-AS1
CASC11
MIR192
SERPINE1
FARSB
FAM13A
NKILA
MIR4435-2HG
MIRLET7G
AFAP1-AS1
SHH
HLA-G
MIR106A
ENO2
MIF
MIR203A
MIR497
IL1RN
MIR224

GSTT1
GATA2
CD44
CDK4
CDKN1B
SLC34A2
PGR-AS1
NNT-AS1
FBXL19-AS1
MNX1-AS1
MXRA5
THBD
RMRP
FAM83A-AS1
XRCC1
HLA-DQB1
STAT1
AKT3
MIR197
IL7R
LINC00520
GSTM3
CCL5
MIR27B
MIR33A
TIMP1
HLA-C
F2
CD40LG
MIR425
DYNC2H1
MAP3K8
MIR32
RUNX1T1
LINC00673
FLT1
AGAP2-AS1
TTR
NOD2
EPHX1
KITLG
RTEL1-TNFRSF6B
CASC9

GSTT1
GATA2
CD44
CDK4
CDKN1B
SLC34A2
PGR-AS1
NNT-AS1
FBXL19-AS1
MNX1-AS1
MXRA5
THBD
RMRP
FAM83A-AS1
XRCC1
HLA-DQB1
STAT1
AKT3
MIR197
IL7R
LINC00520
GSTM3
CCL5
MIR27B
MIR33A
TIMP1
HLA-C
F2
CD40LG
MIR425
DYNC2H1
MAP3K8
MIR32
RUNX1T1
LINC00673
FLT1
AGAP2-AS1
TTR
NOD2
EPHX1
KITLG
RTEL1-TNFRSF6B
CASC9

JUN
MIR199B
BMP4
ENO1
MIR377
MIRLET7E
IL18
CCAT2
DKC1
MIR339
HULC
BMP6
AGTR1
TTN-AS1
CERNA3
KRT19
EPO
VHL
LIG4
IL3
PIK3R1
MIR219A1
LINC00205
DGCR5
NOS2
NLRP3
ABCC1
MGMT
MIRLET7A2
FOXD3-AS1
TINF2
MIR24-2
GRP
HAGLROS
TNFRSF1A
MIR181C
MIR128-2
LINC00662
BIRC5
INS
BMP2
LINC00857
SNHG4

JUN
MIR199B
BMP4
ENO1
MIR377
MIRLET7E
IL18
CCAT2
DKC1
MIR339
HULC
BMP6
AGTR1
TTN-AS1
CERNA3
KRT19
EPO
VHL
LIG4
IL3
PIK3R1
MIR219A1
LINC00205
DGCR5
NOS2
NLRP3
ABCC1
MGMT
MIRLET7A2
FOXD3-AS1
TINF2
MIR24-2
GRP
HAGLROS
TNFRSF1A
MIR181C
MIR128-2
LINC00662
BIRC5
INS
BMP2
LINC00857
SNHG4

LINC00261	LINC00261
CBS	CBS
JAK3	JAK3
ACVRL1	ACVRL1
MIR511	MIR511
EZH2	EZH2
CD4	CD4
VDR	VDR
XIAP	XIAP
IFNA1	IFNA1
MYCN	MYCN
SNHG11	SNHG11
OGFRP1	OGFRP1
SOX9	SOX9
FHIT	FHIT
SMO	SMO
MIR125B2	MIR125B2
MAPK8	MAPK8
MIR222	MIR222
CASC15	CASC15
ADA	ADA
AGT	AGT
MSH2	MSH2
HLA-DPB1	HLA-DPB1
CFI	CFI
GATA6-AS1	GATA6-AS1
CXCL12	CXCL12
COL4A3	COL4A3
TLR2	TLR2
S100B	S100B
KRT7	KRT7
THADA	THADA
MIRLET7F1	MIRLET7F1
SBF2-AS1	SBF2-AS1
SOX21-AS1	SOX21-AS1
LCIIAR	LCIIAR
MIR15A	MIR15A
GNAQ	GNAQ
IL5	IL5
SLC7A7	SLC7A7
MEG3	MEG3
CST3	CST3
ZFPM2-AS1	ZFPM2-AS1

ITGB1
TYMS
EPIC1
ARID1A
SOD1
CD40
BCL2L1
ABCC2
EPCAM
CCR5
NBN
MIRLET7A3
SNHG20
LINC00336
PDGFRB
IL1R1
NFKBIA
HMGB1
PRNCR1
COL4A1
VIM
MIR31HG
MYCL
DLEU2
PTK2
JAK2
LMNA
HOXB-AS3
MIR345
CXCR3
CEACAM3
MUC5AC
MIR29B2
MVP
CD8A
HFE
SFTA1P
MIR429
VEGFC
PTCH1
DPP9
MIR124-3
MIR218-2

ITGB1
TYMS
EPIC1
ARID1A
SOD1
CD40
BCL2L1
ABCC2
EPCAM
CCR5
NBN
MIRLET7A3
SNHG20
LINC00336
PDGFRB
IL1R1
NFKBIA
HMGB1
PRNCR1
COL4A1
VIM
MIR31HG
MYCL
DLEU2
PTK2
JAK2
LMNA
HOXB-AS3
MIR345
CXCR3
CEACAM3
MUC5AC
MIR29B2
MVP
CD8A
HFE
SFTA1P
MIR429
VEGFC
PTCH1
DPP9
MIR124-3
MIR218-2

F3
FER1L4
VCAM1
MIR223
NOTCH3
SMAD2
PECAM1
MIR95
PCNA
PLAU
LINC00665
TGFBR1
EIF2AK4
KCNQ1OT1
IL2RB
GATA6
TDRG1
NQO1
PSMA3-AS1
BIRC3
MIR96
CXCL10
GZMB
PARP1
LINC00839
GNA11
PTPRC
IL15
PPP2R1A
MIR185
TGFA
CALCA
PLG
IL7
TGFB2
NBAT1
NCAPG
PTPRG
TNFSF10
PRF1
TTN
LINC01194
B2M
F3
FER1L4
VCAM1
MIR223
NOTCH3
SMAD2
PECAM1
MIR95
PCNA
PLAU
LINC00665
TGFBR1
EIF2AK4
KCNQ1OT1
IL2RB
GATA6
TDRG1
NQO1
PSMA3-AS1
BIRC3
MIR96
CXCL10
GZMB
PARP1
LINC00839
GNA11
PTPRC
IL15
PPP2R1A
MIR185
TGFA
CALCA
PLG
IL7
TGFB2
NBAT1
NCAPG
PTPRG
TNFSF10
PRF1
TTN
LINC01194
B2M

PIK3CD
CASC2
CCL11
MIR30D
MMP7
MAPK14
MKI67
NOS3
AKT2
ZFAS1
CEACAM5
CHGA
FLT3
MIR142
HPS1
CASP9
PDGFB
STN1
PKD1
TRG-AS1
ATP11A
CSF2RA
MIR15B
CYTOR
CERS6-AS1
ADAM12
SOX4
MIAT
CCL3
ZNF674-AS1
SNHG3
CREBBP
LAMC2
SNHG17
CDK6
ABCG2
ZMYND10
TUSC7
SLC11A1
CADM1
PTPN11
HOXA11-AS
CD36

PIK3CD
CASC2
CCL11
MIR30D
MMP7
MAPK14
MKI67
NOS3
AKT2
ZFAS1
CEACAM5
CHGA
FLT3
MIR142
HPS1
CASP9
PDGFB
STN1
PKD1
TRG-AS1
ATP11A
CSF2RA
MIR15B
CYTOR
CERS6-AS1
ADAM12
SOX4
MIAT
CCL3
ZNF674-AS1
SNHG3
CREBBP
LAMC2
SNHG17
CDK6
ABCG2
ZMYND10
TUSC7
SLC11A1
CADM1
PTPN11
HOXA11-AS
CD36

FCGR2A
IGFBP3
VWF
EDNRA
HIF1A-AS2
CBL
OGG1
NPPA
IGF2
HOTTIP
MIR198
NPM1
HAGLR
ARAP1-AS1
NOTCH2
MT1JP
MAPK3
RUNX1-IT1
BCYRN1
NSMCE3
HPS4
WFDC21P
TLR9
HLA-DQA1
HCP5
AGER
SCGB3A2
TLR3
FEZF1-AS1
SNHG12
GRM8
CYP2E1
FLT4
ACTC1
CSF2RB
LINC00467
SCNN1A
TFRC
ADIPOQ
CEACAM6
XPC
KRT18
HCG11

FCGR2A
IGFBP3
VWF
EDNRA
HIF1A-AS2
CBL
OGG1
NPPA
IGF2
HOTTIP
MIR198
NPM1
HAGLR
ARAP1-AS1
NOTCH2
MT1JP
MAPK3
RUNX1-IT1
BCYRN1
NSMCE3
HPS4
WFDC21P
TLR9
HLA-DQA1
HCP5
AGER
SCGB3A2
TLR3
FEZF1-AS1
SNHG12
GRM8
CYP2E1
FLT4
ACTC1
CSF2RB
LINC00467
SCNN1A
TFRC
ADIPOQ
CEACAM6
XPC
KRT18
HCG11

INPP5E	INPP5E
DMTF1	DMTF1
TOP1	TOP1
NRAD1	NRAD1
LEP	LEP
NCAM1	NCAM1
SP1	SP1
MIR499A	MIR499A
SOD2-OT1	SOD2-OT1
MMP14	MMP14
NPPB	NPPB
TNFRSF1B	TNFRSF1B
CDR1-AS	CDR1-AS
REN	REN
RELA	RELA
COL4A5	COL4A5
MIR193A	MIR193A
MIR100	MIR100
ELMOD2	ELMOD2
CSF1	CSF1
SLC17A5	SLC17A5
WT1-AS	WT1-AS
SNHG7	SNHG7
THBS1	THBS1
COL4A2	COL4A2
GJA1	GJA1
LINC01116	LINC01116
MMP3	MMP3
AHR	AHR
TIMP2	TIMP2
MIR34B	MIR34B
CD226	CD226
SYP	SYP
HSP90AA1	HSP90AA1
MPL	MPL
SETD2	SETD2
PRTN3	PRTN3
GPT	GPT
DNMT3A	DNMT3A
SOX2-OT	SOX2-OT
LINC00485	LINC00485
COL3A1	COL3A1
FOXM1	FOXM1

SNAI1
RHOA
IL11
CDK2
DES
SIRT1
GSN
LOXL1-AS1
DNAH5
MEN1
IL6R
DOCK8
DNMT1
EP300
XRCC3
CPS1
SMAD7
CYP1B1
PANDAR
BPIFA1
MSH6
KLF2
SLC2A1
E2F1
SERPINC1
METTL3
CD28
APOA1
GLA
LCN2
LOX
CDKN1C
CCR7
PDPN
ANGPT1
MCL1
NAPSA
IKBKG
INVS
AGO2
CHUK
MIR144
GSK3B

SNAI1
RHOA
IL11
CDK2
DES
SIRT1
GSN
LOXL1-AS1
DNAH5
MEN1
IL6R
DOCK8
DNMT1
EP300
XRCC3
CPS1
SMAD7
CYP1B1
PANDAR
BPIFA1
MSH6
KLF2
SLC2A1
E2F1
SERPINC1
METTL3
CD28
APOA1
GLA
LCN2
LOX
CDKN1C
CCR7
PDPN
ANGPT1
MCL1
NAPSA
IKBKG
INVS
AGO2
CHUK
MIR144
GSK3B

CYBB
CDK12
CD34
ABL1
HNF1A-AS1
LINC01123
MIR27A
ALOX5
COL1A1
APEX1
TOP2A
MMP12
DNMT3B
IKBKB
CLEC7A
SST
CD79A
FOXJ1
DMBT1
FANCA
MYLK
IGFBP2
CASP10
TF
RAG1
HBB
RBM10
LAMA5
GLI1
TNFRSF10B
FARSA
RAC1
TKT
FADD
GPC3
ARG1
LTA
CD46
MIR375
TP53TG1
SCNN1B
NUTM2A-AS1
THPO

CYBB
CDK12
CD34
ABL1
HNF1A-AS1
LINC01123
MIR27A
ALOX5
COL1A1
APEX1
TOP2A
MMP12
DNMT3B
IKBKB
CLEC7A
SST
CD79A
FOXJ1
DMBT1
FANCA
MYLK
IGFBP2
CASP10
TF
RAG1
HBB
RBM10
LAMA5
GLI1
TNFRSF10B
FARSA
RAC1
TKT
FADD
GPC3
ARG1
LTA
CD46
MIR375
TP53TG1
SCNN1B
NUTM2A-AS1
THPO

PLAUR	PLAUR
KRT5	KRT5
ESR2	ESR2
NME1	NME1
CYCS	CYCS
F5	F5
SOD3	SOD3
ANGPT2	ANGPT2
SPARC	SPARC
MIR23A	MIR23A
ASCL1	ASCL1
IREB2	IREB2
HSPB1	HSPB1
ADRB2	ADRB2
GBA1	GBA1
SLPI	SLPI
RXRA	RXRA
JAG1	JAG1
FOXA2	FOXA2
ACTB	ACTB
TYMP	TYMP
ALDOA	ALDOA
TINCR	TINCR
AGTR2	AGTR2
MYH7	MYH7
WNT4	WNT4
KRT20	KRT20
PRKDC	PRKDC
NAT2	NAT2
ERCC5	ERCC5
CXCL2	CXCL2
LAMB3	LAMB3
FGFR4	FGFR4
ABCB11	ABCB11
BCL6	BCL6
LOC110806306	LOC110806306
SNHG14	SNHG14
MIR372	MIR372
LAMA3	LAMA3
IL2RG	IL2RG
FBN1	FBN1
CAT	CAT
CCDC40	CCDC40

COL4A4	COL4A4
ERCC4	ERCC4
SOCS1	SOCS1
FOS	FOS
GC	GC
RARA	RARA
LINC00473	LINC00473
KRT8	KRT8
LTBP4	LTBP4
PDGFA	PDGFA
ACE2	ACE2
OFD1	OFD1
ITGAM	ITGAM
ITGB3	ITGB3
POT1	POT1
MIR30B	MIR30B
STING1	STING1
CREB1	CREB1
HSPA4	HSPA4
SNAI2	SNAI2
NAGLU	NAGLU
MUSK	MUSK
MUTYH	MUTYH
CD14	CD14
PAX2	PAX2
PTHLH	PTHLH
DUXAP8	DUXAP8
TUBB3	TUBB3
PLAT	PLAT
NRP1	NRP1
FBLN5	FBLN5
IL4R	IL4R
TNFRSF11B	TNFRSF11B
BAK1	BAK1
ACTA2-AS1	ACTA2-AS1
RPGR	RPGR
RRM1	RRM1
CEBPA	CEBPA
HAVCR2	HAVCR2
SOS1	SOS1
NCK1-DT	NCK1-DT
POSTN	POSTN
MILIP	MILIP

DPYD
CD81
MAGI2-AS3
LINC-PINT
UGT1A1
CCL4
TGFB3
LINC00958
CX3CR1
HLA-DPA1
GPRC5A
DLEU1
CTSB
LPL
MYD88
MIR137
LINC00472
LIPA
PMS2
SNHG6
CYP1A2
TPX2
ITGA2
IL12B
MIR107
TBX5
FCGR3A
CHI3L1
NPHS2
GUSB
PRKCA
EPAS1
IFNGR1
XDH
APOE
CRNDE
MICA
FTX
C3
RUNX1
CYP2D6
ODAD1
STAT6

DPYD
CD81
MAGI2-AS3
LINC-PINT
UGT1A1
CCL4
TGFB3
LINC00958
CX3CR1
HLA-DPA1
GPRC5A
DLEU1
CTSB
LPL
MYD88
MIR137
LINC00472
LIPA
PMS2
SNHG6
CYP1A2
TPX2
ITGA2
IL12B
MIR107
TBX5
FCGR3A
CHI3L1
NPHS2
GUSB
PRKCA
EPAS1
IFNGR1
XDH
APOE
CRNDE
MICA
FTX
C3
RUNX1
CYP2D6
ODAD1
STAT6

BMP7
TNFRSF8
YAP1
SMAD9
LGALS3
NCF2
ERCC3
AREG
CYP2C19
HDAC1
DNAH8
NEU1
ZEB1
HAND2-AS1
RAD51
MIR122
PTK2B
TXN
ACACA
LINC00342
HMGA2
BPTF
ANXA5
CYBA
DUSP1
JUP
LIG1
RPS6KB1
KIR3DL1
NR3C1
CDH2
CDKN3
TIMP3
THY1
SELP
LINC01234
DAPK1
ITGAV
KLF6
IDO1
MAP2K2
SPAG1
WRN

BMP7
TNFRSF8
YAP1
SMAD9
LGALS3
NCF2
ERCC3
AREG
CYP2C19
HDAC1
DNAH8
NEU1
ZEB1
HAND2-AS1
RAD51
MIR122
PTK2B
TXN
ACACA
LINC00342
HMGA2
BPTF
ANXA5
CYBA
DUSP1
JUP
LIG1
RPS6KB1
KIR3DL1
NR3C1
CDH2
CDKN3
TIMP3
THY1
SELP
LINC01234
DAPK1
ITGAV
KLF6
IDO1
MAP2K2
SPAG1
WRN

HOPX
CDC42
AURKA
IDUA
WAS
ABCC3
DNAH11
CXCR2
CD27
PTX3
PIK3CB
BSG
TEK
PSMG3-AS1
BAG3
APOL1
HNF1B
PLK1
PAX5
BANCR
WNT5A
VEGFD
DLX6-AS1
EPHB4
NAF1
PRKCI
CCNE1
BID
GLI2
HDAC9
SNHG5
MIR148B
CXCL9
CDK1
GLI3
LINC00968
PITPNA-AS1
BCR
TET2
MUC4
MIR155HG
MIR132
ATRX

HOPX
CDC42
AURKA
IDUA
WAS
ABCC3
DNAH11
CXCR2
CD27
PTX3
PIK3CB
BSG
TEK
PSMG3-AS1
BAG3
APOL1
HNF1B
PLK1
PAX5
BANCR
WNT5A
VEGFD
DLX6-AS1
EPHB4
NAF1
PRKCI
CCNE1
BID
GLI2
HDAC9
SNHG5
MIR148B
CXCL9
CDK1
GLI3
LINC00968
PITPNA-AS1
BCR
TET2
MUC4
MIR155HG
MIR132
ATRX

CCDC39	CCDC39
GAPDH	GAPDH
DNAAF3	DNAAF3
FGA	FGA
DNAAF1	DNAAF1
DDR1	DDR1
DHFR	DHFR
SNHG15	SNHG15
DNAAF4	DNAAF4
GGT1	GGT1
MIR211	MIR211
CFLAR	CFLAR
LAMA2	LAMA2
TRAF6	TRAF6
CCL17	CCL17
CCNO	CCNO
FGD5-AS1	FGD5-AS1
CD80	CD80
MSLN	MSLN
MAX	MAX
TCF7L2	TCF7L2
COMT	COMT
MIR181A1	MIR181A1
GDF15	GDF15
DLK1	DLK1
EPB41L3	EPB41L3
ENTREP2	ENTREP2
MIR106B	MIR106B
GREM1	GREM1
WNT7B	WNT7B
NODAL	NODAL
ABCC6	ABCC6
FOXO3	FOXO3
CTSD	CTSD
MIR7-3HG	MIR7-3HG
GATA4	GATA4
COL1A2	COL1A2
BIRC7	BIRC7
MIR99AHG	MIR99AHG
CDH13	CDH13
BBOX1-AS1	BBOX1-AS1
HNRNPA2B1	HNRNPA2B1
SELL	SELL

PGR
DNAI1
HMGCR
BLM
RBM6
MIR16-1
MIR25
CD24
PKM
MMP13
BUB1B
NPC2
ACTN4
NBR2
TAP1
TRAF3
RYR1
ILK
ANPEP
MBD4
IL1RL1
CCR3
SELE
CD55
CCNA2
ADAM17
KLRK1
DHCR7
MIR215
CCND3
SLC26A4
STXBP2
MIR9-2HG
IL12RB1
PART1
IL33
ALDH2
PON1
F8
ABCC4
DDB2
AXIN2
PCBP1-AS1
PGR
DNAI1
HMGCR
BLM
RBM6
MIR16-1
MIR25
CD24
PKM
MMP13
BUB1B
NPC2
ACTN4
NBR2
TAP1
TRAF3
RYR1
ILK
ANPEP
MBD4
IL1RL1
CCR3
SELE
CD55
CCNA2
ADAM17
KLRK1
DHCR7
MIR215
CCND3
SLC26A4
STXBP2
MIR9-2HG
IL12RB1
PART1
IL33
ALDH2
PON1
F8
ABCC4
DDB2
AXIN2
PCBP1-AS1

ITGB2	ITGB2
MTR	MTR
LINC00668	LINC00668
CD163	CD163
RAB27A	RAB27A
AGXT	AGXT
BRIP1	BRIP1
SOCS3	SOCS3
RSPH1	RSPH1
CDH5	CDH5
ICOS	ICOS
MIR10B	MIR10B
EGR1	EGR1
CHAT	CHAT
CCNB1	CCNB1
PCAT6	PCAT6
TRPC6	TRPC6
UNC13D	UNC13D
S100A4	S100A4
ADM	ADM
MIR152	MIR152
MIR423	MIR423
DNAAF5	DNAAF5
NPHS1	NPHS1
IL16	IL16
RSPH9	RSPH9
TWIST1	TWIST1
ANXA1	ANXA1
HSPA5	HSPA5
MIR127	MIR127
CALR	CALR
ODAD2	ODAD2
MIR342	MIR342
IL22	IL22
KCNK3	KCNK3
ITCH	ITCH
TCF21	TCF21
HSPA8	HSPA8
HPGD	HPGD
CDKN2C	CDKN2C
TNNT2	TNNT2
MMP8	MMP8
HLA-E	HLA-E

HOXA-AS2
UFC1
MST1R
CASP1
BCL10
DKK1
DPP4
CYP24A1
ID1
CTC1
LGALS1
CEBPB
PPP1R13L
CABIN1
LBR
CCR1
RACK1
MIR125B1
TLR7
HSPA1A
CYP17A1
MIR373
SF3B2
BMI1
PCAT19
LEF1-AS1
PDPK1
NSD1
CP
MANCR
PROM1
ALDH1A1
CASR
ATP7B
MIR374A
HYDIN
ITGA5
KTN1-AS1
TNNI3
DDX11-AS1
FAS-AS1
IRS1
BGLAP
HOXA-AS2
UFC1
MST1R
CASP1
BCL10
DKK1
DPP4
CYP24A1
ID1
CTC1
LGALS1
CEBPB
PPP1R13L
CABIN1
LBR
CCR1
RACK1
MIR125B1
TLR7
HSPA1A
CYP17A1
MIR373
SF3B2
BMI1
PCAT19
LEF1-AS1
PDPK1
NSD1
CP
MANCR
PROM1
ALDH1A1
CASR
ATP7B
MIR374A
HYDIN
ITGA5
KTN1-AS1
TNNI3
DDX11-AS1
FAS-AS1
IRS1
BGLAP

NR2F1-AS1
HLA-DQB1-AS1
MIR151A
FGF1
SLC25A13
WRAP53
BTK
MIR10A
BIRC2
CXCR1
POLG
MIR23B
ETS1
FOXA1
RUNX3
IFI27
IGFBP6
RAD50
CC2D2A
ZEB1-AS1
CD82
HIPK2
TUBB
NPHP3
MIR9-1
MYH9
CYP19A1
JAK1
LINC00460
ELF3-AS1
MALT1
GLB1
G6PD
PRKG1
ZIC3
MIR186
SH2D1A
MIR424
CD9
MEG8
CSF1R
AQP5
ITGAL

NR2F1-AS1
HLA-DQB1-AS1
MIR151A
FGF1
SLC25A13
WRAP53
BTK
MIR10A
BIRC2
CXCR1
POLG
MIR23B
ETS1
FOXA1
RUNX3
IFI27
IGFBP6
RAD50
CC2D2A
ZEB1-AS1
CD82
HIPK2
TUBB
NPHP3
MIR9-1
MYH9
CYP19A1
JAK1
LINC00460
ELF3-AS1
MALT1
GLB1
G6PD
PRKG1
ZIC3
MIR186
SH2D1A
MIR424
CD9
MEG8
CSF1R
AQP5
ITGAL

ITGA6
LIF
GSTA1
BCL2L11
POU5F1
GRPR
SHC1
CCNH
RMST
WDR19
YBX1
TNFRSF10A
GADD45A
DHRS4-AS1
BLACAT1
FGF9
CCK
EFEMP2
ROBO1
ASAH1
AIFM1
BICDL3P
RSPH4A
ZNF281
IL9
SMARCB1
ELAVL1
SMARCA5
SNHG8
ARHGAP45
AR
HPRT1
SERPINA3
CXCL1
DSCAM-AS1
XRCC5
MYH11
GMDS-DT
ALPL
TCF4
IGFBP5
XRCC2
CCBE1

ITGA6
LIF
GSTA1
BCL2L11
POU5F1
GRPR
SHC1
CCNH
RMST
WDR19
YBX1
TNFRSF10A
GADD45A
DHRS4-AS1
BLACAT1
FGF9
CCK
EFEMP2
ROBO1
ASAH1
AIFM1
BICDL3P
RSPH4A
ZNF281
IL9
SMARCB1
ELAVL1
SMARCA5
SNHG8
ARHGAP45
AR
HPRT1
SERPINA3
CXCL1
DSCAM-AS1
XRCC5
MYH11
GMDS-DT
ALPL
TCF4
IGFBP5
XRCC2
CCBE1

MTHFD1	MTHFD1
GFUS	GFUS
EFEMP1	EFEMP1
VIP	VIP
ZEB2-AS1	ZEB2-AS1
MIR149	MIR149
NEK8	NEK8
NES	NES
RXRB	RXRB
EPHX2	EPHX2
NF2	NF2
HP	HP
TMPRSS2	TMPRSS2
PPARA	PPARA
CELF1	CELF1
MKS1	MKS1
AGR2	AGR2
SIX1	SIX1
ATP8B1	ATP8B1
ARNT	ARNT
FLNC	FLNC
RNASE3	RNASE3
LINC01503	LINC01503
IGF2R	IGF2R
GAA	GAA
IL6ST	IL6ST
ODC1	ODC1
MTAP	MTAP
XRCC6	XRCC6
ASXL1	ASXL1
MINCR	MINCR
CTCF	CTCF
GDF1	GDF1
GAST	GAST
CTNND1	CTNND1
TRAF2	TRAF2
CIITA	CIITA
MTHFD2	MTHFD2
SOX17	SOX17
GHRL	GHRL
CLDN7	CLDN7
SOX13	SOX13
MELTF-AS1	MELTF-AS1

EPHA2	EPHA2
LDLR	LDLR
SERPINH1	SERPINH1
SKP2	SKP2
MIR26B	MIR26B
IFIH1	IFIH1
MIRLET7A1	MIRLET7A1
EZR	EZR
AFP	AFP
DLGAP1-AS1	DLGAP1-AS1
DCK	DCK
IFT140	IFT140
KMT2A	KMT2A
TATDN1	TATDN1
TYK2	TYK2
CHKA	CHKA
DARS1-AS1	DARS1-AS1
COL18A1	COL18A1
MIR503HG	MIR503HG
KCNN4	KCNN4
CSNK2A3	CSNK2A3
MIR29B1	MIR29B1
MASP2	MASP2
LYST	LYST
RAG2	RAG2
MTA1	MTA1
SCARNA5	SCARNA5
NPSR1-AS1	NPSR1-AS1
COL5A1	COL5A1
MSH3	MSH3
RASA1	RASA1
BMPR1B	BMPR1B
PDCD1LG2	PDCD1LG2
MME	MME
MIR378A	MIR378A
SEMA3B	SEMA3B
CFTR-AS1	CFTR-AS1
LINC01512	LINC01512
TNC	TNC
BARD1	BARD1
ITGA9	ITGA9
RPSA	RPSA
IGFBP7	IGFBP7

PINK1-AS
MIR193B
OSER1-DT
RUNX2
LINC00324
SLC16A1-AS1
CLU
KAT7
CXCL5
LINC01572
LAMB1
TCF3
FASN
PRKACA
GANAB
YY1
DNAL1
PRDX6
MIR379
PTPRM
NCOA3
RCVRN
GFAP
CTSL
TSPOAP1-AS1
MIR196A1
ADAMTS13
YWHAE
PDK1
PKD2
PHGDH
CD86
RHOB
MAN2B1
CCL18
SKP1
TAP2
LRBA
CD3E
TGIF1
TNFSF11
EXO1
ACHE
PINK1-AS
MIR193B
OSER1-DT
RUNX2
LINC00324
SLC16A1-AS1
CLU
KAT7
CXCL5
LINC01572
LAMB1
TCF3
FASN
PRKACA
GANAB
YY1
DNAL1
PRDX6
MIR379
PTPRM
NCOA3
RCVRN
GFAP
CTSL
TSPOAP1-AS1
MIR196A1
ADAMTS13
YWHAE
PDK1
PKD2
PHGDH
CD86
RHOB
MAN2B1
CCL18
SKP1
TAP2
LRBA
CD3E
TGIF1
TNFSF11
EXO1
ACHE

HMHB1
TPM1
BCAR4
NT5E
BAD
DMD
FANCC
ARSB
EPB41
TPMT
DRC1
DNAI2
MIR181B1
CPA4
CCDC144NL-AS1
DIABLO
SAT1
HIPK3
TFAP2A
SNHG10
ASS1
HBEGF
F2RL3
FCGR3B
GAPLINC
DUXAP9
CLDN1
TRIP13
C4B
POU3F3
E2F2
BDNF
MYBPC3
NPC1
SYK
AURKB
UPF1
RNF32-DT
WNT7A
DCTN4
SPRY2
PNP
FOXP2

HMHB1
TPM1
BCAR4
NT5E
BAD
DMD
FANCC
ARSB
EPB41
TPMT
DRC1
DNAI2
MIR181B1
CPA4
CCDC144NL-AS1
DIABLO
SAT1
HIPK3
TFAP2A
SNHG10
ASS1
HBEGF
F2RL3
FCGR3B
GAPLINC
DUXAP9
CLDN1
TRIP13
C4B
POU3F3
E2F2
BDNF
MYBPC3
NPC1
SYK
AURKB
UPF1
RNF32-DT
WNT7A
DCTN4
SPRY2
PNP
FOXP2

PRKCE
MIR574
TTC7A
NEK6
SLC7A5
PTGS1
TP53COR1
IKZF1
ITK
KIR2DL3
CYP27A1
LCK
APAF1
VPS9D1-AS1
HHIP-AS1
LIG3
MITF
THORLNC
FSCN1
DCN
PRECSIT
DRC2
CENPF
DNAAF2
TLCD3A
TRAF1
SERPINB5
SERPINF1
MEFV
MIR532
BTRC
CD59
FAH
SCNN1G
DVL1
PTPN22
AXIN1
MYB
MECP2
C4A
STAT5B
PLCG1
TTC21B

PRKCE
MIR574
TTC7A
NEK6
SLC7A5
PTGS1
TP53COR1
IKZF1
ITK
KIR2DL3
CYP27A1
LCK
APAF1
VPS9D1-AS1
HHIP-AS1
LIG3
MITF
THORLNC
FSCN1
DCN
PRECSIT
DRC2
CENPF
DNAAF2
TLCD3A
TRAF1
SERPINB5
SERPINF1
MEFV
MIR532
BTRC
CD59
FAH
SCNN1G
DVL1
PTPN22
AXIN1
MYB
MECP2
C4A
STAT5B
PLCG1
TTC21B

CDX2
PTGER4
MCM3AP
IL12A
LTBP1
TLR5
PTGIS
GALC
PTPN13
HOXC9
PSMB9
ABCA1
LINC00355
MIR130B
AOC3
IFNA2
PLAG1
LAMA4
ASMTL-AS1
STMN1
DNAAF11
IDS
LINC01833
POR
CEACAM1
HPSE
PGM3
LBX2-AS1
RBM5
HNF4A
ITGB4
TNFRSF9
EPOR
TERF1
HYAL2
DNAAF19
ETV6
EIF2AK2
HMMR-AS1
LINC00852
ST3GAL6-AS1
GBE1
TK1

CDX2
PTGER4
MCM3AP
IL12A
LTBP1
TLR5
PTGIS
GALC
PTPN13
HOXC9
PSMB9
ABCA1
LINC00355
MIR130B
AOC3
IFNA2
PLAG1
LAMA4
ASMTL-AS1
STMN1
DNAAF11
IDS
LINC01833
POR
CEACAM1
HPSE
PGM3
LBX2-AS1
RBM5
HNF4A
ITGB4
TNFRSF9
EPOR
TERF1
HYAL2
DNAAF19
ETV6
EIF2AK2
HMMR-AS1
LINC00852
ST3GAL6-AS1
GBE1
TK1

FRAS1
MIR92A1
LINC01270
WNT1
SLC9A3-AS1
PXN
UCHL1
NPHP1
IFNGR2
PLA2G4A
ITGA2B
LAMP2
FOXP1
RECK
RBP4
VTN
PSG2
MIR494
MIR582
ADA2
LTF
PIM1
KNG1
DRD2
TPTEP1
STRA6
MIR24-1
ACP5
KLF4
MIRLET7I
HDGF
PRL
SSTR2
SDHB
MIR590
RAC2
IRF4
AP3B1
RNH1
PIK3R2
PRKAR1A
HYAL1
ARAF

FRAS1
MIR92A1
LINC01270
WNT1
SLC9A3-AS1
PXN
UCHL1
NPHP1
IFNGR2
PLA2G4A
ITGA2B
LAMP2
FOXP1
RECK
RBP4
VTN
PSG2
MIR494
MIR582
ADA2
LTF
PIM1
KNG1
DRD2
TPTEP1
STRA6
MIR24-1
ACP5
KLF4
MIRLET7I
HDGF
PRL
SSTR2
SDHB
MIR590
RAC2
IRF4
AP3B1
RNH1
PIK3R2
PRKAR1A
HYAL1
ARAF

HSPD1
HSP90B1
XBP1
BMPR1A
ATP2A2
NLRC4
SDHAP1
CR1
HNRNPU
CCN1
LNCRNA-ATB
TLR1
BRD4
EPHA3
ABCB4
MIR330
S100A9
WIF1
CYP2A13
CDK5
NKX2-5
CD19
POLI
DDX5
IGFBP1
POLB
IL18R1
FGF23
LINC00491
LMCD1-AS1
LAMA1
CEP290
HAMP
RARS1
HDAC6
SLC6A14
PRKCB
CALB2
CCR6
KAT5
AQP1
MIR483
RYR2

HSPD1
HSP90B1
XBP1
BMPR1A
ATP2A2
NLRC4
SDHAP1
CR1
HNRNPU
CCN1
LNCRNA-ATB
TLR1
BRD4
EPHA3
ABCB4
MIR330
S100A9
WIF1
CYP2A13
CDK5
NKX2-5
CD19
POLI
DDX5
IGFBP1
POLB
IL18R1
FGF23
LINC00491
LMCD1-AS1
LAMA1
CEP290
HAMP
RARS1
HDAC6
SLC6A14
PRKCB
CALB2
CCR6
KAT5
AQP1
MIR483
RYR2

GAS6-DT
GRB2
LYZ
PRDX1
LINC00667
CTAG1B
NOS1
MIR196B
MIR409
DNAH1
MAGEA4
MIR361
CAVIN3
EWSR1
UGT1A7
MIR708
SEMA3F
EBLN3P
DUSP6
CTNNA1
HSP90AB1
XRCC4
CEACAM7
MVK
CD63
ETV4
AHRR
C5AR1
NR4A1
MAT2B
CCR4
ENPP2
COX4I2
HOXA5
FEN1
MIR138-1
KIR2DL4
CA9
WNT3
CCL22
TRPV4
MIR503
TRERNA1
GAS6-DT
GRB2
LYZ
PRDX1
LINC00667
CTAG1B
NOS1
MIR196B
MIR409
DNAH1
MAGEA4
MIR361
CAVIN3
EWSR1
UGT1A7
MIR708
SEMA3F
EBLN3P
DUSP6
CTNNA1
HSP90AB1
XRCC4
CEACAM7
MVK
CD63
ETV4
AHRR
C5AR1
NR4A1
MAT2B
CCR4
ENPP2
COX4I2
HOXA5
FEN1
MIR138-1
KIR2DL4
CA9
WNT3
CCL22
TRPV4
MIR503
TRERNA1

CXADR
ESM1
DDIT3
STAT5A
IL24
PPM1D
GSR
MAGEA1
NME8
NCF4
MAPKAPK5-AS1
KIR2DL2
GDNF
ST8SIA6-AS1
ARSA
SNHG9
NEK1
TYMSOS
MIR30C1
WWOX
COL2A1
MIR495
ZAP70
E2F3
MIR320A
ING1
TCIM
GPX3
LINC01138
MIR17HG
MACC1-AS1
MAP2K4
HSPG2
SYNE1
ZFPM2
IL10RA
ST7-AS1
SIRT3
CDC25A
LIFR
KRT14
TERF2
FAM83H

CXADR
ESM1
DDIT3
STAT5A
IL24
PPM1D
GSR
MAGEA1
NME8
NCF4
MAPKAPK5-AS1
KIR2DL2
GDNF
ST8SIA6-AS1
ARSA
SNHG9
NEK1
TYMSOS
MIR30C1
WWOX
COL2A1
MIR495
ZAP70
E2F3
MIR320A
ING1
TCIM
GPX3
LINC01138
MIR17HG
MACC1-AS1
MAP2K4
HSPG2
SYNE1
ZFPM2
IL10RA
ST7-AS1
SIRT3
CDC25A
LIFR
KRT14
TERF2
FAM83H

PGF
MIR328
GDF2
POMC
AIMP2
BAG1
NMBR
FLNB
HEIH
SNHG18
MCM2
LINC01559
NAT1
PPP1R13B-DT
LAMC1
TBX1
SREBF1
SMAD6
ABCA7
THBS2
LINC01139
CASP7
FGF8
MIR134
EIF4E
LZTR1
TRB
LATS2
HCK
IRF8
UBE2D2
IRAK1
SULT1A1
KDM4B
AKR1B10
TOLLIP
CXCL11
LINC00641
IFT172
AK2
EMD
BBC3
CCND2

PGF
MIR328
GDF2
POMC
AIMP2
BAG1
NMBR
FLNB
HEIH
SNHG18
MCM2
LINC01559
NAT1
PPP1R13B-DT
LAMC1
TBX1
SREBF1
SMAD6
ABCA7
THBS2
LINC01139
CASP7
FGF8
MIR134
EIF4E
LZTR1
TRB
LATS2
HCK
IRF8
UBE2D2
IRAK1
SULT1A1
KDM4B
AKR1B10
TOLLIP
CXCL11
LINC00641
IFT172
AK2
EMD
BBC3
CCND2

NEK9	NEK9
RXRG	RXRG
NCF1	NCF1
PRR11-AS1	PRR11-AS1
ARHGAP10	ARHGAP10
SLC9A3	SLC9A3
ALDH1A2	ALDH1A2
BTG2	BTG2
GADD45G	GADD45G
MIR136	MIR136
CD74	CD74
RIT1	RIT1
ABCC10	ABCC10
COMETT	COMETT
WEE1	WEE1
TGFBI	TGFBI
IMPDH2	IMPDH2
STAT4	STAT4
EIF2AK3	EIF2AK3
LACTB	LACTB
ADAR	ADAR
NMB	NMB
TNXB	TNXB
FOXN1	FOXN1
FGF18	FGF18
SDCCAG8	SDCCAG8
SUMO1P3	SUMO1P3
ITGA4	ITGA4
TNFSF13B	TNFSF13B
MIR28	MIR28
MIR210HG	MIR210HG
PI3	PI3
SERPINB1	SERPINB1
SMARCA2	SMARCA2
PSAP	PSAP
NOP10	NOP10
PGK1	PGK1
TCTN3	TCTN3
PIK3R3	PIK3R3
SNORD15A	SNORD15A
EWSAT1	EWSAT1
ASAP1-IT1	ASAP1-IT1
S100A2	S100A2

MMUT
GALNS
MIR584
DROSHA
NR1H4
PLA2G6
DKK3
GJB2
GACAT3
CD247
DIO3OS
OTC
ADGRG6
RPS27A
SPTAN1
DPYSL5
XPO5
EDNRB
CUL1
CXCR5
MAT2A
PTTG1
GADD45B
ARHGAP9
ARRB1
CKS1B
IGHE
STIL
BMP1
CDA
FOXC2-AS1
PRKAA1
CAV3
LINC01224
G6PC1
DACH1
HLA-DRB5
NCOR1
HOXB9
NHP2
ZEB2
TRAF4
CCKBR
MMUT
GALNS
MIR584
DROSHA
NR1H4
PLA2G6
DKK3
GJB2
GACAT3
CD247
DIO3OS
OTC
ADGRG6
RPS27A
SPTAN1
DPYSL5
XPO5
EDNRB
CUL1
CXCR5
MAT2A
PTTG1
GADD45B
ARHGAP9
ARRB1
CKS1B
IGHE
STIL
BMP1
CDA
FOXC2-AS1
PRKAA1
CAV3
LINC01224
G6PC1
DACH1
HLA-DRB5
NCOR1
HOXB9
NHP2
ZEB2
TRAF4
CCKBR

CSNK1A1
PPIE
HBG2
KDM1A
MAEA
CRB2
VIPR1
BUB1
ACVR2B
RECQL4
NPRL2
HK2
AIF1
NR1H2
CCN4
APOB
NFKB2
FPGS
TIMELESS
CD276
BCOR
MAPKAPK3
MCM4
MIR455
HDAC4
ALMS1
PIK3C2A
MIR615
HAR1A
LRP5
TP53BP1
MIR133A1
CDC25B
MIR218-1
OSM
PTGES
LPAR1
HDAC2
MIR135B
STK4
SOX11
CD69
STIM1

CSNK1A1
PPIE
HBG2
KDM1A
MAEA
CRB2
VIPR1
BUB1
ACVR2B
RECQL4
NPRL2
HK2
AIF1
NR1H2
CCN4
APOB
NFKB2
FPGS
TIMELESS
CD276
BCOR
MAPKAPK3
MCM4
MIR455
HDAC4
ALMS1
PIK3C2A
MIR615
HAR1A
LRP5
TP53BP1
MIR133A1
CDC25B
MIR218-1
OSM
PTGES
LPAR1
HDAC2
MIR135B
STK4
SOX11
CD69
STIM1

LINC01232
FANCD2
PTMA
TLR8
STX11
TNFRSF6B
WNT3A
MIR135A1
CHD7
IBSP
MIR154
MIR340
MED19
MRE11
RBL2
TGM2
HMGA1
C11orf65
AKAP13
PPIA
FGF20
TMEM67
RRM2
PDCD4
RPL5
RIOX2
HSD17B1
SRSF2
PDE4A
PEX1
LAMB2
PIK3C3
CHRM3
LEFTY2
CHRNA7
CTPS1
SUFU
MAGEA3
LIMD1
MTRR
PLA2G2A
EREG
CDCP1

LINC01232
FANCD2
PTMA
TLR8
STX11
TNFRSF6B
WNT3A
MIR135A1
CHD7
IBSP
MIR154
MIR340
MED19
MRE11
RBL2
TGM2
HMGA1
C11orf65
AKAP13
PPIA
FGF20
TMEM67
RRM2
PDCD4
RPL5
RIOX2
HSD17B1
SRSF2
PDE4A
PEX1
LAMB2
PIK3C3
CHRM3
LEFTY2
CHRNA7
CTPS1
SUFU
MAGEA3
LIMD1
MTRR
PLA2G2A
EREG
CDCP1

PITX2
FAM138B
SAMHD1
MB
SDC1
PRMT5
LIN28B-AS1
PPP1CB
CSF3R
TMEM216
NGF
PPARD
RAD23B
HES1
SAA1
NAMPT
SRGAP1
LPCAT1
MMP10
TRA-TGC7-1
TRAF5
SGK1
TNFAIP3
HPS6
IL10RB
PROC
MAGT1
SEC61A1
NOX4
LBP
PAX8
PML
FOLR1
ECT2
CACNA1C
PWAR1
SDHD
INSM1
PROCR
C1S
MIR296
RPGRIP1L
DCLRE1C

PITX2
FAM138B
SAMHD1
MB
SDC1
PRMT5
LIN28B-AS1
PPP1CB
CSF3R
TMEM216
NGF
PPARD
RAD23B
HES1
SAA1
NAMPT
SRGAP1
LPCAT1
MMP10
TRA-TGC7-1
TRAF5
SGK1
TNFAIP3
HPS6
IL10RB
PROC
MAGT1
SEC61A1
NOX4
LBP
PAX8
PML
FOLR1
ECT2
CACNA1C
PWAR1
SDHD
INSM1
PROCR
C1S
MIR296
RPGRIP1L
DCLRE1C

LEPR
MIR103A1
ELAVL4
FOXH1
CHIT1
CTSK
MIR545
AQP3
PCSK9
FANCF
GPX4
PSMA4
SLC2A3
H2AX
USP8
NFATC2
CACNA2D2
MIR370
LGALS3BP
CD47
TBX2
CD151
KDM6A
RALBP1
LAG3
ANLN
HADHA
SLC22A5
IL23R
MIR302A
FANCL
KL
CARD11
ZBTB17
INF2
MIR485
NR1I2
TRIM28
CXCL13
SERPINB2
IFNB1
MIR302C
CCNE2

LEPR
MIR103A1
ELAVL4
FOXH1
CHIT1
CTSK
MIR545
AQP3
PCSK9
FANCF
GPX4
PSMA4
SLC2A3
H2AX
USP8
NFATC2
CACNA2D2
MIR370
LGALS3BP
CD47
TBX2
CD151
KDM6A
RALBP1
LAG3
ANLN
HADHA
SLC22A5
IL23R
MIR302A
FANCL
KL
CARD11
ZBTB17
INF2
MIR485
NR1I2
TRIM28
CXCL13
SERPINB2
IFNB1
MIR302C
CCNE2

XPO1
MIR362
MAP3K2
CD2AP
NTS
TFPI2
ADAM15
CUBN
TLE1
PPIG
RETN
PRKCSH
SF3B1
CCL20
FREM2
TBXT
MIR128-1
SLC40A1
SMARCAL1
PLCE1
IL17F
EYA1
CD3D
MGST1
BPIFB1
FOSL1
TGM1
RAD52
F2RL1
FUS
ITGB6
TUBA1B
NPHP4
SPI1
MPG
F2R
PDP1
CRYAB
CR2
CDC25C
MIR188
ELFN1-AS1
MICB

XPO1
MIR362
MAP3K2
CD2AP
NTS
TFPI2
ADAM15
CUBN
TLE1
PPIG
RETN
PRKCSH
SF3B1
CCL20
FREM2
TBXT
MIR128-1
SLC40A1
SMARCAL1
PLCE1
IL17F
EYA1
CD3D
MGST1
BPIFB1
FOSL1
TGM1
RAD52
F2RL1
FUS
ITGB6
TUBA1B
NPHP4
SPI1
MPG
F2R
PDP1
CRYAB
CR2
CDC25C
MIR188
ELFN1-AS1
MICB

TMSB10
FANCE
WIPF1
PLCG2
HBA1
S100A8
ACY1
VCP
GRIP1
OAS1
MIR33B
MIR365A
PODXL
SDHC
PIGA
MIR625
ATG7
BAG6
HNF1A
MMACHC
PCGEM1
MIR7-1
CAV2
MIR490
SAMD9
ITGAX
S100A1
MSR1
ACTA1
MCM3
TNFRSF13B
GATA5
MIR20B
GPX1
EXT1
PNKP
MIR331
FCN3
HSF1
AKR1C3
RIPK1
IFNAR1
TNFSF12

TMSB10
FANCE
WIPF1
PLCG2
HBA1
S100A8
ACY1
VCP
GRIP1
OAS1
MIR33B
MIR365A
PODXL
SDHC
PIGA
MIR625
ATG7
BAG6
HNF1A
MMACHC
PCGEM1
MIR7-1
CAV2
MIR490
SAMD9
ITGAX
S100A1
MSR1
ACTA1
MCM3
TNFRSF13B
GATA5
MIR20B
GPX1
EXT1
PNKP
MIR331
FCN3
HSF1
AKR1C3
RIPK1
IFNAR1
TNFSF12

ALPP
TH
HLA-DRA
CUX1
VCL
WFDC2
FANCG
RSPO2
MGP
TRC-GCA24-1
COL14A1
TLR6
MMP11
SOS2
SLC37A4
MIR99B
PRKCD
TCIRG1
ASL
FUT4
UMOD
RAD51C
HBA2
CTNS
EFNB3
BMX
NTHL1
CYP7B1
CSTB
LOC111674472
UBA7
FECH
MYO1G
IRF5
MIR675
AVP
PDE5A
SMARCE1
COPS5
PSMB8
KCNQ1
CDK7
CD38

ALPP
TH
HLA-DRA
CUX1
VCL
WFDC2
FANCG
RSPO2
MGP
TRC-GCA24-1
COL14A1
TLR6
MMP11
SOS2
SLC37A4
MIR99B
PRKCD
TCIRG1
ASL
FUT4
UMOD
RAD51C
HBA2
CTNS
EFNB3
BMX
NTHL1
CYP7B1
CSTB
LOC111674472
UBA7
FECH
MYO1G
IRF5
MIR675
AVP
PDE5A
SMARCE1
COPS5
PSMB8
KCNQ1
CDK7
CD38

PADI4	PADI4
STUB1	STUB1
PPP2R2A	PPP2R2A
FGF3	FGF3
SMC1A	SMC1A
SGCD	SGCD
DOCK2	DOCK2
FYN	FYN
PEBP1	PEBP1
PXDN	PXDN
CD22	CD22
ALOX12	ALOX12
IRF3	IRF3
CHD4	CHD4
MST1	MST1
EMP2	EMP2
TBK1	TBK1
MYH6	MYH6
NNMT	NNMT
SHMT1	SHMT1
PTPRD	PTPRD
PTH	PTH
MIR501	MIR501
FHL1	FHL1
PKD1L1	PKD1L1
AGRN	AGRN
CLDN14-AS1	CLDN14-AS1
PLEC	PLEC
CBLB	CBLB
SH3GL1	SH3GL1
LATS1	LATS1
MIR382	MIR382
DDX41	DDX41
RPA1	RPA1
AHI1	AHI1
SLC6A3	SLC6A3
PRNP	PRNP
CTSG	CTSG
HSD11B2	HSD11B2
IGF2BP3	IGF2BP3
TCTN2	TCTN2
FKRP	FKRP
PDE3B	PDE3B

COL11A1
ABCC5
P2RX7
CLDN4
MIR301A
RASGRP1
IL17RA
CAP1
MIR196A2
FGL1
LCAT
SKIC2
LOC111674475
FHL2
CS
MYO5A
FANCB
TARDBP
CRABP2
MBD2
PINK1
PBX1
TNFRSF10D
GRHPR
DEFB4A
MAD2L1
NOG
VDAC1
PSMC4
LINC02893
CYSLTR1
USP7
SLIT2
TNFRSF11A
DNM2
PAX6
MKKS
SH2B3
SLC28A1
TBX20
CYP2R1
HOXC13
SETBP1

COL11A1
ABCC5
P2RX7
CLDN4
MIR301A
RASGRP1
IL17RA
CAP1
MIR196A2
FGL1
LCAT
SKIC2
LOC111674475
FHL2
CS
MYO5A
FANCB
TARDBP
CRABP2
MBD2
PINK1
PBX1
TNFRSF10D
GRHPR
DEFB4A
MAD2L1
NOG
VDAC1
PSMC4
LINC02893
CYSLTR1
USP7
SLIT2
TNFRSF11A
DNM2
PAX6
MKKS
SH2B3
SLC28A1
TBX20
CYP2R1
HOXC13
SETBP1

TIA1	TIA1
H1-0	H1-0
OCA2	OCA2
CYBC1	CYBC1
AMFR	AMFR
ECE1	ECE1
UGT2B7	UGT2B7
DLL4	DLL4
SLCO1B1	SLCO1B1
KIAA0586	KIAA0586
SDHA	SDHA
SPRED1	SPRED1
LOXL2	LOXL2
IGBP1	IGBP1
TXNRD1	TXNRD1
MIR369	MIR369
MIR491	MIR491
TG	TG
WDR35	WDR35
PLOD1	PLOD1
AGL	AGL
LNCARSR	LNCARSR
CD68	CD68
ENTPD1	ENTPD1
APP	APP
COQ8B	COQ8B
MIR19B1	MIR19B1
KIR2DL5A	KIR2DL5A
FOXK2	FOXK2
PRKD1	PRKD1
FCN2	FCN2
NFKBIB	NFKBIB
HFE-AS1	HFE-AS1
PF4	PF4
HABP2	HABP2
EDN3	EDN3
MYOD1	MYOD1
CEP83	CEP83
UROS	UROS
CORO1A	CORO1A
PTGER2	PTGER2
ELP1	ELP1
ABCG1	ABCG1

LGALS9
FAT4
CYP2B6
MECOM
WNT2
CMA1
CLEC1A
IL1RAPL2
MAP3K14
MIR190A
ITGA8
FOXF2
MIR487B
CCNA1
SPINT2
NEDD9
HHIP
UGT1A9
PIAS3
DDX3X
ADH1C
FTL
SBDS
TRIM8
FLI1
MSRA
BECN1
BBS1
DLC1
MYPN
ABCB7
NFIB
SLFN11
IL21
FURIN
FKBP5
CD52
MS4A1
RPL13A
HCG18
APOA4
GRHL2
KIF14

LGALS9
FAT4
CYP2B6
MECOM
WNT2
CMA1
CLEC1A
IL1RAPL2
MAP3K14
MIR190A
ITGA8
FOXF2
MIR487B
CCNA1
SPINT2
NEDD9
HHIP
UGT1A9
PIAS3
DDX3X
ADH1C
FTL
SBDS
TRIM8
FLI1
MSRA
BECN1
BBS1
DLC1
MYPN
ABCB7
NFIB
SLFN11
IL21
FURIN
FKBP5
CD52
MS4A1
RPL13A
HCG18
APOA4
GRHL2
KIF14

SERPINB3
FAT1
GSTM4
TRPS1
SLC29A1
TP53BP2
ROR2
RANBP9
UBA1
DLL3
APOA2
CYLD
ERG
MTDH
ABCD1
PTRH2
HOXA9
CFB
TTF1
HSD17B4
UNG
PAPPA
CITED2
CPSF6
SPRED2
PCLAF
CRELD1
TYR
CYP27B1
ABCC8
PABPN1
MIR542
COL5A2
TSLP
BPI
PHOX2B
TBXAS1
FIRRE
TJP1
FH
BMAL2
CYP4B1
MIR337

SERPINB3
FAT1
GSTM4
TRPS1
SLC29A1
TP53BP2
ROR2
RANBP9
UBA1
DLL3
APOA2
CYLD
ERG
MTDH
ABCD1
PTRH2
HOXA9
CFB
TTF1
HSD17B4
UNG
PAPPA
CITED2
CPSF6
SPRED2
PCLAF
CRELD1
TYR
CYP27B1
ABCC8
PABPN1
MIR542
COL5A2
TSLP
BPI
PHOX2B
TBXAS1
FIRRE
TJP1
FH
BMAL2
CYP4B1
MIR337

CEACAM8
RAPSN
FOXC2
LYN
CAVIN1
CLTC
KMT2C
TRE-TTC3-1
SCN5A
RPL11
MBD1
CBR1
SALL1
ALAD
DLL1
RFXANK
SQSTM1
FTH1
MIR202
RANBP2
FOXP4-AS1
LTBP3
RAD9A
ATOH1
ADAM19
MIR299
ACVR1
KLRC1
ADAM28
MIR452
PTP4A3
ADRB1
MAD1L1
LSM1
UROD
FGF4
SAMD9L
TRMU
CRKL
STK36
COL6A1
TRAP1
CLN6

CEACAM8
RAPSN
FOXC2
LYN
CAVIN1
CLTC
KMT2C
TRE-TTC3-1
SCN5A
RPL11
MBD1
CBR1
SALL1
ALAD
DLL1
RFXANK
SQSTM1
FTH1
MIR202
RANBP2
FOXP4-AS1
LTBP3
RAD9A
ATOH1
ADAM19
MIR299
ACVR1
KLRC1
ADAM28
MIR452
PTP4A3
ADRB1
MAD1L1
LSM1
UROD
FGF4
SAMD9L
TRMU
CRKL
STK36
COL6A1
TRAP1
CLN6

SEMA3E
NCOR2
RAD51B
TRA
DMAP1
UMPS
DSG2
PRKAG2
TBX21
PIP
ELAC2
MC1R
MIR885
B9D2
IGFBP4
FLT3LG
SRSF1
MIR500A
BRS3
CHD8
HAS2
ZNF350
COPS2
KRT17
CD5
ARID1B
SRF
TAC1
IL13RA1
PEX5
YWHAZ
MIR129-1
ZCCHC8
BBS2
ABCG8
LAMP1
GALK1
AVPR2
EIF4EBP1
TEP1
PRKCG
PTPN1
FBP1

SEMA3E
NCOR2
RAD51B
TRA
DMAP1
UMPS
DSG2
PRKAG2
TBX21
PIP
ELAC2
MC1R
MIR885
B9D2
IGFBP4
FLT3LG
SRSF1
MIR500A
BRS3
CHD8
HAS2
ZNF350
COPS2
KRT17
CD5
ARID1B
SRF
TAC1
IL13RA1
PEX5
YWHAZ
MIR129-1
ZCCHC8
BBS2
ABCG8
LAMP1
GALK1
AVPR2
EIF4EBP1
TEP1
PRKCG
PTPN1
FBP1

AP3D1
STAG2
TUBA1A
FMO3
PTPN3
CSK
MRTFA
LINC00941
MMAA
F13A1
MADD
PLA2G7
MIR181A2
DIS3L2
NARS1
ADCY10
CERS1
C5
SRPK1
CDC73
TPM2
MYL2
AMACR
CDC6
SIX3
IL25
MIR628
MIR506
PEPD
WWTR1
IFT81
PIEZO1
ETS2
MRC1
HSPA9
MIR367
IGH
MIR1207
LYVE1
BBS9
TNKS
AHCY
HIC1

AP3D1
STAG2
TUBA1A
FMO3
PTPN3
CSK
MRTFA
LINC00941
MMAA
F13A1
MADD
PLA2G7
MIR181A2
DIS3L2
NARS1
ADCY10
CERS1
C5
SRPK1
CDC73
TPM2
MYL2
AMACR
CDC6
SIX3
IL25
MIR628
MIR506
PEPD
WWTR1
IFT81
PIEZO1
ETS2
MRC1
HSPA9
MIR367
IGH
MIR1207
LYVE1
BBS9
TNKS
AHCY
HIC1

MIR381
PRDM1
RBPJ
MIR519D
USF2
SMARCD2
FOXC1
IFNL3
KIR2DL1
HGSNAT
RPS14
GHR
CXCR6
MID1
INSR
DYSF
NOP2
IFNAR2
HNRNPA1
S1PR1
MMP19
TBX3
AICDA
HNMT
TPI1
KAT6B
RPS24
ERN1
RBCK1
BBS4
KRT10
KLK3
LDHA
JUNB
LINC01133
ATP5F1A
MDM4
BCKDHB
SNORD44
LOXL1
HAVCR1
SP3
REST

MIR381
PRDM1
RBPJ
MIR519D
USF2
SMARCD2
FOXC1
IFNL3
KIR2DL1
HGSNAT
RPS14
GHR
CXCR6
MID1
INSR
DYSF
NOP2
IFNAR2
HNRNPA1
S1PR1
MMP19
TBX3
AICDA
HNMT
TPI1
KAT6B
RPS24
ERN1
RBCK1
BBS4
KRT10
KLK3
LDHA
JUNB
LINC01133
ATP5F1A
MDM4
BCKDHB
SNORD44
LOXL1
HAVCR1
SP3
REST

F9
ABCG5
TFAP2B
IGHMBP2
CTSC
DSTYK
PTH1R
RPS19
SCARB2
GLS
ATP13A3
CD70
PAK6
MIR22HG
ZNF469
ODAD3
INTS6
RASA2
GJA5
GPI
MED12
BRDT
TMEM237
LOC107133510
UBE2I
HOXA10
FUCA1
MIR520A
TRRAP
MIR502
FKBP1A
RFX5
SLC7A11
SLC16A1
SELPLG
TPM3
DZIP1L
MIR505
BRMS1
ZRSR2
ACTG1
F10
TMEM231
F9
ABCG5
TFAP2B
IGHMBP2
CTSC
DSTYK
PTH1R
RPS19
SCARB2
GLS
ATP13A3
CD70
PAK6
MIR22HG
ZNF469
ODAD3
INTS6
RASA2
GJA5
GPI
MED12
BRDT
TMEM237
LOC107133510
UBE2I
HOXA10
FUCA1
MIR520A
TRRAP
MIR502
FKBP1A
RFX5
SLC7A11
SLC16A1
SELPLG
TPM3
DZIP1L
MIR505
BRMS1
ZRSR2
ACTG1
F10
TMEM231

SALL4
IRAK4
TNFRSF13C
NEB
MIR877
DEFB1
SOX18
PMM2
PBRM1
FCGR2B
PPARGC1A
RELN
IQSEC1
SERPINF2
PAX9
SHROOM3
RFC2
SERPINE2
B3GAT1
SFN
IL12RB2
LOC106099062
TFPI
CYP11A1
CDH23
SNCA
MIR411
RELB
NRP2
MEIS2
SF3A3
CA8
PLXND1
MLH3
IGF2BP2
BBS10
FANCI
TGFBR3
SCARB1
SIX2
ARHGAP24
MAP3K1
ZBTB7A

SALL4
IRAK4
TNFRSF13C
NEB
MIR877
DEFB1
SOX18
PMM2
PBRM1
FCGR2B
PPARGC1A
RELN
IQSEC1
SERPINF2
PAX9
SHROOM3
RFC2
SERPINE2
B3GAT1
SFN
IL12RB2
LOC106099062
TFPI
CYP11A1
CDH23
SNCA
MIR411
RELB
NRP2
MEIS2
SF3A3
CA8
PLXND1
MLH3
IGF2BP2
BBS10
FANCI
TGFBR3
SCARB1
SIX2
ARHGAP24
MAP3K1
ZBTB7A

ACKR1	ACKR1
KCNH2	KCNH2
VEGFB	VEGFB
ABT1	ABT1
DSC2	DSC2
AATF	AATF
LINC00963	LINC00963
MIR873	MIR873
C1QB	C1QB
RNF43	RNF43
AHSG	AHSG
ARHGDIA	ARHGDIA
MIR671	MIR671
CUL5	CUL5
ANKS6	ANKS6
EPRS1	EPRS1
HMBS	HMBS
DGUOK	DGUOK
NUP85	NUP85
HLA-DRB3	HLA-DRB3
HTATIP2	HTATIP2
MUC2	MUC2
LPP	LPP
ATF6	ATF6
HOXA1	HOXA1
IL13RA2	IL13RA2
PCCA	PCCA
PDGFRL	PDGFRL
PKP2	PKP2
S100A13	S100A13
SOCS2	SOCS2
HSPA1L	HSPA1L
IRAK3	IRAK3
ICOSLG	ICOSLG
SPINK1	SPINK1
MUC3A	MUC3A
MIR627	MIR627
CPLANE1	CPLANE1
SLC39A7	SLC39A7
MIR493	MIR493
IL23A	IL23A
NR3C2	NR3C2
PLA2R1	PLA2R1

HOXB2
MDC1
MAP2
RRAS
POLH
KRT13
DUOX2
SPRY4
DNASE1
CD58
GAS1
TCTN1
ELAVL2
IFT80
APOH
IRF2BP2
GH1
GNPTAB
MIR605
PARK7
PRDM14
RPL36A-HNRNPH2
MYO18A
SGCB
PRMT1
SIX5
ATF3
NOVA1
NPY
HDAC3
CHRND
TOB1
DPAGT1
MIR551B
CEP120
NFKBIL1
TAFAZZIN
ADORA2B
GNS
HSD3B7
GRK2
PLS3
APLN

HOXB2
MDC1
MAP2
RRAS
POLH
KRT13
DUOX2
SPRY4
DNASE1
CD58
GAS1
TCTN1
ELAVL2
IFT80
APOH
IRF2BP2
GH1
GNPTAB
MIR605
PARK7
PRDM14
RPL36A-HNRNPH2
MYO18A
SGCB
PRMT1
SIX5
ATF3
NOVA1
NPY
HDAC3
CHRND
TOB1
DPAGT1
MIR551B
CEP120
NFKBIL1
TAFAZZIN
ADORA2B
GNS
HSD3B7
GRK2
PLS3
APLN

CDK8
TPBG
CAPNS1
POLR2A
MATR3
MT-CYB
WNT2B
CDH3
KHSRP
MBP
EFNB2
LIPC
LTBP2
MX1
PAFAH1B1
BDKRB2
FMR1
FUBP1
METTL13
MIR206
MYO1E
ELAVL3
AGA
CALM3
IQGAP1
APRT
MIR187
NUP133
CEBPE
AIRE
ID3
SETD1A
ACD
BBS7
LRPPRC
CCL26
SUZ12
MIR92B
RHEB
MAPK10
CTDSPL
SRP54
A2M

CDK8
TPBG
CAPNS1
POLR2A
MATR3
MT-CYB
WNT2B
CDH3
KHSRP
MBP
EFNB2
LIPC
LTBP2
MX1
PAFAH1B1
BDKRB2
FMR1
FUBP1
METTL13
MIR206
MYO1E
ELAVL3
AGA
CALM3
IQGAP1
APRT
MIR187
NUP133
CEBPE
AIRE
ID3
SETD1A
ACD
BBS7
LRPPRC
CCL26
SUZ12
MIR92B
RHEB
MAPK10
CTDSPL
SRP54
A2M

RAB5A
LMNB1
HOXB5
ID2
PMS1
JMJD6
MIR1228
TMPO
MPV17
NHEJ1
NUP98
CRIPTO
PAH
NECTIN4
PIN1
CTTN
CFL1
NSD3
CHST14
MIR16-2
CX3CL1
MIR498
SGSH
BCL2A1
CLCN1
B9D1
HAX1
SPON2
MIR489
SLC1A5
MSN
NOTCH4
PPBP
NOX1
AIMP1
NANOG
DAB2
TRAF3IP1
MIR515-1
DDAH2
SPHK1
SHOC2
MIR508

RAB5A
LMNB1
HOXB5
ID2
PMS1
JMJD6
MIR1228
TMPO
MPV17
NHEJ1
NUP98
CRIPTO
PAH
NECTIN4
PIN1
CTTN
CFL1
NSD3
CHST14
MIR16-2
CX3CL1
MIR498
SGSH
BCL2A1
CLCN1
B9D1
HAX1
SPON2
MIR489
SLC1A5
MSN
NOTCH4
PPBP
NOX1
AIMP1
NANOG
DAB2
TRAF3IP1
MIR515-1
DDAH2
SPHK1
SHOC2
MIR508

TTK
CISH
C4BPA
CD99
MIR654
MMP21
LINC01082
TSHR
MIR1224
CLDN5
SFRP1
LRP2
BCS1L
GATA1
WNT11
GSS
LMX1B
SKI
MAPK7
MYOCD
TAGLN
ATIC
CTSH
NAT10
CEP164
NR2F2
ACTN1
PYCR1
DVL3
VANGL1
ACADVL
SETDB1
FBL
RPL3
SELENBP1
IGF2BP1
MIR512-1
TPSAB1
GNE
SLC6A4
LTBR
ALG9
HOXB13
TTK
CISH
C4BPA
CD99
MIR654
MMP21
LINC01082
TSHR
MIR1224
CLDN5
SFRP1
LRP2
BCS1L
GATA1
WNT11
GSS
LMX1B
SKI
MAPK7
MYOCD
TAGLN
ATIC
CTSH
NAT10
CEP164
NR2F2
ACTN1
PYCR1
DVL3
VANGL1
ACADVL
SETDB1
FBL
RPL3
SELENBP1
IGF2BP1
MIR512-1
TPSAB1
GNE
SLC6A4
LTBR
ALG9
HOXB13

MIR744
ANXA3
HMMR
CLIC1
TRIM65
PSTPIP1
ANKRD26
MIR326
MIR371A
IL15RA
DTNBP1
PEX6
IQGAP3
PTPRO
NBAS
BHLHE40
SERPING1
EDN2
HOXA11
TAB2
NUP214
SMC3
AMBP
SPDEF
GLIS2
TRIM32
MIR383
KAT6A
GRK5
IHH
MIR454
GPC4
SCT
HPS3
LRRC37A2
ADAM10
IKBKE
PXDNL
RLBP1
GRN
GLUL
TBL1XR1
COL6A3
MIR744
ANXA3
HMMR
CLIC1
TRIM65
PSTPIP1
ANKRD26
MIR326
MIR371A
IL15RA
DTNBP1
PEX6
IQGAP3
PTPRO
NBAS
BHLHE40
SERPING1
EDN2
HOXA11
TAB2
NUP214
SMC3
AMBP
SPDEF
GLIS2
TRIM32
MIR383
KAT6A
GRK5
IHH
MIR454
GPC4
SCT
HPS3
LRRC37A2
ADAM10
IKBKE
PXDNL
RLBP1
GRN
GLUL
TBL1XR1
COL6A3

SS18
SMN1
TAPBP
PSAT1
AXDND1
RHO
TNS1
WNT9B
TJP2
EGLN1
NUP160
PTPA
HOXD13
ATP6V0A2
CFC1
MED1
IL1RAP
ESS2
LRP1
CSPP1
GTF2I
MCAM
PEX13
ATRIP
FSTL1
LONP1
NPPC
SOX10
TIGIT
BLOC1S6
DUOX1
KIF7
TMEM43
CASK
IFT74
DNAJB11
DEFA1
CYP2C9
ZBTB16
DNM1L
GABRA3
GGH
SKIL

SS18
SMN1
TAPBP
PSAT1
AXDND1
RHO
TNS1
WNT9B
TJP2
EGLN1
NUP160
PTPA
HOXD13
ATP6V0A2
CFC1
MED1
IL1RAP
ESS2
LRP1
CSPP1
GTF2I
MCAM
PEX13
ATRIP
FSTL1
LONP1
NPPC
SOX10
TIGIT
BLOC1S6
DUOX1
KIF7
TMEM43
CASK
IFT74
DNAJB11
DEFA1
CYP2C9
ZBTB16
DNM1L
GABRA3
GGH
SKIL

RNASEH2B	RNASEH2B
UTP4	UTP4
LOC111674477	LOC111674477
PSMD3	PSMD3
ROCK1	ROCK1
RRAS2	RRAS2
MIR487A	MIR487A
BICC1	BICC1
FZD7	FZD7
SRCAP	SRCAP
NKX2-6	NKX2-6
NUP107	NUP107
KCNH1	KCNH1
TK2	TK2
SULT1A3	SULT1A3
TNFRSF4	TNFRSF4
REL	REL
MAP1B	MAP1B
CASP2	CASP2
MIR432	MIR432
IFT122	IFT122
CXCL17	CXCL17
CARD9	CARD9
WSPAR	WSPAR
SLCO1B3	SLCO1B3
FGF5	FGF5
MAGI2	MAGI2
RIGI	RIGI
RAD21	RAD21
NTF3	NTF3
DEK	DEK
ANO1	ANO1
ALDOB	ALDOB
NDRG1	NDRG1
RPLP0	RPLP0
APOC3	APOC3
ANG	ANG
CYB5R3	CYB5R3
HCFC1	HCFC1
GREB1L	GREB1L
MIR874	MIR874
MFN2	MFN2
TPO	TPO

NAB2
MUS81
BBS12
NCL
MIR1247
SNRPA
G6PC3
FTO
KLRD1
EIF4A1
BBS5
RRM2B
SF3B4
ALCAM
OLR1
PTAFR
CYP7A1
IL32
CYP51A1
MGA
BCL11B
VCAN
KLF5
DDC
RPL26
EHMT2
HEXA
SRP72
BRD2
PHF6
NEUROD1
PPOX
BTG1
VKORC1
LIMK1
SLC12A6
CCL7
NID1
TRIM21
RAD18
SDC2
TRIM24
TOP2B

NAB2
MUS81
BBS12
NCL
MIR1247
SNRPA
G6PC3
FTO
KLRD1
EIF4A1
BBS5
RRM2B
SF3B4
ALCAM
OLR1
PTAFR
CYP7A1
IL32
CYP51A1
MGA
BCL11B
VCAN
KLF5
DDC
RPL26
EHMT2
HEXA
SRP72
BRD2
PHF6
NEUROD1
PPOX
BTG1
VKORC1
LIMK1
SLC12A6
CCL7
NID1
TRIM21
RAD18
SDC2
TRIM24
TOP2B

CAMKK2	CAMKK2
SPECC1L	SPECC1L
GFI1	GFI1
ERAP1	ERAP1
TNNC1	TNNC1
TMSB4X	TMSB4X
CPT2	CPT2
PCGF2	PCGF2
MYSM1	MYSM1
SYNE2	SYNE2
ISG15	ISG15
CAMP	CAMP
CCL21	CCL21
PAK4	PAK4
ETV5	ETV5
SSTR1	SSTR1
MIR660	MIR660
MLPH	MLPH
S100A6	S100A6
BACH1	BACH1
CD79B	CD79B
NCOA2	NCOA2
FZD1	FZD1
SLX4	SLX4
RPS26	RPS26
CUL3	CUL3
GDPD3	GDPD3
YTHDF1	YTHDF1
U2AF2	U2AF2
SLC34A1	SLC34A1
BCL11A	BCL11A
ORAI1	ORAI1
CELF2	CELF2
SIRT6	SIRT6
TACSTD2	TACSTD2
IL1R2	IL1R2
NTN1	NTN1
MAPT	MAPT
TLR10	TLR10
HSPA1B	HSPA1B
COL4A6	COL4A6
KDM3B	KDM3B
GCLM	GCLM

HNRNPC
NOD1
FREM1
PFN1
ARVCF
YWHAQ
CDC5L
ZIC2
ASCC1
SLCO2B1
ARL13B
BGN
MFF-DT
MIR876
ITPA
NR4A2
MIR589
PRKCZ
RBM20
ERCC6L2
STAT2
COLQ
POLR1G
MAPKBP1
SLCO2A1
CALM2
LOC113633877
MIR769
HNRNPK
ADORA2A
ADNP
MUC6
ITPR1
ADCYAP1
SEMA4D
MPI
AARS1
CARD10
LAMP3
TFAM
TMEM138
KIR2DS4
BCKDHA
HNRNPC
NOD1
FREM1
PFN1
ARVCF
YWHAQ
CDC5L
ZIC2
ASCC1
SLCO2B1
ARL13B
BGN
MFF-DT
MIR876
ITPA
NR4A2
MIR589
PRKCZ
RBM20
ERCC6L2
STAT2
COLQ
POLR1G
MAPKBP1
SLCO2A1
CALM2
LOC113633877
MIR769
HNRNPK
ADORA2A
ADNP
MUC6
ITPR1
ADCYAP1
SEMA4D
MPI
AARS1
CARD10
LAMP3
TFAM
TMEM138
KIR2DS4
BCKDHA

TNFRSF25
PLP1
CTHRC1
IQCB1
FANCM
FLII
SLK
SUMF1
DCDC2
PHB1
DAG1
EIF2S1
CRH
RPS6
PRPF8
COL7A1
NUMA1
CLCN5
BAZ1B
IRS2
C3AR1
PYROXD1
SMCHD1
OXA1L
PCSK1
SEPTIN9
ADAMTSL1
CTBP1
YTHDC2
RFXAP
ATF1
VPS33B
KDM6B
LINC00922
LPAR3
MDK
NSUN2
UHRF1
CAST
PSEN1
TNFSF4
ZFP36
GSTO1

TNFRSF25
PLP1
CTHRC1
IQCB1
FANCM
FLII
SLK
SUMF1
DCDC2
PHB1
DAG1
EIF2S1
CRH
RPS6
PRPF8
COL7A1
NUMA1
CLCN5
BAZ1B
IRS2
C3AR1
PYROXD1
SMCHD1
OXA1L
PCSK1
SEPTIN9
ADAMTSL1
CTBP1
YTHDC2
RFXAP
ATF1
VPS33B
KDM6B
LINC00922
LPAR3
MDK
NSUN2
UHRF1
CAST
PSEN1
TNFSF4
ZFP36
GSTO1

WDR1	WDR1
DAXX	DAXX
GORAB	GORAB
DNAJA1	DNAJA1
SLC25A3	SLC25A3
ACTR3C	ACTR3C
PLA2G10	PLA2G10
SLC39A8	SLC39A8
EEF1A1	EEF1A1
ERF	ERF
PDE4D	PDE4D
PEX26	PEX26
UBE2T	UBE2T
PUF60	PUF60
HOXB4	HOXB4
NFATC1	NFATC1
FGF19	FGF19
GAS6	GAS6
HUS1	HUS1
EIF4G1	EIF4G1
UGT2B17	UGT2B17
CD83	CD83
KCNMA1	KCNMA1
ADSL	ADSL
CEP89	CEP89
MANBA	MANBA
KRT3	KRT3
ZNF423	ZNF423
RIN3	RIN3
FAM111B	FAM111B
CORO1C	CORO1C
GLDC	GLDC
LTA4H	LTA4H
PLEK2	PLEK2
MARCKS	MARCKS
BLVRB	BLVRB
GOLPH3	GOLPH3
NOP53	NOP53
EPHB2	EPHB2
MIR520D	MIR520D
USP53	USP53
KCNJ6	KCNJ6
ALOX15	ALOX15

CD209
EPX
PTBP1
GNLY
MIR524
ALOX15B
LOC113664106
MAP3K7
MIR504
CDON
CYP3A7
ROCK2
GCK
NEK2
CD244
ATF4
PRDM16
PPT1
FOSL2
DHX16
INHBA
TRIM33
IFT27
SLC26A3
ABCB10
USP9X
LMOD1
DDX1
MAGEC2
NFIX
NR0B1
SEC63
VPS13B
TFDP1
LILRB4
PEX11B
HELLS
HTR2A
PSEN2
PRRC2A
SIRPA
ZNF667-AS1
ZNRF3

CD209
EPX
PTBP1
GNLY
MIR524
ALOX15B
LOC113664106
MAP3K7
MIR504
CDON
CYP3A7
ROCK2
GCK
NEK2
CD244
ATF4
PRDM16
PPT1
FOSL2
DHX16
INHBA
TRIM33
IFT27
SLC26A3
ABCB10
USP9X
LMOD1
DDX1
MAGEC2
NFIX
NR0B1
SEC63
VPS13B
TFDP1
LILRB4
PEX11B
HELLS
HTR2A
PSEN2
PRRC2A
SIRPA
ZNF667-AS1
ZNRF3

PHEX
CXCL16
RPL19
MAP3K20
HPS5
MIR509-1
FGF17
SMARCC2
GFRA1
PNPLA3
NUP93
PCCB
LMNB2
PHLDA2
LPAR2
TSG101
ING4
NCOA1
SATB1
ARID2
FGG
MHRT
CPQ
KIF11
MIR217
IGKC
SCN4A
AQP2
CUL4A
CDIPT
P2RY2
ACTN2
SOST
CTSF
KDM5D
PRSS2
NDUFAF6
ACP1
ERGIC3
HK1
SPARCL1
TACR1
HOXA13

PHEX
CXCL16
RPL19
MAP3K20
HPS5
MIR509-1
FGF17
SMARCC2
GFRA1
PNPLA3
NUP93
PCCB
LMNB2
PHLDA2
LPAR2
TSG101
ING4
NCOA1
SATB1
ARID2
FGG
MHRT
CPQ
KIF11
MIR217
IGKC
SCN4A
AQP2
CUL4A
CDIPT
P2RY2
ACTN2
SOST
CTSF
KDM5D
PRSS2
NDUFAF6
ACP1
ERGIC3
HK1
SPARCL1
TACR1
HOXA13

DYNC1H1
L1CAM
FCGRT
PRDX2
ARX
REV3L
SEMA3C
FZD4
FER
SPN
AGK
SCD
PROX1
WNK1
CBFB
PTPRU
TWNK
ROBO2
EXOSC3
DDAH1
TREM1
SYNPO
AQP4
TRAPPC11
RPS6KA1
SETX
HJV
KIF1B
SNRNP70
VIRMA
PAX3
RNASEH2A
H6PD
CARMIL2
FMO2
CANX
CD33
SCO2
ACLY
MIR518A1
ROR1
SEC16A
CALCRL
DYNC1H1
L1CAM
FCGRT
PRDX2
ARX
REV3L
SEMA3C
FZD4
FER
SPN
AGK
SCD
PROX1
WNK1
CBFB
PTPRU
TWNK
ROBO2
EXOSC3
DDAH1
TREM1
SYNPO
AQP4
TRAPPC11
RPS6KA1
SETX
HJV
KIF1B
SNRNP70
VIRMA
PAX3
RNASEH2A
H6PD
CARMIL2
FMO2
CANX
CD33
SCO2
ACLY
MIR518A1
ROR1
SEC16A
CALCRL

MIR767
TFF3
PMAIP1
JMJD1C
BCL3
MCC
ST3GAL4
KANSL1
PTK7
SLC11A2
MRI1
CGAS
ACVR1B
GART
MIR548B
TAF15
PEX16
SLC22A4
RPS10
TFR2
ALDH18A1
DNAJB1
NGFR
PSMC3
CTSA
CDC20
OCRL
ULK1
FGF22
RPL18
YTHDF2
BSND
GLUD1
DHX8
PARD3
LEPQTL1
CALML5
PEX3
FZD6
RPL35A
KDM5C
MAPK6
CDK9
MIR767
TFF3
PMAIP1
JMJD1C
BCL3
MCC
ST3GAL4
KANSL1
PTK7
SLC11A2
MRI1
CGAS
ACVR1B
GART
MIR548B
TAF15
PEX16
SLC22A4
RPS10
TFR2
ALDH18A1
DNAJB1
NGFR
PSMC3
CTSA
CDC20
OCRL
ULK1
FGF22
RPL18
YTHDF2
BSND
GLUD1
DHX8
PARD3
LEPQTL1
CALML5
PEX3
FZD6
RPL35A
KDM5C
MAPK6
CDK9

NLRP1
VPS33A
DNAJB6
IGLL1
ADIPOR1
GPX2
MAOA
MEIS1
SAA4
FOXD2-AS1
CLCN7
PTGIR
POGZ
RPS11
IL19
DDX39B
DVL2
RBP1
GFPT1
HOXB3
VASP
MYL9
ITGA7
CD7
EFNA1
HLA-DMA
MYL3
POLA2
SGCG
ZMPSTE24
JAM3
HNRNPUL1
MIR541
IPO8
SLC29A3
UBAP2
HTRA1
SSX2
NHERF1
PTN
PAX7
HTR4
MIR450B

NLRP1
VPS33A
DNAJB6
IGLL1
ADIPOR1
GPX2
MAOA
MEIS1
SAA4
FOXD2-AS1
CLCN7
PTGIR
POGZ
RPS11
IL19
DDX39B
DVL2
RBP1
GFPT1
HOXB3
VASP
MYL9
ITGA7
CD7
EFNA1
HLA-DMA
MYL3
POLA2
SGCG
ZMPSTE24
JAM3
HNRNPUL1
MIR541
IPO8
SLC29A3
UBAP2
HTRA1
SSX2
NHERF1
PTN
PAX7
HTR4
MIR450B

DCTN1	DCTN1
PLA2G1B	PLA2G1B
DNAI7	DNAI7
FERMT3	FERMT3
SERPINB9	SERPINB9
MTM1	MTM1
ARG2	ARG2
MASP1	MASP1
H3-3A	H3-3A
UGT1A8	UGT1A8
HOGA1	HOGA1
CLEC12A	CLEC12A
CD3G	CD3G
TARBP2	TARBP2
PDHA1	PDHA1
THRA	THRA
CNTF	CNTF
CSNK2A1	CSNK2A1
TFF1	TFF1
DBH	DBH
SP110	SP110
TDG	TDG
NUMB	NUMB
FLG	FLG
CYP21A2	CYP21A2
SKIC3	SKIC3
KCNJ11	KCNJ11
ZNF276	ZNF276
E2F4	E2F4
TNFSF13	TNFSF13
HRG	HRG
PRSS1	PRSS1
UBC	UBC
TNFRSF14	TNFRSF14
HYAL3	HYAL3
MIR329-1	MIR329-1
GLO1	GLO1
MCPH1	MCPH1
HSD3B2	HSD3B2
MAPK9	MAPK9
SLC31A1	SLC31A1
FGB	FGB
TNIP1	TNIP1

CALML3
MIR642A
FLOT1
NFKBIE
CCT8
HSPB8
RAP1A
TCAP
NDUFS4
DCBLD2
FOXE1
PEX14
RBM8A
MIR488
MIR539
PEX19
LDB3
PARVA
MAP1LC3A
ADAMTS8
SULT2B1
ZC3H4
EFL1
TNFRSF18
RAB8A
TIAM1
KRT16
ABCF1
SPEN
MACC1
CEP55
PDHX
PAK1
EFTUD2
EPHA4
BMAL1
TXNRD2
SLC10A1
CA2
MCOLN1
CAPN2
JPH2
MYOG

CALML3
MIR642A
FLOT1
NFKBIE
CCT8
HSPB8
RAP1A
TCAP
NDUFS4
DCBLD2
FOXE1
PEX14
RBM8A
MIR488
MIR539
PEX19
LDB3
PARVA
MAP1LC3A
ADAMTS8
SULT2B1
ZC3H4
EFL1
TNFRSF18
RAB8A
TIAM1
KRT16
ABCF1
SPEN
MACC1
CEP55
PDHX
PAK1
EFTUD2
EPHA4
BMAL1
TXNRD2
SLC10A1
CA2
MCOLN1
CAPN2
JPH2
MYOG

DPM1
PSMA7
RFC3
RIPK4
TAB1
TCN2
SLC22A3
PPP2R5C
LRP6
RPN1
TET1
HHAT
ADK
BBIP1
ITGAE
ILF3
TRIM27
FMOD
APCS
USP22
FGF13
CCNK
CYSLTR2
FUT8
GCH1
CDC42BPA
SULF2
TPP1
ENPP1
OPA1
RFX1
EMILIN1
MIR513A1
BCORL1
DOCK6
RHD
STC1
JUND
ASAH2
SPG7
KCNJ1
RFC4
MAP1LC3B
DPM1
PSMA7
RFC3
RIPK4
TAB1
TCN2
SLC22A3
PPP2R5C
LRP6
RPN1
TET1
HHAT
ADK
BBIP1
ITGAE
ILF3
TRIM27
FMOD
APCS
USP22
FGF13
CCNK
CYSLTR2
FUT8
GCH1
CDC42BPA
SULF2
TPP1
ENPP1
OPA1
RFX1
EMILIN1
MIR513A1
BCORL1
DOCK6
RHD
STC1
JUND
ASAH2
SPG7
KCNJ1
RFC4
MAP1LC3B

PTENP1
KDM4A
THRB
CRAT
RBBP4
CSNK2B
MIR410
LPA
COQ2
YWHAG
GSTO2
SART1
NEUROG3
COQ8A
PDX1
GZMA
PPP1R12A
NEXN
AFDN
ADD3
ARTN
HUWE1
SLC25A4
PRG2
MYH14
TRPM6
GYPA
PLOD2
MCM7
CHTOP
IGF2-AS
MYLK2
USH2A
GRWD1
MED13L
SEMA3A
BCAR1
BUB3
TRIM37
MAP3K5
MEST
LOC126861898
JARID2

PTENP1
KDM4A
THRB
CRAT
RBBP4
CSNK2B
MIR410
LPA
COQ2
YWHAG
GSTO2
SART1
NEUROG3
COQ8A
PDX1
GZMA
PPP1R12A
NEXN
AFDN
ADD3
ARTN
HUWE1
SLC25A4
PRG2
MYH14
TRPM6
GYPA
PLOD2
MCM7
CHTOP
IGF2-AS
MYLK2
USH2A
GRWD1
MED13L
SEMA3A
BCAR1
BUB3
TRIM37
MAP3K5
MEST
LOC126861898
JARID2

PPP1R10
PEX10
UTS2
CD93
HEY2
TRIB2
MIR3613
TLE3
SLC4A1
MLXIPL
C1QA
POLD3
RPS17
IL37
VPS45
SCAP
GATM
TNK2
LAT
TM4SF1
PTGER3
NUDT6
PRKAA2
DDX39A
TFEB
RFC1
RGS6
CXCL3
CD200
GPC6
SLC4A2
PLD1
B3GAT3
DLD
RPL34
DUT
AARS2
ABCC9
MCM5
CTSE
SIN3A
TPD52
NCOA6

PPP1R10
PEX10
UTS2
CD93
HEY2
TRIB2
MIR3613
TLE3
SLC4A1
MLXIPL
C1QA
POLD3
RPS17
IL37
VPS45
SCAP
GATM
TNK2
LAT
TM4SF1
PTGER3
NUDT6
PRKAA2
DDX39A
TFEB
RFC1
RGS6
CXCL3
CD200
GPC6
SLC4A2
PLD1
B3GAT3
DLD
RPL34
DUT
AARS2
ABCC9
MCM5
CTSE
SIN3A
TPD52
NCOA6

RPLP2	RPLP2
COMP	COMP
AQP9	AQP9
SCTR	SCTR
CHERP	CHERP
RTKN2	RTKN2
CKB	CKB
PC	PC
DDB1	DDB1
CTAG2	CTAG2
UBA2	UBA2
MAF	MAF
ADAMTS10	ADAMTS10
POLR1A	POLR1A
INPP5D	INPP5D
RPS15A	RPS15A
EEF2	EEF2
ETFA	ETFA
UCP2	UCP2
CD2	CD2
IL21R	IL21R
MIR3187	MIR3187
KRT4	KRT4
RAPGEF5	RAPGEF5
VIPAS39	VIPAS39
EHHADH	EHHADH
LTB	LTB
PEX2	PEX2
SULF1	SULF1
MTUS1	MTUS1
ARPC2	ARPC2
ADGRE5	ADGRE5
LTB4R	LTB4R
GNRH1	GNRH1
RPTOR	RPTOR
HPX	HPX
CXCL6	CXCL6
TAGLN2	TAGLN2
HIF3A	HIF3A
EPHB3	EPHB3
LRRC8A	LRRC8A
ALG8	ALG8
ANKRD1	ANKRD1

ANGPTL4	ANGPTL4
PIGR	PIGR
PURA	PURA
PSMA6	PSMA6
ERC1	ERC1
BLK	BLK
VSIR	VSIR
PAF1	PAF1
PABPC1	PABPC1
HGS	HGS
CDT1	CDT1
MIR103A2	MIR103A2
SSTR4	SSTR4
ADIPOR2	ADIPOR2
LRMDA	LRMDA
PSMC5	PSMC5
KIR2DS2	KIR2DS2
PSMD12	PSMD12
TRIB1	TRIB1
TCF7	TCF7
ADPRH	ADPRH
CDC45	CDC45
ADGRF5	ADGRF5
MBNL1	MBNL1
GJB1	GJB1
SFPQ	SFPQ
NEBL	NEBL
TOE1	TOE1
GAL	GAL
GPR87	GPR87
RICTOR	RICTOR
NUAK1	NUAK1
FPR2	FPR2
TRAF3IP2	TRAF3IP2
CCL19	CCL19
PEX7	PEX7
DSG1	DSG1
LAPTM4B	LAPTM4B
ACTG2	ACTG2
KHDRBS1	KHDRBS1
GARS1	GARS1
MYBL2	MYBL2
ZBTB24	ZBTB24

LZTFL1
RAB11A
TBCD
RARS2
PLK2
VTCN1
PPP2CA
SLC3A2
EFS
CYB5A
TSPO
ARHGEF7
RPL15
CCT3
TNS4
MMADHC
DDX18
DNAJC21
COL6A2
NONO
ARHGAP26
CYP2C8
FLRT3
TRIB3
ANK1
COQ7
FERMT1
SLC9A1
KIR3DL2
ADGRG1
EGFL6
IFT43
PITX1
POMT2
MACF1
FKTN
ACKR3
SHBG
G3BP1
CDC37
DYRK2
MUC7
EXOC4

LZTFL1
RAB11A
TBCD
RARS2
PLK2
VTCN1
PPP2CA
SLC3A2
EFS
CYB5A
TSPO
ARHGEF7
RPL15
CCT3
TNS4
MMADHC
DDX18
DNAJC21
COL6A2
NONO
ARHGAP26
CYP2C8
FLRT3
TRIB3
ANK1
COQ7
FERMT1
SLC9A1
KIR3DL2
ADGRG1
EGFL6
IFT43
PITX1
POMT2
MACF1
FKTN
ACKR3
SHBG
G3BP1
CDC37
DYRK2
MUC7
EXOC4

DHX9	DHX9
IQANK1	IQANK1
CETP	CETP
DOT1L	DOT1L
COX5A	COX5A
PAG1	PAG1
CUL7	CUL7
CRK	CRK
GLP1R	GLP1R
GLCCI1	GLCCI1
ZNF384	ZNF384
MT-CO1	MT-CO1
PROS1	PROS1
GGA3	GGA3
RNASEH2C	RNASEH2C
DDIT4	DDIT4
RBM14	RBM14
ELF3	ELF3
RPL27	RPL27
CPOX	CPOX
CPNE1	CPNE1
CFP	CFP
PCNT	PCNT
LPAR6	LPAR6
RHOC	RHOC
NUDC	NUDC
TONSL	TONSL
FGF16	FGF16
BIN1	BIN1
CYP26A1	CYP26A1
QARS1	QARS1
LILRB1	LILRB1
RPLP1	RPLP1
SSTR3	SSTR3
LSM2	LSM2
MIR939	MIR939
IL17RC	IL17RC
IRX1	IRX1
TALDO1	TALDO1
LILRB2	LILRB2
TUBB6	TUBB6
LTK	LTK
UNC45A	UNC45A

LIMA1
IMMT
PCBP1
SIAH1
EGLN3
SON
SATB2
ABCC11
MMP16
ROBO4
PCSK2
ACAT1
ACTR3B
PEX12
SMAD1
RLF
CYP2F1
DDX17
PKLR
IKZF2
SLC12A3
CDC27
ACKR2
ATG5
SMURF2
MIR616
HMSD
FIG4
ZNFX1
FGF6
EIF3A
RPL10
FPR1
CNP
YME1L1
GAD2
TBL2
BTG3
KIR2DS5
GNB3
IAPP
GSE1
RPS15

LIMA1
IMMT
PCBP1
SIAH1
EGLN3
SON
SATB2
ABCC11
MMP16
ROBO4
PCSK2
ACAT1
ACTR3B
PEX12
SMAD1
RLF
CYP2F1
DDX17
PKLR
IKZF2
SLC12A3
CDC27
ACKR2
ATG5
SMURF2
MIR616
HMSD
FIG4
ZNFX1
FGF6
EIF3A
RPL10
FPR1
CNP
YME1L1
GAD2
TBL2
BTG3
KIR2DS5
GNB3
IAPP
GSE1
RPS15

MSC-AS1	MSC-AS1
GCG	GCG
BRWD1	BRWD1
PON2	PON2
PVR	PVR
SLC25A1	SLC25A1
ATF6B	ATF6B
SPRY1	SPRY1
RTN4	RTN4
TAL1	TAL1
HLA-H	HLA-H
IKZF3	IKZF3
CCN6	CCN6
FCHO1	FCHO1
RHBDF2	RHBDF2
IVL	IVL
NCR3	NCR3
HNRNPL	HNRNPL
CGB3	CGB3
DPEP1	DPEP1
ETHE1	ETHE1
SUN2	SUN2
H3-4	H3-4
NUMBL	NUMBL
TBX18	TBX18
SYVN1	SYVN1
TBX6	TBX6
RPS27	RPS27
GLIS3	GLIS3
CFHR1	CFHR1
PSMB4	PSMB4
SENP1	SENP1
AAAS	AAAS
CLDN3	CLDN3
EXOSC10	EXOSC10
SRSF3	SRSF3
HNRNPDL	HNRNPDL
PDLIM1	PDLIM1
NTN4	NTN4
PFKP	PFKP
GSTM2	GSTM2
DDX3Y	DDX3Y
KRIT1	KRIT1

SLC22A2
NREP
CSNK1D
BLNK
CPB2
RBBP8
RPL31
SGCA
FBXO11
MIR449A
POLR2B
SCRIB
ACSL4
SMPD2
ISL1
KPNB1
B4GAT1
ANAPC1
NOS1AP
RBBP6
STYK1
JAG2
FLNC-AS1
SPTBN1
RPS7
KLRC2
RECQL
PAK5
DBT
WDR5
RBM26
AHSP
CCN3
FUT2
PFKFB3
USP11
TNFSF14
FUT3
NR5A2
MLLT3
LPO
HSD3B1
PRG4

SLC22A2
NREP
CSNK1D
BLNK
CPB2
RBBP8
RPL31
SGCA
FBXO11
MIR449A
POLR2B
SCRIB
ACSL4
SMPD2
ISL1
KPNB1
B4GAT1
ANAPC1
NOS1AP
RBBP6
STYK1
JAG2
FLNC-AS1
SPTBN1
RPS7
KLRC2
RECQL
PAK5
DBT
WDR5
RBM26
AHSP
CCN3
FUT2
PFKFB3
USP11
TNFSF14
FUT3
NR5A2
MLLT3
LPO
HSD3B1
PRG4

FERMT2
CDH11
SYNM
TIE1
NR2C2
PFAS
CD248
SSTR5
RBX1
ASH2L
MGAT5
SHOX2
REG3A
DST
EGFL7
ESPL1
ALKBH5
RAD17
HMGN2
FNIP1
RNASEL
ORM1
CELSR1
IARS1
MRAS
MSMB
PLTP
UBTF
ELF5
NUBP2
BCL2L2
EIF4B
SIPA1
BNC2
GNB1
GINS2
SP7
XK
KIR2DS1
MSX2
CALB1
CRTC2
PPP1CA
FERMT2
CDH11
SYNM
TIE1
NR2C2
PFAS
CD248
SSTR5
RBX1
ASH2L
MGAT5
SHOX2
REG3A
DST
EGFL7
ESPL1
ALKBH5
RAD17
HMGN2
FNIP1
RNASEL
ORM1
CELSR1
IARS1
MRAS
MSMB
PLTP
UBTF
ELF5
NUBP2
BCL2L2
EIF4B
SIPA1
BNC2
GNB1
GINS2
SP7
XK
KIR2DS1
MSX2
CALB1
CRTC2
PPP1CA

TNPO3
EIF1AX
CERK
CCR8
STK40
GNL3
SPAAR
NAGA
STX3
DDX27
RAMP2
HTR3A
FCGR1A
SMPD3
MYO1B
MAPKAPK2
CAD
CGA
RTRAF
ZNF875
MFAP5
PRODH
BTLA
CLEC6A
UGT1A
SCP2
ASB16-AS1
NELFA
USB1
RGCC
CCL1
AOC1
IL34
CEBPD
ADAM8
TOPBP1
TPM4
MARK2
PDGFC
ALOX12B
RPL22
C19orf48P
CARMN

TNPO3
EIF1AX
CERK
CCR8
STK40
GNL3
SPAAR
NAGA
STX3
DDX27
RAMP2
HTR3A
FCGR1A
SMPD3
MYO1B
MAPKAPK2
CAD
CGA
RTRAF
ZNF875
MFAP5
PRODH
BTLA
CLEC6A
UGT1A
SCP2
ASB16-AS1
NELFA
USB1
RGCC
CCL1
AOC1
IL34
CEBPD
ADAM8
TOPBP1
TPM4
MARK2
PDGFC
ALOX12B
RPL22
C19orf48P
CARMN

LCT	LCT
PDHB	PDHB
LMO2	LMO2
SEC23B	SEC23B
MIR518D	MIR518D
MYO5B	MYO5B
TFE3	TFE3
RDX	RDX
MIR100HG	MIR100HG
SIRT2	SIRT2
VAPA	VAPA
POGLUT1	POGLUT1
SASH1	SASH1
ATAD3B	ATAD3B
HMGCL	HMGCL
MTA2	MTA2
PADI2	PADI2
KDM4C	KDM4C
ALAS2	ALAS2
LUM	LUM
PELP1	PELP1
ARCN1	ARCN1
ZKSCAN3	ZKSCAN3
ANXA4	ANXA4
LBX1	LBX1
SMARCD1	SMARCD1
HLA-DRB4	HLA-DRB4
DDX59	DDX59
MDH2	MDH2
LEF1	LEF1
MAP3K11	MAP3K11
MAP3K3	MAP3K3
WWP2	WWP2
RUVBL1	RUVBL1
PBK	PBK
RHOD	RHOD
DCAF8	DCAF8
FABP4	FABP4
SF3A1	SF3A1
SLCO1A2	SLCO1A2
ARHGAP31	ARHGAP31
GATAD1	GATAD1
BCLAF1	BCLAF1

DNTT	DNTT
CRMP1	CRMP1
ATP1A1	ATP1A1
RBSN	RBSN
CLDN16	CLDN16
ADD1	ADD1
SLC4A4	SLC4A4
RPS16	RPS16
USP4	USP4
CPM	CPM
ETV1	ETV1
P4HA2	P4HA2
FIP1L1	FIP1L1
SLC5A1	SLC5A1
PSMB1	PSMB1
WRNIP1	WRNIP1
TLL1	TLL1
BAMBI	BAMBI
TNFSF8	TNFSF8
OSMR	OSMR
ABO	ABO
CIRBP	CIRBP
CTBP1-DT	CTBP1-DT
UBR5	UBR5
NKX2-8	NKX2-8
RBM15	RBM15
RBM39	RBM39
NCR1	NCR1
KIR3DS1	KIR3DS1
PIAS1	PIAS1
KMT2B	KMT2B
GPR68	GPR68
UGCG	UGCG
VSIG4	VSIG4
ATP2B4	ATP2B4
IGES	IGES
MAFB	MAFB
TACC3	TACC3
AGPS	AGPS
ADAM9	ADAM9
ACTR2	ACTR2
AOPEP	AOPEP
HOXA-AS3	HOXA-AS3

RPS3
CCR9
AFF1
PUM3
NR1H3
PSMB10
ITGA1
ADAMTS1
MEPCE
RECQL5
TXNIP
RAN
BMP5
NCAPD2
MIR101-2
CREBZF
ADAMTS17
TNFSF9
CD1D
HOXB7
AKR1A1
CALU
ZMYND8
ANGPTL2
GALT
ANKRD11
SF1
SIK1
MYEOV
PARP2
NUP205
RARG
DMPK
TNFAIP8
KRT1
GAK
GEMIN4
MSI2
EEF1G
PDIA3
CCNI
RNLS
KDM2B

RPS3
CCR9
AFF1
PUM3
NR1H3
PSMB10
ITGA1
ADAMTS1
MEPCE
RECQL5
TXNIP
RAN
BMP5
NCAPD2
MIR101-2
CREBZF
ADAMTS17
TNFSF9
CD1D
HOXB7
AKR1A1
CALU
ZMYND8
ANGPTL2
GALT
ANKRD11
SF1
SIK1
MYEOV
PARP2
NUP205
RARG
DMPK
TNFAIP8
KRT1
GAK
GEMIN4
MSI2
EEF1G
PDIA3
CCNI
RNLS
KDM2B

ZNF143
SCYL1
PLCB4
PRDM2
SEPSECS
MEF2D
RPS28
ADCY6
NAA15
S1PR3
SLC12A2
CD27-AS1
KNSTRN
NFAT5
SEPTIN7
KPNA2
CALM1
NRIP1
RPS20
DDX47
LRIG2
NELFE
M6PR
TEAD1
MT-CO2
UFD1
RPS18
MERTK
TCF20
ALDH3A1
SLC2A2
TAX1BP1
ATP5F1B
RNF40
NIPAL4
PER1
DYRK1A
TTF2
CEP104
VANGL2
CDK5RAP3
CCN5
CCL8

ZNF143
SCYL1
PLCB4
PRDM2
SEPSECS
MEF2D
RPS28
ADCY6
NAA15
S1PR3
SLC12A2
CD27-AS1
KNSTRN
NFAT5
SEPTIN7
KPNA2
CALM1
NRIP1
RPS20
DDX47
LRIG2
NELFE
M6PR
TEAD1
MT-CO2
UFD1
RPS18
MERTK
TCF20
ALDH3A1
SLC2A2
TAX1BP1
ATP5F1B
RNF40
NIPAL4
PER1
DYRK1A
TTF2
CEP104
VANGL2
CDK5RAP3
CCN5
CCL8

VDAC2	VDAC2
EEF2K	EEF2K
KAT8	KAT8
LGR4	LGR4
GAP43	GAP43
MIR26A2	MIR26A2
KAT2A	KAT2A
PCBP2	PCBP2
AKAP1	AKAP1
PLEK	PLEK
SPTB	SPTB
CEMIP	CEMIP
MFAP2	MFAP2
CSNK1E	CSNK1E
	DBR1
	SLC67A1
	CHRNA3
	SARS2
