# Supplementary material for: Barbaloin Alleviates Lung Ischemia-Reperfusion Injury by Dual-Targeting IL-6 and PNP
Source: Int J Mol Sci. 2026 Jun 10;27(12):5276. doi: 10.3390/ijms27125276 (PMC13300021; doi:10.3390/ijms27125276)
Supplement: Supplementary file 1 [file ijms-27-05276-s001.zip › Supplementary Table S3.pdf]

Supplementary Table S3. Barbaloin Predicted Target Genes from Four Databases (PharmMapper, SEA, SuperPred, SwissTargetPrediction)

PharmMapper	SEA	SuperPred	SwissTargetPrediction	Union
NUDT9	SLC5A2	TDP1	EPHX2	NUDT9
CTSV	NRAS	HSD17B10	TYR	CTSV
MAPK8	SLC28A3	APEX1	CA2	MAPK8
CHEK1	CDA	CTSD	CA7	CHEK1
STS	SLC5A1	NR1I2	CA1	STS
MAPK10	SLC5A11	ADORA1	CA12	MAPK10
TGFBR2	TOP1	TRIM24	CA14	TGFBR2
EPHB4	UMPS	TOP2A	CA9	EPHB4
CA2	TYR	CLK4	CA4	CA2
APOA2	HRAS	NFKB1	CA13	APOA2
CA1	CA12	THRA	CA5A	CA1
CLPP	SLC5A4	DUSP3	SLC5A2	CLPP
EGFR	CA14	HDAC5	CA6	EGFR
GSTP1	CA9	CNR1	HRAS	GSTP1
CTSD	IL2	NTRK3	ADORA3	CTSD
PNP	ALDH1B1	KDM1A	IGFBP3	PNP
ALB	CA1	HDAC8	LGALS3	ALB
MMP3	ALDH1A2	KLF5	LGALS9	MMP3
BCHE	IL6	HSP90AA1	ADORA2A	BCHE
PPIA	SLC37A4	SLC6A5	CA3	PPIA
BACE1	FGF1	F13A1	MMP13	BACE1
DDX6	TNNC1	GLRA1	MMP1	DDX6
HSP90AA1	TNNI3	CHUK	MMP7	HSP90AA1
MTAP	TNNT2	GRIN1	MMP12	MTAP
FAP	CA2	ABCC1	MMP8	FAP
CFB	FGF2	CSNK2B	CDA	CFB
RTN4R	CA13	CDK1	ADA	RTN4R
CMA1	P4HB	GRIA2	ADK	CMA1
F2	ERAP1	CDK5	SLC29A1	F2
ESR1	PYGM	ALOX12	NOX4	ESR1
PGR	HKDC1	KIF11	PNP	PGR
CA12	YARS1	DPP9	CAMKK2	CA12
PIM1		NR3C2	IGFBP5	PIM1
QPCT		TTR	HSP90AA1	QPCT
CTSS		HDAC10	ADORA2B	CTSS
FCAR		GRK5	CDK2	FCAR
ICAM2		SCD	FUCA1	ICAM2
NR1H2		CCNE1	GAA	NR1H2
AKR1B1		NFE2L2	MANBA	AKR1B1
MAOB		KLK1	AMPD3	MAOB

CDK2	C5AR1	TYMP	CDK2
TREM1	CNR2	TOP1	TREM1
HK1	SCN3A	MGAM	HK1
CSNK1G2	METAP2	SLC5A4	CSNK1G2
CTSB	S1PR4	ERN1	CTSB
TTR	CDC25C	SLC28A3	TTR
DHFR	NTSR2	MMP3	DHFR
CDK5R1	PTPN7	MMP9	CDK5R1
AMD1	PSMB1	ADAM17	AMD1
HSPA8	ACHE	GBA	HSPA8
GSR	PDGFRA	PYGL	GSR
ADH1B	EGLN1	GART	ADH1B
CASP7	CAPN1	HK2	CASP7
PDE3B	ADAM10	HK1	PDE3B
TGFBR1	MDM4	GPR35	TGFBR1
KIF11	SLC2A1	POLB	KIF11
DPP4	TFPI	TP53	DPP4
ANG	CACNA1B	AMY2A	ANG
BCAT2	BLM	SELE	BCAT2
DCK	GCK	KDM4C	DCK
ESRRG	TBXA2R	MMP2	ESRRG
ADH5	SCN4A	FOLH1	ADH5
NPR3	MAOA	MCL1	NPR3
GC	P2RX4	MME	GC
IMPA1	STING1	SI	IMPA1
FGFR1	MAPK1	P2RX3	FGFR1
SRC	IDO1	YARS	SRC
BAG1	TDO2	HPRT1	BAG1
AMY1A	ERAP1	OGA	AMY1A
AMY1B	BMP2K	PFKFB3	AMY1B
AMY1C	GABRA1	BCL2A1	AMY1C
PDE4B	QRFPR	EIF4H	PDE4B
TYMS	ITK	PABPC1	TYMS
IGLV2-8	CHRM5	LGALS4	IGLV2-8
Esr2	CDC25B	TYMS	Esr2
REN	TACR2	LGALS8	REN
BMP7	PKN1	RNASEH1	BMP7
NQO1	TLR8	F2	NQO1
PLA2G10	CYP3A4	AGTR1	PLA2G10
PYGL	TOP1	ATIC	PYGL
KDR	GLS	ALOX12	KDR
DUSP6	AOC3	DAO	DUSP6
MIF	PIN1	KDM3A	MIF

CFD	PRCP	MAP2K1	CFD
PDE4D	LDHB	KDM5B	PDE4D
HADH	MAP2K2	KDM4D	HADH
NOS3	SLC1A3	CDK2	NOS3
DAPK1	TACR1	CCNA1	DAPK1
TYMP	CTSS	CCNA2	TYMP
IGF1R	GPBAR1	HSPA8	IGF1R
CCNA2	PLAT	HSPA5	CCNA2
AKR1C1	MARK4	PTPN2	AKR1C1
CTSK	GPR17	MKNK2	CTSK
PAH	PROC	CHEK1	PAH
MAPK14	AVPR1B	IMPDH1	MAPK14
CBR1	CBX4	IMPDH2	CBR1
SHBG	P2RX7	CASP6	SHBG
GSK3B	HDAC11	CASP7	GSK3B
PPARD	TLR4	CASP8	PPARD
CDA	ABL1	METAP2	CDA
SOD2	MIF	AMD1	SOD2
PPARG	ZAP70	CASP1	PPARG
MMP7	TYRO3		MMP7
PPP5C	CHRNA1		PPP5C
PLK1			PLK1
DHODH			DHODH
FNTA			FNTA
MMP8			MMP8
AKR1C3			AKR1C3
ADH1C			ADH1C
HDAC8			HDAC8
REG1A			REG1A
LCK			LCK
B3GAT1			B3GAT1
AMY2A			AMY2A
AKT1			AKT1
SORD			SORD
EPHA2			EPHA2
IMPDH2			IMPDH2
MAN1B1			MAN1B1
CES1			CES1
F10			F10
ISG20			ISG20
SULT2A1			SULT2A1
HEXB			HEXB
LGALS7			LGALS7

LGALS7B	LGALS7B
AR	AR
ADK	ADK
HSD17B1	HSD17B1
YARS1	YARS1
HSD11B1	HSD11B1
TPH1	TPH1
EPHX2	EPHX2
AHCY	AHCY
PDPK1	PDPK1
ATOX1	ATOX1
CTSG	CTSG
LGALS2	LGALS2
PLAU	PLAU
RAC2	RAC2
SEC14L2	SEC14L2
PTPN1	PTPN1
JAK3	JAK3
PRKACA	PRKACA
ADAM17	ADAM17
BST1	BST1
HPN	HPN
TNK2	TNK2
RHOA	RHOA
ACP3	ACP3
FABP4	FABP4
ALDH2	ALDH2
ELANE	ELANE
PARP1	PARP1
MMP12	MMP12
CSNK2A1	CSNK2A1
CDK6	CDK6
HMGCR	HMGCR
SYK	SYK
UCK2	UCK2
GPI	GPI
LDHB	LDHB
GNPDA1	GNPDA1
NR1I2	NR1I2
NOS2	NOS2
PDHB	PDHB
RNASE4	RNASE4
PGF	PGF

IL2
CASP3
FHIT
NR3C2
AZGP1
C1R
JAK2
HCK
ITK
F7
RXRA
RHEB
S100A9
NCS1
PCK1
RAB11A
ABO
LGALS3
ARG2
MMP13
MET
ABO
TGM3
CYP2C9
IGF1
FKBP1A
THRB
CTNNA1
NQO2
SELP
ALDOA
RNASE3
DTYMK
TK1
DPEP1
SSE1
MAPKAPK2
ADAM17
TPI1
ZAP70
PTPN11
MTHFD1
UMPS

IL2
CASP3
FHIT
NR3C2
AZGP1
C1R
JAK2
HCK
ITK
F7
RXRA
RHEB
S100A9
NCS1
PCK1
RAB11A
ABO
LGALS3
ARG2
MMP13
MET
TGM3
CYP2C9
IGF1
FKBP1A
THRB
CTNNA1
NQO2
SELP
ALDOA
RNASE3
DTYMK
TK1
DPEP1
SSE1
MAPKAPK2
TPI1
ZAP70
PTPN11
MTHFD1
UMPS
CAT
SHMT1

CAT	CCNT1
SHMT1	GSTA1
CCNT1	CCL5
GSTA1	ARHGAP1
CCL5	HINT1
ARHGAP1	GSTT2B
HINT1	AKR1C2
GSTT2B	PDE5A
AKR1C2	APRT
PDE5A	BIRC7
APRT	C1S
BIRC7	PDK2
C1S	SERPINA1
PDK2	LYZ
SERPINA1	KYAT1
LYZ	FECH
KYAT1	CHIT1
FECH	RAB5A
CHIT1	ST14
RAB5A	GALE
ST14	ABL1
GALE	NR3C1
ABL1	ERBB4
NR3C1	CSK
ERBB4	FDPS
CSK	WARS1
FDPS	ATIC
WARS1	CLK1
ATIC	IMPDH1
CLK1	CD1A
IMPDH1	CD209
CD1A	FABP6
CD209	STAT1
FABP6	INSR
STAT1	SRM
THRB	MMP9
INSR	SETD7
SRM	THRA
MMP9	PADI4
SETD7	FABP3
THRA	XIAP
PADI4	PIK3R1
FABP3	ITPKA

XIAP
PIK3R1
ITPKA
CYP2C8
KIT
NMNAT1
ARG1
MMP16
PLEKHA4
PAK6
PAPSS1
ARSA
MMP2
TRAPPC3
PRKCQ
KAT2B
HRAS
HAGH
SDS
PLA2G2A
GP1BA
PITPNA
GSTA3
ARL5A
NT5M
AKT2
SULT2B1
SULT1E1
ACADM
GSTM2
GCK
PPCDC
MAPK1
CBS
PTK2

CYP2C8
KIT
NMNAT1
ARG1
MMP16
PLEKHA4
PAK6
PAPSS1
ARSA
MMP2
TRAPPC3
PRKCQ
KAT2B
HRAS
HAGH
SDS
PLA2G2A
GP1BA
PITPNA
GSTA3
ARL5A
NT5M
AKT2
SULT2B1
SULT1E1
ACADM
GSTM2
GCK
PPCDC
MAPK1
CBS
PTK2
SLC5A2
NRAS
SLC28A3
SLC5A1
SLC5A11
TOP1
TYR
SLC5A4
CA14
CA9
ALDH1B1

ALDH1A2
IL6
SLC37A4
FGF1
TNNC1
TNNI3
TNNT2
FGF2
CA13
P4HB
ERAP1
PYGM
HKDC1
TDP1
HSD17B10
APEX1
ADORA1
TRIM24
TOP2A
CLK4
NFKB1
DUSP3
HDAC5
CNR1
NTRK3
KDM1A
KLF5
SLC6A5
F13A1
GLRA1
CHUK
GRIN1
ABCC1
CSNK2B
CDK1
GRIA2
CDK5
ALOX12
DPP9
HDAC10
GRK5
SCD
CCNE1

NFE2L2
KLK1
C5AR1
CNR2
SCN3A
METAP2
S1PR4
CDC25C
NTSR2
PTPN7
PSMB1
ACHE
PDGFRA
EGLN1
CAPN1
ADAM10
MDM4
SLC2A1
TFPI
CACNA1B
BLM
TBXA2R
SCN4A
MAOA
P2RX4
STING1
IDO1
TDO2
BMP2K
GABRA1
QRFPR
CHRM5
CDC25B
TACR2
PKN1
TLR8
CYP3A4
GLS
AOC3
PIN1
PRCP
MAP2K2
SLC1A3

TACR1
GPBAR1
PLAT
MARK4
GPR17
PROC
AVPR1B
CBX4
P2RX7
HDAC11
TLR4
TYRO3
CHRNA1
CA7
CA4
CA5A
CA6
ADORA3
IGFBP3
LGALS9
ADORA2A
CA3
MMP1
ADA
SLC29A1
NOX4
CAMKK2
IGFBP5
ADORA2B
FUCA1
GAA
MANBA
AMPD3
MGAM
ERN1
GBA
GART
HK2
GPR35
POLB
TP53
SELE
KDM4C

FOLH1
MCL1
MME
SI
P2RX3
YARS
HPRT1
OGA
PFKFB3
BCL2A1
EIF4H
PABPC1
LGALS4
LGALS8
RNASEH1
AGTR1
DAO
KDM3A
MAP2K1
KDM5B
KDM4D
CCNA1
HSPA5
PTPN2
MKNK2
CASP6
CASP8
CASP1
