# Supplementary material for: Barbaloin Alleviates Lung Ischemia-Reperfusion Injury by Dual-Targeting IL-6 and PNP
Source: Int J Mol Sci. 2026 Jun 10;27(12):5276. doi: 10.3390/ijms27125276 (PMC13300021; doi:10.3390/ijms27125276)
Supplement: Supplementary file 1 [file ijms-27-05276-s001.zip › Supplementary Table S4.pdf]

Supplementary Table S4. Antibodies used for western blot and immunofluorescence.

Target Protein	Host Species	Modification	Source (Catalog No.)	Dilution
Phospho-NF-κB p65	Rabbit	Ser536	Cell Signaling Technology (3033)	1:1000
NF-κB p65	Rabbit	Total	Cell Signaling Technology (3034)	1:1000
NLRP3	Rabbit	-	Immunoway (YT5382)	1:1000
IL-1β (for WB)	Rabbit	-	Abclonal (A20527)	1:1000
Cleaved caspase-1	Rabbit	-	Proteintech (22915-1-AP)	1:1000
GAPDH	Mouse	-	Proteintech (60004-1-Ig)	1:10000
IL-1β (for IHC)	Rabbit	-	Servicebio (GB11113)	1:800
IL-6 (for IHC)	Rabbit	-	Servicebio (GB11117)	1:400
