# Supplementary material for: Barbaloin Alleviates Lung Ischemia-Reperfusion Injury by Dual-Targeting IL-6 and PNP
Source: Int J Mol Sci. 2026 Jun 10;27(12):5276. doi: 10.3390/ijms27125276 (PMC13300021; doi:10.3390/ijms27125276)
Supplement: Supplementary file 1 [file ijms-27-05276-s001.zip › Supplementary Table S5.pdf]

Supplementary Table S5. Primers Used for qRT-PCR.

Gene	Species	Sequence	
		Forward (5'→3')	Reverse (5'→3')
Gapdh	Mouse	CTCGCTCCTGGAAGATGGTGATGG	TCCAGTATGACTCCACTCACGGCA
Il-6	Mouse	CCTTCTTGGGACTGATGCTGGTGA	TGGGAGTGGTATCCTCTGTGAAGT
Pnp	Mouse	TCCCAGAAGTTATCGTCGCAAGGC	CCGGCATCCAGAACTTCCATGTGA
Il-1β	Mouse	TGACCCTGAGCGACCTGTCTTGGC	TCCCCAGGGCATGTTAAGGAGCTC
Gapdh	Human	CCTGCCGTCTAGAAAAACCTGCCA	GTGGGTGTCGCTGTTGAAGTCAGA
Il-6	Human	CTTCGGCAAATGTAGCATGGGCAC	TTCTGTGCCCAGTGGACAGGTTTC
Pnp	Human	TTATGGCCAGCATTCCACTCCCTG	ACTTGGGTCTTGTGTGGGAGATGC
