# Supplementary material for: Barbaloin Alleviates Lung Ischemia-Reperfusion Injury by Dual-Targeting IL-6 and PNP
Source: Int J Mol Sci. 2026 Jun 10;27(12):5276. doi: 10.3390/ijms27125276 (PMC13300021; doi:10.3390/ijms27125276)
Supplement: Supplementary file 1 [file ijms-27-05276-s001.zip › Supplementary Material.pdf]

# Supplementary Material for

## Barbaloin Alleviates Lung Ischemia-Reperfusion Injury by Dual-Targeting IL-6 and PNP

This file includes:
Supplementary Figures S1-3, Tables S1-5

**Figure S1:**

Transcriptomic data from two datasets (GSE127003 and GSE145989; 113 paired samples from cold ischemia and 1-2 h post-reperfusion) were merged and subjected to batch-effect correction. PCA demonstrated that before correction, samples clustered strongly by dataset, whereas after correction they overlapped extensively and separated clearly by biological group (cold ischemia vs. reperfusion). Variance distribution across principal components became more balanced following correction. These results confirm that technical batch effects were effectively removed, enabling subsequent analyses to focus on biological differences between ischemia and reperfusion phases.

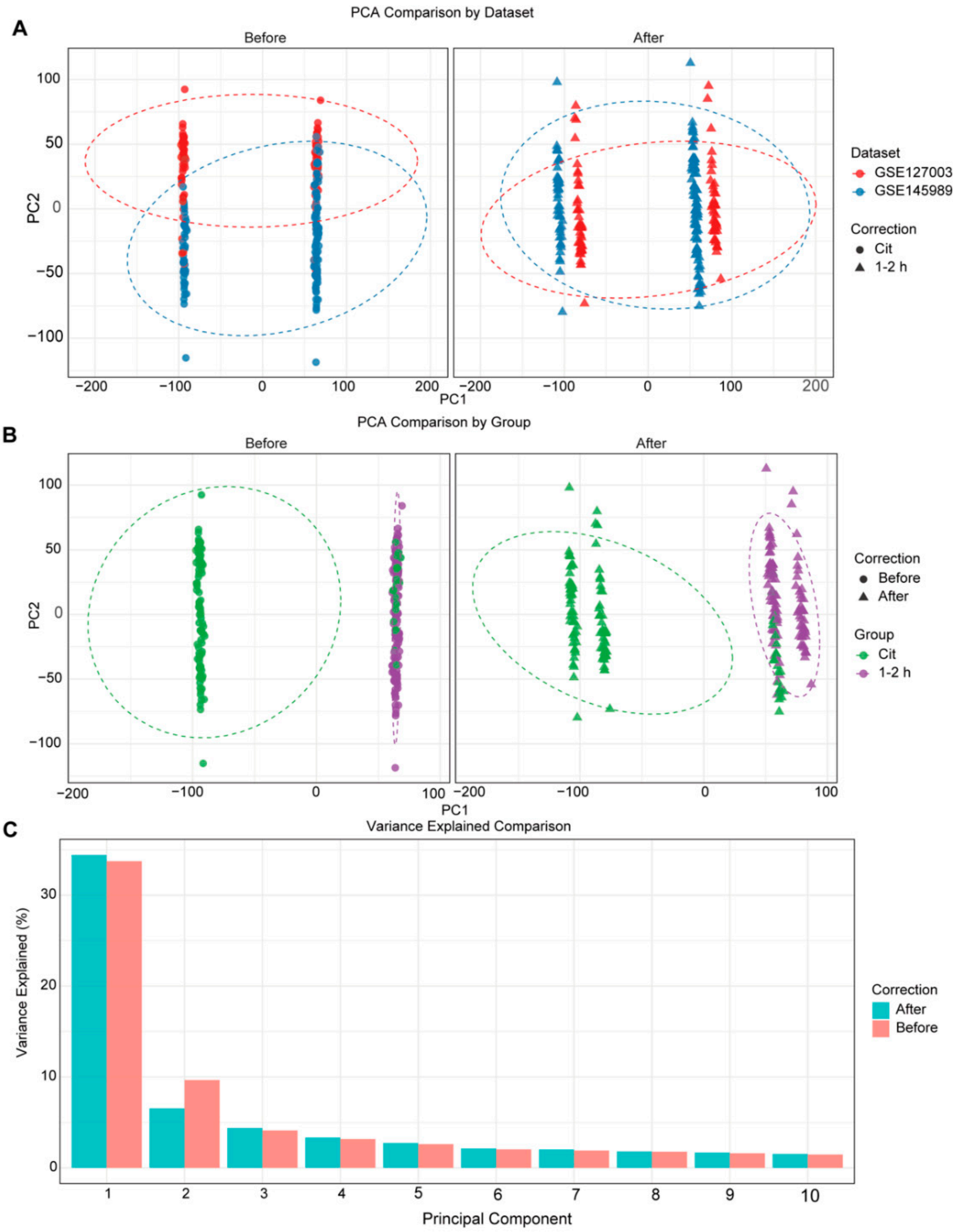


**Figure S1. Batch correction validation of merged transcriptomic datasets from human lung transplant biopsies.** (A) Principal Component Analysis (PCA) plots of samples stratified by dataset, before (left) and after (right) batch correction. (B) PCA plots of samples stratified by experimental group (cold ischemia vs. reperfusion), before (left) and after (right) batch correction. (C) Bar plot showing the variance explained by each principal component, before and after batch correction.

**Figure S2:**

Functional enrichment analysis showed distinct roles for the two key modules: MEyellow genes were enriched in acute innate immunity (e.g., neutrophil activation), whereas MEturquoise genes were associated with broad inflammatory signaling (e.g., IL-17, TNF, NF-κB) (Supplementary Figure. S2). These modules likely represent complementary pathological axes in early graft injury.

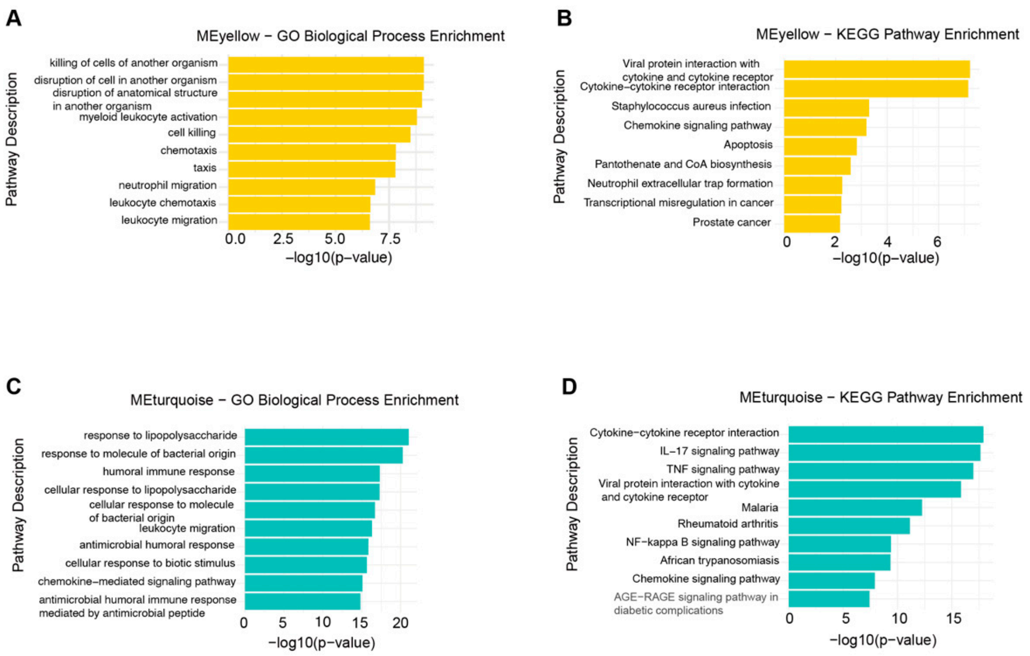


**Figure S2. Functional enrichment analysis of WGCNA-derived lung transplant-related co-expression modules**. (A) Gene Ontology (GO) Biological Process enrichment results for the MEyellow module. (B) Kyoto Encyclopedia of Genes and Genomes (KEGG) pathway enrichment results for the MEyellow module. (C) GO Biological Process enrichment results for the MEturquoise module. (D) KEGG pathway enrichment results for the MEturquoise module.

**Figure S3:**

Supplementary Figure S3 summarizes the quality control (QC) metrics for the single-cell RNA-seq dataset from six lung transplant patients (12 samples: CIT and 2h reperfusion). (A) The number of cells retained per sample after QC filtering ranged from 2,757 to 14,342. (B) The cell retention rate post-QC varied between 70.7 % and 84 %. (C) The number of genes detected per sample after QC was similar between the CIT and 2-h reperfusion groups (range: 19,890-23,632 genes). (D) UMAP visualization of all integrated cells, colored by sample origin, shows well-mixed clustering, indicating successful data integration and minimal batch-specific bias.

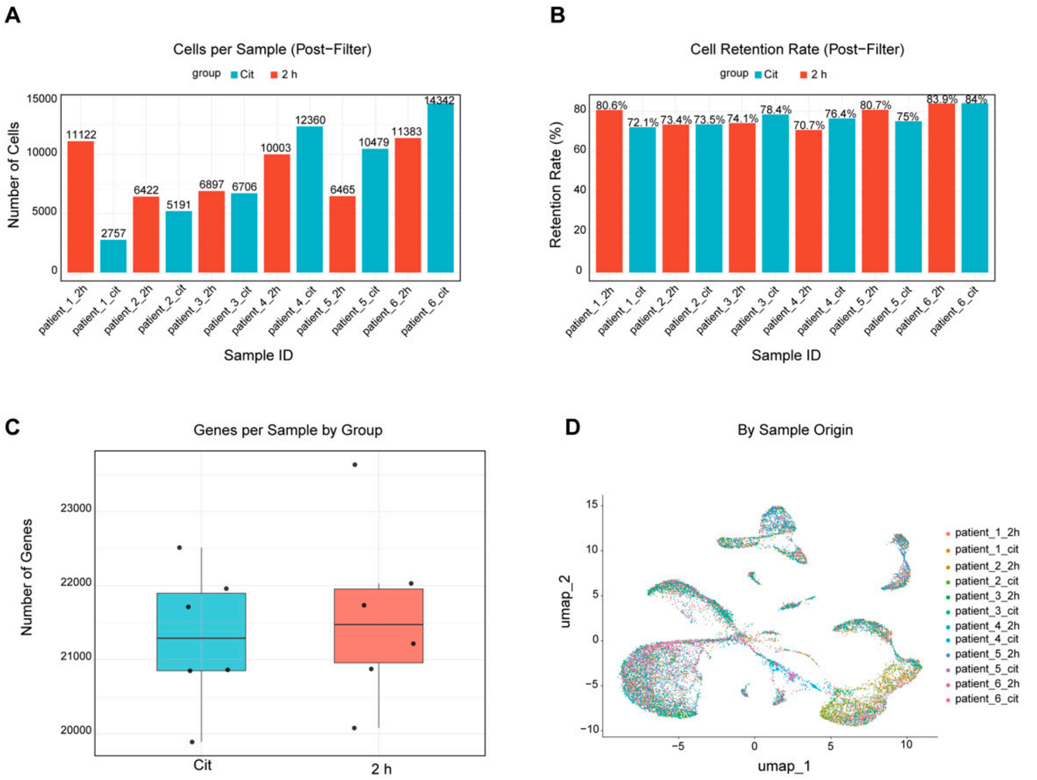


**Figure S3. Quality control and dimensionality reduction of single-cell sequencing dat**a. (A) Number of cells per sample after quality control (QC) and filtering. (B) Cell retention rate of each sample post QC and filtering. (C) Box plot showing gene count distribution per sample after QC. (D) UMAP plot of cells colored by 12 lung transplant samples.

**Table S1. Differentially Expressed Genes (DEGs) and Phenotype-Linked WGCNA Module Genes (MEyellow & MEturquoise)**

| DEGs | MEyellow | MEturquoise |
|---|---|---|
| PLAGL2 | ADGRG3 | ACKR1 |
| RIPK2 | ANKRD22 | ADAMTS9-AS1 |
| NFKBIE | AQP9 | ADM |
| TNFAIP3 | BCL2A1 | AGTR2 |
| GNA13 | BTNL8 | AMPD1 |
| TICAM1 | CAMP | ANGPTL4 |
| KBTBD2 | CCDC178 | ANKRD1 |
| ITPRIP | CD177 | APOBEC3B |
| PMAIP1 | CDA | ARC |
| JMJD6 | CLC | ARID5A |
| RASGEF1B | CLEC4D | ATF3 |
| PPP1R15A | CMTM2 | ATP13A4-AS1 |
| ZBTB43 | CRISP3 | B3GALT2 |
| B3GNT5 | CST7 | BABAM2-AS1 |
| IER5 | CX3CR1 | BAG3 |
| NFKB2 | CXCR1 | BDKRB2 |
| ARL5B | CXCR2 | BIRC3 |
| RLIM | CYTL1 | C15orf48 |
| ELL | DHRS9 | C2CD4A |
| RLF | ENPP2 | C9orf152 |
| RNF148 | FCAR | CA3 |
| GADD45B | FCN1 | CCK |
| SSH1 | FFAR2 | CCL11 |
| ALMS1-IT1 | FGFBP2 | CCL2 |
| GCH1 | FOLR3 | CCL20 |
| MCL1 | FPR2 | CCL4 |
| BAG3 | GJB6 | CCL5 |
| CSF3 | GLT1D1 | CCL7 |
| EPOP | GNLY | CCL8 |
| IRF1 | GPR84 | CCN4 |
| PELI1 | GZMB | CD274 |
| DNAJB1 | GZMH | CD69 |
| AGO2 | IL18R1 | CD83 |
| MSX1 | IL18RAP | CD8A |
| ZBTB2 | IL1R2 | CH25H |
| IER3 | IL1RL1 | CHI3L1 |
| CCNH | KLB | CLDN1 |
| SLC30A1 | KLRF1 | CSF3 |
| SLC2A3 | KRT23 | CX3CL1 |
| CCNL1 | LCN2 | CXCL1 |

SLC25A33
HSPB8
RELB
MT1F
CNKSR3
RRAD
KLHL15
ATF3
CD83
RNF19B
YOD1
ELF3
TIPARP
MT1HL1
DNAJA1
STX11
NFKBIZ
CHIC2
DUSP5
ABL2
UGCG
ATG4C
IRAK2
ICAM1
HOXD1
ARID5A
ZNF267
KDM6B
BCL10
MT1M
GABPB1
FOSL1
GUCY1A1
CXCL2
MT1G
CXCL8
SESN2
SOCS2
ZNF573
BIRC3
PPIF
ARC
FPGT
REL

LMNB1
LRG1
LY6H
MGAM
MMP8
MMP9
MSLN
MYRIP
NFE2
NKG7
NTM
OLAH
ORM1
OSM
PADI4
PF4
PI3
PPBP
PRF1
PROK2
RGL4
RGS18
RIPOR2
RNASE2
RUBCNL
S100A12
S100P
SAMSN1
SELL
TMEM71
TOX3
TRIM58
VNN1
VNN2

CXCL10
CXCL11
CXCL3
CXCL5
CXCL8
CXCL9
DACT1
DKK1
DNAJA4
DNAJB1
DUSP2
DUSP5
EGR1
EGR2
EGR3
ELF3
EREG
ESM1
F8
FGA
FGFBP1
FGG
FNDC1
FOS
FOSB
FOSL1
G0S2
GADD45B
GALNT15
GCH1
GCLM
GDF10
GEM
GFPT2
GJB2
GPA33
GPIHBP1
GPR171
GPR34
GZMK
HAS1
HAS2
HBB
HBD

ITPKC
CLCF1
KBTBD8
DUSP2
MT1X
PIM2
SERPINE1
SBNO2
SAMSN1
NOCT
IL1A
MAP3K8
PLK3
MAFF
ZFP36
MXD1
B4GALT5
ASH1L-AS1
CDKN1A
SOCS3
FOSL2
C3orf52
PPRC1
CXCL3
LIMK2
OSGEPL1
SPRY4
HSPA6
UPP1
VPS37B
SERTAD1
PHF13
TLNRD1
NFKB1
VMP1
EPHA2
PTGS2
NAMPT
GJB6
SLC25A25
IL1RAP
ZBTB21
ZNF165
SPSB1

HMOX1
HOXA5
HOXC6
HRCT1
HSD17B2
HSPA6
HSPH1
HTATSF1P2
ICAM1
ICAM4
IDO1
IER3
IFNG
IGHD
IGHM
IGLL3P
IL1A
IL1B
IL24
IL6
IRF1
ITGA2
ITLN1
ITPKC
JCHAIN
KCNN2
KDM6B
KRT80
LIF
LINC00844
LIPG
LRRC32
LRRN3
MEDAG
MEOX2
MIR3682
MIR3945HG
MMP10
MS4A15
MSX1
MT1F
MT1G
MT1M
MUC15

DCUN1D3
AREG
TIFA
CXCL1
HBB
GPR84
TMEM217
RALGDS
BBS10
DDIT3
EHHADH
PHLDA2
STK17A
DEDD2
PFKFB3
PHLDA1
HCG11
EREG
PANX1
PER2
NLRP3
BHLHE40
CHSY1
FCAR
IRX3
EFCAB7
PNP
PPP1R3C
ETS1
TOP1
PIM1
TP53INP2
BMP2
ST7-AS1
EID3
SLC25A37
HIVEP2
CLEC4D
SERPINB9
SPHK1
GADD45A
PLAUR
IL6
RHOH
MZB1
NABP1
NFKBIE
NFKBIZ
NIM1K
NPY1R
NR4A2
OMD
P2RY12
PI15
PMAIP1
PNP
POU2AF1
PPIF
PPP1R15A
PRSS21
PTGS2
PTX3
RASGRP1
RASSF10
RELB
RND1
RNF148
RRAD
RTKN2
S100A3
SELE
SERPINB2
SERPINE1
SERTAD1
SERTM1
SFN
SGPP2
SHISA2
SLC10A4
SLC16A6
SLC30A1
SLC6A14
SLC7A11
SLC7A5
SMCO3
SOCS2
SOX7
SPHK1

TNF
SGMS2
MAP3K14
ZFAND2A
FSTL3
RUBCNL
EMP1
IL1B
GJB2
HOXA5
BAZ1A
STC2
CYTIP
TNFRSF10D
LRRK2
LRG1
PIGA
BABAM2-AS1
CRY1
CCL20
TNFRSF12A
DISP1
SLC20A1
CCDC71L
MIR3682
LIF
SERPINB2
TREML2
FFAR2
STC1
XIRP1
SERPINB1
RND1
TNFAIP6
SLC7A5
RGS16
RND3
GPR34
BATF3
TNFSF9
FAM222A
DLGAP1-AS2
GIMAP2
TUBB2A
SPINK1
SPSB1
STC1
STC2
STEAP4
TAC1
TDO2
TENM2
TFPI2
TIMP4
TMEM178A
TMEM217
TNC
TNF
TNFAIP3
TNFAIP6
TNFRSF11B
TNFRSF12A
TNFRSF17
TNFSF9
TNIP3
TSLP
UGT2B4
UTS2
VCAM1
YOD1
ZFAND2A
ZNF165
ZNF331
ZNF404
ZNF750
ZNF880

G0S2
FPR2
CHORDC1
ADM
OSM
MYC
HAS2
EZH2
SOX7
B3GALT2
MIR3945HG
FAM13C
CX3CL1
BATF
CD274
SNAI1
SGPP2
BCL2A1
IL24
GPR183
ADGRG3
CSRNP1
C9orf152
IRX5
HSPH1
PLEK
CH25H
HRH1
PDE4B
RDH10
MSC
JUNB
IL32
FAM110C
PADI4
GZMB
CDA
LMNB1
PTX3
PLEKHG1
CRISP3
DUSP14
IL1RN
SH3RF3-AS1

CCL2
ANGPTL4
IL18R1
IL18RAP
CCL4
TSLP
RGL4
MEOX2
SAMD13
S100P
ADAMTS9
CXCR1
IFNG
SPIN4
LRRC32
RBMS3-AS3
DNAJA4
CMTM2
HMOX1
THBD
DACT1
VCAM1
BYSL
FPR1
RASGRP1
FIBIN
PANK1
CCL11
LIPG
GCLM
SLC19A2
SLC6A14
HBD
ADORA3
STEAP4
ZNF404
C2CD4A
HEY1
SFN
NABP1
CLC
TNFRSF10C
ICAM4
GNLY

ZNF750
ANKRD22
TMEM71
RIPOR2
C15orf48
GEM
ART4
SLC16A6
AQP9
SELE
PRF1
SHISA2
CITED1
IL1R2
CLDN1
TRIL
ADAMTS9-AS1
SLC7A11
FOSB
CNTN3
CXCR2
FCN1
NFE2
SELL
GFPT2
APOBEC3B
TNIP3
CXCL5
VNN1
MMP9
CCL7
CCDC178
TNFRSF17
FOS
TFPI2
VNN2
PI3
RARRES1
PF4
RUNDC3B
S100A12
RGS18
IL1RL1
FGFBP2

| |
|---|
| SLITRK6 |
| PPBP |
| TMEM100 |
| MGAM |
| PROK2 |
| MMP1 |
| SRGAP3-AS2 |
| SOSTDC1 |
| MMP8 |
| TAC1 |
| CEACAM8 |
| OLFM4 |
| ANKRD1 |
| SNTN |

**Table S2. Lung Transplantation-Related Genes Curated from GeneCards and OMIM Databases.**

| Only GeneCards | Only OMIM | GeneCards and OMIM |
|---|---|---|
| EGFR | FMO2 | EGFR |
| TERT | FASLG | TERT |
| TP53 | KCNK3 | TP53 |
| KRAS | SFTPB | KRAS |
| BRAF | CASP8 | BRAF |
| TSC2 | BMPR2 | TSC2 |
| ABCA3 | CPS1 | ABCA3 |
| NKX2-1 | FARSB | NKX2-1 |
| ERBB2 | DLEC1 | ERBB2 |
| CDKN2A | CCR2 | CDKN2A |
| SFTPC | DBR1 | SFTPC |
| ALK | TERC | ALK |
| DICER1 | PIK3CA | DICER1 |
| RB1 | EIF4G1 | RB1 |
| TGFB1 | ATP13A3 | TGFB1 |
| IL6 | SLC34A2 | IL6 |
| PIK3CA | IGFBP7 | PIK3CA |
| MALAT1 | NAF1 | MALAT1 |
| TNF | TERT | TNF |
| PTEN | LUCAT1 | PTEN |
| TSC1 | IRF1 | TSC1 |
| AKT1 | PRKN | AKT1 |
| COPA | EGFR | COPA |
| IL10 | CAV1 | IL10 |
| MARS1 | POT1 | MARS1 |

| | | |
|---|---|---|
| SFTPB | BRAF | SFTPB |
| H19 | SFTPC | H19 |
| MYC | SOX17 | MYC |
| SFTPA1 | MAP3K8 | SFTPA1 |
| ITGA3 | ERCC6 | ITGA3 |
| MET | SFTPA2 | MET |
| FAS | SFTPA1 | FAS |
| MIR21 | SFTPD | MIR21 |
| CTNNB1 | MUC5B | CTNNB1 |
| IFNG | SLC67A1 | IFNG |
| STK11 | FAM111B | STK11 |
| STAT3 | MEN1 | STAT3 |
| FASLG | MALAT1 | FASLG |
| FGFR1 | PPP2R1B | FGFR1 |
| CCND1 | KRAS | CCND1 |
| ATM | MARS1 | ATM |
| MUC5B | OAS1 | MUC5B |
| CASP8 | ZCCHC8 | CASP8 |
| HRAS | SMAD9 | HRAS |
| PVT1 | RB1 | PVT1 |
| NFE2L2 | NSMCE3 | NFE2L2 |
| ERCC6 | NOP10 | ERCC6 |
| CFTR | EIF2AK4 | CFTR |
| FGFR3 | CHRNA3 | FGFR3 |
| CSF3 | ABCA3 | CSF3 |
| KIT | PARN | KIT |
| NOTCH1 | FOXF1 | NOTCH1 |
| CXCL8 | RPA1 | CXCL8 |
| MAP2K1 | ERBB2 | MAP2K1 |
| SMAD4 | ITGA3 | SMAD4 |
| ABCB1 | MPO | ABCB1 |
| FGFR2 | TBX4 | FGFR2 |
| PRKN | FARSA | PRKN |
| ROS1 | CAPNS1 | ROS1 |
| SERPINA1 | SARS2 | SERPINA1 |
| LOC110806263 | CYP2A6 | LOC110806263 |
| RET | TGFB1 | RET |
| SFTPA2 | HCK | SFTPA2 |
| GAS5 | RTEL1 | GAS5 |
| MIR125A | HMOX1 | MIR125A |
| BRCA2 | CSF2RB | BRCA2 |
| PDGFRA | CSF2RA | PDGFRA |
| NF1 | | NF1 |
| MIR155 | | MIR155 |
| MLH1 | | MLH1 |

IL1B
HMOX1
VEGFA
FGF10
PPARG
MDM2
NEAT1
NRAS
PIK3CG
RARB
SFTPD
ACTA2
LUCAT1
RAF1
CD274
SMARCA4
CHEK2
MIR17
RTEL1
MIR126
CAV1
BRCA1
RASSF1
BCL2
DDR2
KEAP1
KMT2D
MIR146A
LINC02605
HLA-DRB1
MIR145
APC
MIR30A
ESR1
CTLA4
NRG1
IL4
TUG1
MIRLET7C
SFTA3
IL2RA
IGF1R
IL2
MBL2
MIR140

IL1B
HMOX1
VEGFA
FGF10
PPARG
MDM2
NEAT1
NRAS
PIK3CG
RARB
SFTPD
ACTA2
LUCAT1
RAF1
CD274
SMARCA4
CHEK2
MIR17
RTEL1
MIR126
CAV1
BRCA1
RASSF1
BCL2
DDR2
KEAP1
KMT2D
MIR146A
LINC02605
HLA-DRB1
MIR145
APC
MIR30A
ESR1
CTLA4
NRG1
IL4
TUG1
MIRLET7C
SFTA3
IL2RA
IGF1R
IL2
MBL2
MIR140

| | |
|---|---|
| MUC1 | MUC1 |
| IRF1 | IRF1 |
| TLR4 | TLR4 |
| FOXF1 | FOXF1 |
| DLEC1 | DLEC1 |
| TERC | TERC |
| ERBB4 | ERBB4 |
| HLA-B | HLA-B |
| ELN | ELN |
| GSTM1 | GSTM1 |
| CYP2A6 | CYP2A6 |
| ACE | ACE |
| SNHG1 | SNHG1 |
| MIR143 | MIR143 |
| NTRK1 | NTRK1 |
| MIR29A | MIR29A |
| CSF2 | CSF2 |
| MIR221 | MIR221 |
| MIR146B | MIR146B |
| BAP1 | BAP1 |
| MIR200B | MIR200B |
| MIR98 | MIR98 |
| FLNA | FLNA |
| MIRLET7B | MIRLET7B |
| MMP9 | MMP9 |
| ERBB3 | ERBB3 |
| CCL2 | CCL2 |
| AXL | AXL |
| CDKN2B-AS1 | CDKN2B-AS1 |
| SRC | SRC |
| MIRLET7D | MIRLET7D |
| ALB | ALB |
| MIR205 | MIR205 |
| NTRK3 | NTRK3 |
| FBXW7 | FBXW7 |
| MIR31 | MIR31 |
| CYP1A1 | CYP1A1 |
| ICAM1 | ICAM1 |
| NTRK2 | NTRK2 |
| MMP2 | MMP2 |
| PTGS2 | PTGS2 |
| SOX2 | SOX2 |
| MIR199A1 | MIR199A1 |
| CCR2 | CCR2 |
| MIR214 | MIR214 |

| | |
|---|---|
| HGF | HGF |
| DSP | DSP |
| ATR | ATR |
| MIR182 | MIR182 |
| LINC00511 | LINC00511 |
| PPP2R1B | PPP2R1B |
| TP73 | TP73 |
| CYP3A5 | CYP3A5 |
| PKHD1 | PKHD1 |
| SLC19A1 | SLC19A1 |
| CXCR4 | CXCR4 |
| MTOR | MTOR |
| FLCN | FLCN |
| MIR150 | MIR150 |
| MIR22 | MIR22 |
| CFH | CFH |
| CRP | CRP |
| MIR93 | MIR93 |
| HIF1A | HIF1A |
| CDH1 | CDH1 |
| UCA1 | UCA1 |
| CCAT1 | CCAT1 |
| MMP1 | MMP1 |
| PCAT1 | PCAT1 |
| MIR210 | MIR210 |
| MIR34A | MIR34A |
| HLA-A | HLA-A |
| MIR338 | MIR338 |
| MIR20A | MIR20A |
| GSTP1 | GSTP1 |
| SOD2 | SOD2 |
| IDH1 | IDH1 |
| HOTAIRM1 | HOTAIRM1 |
| ENG | ENG |
| MIR195 | MIR195 |
| MIR141 | MIR141 |
| EDN1 | EDN1 |
| MIR200C | MIR200C |
| GATA3 | GATA3 |
| MIR18A | MIR18A |
| CHEK1 | CHEK1 |
| ANXA2 | ANXA2 |
| MIR133B | MIR133B |
| NFKB1 | NFKB1 |
| MIR335 | MIR335 |

| | |
|---|---|
| SNHG16 | SNHG16 |
| MTHFR | MTHFR |
| MIR130A | MIR130A |
| MIR148A | MIR148A |
| CASP3 | CASP3 |
| MIR19A | MIR19A |
| ELANE | ELANE |
| MIR183 | MIR183 |
| SCGB1A1 | SCGB1A1 |
| MIR216A | MIR216A |
| MUC16 | MUC16 |
| KDR | KDR |
| IDH2 | IDH2 |
| MIR99A | MIR99A |
| CDKN1A | CDKN1A |
| SMAD3 | SMAD3 |
| IL13 | IL13 |
| GNAS | GNAS |
| BDNF-AS | BDNF-AS |
| FOXO1 | FOXO1 |
| MIR200A | MIR200A |
| PALB2 | PALB2 |
| OIP5-AS1 | OIP5-AS1 |
| TGFBR2 | TGFBR2 |
| MIR101-1 | MIR101-1 |
| MIR34C | MIR34C |
| DANCR | DANCR |
| MIR139 | MIR139 |
| EGF | EGF |
| XIST | XIST |
| ADAMTS9-AS2 | ADAMTS9-AS2 |
| MPO | MPO |
| TMPO-AS1 | TMPO-AS1 |
| FGF7 | FGF7 |
| MIR451A | MIR451A |
| MIR26A1 | MIR26A1 |
| BMPR2 | BMPR2 |
| TP63 | TP63 |
| IGF1 | IGF1 |
| BAX | BAX |
| TBX4 | TBX4 |
| FGF2 | FGF2 |
| ERCC2 | ERCC2 |
| FENDRR | FENDRR |
| NSD2 | NSD2 |

| | |
|---|---|
| MIR30E | MIR30E |
| NORAD | NORAD |
| POLD1 | POLD1 |
| HOTAIR | HOTAIR |
| CDKN2B | CDKN2B |
| IL1A | IL1A |
| GFOD3P | GFOD3P |
| MIR191 | MIR191 |
| PARN | PARN |
| MIR324 | MIR324 |
| SPP1 | SPP1 |
| MIR29C | MIR29C |
| LINC01089 | LINC01089 |
| WT1 | WT1 |
| ERCC1 | ERCC1 |
| SMPD1 | SMPD1 |
| MIR486-1 | MIR486-1 |
| FOXP3 | FOXP3 |
| MIR204 | MIR204 |
| MAPK1 | MAPK1 |
| CCN2 | CCN2 |
| IL17A | IL17A |
| CYP3A4 | CYP3A4 |
| POLE | POLE |
| MIR124-1 | MIR124-1 |
| MIR212 | MIR212 |
| U2AF1 | U2AF1 |
| PDCD1 | PDCD1 |
| FN1 | FN1 |
| MCM3AP-AS1 | MCM3AP-AS1 |
| CASC11 | CASC11 |
| MIR192 | MIR192 |
| SERPINE1 | SERPINE1 |
| FARSB | FARSB |
| FAM13A | FAM13A |
| NKILA | NKILA |
| MIR4435-2HG | MIR4435-2HG |
| MIRLET7G | MIRLET7G |
| AFAP1-AS1 | AFAP1-AS1 |
| SHH | SHH |
| HLA-G | HLA-G |
| MIR106A | MIR106A |
| ENO2 | ENO2 |
| MIF | MIF |
| MIR203A | MIR203A |

| | |
|---|---|
| MIR497 | MIR497 |
| IL1RN | IL1RN |
| MIR224 | MIR224 |
| GSTT1 | GSTT1 |
| GATA2 | GATA2 |
| CD44 | CD44 |
| CDK4 | CDK4 |
| CDKN1B | CDKN1B |
| SLC34A2 | SLC34A2 |
| PGR-AS1 | PGR-AS1 |
| NNT-AS1 | NNT-AS1 |
| FBXL19-AS1 | FBXL19-AS1 |
| MNX1-AS1 | MNX1-AS1 |
| MXRA5 | MXRA5 |
| THBD | THBD |
| RMRP | RMRP |
| FAM83A-AS1 | FAM83A-AS1 |
| XRCC1 | XRCC1 |
| HLA-DQB1 | HLA-DQB1 |
| STAT1 | STAT1 |
| AKT3 | AKT3 |
| MIR197 | MIR197 |
| IL7R | IL7R |
| LINC00520 | LINC00520 |
| GSTM3 | GSTM3 |
| CCL5 | CCL5 |
| MIR27B | MIR27B |
| MIR33A | MIR33A |
| TIMP1 | TIMP1 |
| HLA-C | HLA-C |
| F2 | F2 |
| CD40LG | CD40LG |
| MIR425 | MIR425 |
| DYNC2H1 | DYNC2H1 |
| MAP3K8 | MAP3K8 |
| MIR32 | MIR32 |
| RUNX1T1 | RUNX1T1 |
| LINC00673 | LINC00673 |
| FLT1 | FLT1 |
| AGAP2-AS1 | AGAP2-AS1 |
| TTR | TTR |
| NOD2 | NOD2 |
| EPHX1 | EPHX1 |
| KITLG | KITLG |
| RTEL1-TNFRSF6B | RTEL1-TNFRSF6B |

| | |
|---|---|
| CASC9 | CASC9 |
| JUN | JUN |
| MIR199B | MIR199B |
| BMP4 | BMP4 |
| ENO1 | ENO1 |
| MIR377 | MIR377 |
| MIRLET7E | MIRLET7E |
| IL18 | IL18 |
| CCAT2 | CCAT2 |
| DKC1 | DKC1 |
| MIR339 | MIR339 |
| HULC | HULC |
| BMP6 | BMP6 |
| AGTR1 | AGTR1 |
| TTN-AS1 | TTN-AS1 |
| CERNA3 | CERNA3 |
| KRT19 | KRT19 |
| EPO | EPO |
| VHL | VHL |
| LIG4 | LIG4 |
| IL3 | IL3 |
| PIK3R1 | PIK3R1 |
| MIR219A1 | MIR219A1 |
| LINC00205 | LINC00205 |
| DGCR5 | DGCR5 |
| NOS2 | NOS2 |
| NLRP3 | NLRP3 |
| ABCC1 | ABCC1 |
| MGMT | MGMT |
| MIRLET7A2 | MIRLET7A2 |
| FOXD3-AS1 | FOXD3-AS1 |
| TINF2 | TINF2 |
| MIR24-2 | MIR24-2 |
| GRP | GRP |
| HAGLROS | HAGLROS |
| TNFRSF1A | TNFRSF1A |
| MIR181C | MIR181C |
| MIR128-2 | MIR128-2 |
| LINC00662 | LINC00662 |
| BIRC5 | BIRC5 |
| INS | INS |
| BMP2 | BMP2 |
| LINC00857 | LINC00857 |
| SNHG4 | SNHG4 |
| LINC00261 | LINC00261 |

| | |
|---|---|
| CBS | CBS |
| JAK3 | JAK3 |
| ACVRL1 | ACVRL1 |
| MIR511 | MIR511 |
| EZH2 | EZH2 |
| CD4 | CD4 |
| VDR | VDR |
| XIAP | XIAP |
| IFNA1 | IFNA1 |
| MYCN | MYCN |
| SNHG11 | SNHG11 |
| OGFRP1 | OGFRP1 |
| SOX9 | SOX9 |
| FHIT | FHIT |
| SMO | SMO |
| MIR125B2 | MIR125B2 |
| MAPK8 | MAPK8 |
| MIR222 | MIR222 |
| CASC15 | CASC15 |
| ADA | ADA |
| AGT | AGT |
| MSH2 | MSH2 |
| HLA-DPB1 | HLA-DPB1 |
| CFI | CFI |
| GATA6-AS1 | GATA6-AS1 |
| CXCL12 | CXCL12 |
| COL4A3 | COL4A3 |
| TLR2 | TLR2 |
| S100B | S100B |
| KRT7 | KRT7 |
| THADA | THADA |
| MIRLET7F1 | MIRLET7F1 |
| SBF2-AS1 | SBF2-AS1 |
| SOX21-AS1 | SOX21-AS1 |
| LCIIAR | LCIIAR |
| MIR15A | MIR15A |
| GNAQ | GNAQ |
| IL5 | IL5 |
| SLC7A7 | SLC7A7 |
| MEG3 | MEG3 |
| CST3 | CST3 |
| ZFPM2-AS1 | ZFPM2-AS1 |
| ITGB1 | ITGB1 |
| TYMS | TYMS |
| EPIC1 | EPIC1 |

| | |
|---|---|
| ARID1A | ARID1A |
| SOD1 | SOD1 |
| CD40 | CD40 |
| BCL2L1 | BCL2L1 |
| ABCC2 | ABCC2 |
| EPCAM | EPCAM |
| CCR5 | CCR5 |
| NBN | NBN |
| MIRLET7A3 | MIRLET7A3 |
| SNHG20 | SNHG20 |
| LINC00336 | LINC00336 |
| PDGFRB | PDGFRB |
| IL1R1 | IL1R1 |
| NFKBIA | NFKBIA |
| HMGB1 | HMGB1 |
| PRNCR1 | PRNCR1 |
| COL4A1 | COL4A1 |
| VIM | VIM |
| MIR31HG | MIR31HG |
| MYCL | MYCL |
| DLEU2 | DLEU2 |
| PTK2 | PTK2 |
| JAK2 | JAK2 |
| LMNA | LMNA |
| HOXB-AS3 | HOXB-AS3 |
| MIR345 | MIR345 |
| CXCR3 | CXCR3 |
| CEACAM3 | CEACAM3 |
| MUC5AC | MUC5AC |
| MIR29B2 | MIR29B2 |
| MVP | MVP |
| CD8A | CD8A |
| HFE | HFE |
| SFTA1P | SFTA1P |
| MIR429 | MIR429 |
| VEGFC | VEGFC |
| PTCH1 | PTCH1 |
| DPP9 | DPP9 |
| MIR124-3 | MIR124-3 |
| MIR218-2 | MIR218-2 |
| F3 | F3 |
| FER1L4 | FER1L4 |
| VCAM1 | VCAM1 |
| MIR223 | MIR223 |
| NOTCH3 | NOTCH3 |

| | |
|---|---|
| SMAD2 | SMAD2 |
| PECAM1 | PECAM1 |
| MIR95 | MIR95 |
| PCNA | PCNA |
| PLAU | PLAU |
| LINC00665 | LINC00665 |
| TGFBR1 | TGFBR1 |
| EIF2AK4 | EIF2AK4 |
| KCNQ1OT1 | KCNQ1OT1 |
| IL2RB | IL2RB |
| GATA6 | GATA6 |
| TDRG1 | TDRG1 |
| NQO1 | NQO1 |
| PSMA3-AS1 | PSMA3-AS1 |
| BIRC3 | BIRC3 |
| MIR96 | MIR96 |
| CXCL10 | CXCL10 |
| GZMB | GZMB |
| PARP1 | PARP1 |
| LINC00839 | LINC00839 |
| GNA11 | GNA11 |
| PTPRC | PTPRC |
| IL15 | IL15 |
| PPP2R1A | PPP2R1A |
| MIR185 | MIR185 |
| TGFA | TGFA |
| CALCA | CALCA |
| PLG | PLG |
| IL7 | IL7 |
| TGFB2 | TGFB2 |
| NBAT1 | NBAT1 |
| NCAPG | NCAPG |
| PTPRG | PTPRG |
| TNFSF10 | TNFSF10 |
| PRF1 | PRF1 |
| TTN | TTN |
| LINC01194 | LINC01194 |
| B2M | B2M |
| PIK3CD | PIK3CD |
| CASC2 | CASC2 |
| CCL11 | CCL11 |
| MIR30D | MIR30D |
| MMP7 | MMP7 |
| MAPK14 | MAPK14 |
| MKI67 | MKI67 |

| | |
|---|---|
| NOS3 | NOS3 |
| AKT2 | AKT2 |
| ZFAS1 | ZFAS1 |
| CEACAM5 | CEACAM5 |
| CHGA | CHGA |
| FLT3 | FLT3 |
| MIR142 | MIR142 |
| HPS1 | HPS1 |
| CASP9 | CASP9 |
| PDGFB | PDGFB |
| STN1 | STN1 |
| PKD1 | PKD1 |
| TRG-AS1 | TRG-AS1 |
| ATP11A | ATP11A |
| CSF2RA | CSF2RA |
| MIR15B | MIR15B |
| CYTOR | CYTOR |
| CERS6-AS1 | CERS6-AS1 |
| ADAM12 | ADAM12 |
| SOX4 | SOX4 |
| MIAT | MIAT |
| CCL3 | CCL3 |
| ZNF674-AS1 | ZNF674-AS1 |
| SNHG3 | SNHG3 |
| CREBBP | CREBBP |
| LAMC2 | LAMC2 |
| SNHG17 | SNHG17 |
| CDK6 | CDK6 |
| ABCG2 | ABCG2 |
| ZMYND10 | ZMYND10 |
| TUSC7 | TUSC7 |
| SLC11A1 | SLC11A1 |
| CADM1 | CADM1 |
| PTPN11 | PTPN11 |
| HOXA11-AS | HOXA11-AS |
| CD36 | CD36 |
| FCGR2A | FCGR2A |
| IGFBP3 | IGFBP3 |
| VWF | VWF |
| EDNRA | EDNRA |
| HIF1A-AS2 | HIF1A-AS2 |
| CBL | CBL |
| OGG1 | OGG1 |
| NPPA | NPPA |
| IGF2 | IGF2 |

| | |
|---|---|
| HOTTIP | HOTTIP |
| MIR198 | MIR198 |
| NPM1 | NPM1 |
| HAGLR | HAGLR |
| ARAP1-AS1 | ARAP1-AS1 |
| NOTCH2 | NOTCH2 |
| MT1JP | MT1JP |
| MAPK3 | MAPK3 |
| RUNX1-IT1 | RUNX1-IT1 |
| BCYRN1 | BCYRN1 |
| NSMCE3 | NSMCE3 |
| HPS4 | HPS4 |
| WFDC21P | WFDC21P |
| TLR9 | TLR9 |
| HLA-DQA1 | HLA-DQA1 |
| HCP5 | HCP5 |
| AGER | AGER |
| SCGB3A2 | SCGB3A2 |
| TLR3 | TLR3 |
| FEZF1-AS1 | FEZF1-AS1 |
| SNHG12 | SNHG12 |
| GRM8 | GRM8 |
| CYP2E1 | CYP2E1 |
| FLT4 | FLT4 |
| ACTC1 | ACTC1 |
| CSF2RB | CSF2RB |
| LINC00467 | LINC00467 |
| SCNN1A | SCNN1A |
| TFRC | TFRC |
| ADIPOQ | ADIPOQ |
| CEACAM6 | CEACAM6 |
| XPC | XPC |
| KRT18 | KRT18 |
| HCG11 | HCG11 |
| INPP5E | INPP5E |
| DMTF1 | DMTF1 |
| TOP1 | TOP1 |
| NRAD1 | NRAD1 |
| LEP | LEP |
| NCAM1 | NCAM1 |
| SP1 | SP1 |
| MIR499A | MIR499A |
| SOD2-OT1 | SOD2-OT1 |
| MMP14 | MMP14 |
| NPPB | NPPB |

| | |
|---|---|
| TNFRSF1B | TNFRSF1B |
| CDR1-AS | CDR1-AS |
| REN | REN |
| RELA | RELA |
| COL4A5 | COL4A5 |
| MIR193A | MIR193A |
| MIR100 | MIR100 |
| ELMOD2 | ELMOD2 |
| CSF1 | CSF1 |
| SLC17A5 | SLC17A5 |
| WT1-AS | WT1-AS |
| SNHG7 | SNHG7 |
| THBS1 | THBS1 |
| COL4A2 | COL4A2 |
| GJA1 | GJA1 |
| LINC01116 | LINC01116 |
| MMP3 | MMP3 |
| AHR | AHR |
| TIMP2 | TIMP2 |
| MIR34B | MIR34B |
| CD226 | CD226 |
| SYP | SYP |
| HSP90AA1 | HSP90AA1 |
| MPL | MPL |
| SETD2 | SETD2 |
| PRTN3 | PRTN3 |
| GPT | GPT |
| DNMT3A | DNMT3A |
| SOX2-OT | SOX2-OT |
| LINC00485 | LINC00485 |
| COL3A1 | COL3A1 |
| FOXM1 | FOXM1 |
| SNAI1 | SNAI1 |
| RHOA | RHOA |
| IL11 | IL11 |
| CDK2 | CDK2 |
| DES | DES |
| SIRT1 | SIRT1 |
| GSN | GSN |
| LOXL1-AS1 | LOXL1-AS1 |
| DNAH5 | DNAH5 |
| MEN1 | MEN1 |
| IL6R | IL6R |
| DOCK8 | DOCK8 |
| DNMT1 | DNMT1 |

| | |
|---|---|
| EP300 | EP300 |
| XRCC3 | XRCC3 |
| CPS1 | CPS1 |
| SMAD7 | SMAD7 |
| CYP1B1 | CYP1B1 |
| PANDAR | PANDAR |
| BPIFA1 | BPIFA1 |
| MSH6 | MSH6 |
| KLF2 | KLF2 |
| SLC2A1 | SLC2A1 |
| E2F1 | E2F1 |
| SERPINC1 | SERPINC1 |
| METTL3 | METTL3 |
| CD28 | CD28 |
| APOA1 | APOA1 |
| GLA | GLA |
| LCN2 | LCN2 |
| LOX | LOX |
| CDKN1C | CDKN1C |
| CCR7 | CCR7 |
| PDPN | PDPN |
| ANGPT1 | ANGPT1 |
| MCL1 | MCL1 |
| NAPSA | NAPSA |
| IKBKG | IKBKG |
| INVS | INVS |
| AGO2 | AGO2 |
| CHUK | CHUK |
| MIR144 | MIR144 |
| GSK3B | GSK3B |
| CYBB | CYBB |
| CDK12 | CDK12 |
| CD34 | CD34 |
| ABL1 | ABL1 |
| HNF1A-AS1 | HNF1A-AS1 |
| LINC01123 | LINC01123 |
| MIR27A | MIR27A |
| ALOX5 | ALOX5 |
| COL1A1 | COL1A1 |
| APEX1 | APEX1 |
| TOP2A | TOP2A |
| MMP12 | MMP12 |
| DNMT3B | DNMT3B |
| IKBKB | IKBKB |
| CLEC7A | CLEC7A |

| | |
|---|---|
| SST | SST |
| CD79A | CD79A |
| FOXJ1 | FOXJ1 |
| DMBT1 | DMBT1 |
| FANCA | FANCA |
| MYLK | MYLK |
| IGFBP2 | IGFBP2 |
| CASP10 | CASP10 |
| TF | TF |
| RAG1 | RAG1 |
| HBB | HBB |
| RBM10 | RBM10 |
| LAMA5 | LAMA5 |
| GLI1 | GLI1 |
| TNFRSF10B | TNFRSF10B |
| FARSA | FARSA |
| RAC1 | RAC1 |
| TKT | TKT |
| FADD | FADD |
| GPC3 | GPC3 |
| ARG1 | ARG1 |
| LTA | LTA |
| CD46 | CD46 |
| MIR375 | MIR375 |
| TP53TG1 | TP53TG1 |
| SCNN1B | SCNN1B |
| NUTM2A-AS1 | NUTM2A-AS1 |
| THPO | THPO |
| PLAUR | PLAUR |
| KRT5 | KRT5 |
| ESR2 | ESR2 |
| NME1 | NME1 |
| CYCS | CYCS |
| F5 | F5 |
| SOD3 | SOD3 |
| ANGPT2 | ANGPT2 |
| SPARC | SPARC |
| MIR23A | MIR23A |
| ASCL1 | ASCL1 |
| IREB2 | IREB2 |
| HSPB1 | HSPB1 |
| ADRB2 | ADRB2 |
| GBA1 | GBA1 |
| SLPI | SLPI |
| RXRA | RXRA |

| | |
|---|---|
| JAG1 | JAG1 |
| FOXA2 | FOXA2 |
| ACTB | ACTB |
| TYMP | TYMP |
| ALDOA | ALDOA |
| TINCR | TINCR |
| AGTR2 | AGTR2 |
| MYH7 | MYH7 |
| WNT4 | WNT4 |
| KRT20 | KRT20 |
| PRKDC | PRKDC |
| NAT2 | NAT2 |
| ERCC5 | ERCC5 |
| CXCL2 | CXCL2 |
| LAMB3 | LAMB3 |
| FGFR4 | FGFR4 |
| ABCB11 | ABCB11 |
| BCL6 | BCL6 |
| LOC110806306 | LOC110806306 |
| SNHG14 | SNHG14 |
| MIR372 | MIR372 |
| LAMA3 | LAMA3 |
| IL2RG | IL2RG |
| FBN1 | FBN1 |
| CAT | CAT |
| CCDC40 | CCDC40 |
| COL4A4 | COL4A4 |
| ERCC4 | ERCC4 |
| SOCS1 | SOCS1 |
| FOS | FOS |
| GC | GC |
| RARA | RARA |
| LINC00473 | LINC00473 |
| KRT8 | KRT8 |
| LTBP4 | LTBP4 |
| PDGFA | PDGFA |
| ACE2 | ACE2 |
| OFD1 | OFD1 |
| ITGAM | ITGAM |
| ITGB3 | ITGB3 |
| POT1 | POT1 |
| MIR30B | MIR30B |
| STING1 | STING1 |
| CREB1 | CREB1 |
| HSPA4 | HSPA4 |

| | |
|---|---|
| SNAI2 | SNAI2 |
| NAGLU | NAGLU |
| MUSK | MUSK |
| MUTYH | MUTYH |
| CD14 | CD14 |
| PAX2 | PAX2 |
| PTHLH | PTHLH |
| DUXAP8 | DUXAP8 |
| TUBB3 | TUBB3 |
| PLAT | PLAT |
| NRP1 | NRP1 |
| FBLN5 | FBLN5 |
| IL4R | IL4R |
| TNFRSF11B | TNFRSF11B |
| BAK1 | BAK1 |
| ACTA2-AS1 | ACTA2-AS1 |
| RPGR | RPGR |
| RRM1 | RRM1 |
| CEBPA | CEBPA |
| HAVCR2 | HAVCR2 |
| SOS1 | SOS1 |
| NCK1-DT | NCK1-DT |
| POSTN | POSTN |
| MILIP | MILIP |
| DPYD | DPYD |
| CD81 | CD81 |
| MAGI2-AS3 | MAGI2-AS3 |
| LINC-PINT | LINC-PINT |
| UGT1A1 | UGT1A1 |
| CCL4 | CCL4 |
| TGFB3 | TGFB3 |
| LINC00958 | LINC00958 |
| CX3CR1 | CX3CR1 |
| HLA-DPA1 | HLA-DPA1 |
| GPRC5A | GPRC5A |
| DLEU1 | DLEU1 |
| CTSB | CTSB |
| LPL | LPL |
| MYD88 | MYD88 |
| MIR137 | MIR137 |
| LINC00472 | LINC00472 |
| LIPA | LIPA |
| PMS2 | PMS2 |
| SNHG6 | SNHG6 |
| CYP1A2 | CYP1A2 |

| | |
|---|---|
| TPX2 | TPX2 |
| ITGA2 | ITGA2 |
| IL12B | IL12B |
| MIR107 | MIR107 |
| TBX5 | TBX5 |
| FCGR3A | FCGR3A |
| CHI3L1 | CHI3L1 |
| NPHS2 | NPHS2 |
| GUSB | GUSB |
| PRKCA | PRKCA |
| EPAS1 | EPAS1 |
| IFNGR1 | IFNGR1 |
| XDH | XDH |
| APOE | APOE |
| CRNDE | CRNDE |
| MICA | MICA |
| FTX | FTX |
| C3 | C3 |
| RUNX1 | RUNX1 |
| CYP2D6 | CYP2D6 |
| ODAD1 | ODAD1 |
| STAT6 | STAT6 |
| BMP7 | BMP7 |
| TNFRSF8 | TNFRSF8 |
| YAP1 | YAP1 |
| SMAD9 | SMAD9 |
| LGALS3 | LGALS3 |
| NCF2 | NCF2 |
| ERCC3 | ERCC3 |
| AREG | AREG |
| CYP2C19 | CYP2C19 |
| HDAC1 | HDAC1 |
| DNAH8 | DNAH8 |
| NEU1 | NEU1 |
| ZEB1 | ZEB1 |
| HAND2-AS1 | HAND2-AS1 |
| RAD51 | RAD51 |
| MIR122 | MIR122 |
| PTK2B | PTK2B |
| TXN | TXN |
| ACACA | ACACA |
| LINC00342 | LINC00342 |
| HMGA2 | HMGA2 |
| BPTF | BPTF |
| ANXA5 | ANXA5 |

| | |
|---|---|
| CYBA | CYBA |
| DUSP1 | DUSP1 |
| JUP | JUP |
| LIG1 | LIG1 |
| RPS6KB1 | RPS6KB1 |
| KIR3DL1 | KIR3DL1 |
| NR3C1 | NR3C1 |
| CDH2 | CDH2 |
| CDKN3 | CDKN3 |
| TIMP3 | TIMP3 |
| THY1 | THY1 |
| SELP | SELP |
| LINC01234 | LINC01234 |
| DAPK1 | DAPK1 |
| ITGAV | ITGAV |
| KLF6 | KLF6 |
| IDO1 | IDO1 |
| MAP2K2 | MAP2K2 |
| SPAG1 | SPAG1 |
| WRN | WRN |
| HOPX | HOPX |
| CDC42 | CDC42 |
| AURKA | AURKA |
| IDUA | IDUA |
| WAS | WAS |
| ABCC3 | ABCC3 |
| DNAH11 | DNAH11 |
| CXCR2 | CXCR2 |
| CD27 | CD27 |
| PTX3 | PTX3 |
| PIK3CB | PIK3CB |
| BSG | BSG |
| TEK | TEK |
| PSMG3-AS1 | PSMG3-AS1 |
| BAG3 | BAG3 |
| APOL1 | APOL1 |
| HNF1B | HNF1B |
| PLK1 | PLK1 |
| PAX5 | PAX5 |
| BANCR | BANCR |
| WNT5A | WNT5A |
| VEGFD | VEGFD |
| DLX6-AS1 | DLX6-AS1 |
| EPHB4 | EPHB4 |
| NAF1 | NAF1 |

| | |
|---|---|
| PRKCI | PRKCI |
| CCNE1 | CCNE1 |
| BID | BID |
| GLI2 | GLI2 |
| HDAC9 | HDAC9 |
| SNHG5 | SNHG5 |
| MIR148B | MIR148B |
| CXCL9 | CXCL9 |
| CDK1 | CDK1 |
| GLI3 | GLI3 |
| LINC00968 | LINC00968 |
| PITPNA-AS1 | PITPNA-AS1 |
| BCR | BCR |
| TET2 | TET2 |
| MUC4 | MUC4 |
| MIR155HG | MIR155HG |
| MIR132 | MIR132 |
| ATRX | ATRX |
| CCDC39 | CCDC39 |
| GAPDH | GAPDH |
| DNAAF3 | DNAAF3 |
| FGA | FGA |
| DNAAF1 | DNAAF1 |
| DDR1 | DDR1 |
| DHFR | DHFR |
| SNHG15 | SNHG15 |
| DNAAF4 | DNAAF4 |
| GGT1 | GGT1 |
| MIR211 | MIR211 |
| CFLAR | CFLAR |
| LAMA2 | LAMA2 |
| TRAF6 | TRAF6 |
| CCL17 | CCL17 |
| CCNO | CCNO |
| FGD5-AS1 | FGD5-AS1 |
| CD80 | CD80 |
| MSLN | MSLN |
| MAX | MAX |
| TCF7L2 | TCF7L2 |
| COMT | COMT |
| MIR181A1 | MIR181A1 |
| GDF15 | GDF15 |
| DLK1 | DLK1 |
| EPB41L3 | EPB41L3 |
| ENTREP2 | ENTREP2 |

| | |
|---|---|
| MIR106B | MIR106B |
| GREM1 | GREM1 |
| WNT7B | WNT7B |
| NODAL | NODAL |
| ABCC6 | ABCC6 |
| FOXO3 | FOXO3 |
| CTSD | CTSD |
| MIR7-3HG | MIR7-3HG |
| GATA4 | GATA4 |
| COL1A2 | COL1A2 |
| BIRC7 | BIRC7 |
| MIR99AHG | MIR99AHG |
| CDH13 | CDH13 |
| BBOX1-AS1 | BBOX1-AS1 |
| HNRNPA2B1 | HNRNPA2B1 |
| SELL | SELL |
| PGR | PGR |
| DNAI1 | DNAI1 |
| HMGCR | HMGCR |
| BLM | BLM |
| RBM6 | RBM6 |
| MIR16-1 | MIR16-1 |
| MIR25 | MIR25 |
| CD24 | CD24 |
| PKM | PKM |
| MMP13 | MMP13 |
| BUB1B | BUB1B |
| NPC2 | NPC2 |
| ACTN4 | ACTN4 |
| NBR2 | NBR2 |
| TAP1 | TAP1 |
| TRAF3 | TRAF3 |
| RYR1 | RYR1 |
| ILK | ILK |
| ANPEP | ANPEP |
| MBD4 | MBD4 |
| IL1RL1 | IL1RL1 |
| CCR3 | CCR3 |
| SELE | SELE |
| CD55 | CD55 |
| CCNA2 | CCNA2 |
| ADAM17 | ADAM17 |
| KLRK1 | KLRK1 |
| DHCR7 | DHCR7 |
| MIR215 | MIR215 |

| | |
|---|---|
| CCND3 | CCND3 |
| SLC26A4 | SLC26A4 |
| STXBP2 | STXBP2 |
| MIR9-2HG | MIR9-2HG |
| IL12RB1 | IL12RB1 |
| PART1 | PART1 |
| IL33 | IL33 |
| ALDH2 | ALDH2 |
| PON1 | PON1 |
| F8 | F8 |
| ABCC4 | ABCC4 |
| DDB2 | DDB2 |
| AXIN2 | AXIN2 |
| PCBP1-AS1 | PCBP1-AS1 |
| ITGB2 | ITGB2 |
| MTR | MTR |
| LINC00668 | LINC00668 |
| CD163 | CD163 |
| RAB27A | RAB27A |
| AGXT | AGXT |
| BRIP1 | BRIP1 |
| SOCS3 | SOCS3 |
| RSPH1 | RSPH1 |
| CDH5 | CDH5 |
| ICOS | ICOS |
| MIR10B | MIR10B |
| EGR1 | EGR1 |
| CHAT | CHAT |
| CCNB1 | CCNB1 |
| PCAT6 | PCAT6 |
| TRPC6 | TRPC6 |
| UNC13D | UNC13D |
| S100A4 | S100A4 |
| ADM | ADM |
| MIR152 | MIR152 |
| MIR423 | MIR423 |
| DNAAF5 | DNAAF5 |
| NPHS1 | NPHS1 |
| IL16 | IL16 |
| RSPH9 | RSPH9 |
| TWIST1 | TWIST1 |
| ANXA1 | ANXA1 |
| HSPA5 | HSPA5 |
| MIR127 | MIR127 |
| CALR | CALR |

| | |
|---|---|
| ODAD2 | ODAD2 |
| MIR342 | MIR342 |
| IL22 | IL22 |
| KCNK3 | KCNK3 |
| ITCH | ITCH |
| TCF21 | TCF21 |
| HSPA8 | HSPA8 |
| HPGD | HPGD |
| CDKN2C | CDKN2C |
| TNNT2 | TNNT2 |
| MMP8 | MMP8 |
| HLA-E | HLA-E |
| HOXA-AS2 | HOXA-AS2 |
| UFC1 | UFC1 |
| MST1R | MST1R |
| CASP1 | CASP1 |
| BCL10 | BCL10 |
| DKK1 | DKK1 |
| DPP4 | DPP4 |
| CYP24A1 | CYP24A1 |
| ID1 | ID1 |
| CTC1 | CTC1 |
| LGALS1 | LGALS1 |
| CEBPB | CEBPB |
| PPP1R13L | PPP1R13L |
| CABIN1 | CABIN1 |
| LBR | LBR |
| CCR1 | CCR1 |
| RACK1 | RACK1 |
| MIR125B1 | MIR125B1 |
| TLR7 | TLR7 |
| HSPA1A | HSPA1A |
| CYP17A1 | CYP17A1 |
| MIR373 | MIR373 |
| SF3B2 | SF3B2 |
| BMI1 | BMI1 |
| PCAT19 | PCAT19 |
| LEF1-AS1 | LEF1-AS1 |
| PDPK1 | PDPK1 |
| NSD1 | NSD1 |
| CP | CP |
| MANCR | MANCR |
| PROM1 | PROM1 |
| ALDH1A1 | ALDH1A1 |
| CASR | CASR |

| | |
|---|---|
| ATP7B | ATP7B |
| MIR374A | MIR374A |
| HYDIN | HYDIN |
| ITGA5 | ITGA5 |
| KTN1-AS1 | KTN1-AS1 |
| TNNI3 | TNNI3 |
| DDX11-AS1 | DDX11-AS1 |
| FAS-AS1 | FAS-AS1 |
| IRS1 | IRS1 |
| BGLAP | BGLAP |
| NR2F1-AS1 | NR2F1-AS1 |
| HLA-DQB1-AS1 | HLA-DQB1-AS1 |
| MIR151A | MIR151A |
| FGF1 | FGF1 |
| SLC25A13 | SLC25A13 |
| WRAP53 | WRAP53 |
| BTK | BTK |
| MIR10A | MIR10A |
| BIRC2 | BIRC2 |
| CXCR1 | CXCR1 |
| POLG | POLG |
| MIR23B | MIR23B |
| ETS1 | ETS1 |
| FOXA1 | FOXA1 |
| RUNX3 | RUNX3 |
| IFI27 | IFI27 |
| IGFBP6 | IGFBP6 |
| RAD50 | RAD50 |
| CC2D2A | CC2D2A |
| ZEB1-AS1 | ZEB1-AS1 |
| CD82 | CD82 |
| HIPK2 | HIPK2 |
| TUBB | TUBB |
| NPHP3 | NPHP3 |
| MIR9-1 | MIR9-1 |
| MYH9 | MYH9 |
| CYP19A1 | CYP19A1 |
| JAK1 | JAK1 |
| LINC00460 | LINC00460 |
| ELF3-AS1 | ELF3-AS1 |
| MALT1 | MALT1 |
| GLB1 | GLB1 |
| G6PD | G6PD |
| PRKG1 | PRKG1 |
| ZIC3 | ZIC3 |

| | |
|---|---|
| MIR186 | MIR186 |
| SH2D1A | SH2D1A |
| MIR424 | MIR424 |
| CD9 | CD9 |
| MEG8 | MEG8 |
| CSF1R | CSF1R |
| AQP5 | AQP5 |
| ITGAL | ITGAL |
| ITGA6 | ITGA6 |
| LIF | LIF |
| GSTA1 | GSTA1 |
| BCL2L11 | BCL2L11 |
| POU5F1 | POU5F1 |
| GRPR | GRPR |
| SHC1 | SHC1 |
| CCNH | CCNH |
| RMST | RMST |
| WDR19 | WDR19 |
| YBX1 | YBX1 |
| TNFRSF10A | TNFRSF10A |
| GADD45A | GADD45A |
| DHRS4-AS1 | DHRS4-AS1 |
| BLACAT1 | BLACAT1 |
| FGF9 | FGF9 |
| CCK | CCK |
| EFEMP2 | EFEMP2 |
| ROBO1 | ROBO1 |
| ASAH1 | ASAH1 |
| AIFM1 | AIFM1 |
| BICDL3P | BICDL3P |
| RSPH4A | RSPH4A |
| ZNF281 | ZNF281 |
| IL9 | IL9 |
| SMARCB1 | SMARCB1 |
| ELAVL1 | ELAVL1 |
| SMARCA5 | SMARCA5 |
| SNHG8 | SNHG8 |
| ARHGAP45 | ARHGAP45 |
| AR | AR |
| HPRT1 | HPRT1 |
| SERPINA3 | SERPINA3 |
| CXCL1 | CXCL1 |
| DSCAM-AS1 | DSCAM-AS1 |
| XRCC5 | XRCC5 |
| MYH11 | MYH11 |

| | |
|---|---|
| GMDS-DT | GMDS-DT |
| ALPL | ALPL |
| TCF4 | TCF4 |
| IGFBP5 | IGFBP5 |
| XRCC2 | XRCC2 |
| CCBE1 | CCBE1 |
| MTHFD1 | MTHFD1 |
| GFUS | GFUS |
| EFEMP1 | EFEMP1 |
| VIP | VIP |
| ZEB2-AS1 | ZEB2-AS1 |
| MIR149 | MIR149 |
| NEK8 | NEK8 |
| NES | NES |
| RXRB | RXRB |
| EPHX2 | EPHX2 |
| NF2 | NF2 |
| HP | HP |
| TMPRSS2 | TMPRSS2 |
| PPARA | PPARA |
| CELF1 | CELF1 |
| MKS1 | MKS1 |
| AGR2 | AGR2 |
| SIX1 | SIX1 |
| ATP8B1 | ATP8B1 |
| ARNT | ARNT |
| FLNC | FLNC |
| RNASE3 | RNASE3 |
| LINC01503 | LINC01503 |
| IGF2R | IGF2R |
| GAA | GAA |
| IL6ST | IL6ST |
| ODC1 | ODC1 |
| MTAP | MTAP |
| XRCC6 | XRCC6 |
| ASXL1 | ASXL1 |
| MINCR | MINCR |
| CTCF | CTCF |
| GDF1 | GDF1 |
| GAST | GAST |
| CTNND1 | CTNND1 |
| TRAF2 | TRAF2 |
| CIITA | CIITA |
| MTHFD2 | MTHFD2 |
| SOX17 | SOX17 |

| | |
|---|---|
| GHRL | GHRL |
| CLDN7 | CLDN7 |
| SOX13 | SOX13 |
| MELTF-AS1 | MELTF-AS1 |
| EPHA2 | EPHA2 |
| LDLR | LDLR |
| SERPINH1 | SERPINH1 |
| SKP2 | SKP2 |
| MIR26B | MIR26B |
| IFIH1 | IFIH1 |
| MIRLET7A1 | MIRLET7A1 |
| EZR | EZR |
| AFP | AFP |
| DLGAP1-AS1 | DLGAP1-AS1 |
| DCK | DCK |
| IFT140 | IFT140 |
| KMT2A | KMT2A |
| TATDN1 | TATDN1 |
| TYK2 | TYK2 |
| CHKA | CHKA |
| DARS1-AS1 | DARS1-AS1 |
| COL18A1 | COL18A1 |
| MIR503HG | MIR503HG |
| KCNN4 | KCNN4 |
| CSNK2A3 | CSNK2A3 |
| MIR29B1 | MIR29B1 |
| MASP2 | MASP2 |
| LYST | LYST |
| RAG2 | RAG2 |
| MTA1 | MTA1 |
| SCARNA5 | SCARNA5 |
| NPSR1-AS1 | NPSR1-AS1 |
| COL5A1 | COL5A1 |
| MSH3 | MSH3 |
| RASA1 | RASA1 |
| BMPR1B | BMPR1B |
| PDCD1LG2 | PDCD1LG2 |
| MME | MME |
| MIR378A | MIR378A |
| SEMA3B | SEMA3B |
| CFTR-AS1 | CFTR-AS1 |
| LINC01512 | LINC01512 |
| TNC | TNC |
| BARD1 | BARD1 |
| ITGA9 | ITGA9 |

| | |
|---|---|
| RPSA | RPSA |
| IGFBP7 | IGFBP7 |
| PINK1-AS | PINK1-AS |
| MIR193B | MIR193B |
| OSER1-DT | OSER1-DT |
| RUNX2 | RUNX2 |
| LINC00324 | LINC00324 |
| SLC16A1-AS1 | SLC16A1-AS1 |
| CLU | CLU |
| KAT7 | KAT7 |
| CXCL5 | CXCL5 |
| LINC01572 | LINC01572 |
| LAMB1 | LAMB1 |
| TCF3 | TCF3 |
| FASN | FASN |
| PRKACA | PRKACA |
| GANAB | GANAB |
| YY1 | YY1 |
| DNAL1 | DNAL1 |
| PRDX6 | PRDX6 |
| MIR379 | MIR379 |
| PTPRM | PTPRM |
| NCOA3 | NCOA3 |
| RCVRN | RCVRN |
| GFAP | GFAP |
| CTSL | CTSL |
| TSPOAP1-AS1 | TSPOAP1-AS1 |
| MIR196A1 | MIR196A1 |
| ADAMTS13 | ADAMTS13 |
| YWHAE | YWHAE |
| PDK1 | PDK1 |
| PKD2 | PKD2 |
| PHGDH | PHGDH |
| CD86 | CD86 |
| RHOB | RHOB |
| MAN2B1 | MAN2B1 |
| CCL18 | CCL18 |
| SKP1 | SKP1 |
| TAP2 | TAP2 |
| LRBA | LRBA |
| CD3E | CD3E |
| TGIF1 | TGIF1 |
| TNFSF11 | TNFSF11 |
| EXO1 | EXO1 |
| ACHE | ACHE |

HMHB1
TPM1
BCAR4
NT5E
BAD
DMD
FANCC
ARSB
EPB41
TPMT
DRC1
DNAI2
MIR181B1
CPA4
CCDC144NL-AS1
DIABLO
SAT1
HIPK3
TFAP2A
SNHG10
ASS1
HBEGF
F2RL3
FCGR3B
GAPLINC
DUXAP9
CLDN1
TRIP13
C4B
POU3F3
E2F2
BDNF
MYBPC3
NPC1
SYK
AURKB
UPF1
RNF32-DT
WNT7A
DCTN4
SPRY2
PNP
FOXP2
PRKCE
MIR574

HMHB1
TPM1
BCAR4
NT5E
BAD
DMD
FANCC
ARSB
EPB41
TPMT
DRC1
DNAI2
MIR181B1
CPA4
CCDC144NL-AS1
DIABLO
SAT1
HIPK3
TFAP2A
SNHG10
ASS1
HBEGF
F2RL3
FCGR3B
GAPLINC
DUXAP9
CLDN1
TRIP13
C4B
POU3F3
E2F2
BDNF
MYBPC3
NPC1
SYK
AURKB
UPF1
RNF32-DT
WNT7A
DCTN4
SPRY2
PNP
FOXP2
PRKCE
MIR574

| | |
|---|---|
| TTC7A | TTC7A |
| NEK6 | NEK6 |
| SLC7A5 | SLC7A5 |
| PTGS1 | PTGS1 |
| TP53COR1 | TP53COR1 |
| IKZF1 | IKZF1 |
| ITK | ITK |
| KIR2DL3 | KIR2DL3 |
| CYP27A1 | CYP27A1 |
| LCK | LCK |
| APAF1 | APAF1 |
| VPS9D1-AS1 | VPS9D1-AS1 |
| HHIP-AS1 | HHIP-AS1 |
| LIG3 | LIG3 |
| MITF | MITF |
| THORLNC | THORLNC |
| FSCN1 | FSCN1 |
| DCN | DCN |
| PRECSIT | PRECSIT |
| DRC2 | DRC2 |
| CENPF | CENPF |
| DNAAF2 | DNAAF2 |
| TLCD3A | TLCD3A |
| TRAF1 | TRAF1 |
| SERPINB5 | SERPINB5 |
| SERPINF1 | SERPINF1 |
| MEFV | MEFV |
| MIR532 | MIR532 |
| BTRC | BTRC |
| CD59 | CD59 |
| FAH | FAH |
| SCNN1G | SCNN1G |
| DVL1 | DVL1 |
| PTPN22 | PTPN22 |
| AXIN1 | AXIN1 |
| MYB | MYB |
| MECP2 | MECP2 |
| C4A | C4A |
| STAT5B | STAT5B |
| PLCG1 | PLCG1 |
| TTC21B | TTC21B |
| CDX2 | CDX2 |
| PTGER4 | PTGER4 |
| MCM3AP | MCM3AP |
| IL12A | IL12A |

| | |
|---|---|
| LTBP1 | LTBP1 |
| TLR5 | TLR5 |
| PTGIS | PTGIS |
| GALC | GALC |
| PTPN13 | PTPN13 |
| HOXC9 | HOXC9 |
| PSMB9 | PSMB9 |
| ABCA1 | ABCA1 |
| LINC00355 | LINC00355 |
| MIR130B | MIR130B |
| AOC3 | AOC3 |
| IFNA2 | IFNA2 |
| PLAG1 | PLAG1 |
| LAMA4 | LAMA4 |
| ASMTL-AS1 | ASMTL-AS1 |
| STMN1 | STMN1 |
| DNAAF11 | DNAAF11 |
| IDS | IDS |
| LINC01833 | LINC01833 |
| POR | POR |
| CEACAM1 | CEACAM1 |
| HPSE | HPSE |
| PGM3 | PGM3 |
| LBX2-AS1 | LBX2-AS1 |
| RBM5 | RBM5 |
| HNF4A | HNF4A |
| ITGB4 | ITGB4 |
| TNFRSF9 | TNFRSF9 |
| EPOR | EPOR |
| TERF1 | TERF1 |
| HYAL2 | HYAL2 |
| DNAAF19 | DNAAF19 |
| ETV6 | ETV6 |
| EIF2AK2 | EIF2AK2 |
| HMMR-AS1 | HMMR-AS1 |
| LINC00852 | LINC00852 |
| ST3GAL6-AS1 | ST3GAL6-AS1 |
| GBE1 | GBE1 |
| TK1 | TK1 |
| FRAS1 | FRAS1 |
| MIR92A1 | MIR92A1 |
| LINC01270 | LINC01270 |
| WNT1 | WNT1 |
| SLC9A3-AS1 | SLC9A3-AS1 |
| PXN | PXN |

| | |
|---|---|
| UCHL1 | UCHL1 |
| NPHP1 | NPHP1 |
| IFNGR2 | IFNGR2 |
| PLA2G4A | PLA2G4A |
| ITGA2B | ITGA2B |
| LAMP2 | LAMP2 |
| FOXP1 | FOXP1 |
| RECK | RECK |
| RBP4 | RBP4 |
| VTN | VTN |
| PSG2 | PSG2 |
| MIR494 | MIR494 |
| MIR582 | MIR582 |
| ADA2 | ADA2 |
| LTF | LTF |
| PIM1 | PIM1 |
| KNG1 | KNG1 |
| DRD2 | DRD2 |
| TPTEP1 | TPTEP1 |
| STRA6 | STRA6 |
| MIR24-1 | MIR24-1 |
| ACP5 | ACP5 |
| KLF4 | KLF4 |
| MIRLET7I | MIRLET7I |
| HDGF | HDGF |
| PRL | PRL |
| SSTR2 | SSTR2 |
| SDHB | SDHB |
| MIR590 | MIR590 |
| RAC2 | RAC2 |
| IRF4 | IRF4 |
| AP3B1 | AP3B1 |
| RNH1 | RNH1 |
| PIK3R2 | PIK3R2 |
| PRKAR1A | PRKAR1A |
| HYAL1 | HYAL1 |
| ARAF | ARAF |
| HSPD1 | HSPD1 |
| HSP90B1 | HSP90B1 |
| XBP1 | XBP1 |
| BMPR1A | BMPR1A |
| ATP2A2 | ATP2A2 |
| NLRC4 | NLRC4 |
| SDHAP1 | SDHAP1 |
| CR1 | CR1 |

| | |
|---|---|
| HNRNPU | HNRNPU |
| CCN1 | CCN1 |
| LNCRNA-ATB | LNCRNA-ATB |
| TLR1 | TLR1 |
| BRD4 | BRD4 |
| EPHA3 | EPHA3 |
| ABCB4 | ABCB4 |
| MIR330 | MIR330 |
| S100A9 | S100A9 |
| WIF1 | WIF1 |
| CYP2A13 | CYP2A13 |
| CDK5 | CDK5 |
| NKX2-5 | NKX2-5 |
| CD19 | CD19 |
| POLI | POLI |
| DDX5 | DDX5 |
| IGFBP1 | IGFBP1 |
| POLB | POLB |
| IL18R1 | IL18R1 |
| FGF23 | FGF23 |
| LINC00491 | LINC00491 |
| LMCD1-AS1 | LMCD1-AS1 |
| LAMA1 | LAMA1 |
| CEP290 | CEP290 |
| HAMP | HAMP |
| RARS1 | RARS1 |
| HDAC6 | HDAC6 |
| SLC6A14 | SLC6A14 |
| PRKCB | PRKCB |
| CALB2 | CALB2 |
| CCR6 | CCR6 |
| KAT5 | KAT5 |
| AQP1 | AQP1 |
| MIR483 | MIR483 |
| RYR2 | RYR2 |
| GAS6-DT | GAS6-DT |
| GRB2 | GRB2 |
| LYZ | LYZ |
| PRDX1 | PRDX1 |
| LINC00667 | LINC00667 |
| CTAG1B | CTAG1B |
| NOS1 | NOS1 |
| MIR196B | MIR196B |
| MIR409 | MIR409 |
| DNAH1 | DNAH1 |

MAGEA4
MIR361
CAVIN3
EWSR1
UGT1A7
MIR708
SEMA3F
EBLN3P
DUSP6
CTNNA1
HSP90AB1
XRCC4
CEACAM7
MVK
CD63
ETV4
AHRR
C5AR1
NR4A1
MAT2B
CCR4
ENPP2
COX4I2
HOXA5
FEN1
MIR138-1
KIR2DL4
CA9
WNT3
CCL22
TRPV4
MIR503
TRERNA1
CXADR
ESM1
DDIT3
STAT5A
IL24
PPM1D
GSR
MAGEA1
NME8
NCF4
MAPKAPK5-AS1
KIR2DL2

MAGEA4
MIR361
CAVIN3
EWSR1
UGT1A7
MIR708
SEMA3F
EBLN3P
DUSP6
CTNNA1
HSP90AB1
XRCC4
CEACAM7
MVK
CD63
ETV4
AHRR
C5AR1
NR4A1
MAT2B
CCR4
ENPP2
COX4I2
HOXA5
FEN1
MIR138-1
KIR2DL4
CA9
WNT3
CCL22
TRPV4
MIR503
TRERNA1
CXADR
ESM1
DDIT3
STAT5A
IL24
PPM1D
GSR
MAGEA1
NME8
NCF4
MAPKAPK5-AS1
KIR2DL2

| | |
|---|---|
| GDNF | GDNF |
| ST8SIA6-AS1 | ST8SIA6-AS1 |
| ARSA | ARSA |
| SNHG9 | SNHG9 |
| NEK1 | NEK1 |
| TYMSOS | TYMSOS |
| MIR30C1 | MIR30C1 |
| WWOX | WWOX |
| COL2A1 | COL2A1 |
| MIR495 | MIR495 |
| ZAP70 | ZAP70 |
| E2F3 | E2F3 |
| MIR320A | MIR320A |
| ING1 | ING1 |
| TCIM | TCIM |
| GPX3 | GPX3 |
| LINC01138 | LINC01138 |
| MIR17HG | MIR17HG |
| MACC1-AS1 | MACC1-AS1 |
| MAP2K4 | MAP2K4 |
| HSPG2 | HSPG2 |
| SYNE1 | SYNE1 |
| ZFPM2 | ZFPM2 |
| IL10RA | IL10RA |
| ST7-AS1 | ST7-AS1 |
| SIRT3 | SIRT3 |
| CDC25A | CDC25A |
| LIFR | LIFR |
| KRT14 | KRT14 |
| TERF2 | TERF2 |
| FAM83H | FAM83H |
| PGF | PGF |
| MIR328 | MIR328 |
| GDF2 | GDF2 |
| POMC | POMC |
| AIMP2 | AIMP2 |
| BAG1 | BAG1 |
| NMBR | NMBR |
| FLNB | FLNB |
| HEIH | HEIH |
| SNHG18 | SNHG18 |
| MCM2 | MCM2 |
| LINC01559 | LINC01559 |
| NAT1 | NAT1 |
| PPP1R13B-DT | PPP1R13B-DT |

| | |
|---|---|
| LAMC1 | LAMC1 |
| TBX1 | TBX1 |
| SREBF1 | SREBF1 |
| SMAD6 | SMAD6 |
| ABCA7 | ABCA7 |
| THBS2 | THBS2 |
| LINC01139 | LINC01139 |
| CASP7 | CASP7 |
| FGF8 | FGF8 |
| MIR134 | MIR134 |
| EIF4E | EIF4E |
| LZTR1 | LZTR1 |
| TRB | TRB |
| LATS2 | LATS2 |
| HCK | HCK |
| IRF8 | IRF8 |
| UBE2D2 | UBE2D2 |
| IRAK1 | IRAK1 |
| SULT1A1 | SULT1A1 |
| KDM4B | KDM4B |
| AKR1B10 | AKR1B10 |
| TOLLIP | TOLLIP |
| CXCL11 | CXCL11 |
| LINC00641 | LINC00641 |
| IFT172 | IFT172 |
| AK2 | AK2 |
| EMD | EMD |
| BBC3 | BBC3 |
| CCND2 | CCND2 |
| NEK9 | NEK9 |
| RXRG | RXRG |
| NCF1 | NCF1 |
| PRR11-AS1 | PRR11-AS1 |
| ARHGAP10 | ARHGAP10 |
| SLC9A3 | SLC9A3 |
| ALDH1A2 | ALDH1A2 |
| BTG2 | BTG2 |
| GADD45G | GADD45G |
| MIR136 | MIR136 |
| CD74 | CD74 |
| RIT1 | RIT1 |
| ABCC10 | ABCC10 |
| COMETT | COMETT |
| WEE1 | WEE1 |
| TGFBI | TGFBI |

| | |
|---|---|
| IMPDH2 | IMPDH2 |
| STAT4 | STAT4 |
| EIF2AK3 | EIF2AK3 |
| LACTB | LACTB |
| ADAR | ADAR |
| NMB | NMB |
| TNXB | TNXB |
| FOXN1 | FOXN1 |
| FGF18 | FGF18 |
| SDCCAG8 | SDCCAG8 |
| SUMO1P3 | SUMO1P3 |
| ITGA4 | ITGA4 |
| TNFSF13B | TNFSF13B |
| MIR28 | MIR28 |
| MIR210HG | MIR210HG |
| PI3 | PI3 |
| SERPINB1 | SERPINB1 |
| SMARCA2 | SMARCA2 |
| PSAP | PSAP |
| NOP10 | NOP10 |
| PGK1 | PGK1 |
| TCTN3 | TCTN3 |
| PIK3R3 | PIK3R3 |
| SNORD15A | SNORD15A |
| EWSAT1 | EWSAT1 |
| ASAP1-IT1 | ASAP1-IT1 |
| S100A2 | S100A2 |
| MMUT | MMUT |
| GALNS | GALNS |
| MIR584 | MIR584 |
| DROSHA | DROSHA |
| NR1H4 | NR1H4 |
| PLA2G6 | PLA2G6 |
| DKK3 | DKK3 |
| GJB2 | GJB2 |
| GACAT3 | GACAT3 |
| CD247 | CD247 |
| DIO3OS | DIO3OS |
| OTC | OTC |
| ADGRG6 | ADGRG6 |
| RPS27A | RPS27A |
| SPTAN1 | SPTAN1 |
| DPYSL5 | DPYSL5 |
| XPO5 | XPO5 |
| EDNRB | EDNRB |

| | |
|---|---|
| CUL1 | CUL1 |
| CXCR5 | CXCR5 |
| MAT2A | MAT2A |
| PTTG1 | PTTG1 |
| GADD45B | GADD45B |
| ARHGAP9 | ARHGAP9 |
| ARRB1 | ARRB1 |
| CKS1B | CKS1B |
| IGHE | IGHE |
| STIL | STIL |
| BMP1 | BMP1 |
| CDA | CDA |
| FOXC2-AS1 | FOXC2-AS1 |
| PRKAA1 | PRKAA1 |
| CAV3 | CAV3 |
| LINC01224 | LINC01224 |
| G6PC1 | G6PC1 |
| DACH1 | DACH1 |
| HLA-DRB5 | HLA-DRB5 |
| NCOR1 | NCOR1 |
| HOXB9 | HOXB9 |
| NHP2 | NHP2 |
| ZEB2 | ZEB2 |
| TRAF4 | TRAF4 |
| CCKBR | CCKBR |
| CSNK1A1 | CSNK1A1 |
| PPIE | PPIE |
| HBG2 | HBG2 |
| KDM1A | KDM1A |
| MAEA | MAEA |
| CRB2 | CRB2 |
| VIPR1 | VIPR1 |
| BUB1 | BUB1 |
| ACVR2B | ACVR2B |
| RECQL4 | RECQL4 |
| NPRL2 | NPRL2 |
| HK2 | HK2 |
| AIF1 | AIF1 |
| NR1H2 | NR1H2 |
| CCN4 | CCN4 |
| APOB | APOB |
| NFKB2 | NFKB2 |
| FPGS | FPGS |
| TIMELESS | TIMELESS |
| CD276 | CD276 |

| | |
|---|---|
| BCOR | BCOR |
| MAPKAPK3 | MAPKAPK3 |
| MCM4 | MCM4 |
| MIR455 | MIR455 |
| HDAC4 | HDAC4 |
| ALMS1 | ALMS1 |
| PIK3C2A | PIK3C2A |
| MIR615 | MIR615 |
| HAR1A | HAR1A |
| LRP5 | LRP5 |
| TP53BP1 | TP53BP1 |
| MIR133A1 | MIR133A1 |
| CDC25B | CDC25B |
| MIR218-1 | MIR218-1 |
| OSM | OSM |
| PTGES | PTGES |
| LPAR1 | LPAR1 |
| HDAC2 | HDAC2 |
| MIR135B | MIR135B |
| STK4 | STK4 |
| SOX11 | SOX11 |
| CD69 | CD69 |
| STIM1 | STIM1 |
| LINC01232 | LINC01232 |
| FANCD2 | FANCD2 |
| PTMA | PTMA |
| TLR8 | TLR8 |
| STX11 | STX11 |
| TNFRSF6B | TNFRSF6B |
| WNT3A | WNT3A |
| MIR135A1 | MIR135A1 |
| CHD7 | CHD7 |
| IBSP | IBSP |
| MIR154 | MIR154 |
| MIR340 | MIR340 |
| MED19 | MED19 |
| MRE11 | MRE11 |
| RBL2 | RBL2 |
| TGM2 | TGM2 |
| HMGA1 | HMGA1 |
| C11orf65 | C11orf65 |
| AKAP13 | AKAP13 |
| PPIA | PPIA |
| FGF20 | FGF20 |
| TMEM67 | TMEM67 |

| | |
|---|---|
| RRM2 | RRM2 |
| PDCD4 | PDCD4 |
| RPL5 | RPL5 |
| RIOX2 | RIOX2 |
| HSD17B1 | HSD17B1 |
| SRSF2 | SRSF2 |
| PDE4A | PDE4A |
| PEX1 | PEX1 |
| LAMB2 | LAMB2 |
| PIK3C3 | PIK3C3 |
| CHRM3 | CHRM3 |
| LEFTY2 | LEFTY2 |
| CHRNA7 | CHRNA7 |
| CTPS1 | CTPS1 |
| SUFU | SUFU |
| MAGEA3 | MAGEA3 |
| LIMD1 | LIMD1 |
| MTRR | MTRR |
| PLA2G2A | PLA2G2A |
| EREG | EREG |
| CDCP1 | CDCP1 |
| PITX2 | PITX2 |
| FAM138B | FAM138B |
| SAMHD1 | SAMHD1 |
| MB | MB |
| SDC1 | SDC1 |
| PRMT5 | PRMT5 |
| LIN28B-AS1 | LIN28B-AS1 |
| PPP1CB | PPP1CB |
| CSF3R | CSF3R |
| TMEM216 | TMEM216 |
| NGF | NGF |
| PPARD | PPARD |
| RAD23B | RAD23B |
| HES1 | HES1 |
| SAA1 | SAA1 |
| NAMPT | NAMPT |
| SRGAP1 | SRGAP1 |
| LPCAT1 | LPCAT1 |
| MMP10 | MMP10 |
| TRA-TGC7-1 | TRA-TGC7-1 |
| TRAF5 | TRAF5 |
| SGK1 | SGK1 |
| TNFAIP3 | TNFAIP3 |
| HPS6 | HPS6 |

| | |
|---|---|
| IL10RB | IL10RB |
| PROC | PROC |
| MAGT1 | MAGT1 |
| SEC61A1 | SEC61A1 |
| NOX4 | NOX4 |
| LBP | LBP |
| PAX8 | PAX8 |
| PML | PML |
| FOLR1 | FOLR1 |
| ECT2 | ECT2 |
| CACNA1C | CACNA1C |
| PWAR1 | PWAR1 |
| SDHD | SDHD |
| INSM1 | INSM1 |
| PROCR | PROCR |
| C1S | C1S |
| MIR296 | MIR296 |
| RPGRIP1L | RPGRIP1L |
| DCLRE1C | DCLRE1C |
| LEPR | LEPR |
| MIR103A1 | MIR103A1 |
| ELAVL4 | ELAVL4 |
| FOXH1 | FOXH1 |
| CHIT1 | CHIT1 |
| CTSK | CTSK |
| MIR545 | MIR545 |
| AQP3 | AQP3 |
| PCSK9 | PCSK9 |
| FANCF | FANCF |
| GPX4 | GPX4 |
| PSMA4 | PSMA4 |
| SLC2A3 | SLC2A3 |
| H2AX | H2AX |
| USP8 | USP8 |
| NFATC2 | NFATC2 |
| CACNA2D2 | CACNA2D2 |
| MIR370 | MIR370 |
| LGALS3BP | LGALS3BP |
| CD47 | CD47 |
| TBX2 | TBX2 |
| CD151 | CD151 |
| KDM6A | KDM6A |
| RALBP1 | RALBP1 |
| LAG3 | LAG3 |
| ANLN | ANLN |

| | |
|---|---|
| HADHA | HADHA |
| SLC22A5 | SLC22A5 |
| IL23R | IL23R |
| MIR302A | MIR302A |
| FANCL | FANCL |
| KL | KL |
| CARD11 | CARD11 |
| ZBTB17 | ZBTB17 |
| INF2 | INF2 |
| MIR485 | MIR485 |
| NR1I2 | NR1I2 |
| TRIM28 | TRIM28 |
| CXCL13 | CXCL13 |
| SERPINB2 | SERPINB2 |
| IFNB1 | IFNB1 |
| MIR302C | MIR302C |
| CCNE2 | CCNE2 |
| XPO1 | XPO1 |
| MIR362 | MIR362 |
| MAP3K2 | MAP3K2 |
| CD2AP | CD2AP |
| NTS | NTS |
| TFPI2 | TFPI2 |
| ADAM15 | ADAM15 |
| CUBN | CUBN |
| TLE1 | TLE1 |
| PPIG | PPIG |
| RETN | RETN |
| PRKCSH | PRKCSH |
| SF3B1 | SF3B1 |
| CCL20 | CCL20 |
| FREM2 | FREM2 |
| TBXT | TBXT |
| MIR128-1 | MIR128-1 |
| SLC40A1 | SLC40A1 |
| SMARCAL1 | SMARCAL1 |
| PLCE1 | PLCE1 |
| IL17F | IL17F |
| EYA1 | EYA1 |
| CD3D | CD3D |
| MGST1 | MGST1 |
| BPIFB1 | BPIFB1 |
| FOSL1 | FOSL1 |
| TGM1 | TGM1 |
| RAD52 | RAD52 |

| | |
|---|---|
| F2RL1 | F2RL1 |
| FUS | FUS |
| ITGB6 | ITGB6 |
| TUBA1B | TUBA1B |
| NPHP4 | NPHP4 |
| SPI1 | SPI1 |
| MPG | MPG |
| F2R | F2R |
| PDP1 | PDP1 |
| CRYAB | CRYAB |
| CR2 | CR2 |
| CDC25C | CDC25C |
| MIR188 | MIR188 |
| ELFN1-AS1 | ELFN1-AS1 |
| MICB | MICB |
| TMSB10 | TMSB10 |
| FANCE | FANCE |
| WIPF1 | WIPF1 |
| PLCG2 | PLCG2 |
| HBA1 | HBA1 |
| S100A8 | S100A8 |
| ACY1 | ACY1 |
| VCP | VCP |
| GRIP1 | GRIP1 |
| OAS1 | OAS1 |
| MIR33B | MIR33B |
| MIR365A | MIR365A |
| PODXL | PODXL |
| SDHC | SDHC |
| PIGA | PIGA |
| MIR625 | MIR625 |
| ATG7 | ATG7 |
| BAG6 | BAG6 |
| HNF1A | HNF1A |
| MMACHC | MMACHC |
| PCGEM1 | PCGEM1 |
| MIR7-1 | MIR7-1 |
| CAV2 | CAV2 |
| MIR490 | MIR490 |
| SAMD9 | SAMD9 |
| ITGAX | ITGAX |
| S100A1 | S100A1 |
| MSR1 | MSR1 |
| ACTA1 | ACTA1 |
| MCM3 | MCM3 |

| | |
|---|---|
| TNFRSF13B | TNFRSF13B |
| GATA5 | GATA5 |
| MIR20B | MIR20B |
| GPX1 | GPX1 |
| EXT1 | EXT1 |
| PNKP | PNKP |
| MIR331 | MIR331 |
| FCN3 | FCN3 |
| HSF1 | HSF1 |
| AKR1C3 | AKR1C3 |
| RIPK1 | RIPK1 |
| IFNAR1 | IFNAR1 |
| TNFSF12 | TNFSF12 |
| ALPP | ALPP |
| TH | TH |
| HLA-DRA | HLA-DRA |
| CUX1 | CUX1 |
| VCL | VCL |
| WFDC2 | WFDC2 |
| FANCG | FANCG |
| RSPO2 | RSPO2 |
| MGP | MGP |
| TRC-GCA24-1 | TRC-GCA24-1 |
| COL14A1 | COL14A1 |
| TLR6 | TLR6 |
| MMP11 | MMP11 |
| SOS2 | SOS2 |
| SLC37A4 | SLC37A4 |
| MIR99B | MIR99B |
| PRKCD | PRKCD |
| TCIRG1 | TCIRG1 |
| ASL | ASL |
| FUT4 | FUT4 |
| UMOD | UMOD |
| RAD51C | RAD51C |
| HBA2 | HBA2 |
| CTNS | CTNS |
| EFNB3 | EFNB3 |
| BMX | BMX |
| NTHL1 | NTHL1 |
| CYP7B1 | CYP7B1 |
| CSTB | CSTB |
| LOC111674472 | LOC111674472 |
| UBA7 | UBA7 |
| FECH | FECH |

| | |
|---|---|
| MYO1G | MYO1G |
| IRF5 | IRF5 |
| MIR675 | MIR675 |
| AVP | AVP |
| PDE5A | PDE5A |
| SMARCE1 | SMARCE1 |
| COPS5 | COPS5 |
| PSMB8 | PSMB8 |
| KCNQ1 | KCNQ1 |
| CDK7 | CDK7 |
| CD38 | CD38 |
| PADI4 | PADI4 |
| STUB1 | STUB1 |
| PPP2R2A | PPP2R2A |
| FGF3 | FGF3 |
| SMC1A | SMC1A |
| SGCD | SGCD |
| DOCK2 | DOCK2 |
| FYN | FYN |
| PEBP1 | PEBP1 |
| PXDN | PXDN |
| CD22 | CD22 |
| ALOX12 | ALOX12 |
| IRF3 | IRF3 |
| CHD4 | CHD4 |
| MST1 | MST1 |
| EMP2 | EMP2 |
| TBK1 | TBK1 |
| MYH6 | MYH6 |
| NNMT | NNMT |
| SHMT1 | SHMT1 |
| PTPRD | PTPRD |
| PTH | PTH |
| MIR501 | MIR501 |
| FHL1 | FHL1 |
| PKD1L1 | PKD1L1 |
| AGRN | AGRN |
| CLDN14-AS1 | CLDN14-AS1 |
| PLEC | PLEC |
| CBLB | CBLB |
| SH3GL1 | SH3GL1 |
| LATS1 | LATS1 |
| MIR382 | MIR382 |
| DDX41 | DDX41 |
| RPA1 | RPA1 |

| | |
|---|---|
| AHI1 | AHI1 |
| SLC6A3 | SLC6A3 |
| PRNP | PRNP |
| CTSG | CTSG |
| HSD11B2 | HSD11B2 |
| IGF2BP3 | IGF2BP3 |
| TCTN2 | TCTN2 |
| FKRP | FKRP |
| PDE3B | PDE3B |
| COL11A1 | COL11A1 |
| ABCC5 | ABCC5 |
| P2RX7 | P2RX7 |
| CLDN4 | CLDN4 |
| MIR301A | MIR301A |
| RASGRP1 | RASGRP1 |
| IL17RA | IL17RA |
| CAP1 | CAP1 |
| MIR196A2 | MIR196A2 |
| FGL1 | FGL1 |
| LCAT | LCAT |
| SKIC2 | SKIC2 |
| LOC111674475 | LOC111674475 |
| FHL2 | FHL2 |
| CS | CS |
| MYO5A | MYO5A |
| FANCB | FANCB |
| TARDBP | TARDBP |
| CRABP2 | CRABP2 |
| MBD2 | MBD2 |
| PINK1 | PINK1 |
| PBX1 | PBX1 |
| TNFRSF10D | TNFRSF10D |
| GRHPR | GRHPR |
| DEFB4A | DEFB4A |
| MAD2L1 | MAD2L1 |
| NOG | NOG |
| VDAC1 | VDAC1 |
| PSMC4 | PSMC4 |
| LINC02893 | LINC02893 |
| CYSLTR1 | CYSLTR1 |
| USP7 | USP7 |
| SLIT2 | SLIT2 |
| TNFRSF11A | TNFRSF11A |
| DNM2 | DNM2 |
| PAX6 | PAX6 |

| | |
|---|---|
| MKKS | MKKS |
| SH2B3 | SH2B3 |
| SLC28A1 | SLC28A1 |
| TBX20 | TBX20 |
| CYP2R1 | CYP2R1 |
| HOXC13 | HOXC13 |
| SETBP1 | SETBP1 |
| TIA1 | TIA1 |
| H1-0 | H1-0 |
| OCA2 | OCA2 |
| CYBC1 | CYBC1 |
| AMFR | AMFR |
| ECE1 | ECE1 |
| UGT2B7 | UGT2B7 |
| DLL4 | DLL4 |
| SLCO1B1 | SLCO1B1 |
| KIAA0586 | KIAA0586 |
| SDHA | SDHA |
| SPRED1 | SPRED1 |
| LOXL2 | LOXL2 |
| IGBP1 | IGBP1 |
| TXNRD1 | TXNRD1 |
| MIR369 | MIR369 |
| MIR491 | MIR491 |
| TG | TG |
| WDR35 | WDR35 |
| PLOD1 | PLOD1 |
| AGL | AGL |
| LNCARSR | LNCARSR |
| CD68 | CD68 |
| ENTPD1 | ENTPD1 |
| APP | APP |
| COQ8B | COQ8B |
| MIR19B1 | MIR19B1 |
| KIR2DL5A | KIR2DL5A |
| FOXK2 | FOXK2 |
| PRKD1 | PRKD1 |
| FCN2 | FCN2 |
| NFKBIB | NFKBIB |
| HFE-AS1 | HFE-AS1 |
| PF4 | PF4 |
| HABP2 | HABP2 |
| EDN3 | EDN3 |
| MYOD1 | MYOD1 |
| CEP83 | CEP83 |

| | |
|---|---|
| UROS | UROS |
| CORO1A | CORO1A |
| PTGER2 | PTGER2 |
| ELP1 | ELP1 |
| ABCG1 | ABCG1 |
| LGALS9 | LGALS9 |
| FAT4 | FAT4 |
| CYP2B6 | CYP2B6 |
| MECOM | MECOM |
| WNT2 | WNT2 |
| CMA1 | CMA1 |
| CLEC1A | CLEC1A |
| IL1RAPL2 | IL1RAPL2 |
| MAP3K14 | MAP3K14 |
| MIR190A | MIR190A |
| ITGA8 | ITGA8 |
| FOXF2 | FOXF2 |
| MIR487B | MIR487B |
| CCNA1 | CCNA1 |
| SPINT2 | SPINT2 |
| NEDD9 | NEDD9 |
| HHIP | HHIP |
| UGT1A9 | UGT1A9 |
| PIAS3 | PIAS3 |
| DDX3X | DDX3X |
| ADH1C | ADH1C |
| FTL | FTL |
| SBDS | SBDS |
| TRIM8 | TRIM8 |
| FLI1 | FLI1 |
| MSRA | MSRA |
| BECN1 | BECN1 |
| BBS1 | BBS1 |
| DLC1 | DLC1 |
| MYPN | MYPN |
| ABCB7 | ABCB7 |
| NFIB | NFIB |
| SLFN11 | SLFN11 |
| IL21 | IL21 |
| FURIN | FURIN |
| FKBP5 | FKBP5 |
| CD52 | CD52 |
| MS4A1 | MS4A1 |
| RPL13A | RPL13A |
| HCG18 | HCG18 |

| | |
|---|---|
| APOA4 | APOA4 |
| GRHL2 | GRHL2 |
| KIF14 | KIF14 |
| SERPINB3 | SERPINB3 |
| FAT1 | FAT1 |
| GSTM4 | GSTM4 |
| TRPS1 | TRPS1 |
| SLC29A1 | SLC29A1 |
| TP53BP2 | TP53BP2 |
| ROR2 | ROR2 |
| RANBP9 | RANBP9 |
| UBA1 | UBA1 |
| DLL3 | DLL3 |
| APOA2 | APOA2 |
| CYLD | CYLD |
| ERG | ERG |
| MTDH | MTDH |
| ABCD1 | ABCD1 |
| PTRH2 | PTRH2 |
| HOXA9 | HOXA9 |
| CFB | CFB |
| TTF1 | TTF1 |
| HSD17B4 | HSD17B4 |
| UNG | UNG |
| PAPPA | PAPPA |
| CITED2 | CITED2 |
| CPSF6 | CPSF6 |
| SPRED2 | SPRED2 |
| PCLAF | PCLAF |
| CRELD1 | CRELD1 |
| TYR | TYR |
| CYP27B1 | CYP27B1 |
| ABCC8 | ABCC8 |
| PABPN1 | PABPN1 |
| MIR542 | MIR542 |
| COL5A2 | COL5A2 |
| TSLP | TSLP |
| BPI | BPI |
| PHOX2B | PHOX2B |
| TBXAS1 | TBXAS1 |
| FIRRE | FIRRE |
| TJP1 | TJP1 |
| FH | FH |
| BMAL2 | BMAL2 |
| CYP4B1 | CYP4B1 |

MIR337
CEACAM8
RAPSN
FOXC2
LYN
CAVIN1
CLTC
KMT2C
TRE-TTC3-1
SCN5A
RPL11
MBD1
CBR1
SALL1
ALAD
DLL1
RFXANK
SQSTM1
FTH1
MIR202
RANBP2
FOXP4-AS1
LTBP3
RAD9A
ATOH1
ADAM19
MIR299
ACVR1
KLRC1
ADAM28
MIR452
PTP4A3
ADRB1
MAD1L1
LSM1
UROD
FGF4
SAMD9L
TRMU
CRKL
STK36
COL6A1
TRAP1
CLN6
SEMA3E

MIR337
CEACAM8
RAPSN
FOXC2
LYN
CAVIN1
CLTC
KMT2C
TRE-TTC3-1
SCN5A
RPL11
MBD1
CBR1
SALL1
ALAD
DLL1
RFXANK
SQSTM1
FTH1
MIR202
RANBP2
FOXP4-AS1
LTBP3
RAD9A
ATOH1
ADAM19
MIR299
ACVR1
KLRC1
ADAM28
MIR452
PTP4A3
ADRB1
MAD1L1
LSM1
UROD
FGF4
SAMD9L
TRMU
CRKL
STK36
COL6A1
TRAP1
CLN6
SEMA3E

| | |
|---|---|
| NCOR2 | NCOR2 |
| RAD51B | RAD51B |
| TRA | TRA |
| DMAP1 | DMAP1 |
| UMPS | UMPS |
| DSG2 | DSG2 |
| PRKAG2 | PRKAG2 |
| TBX21 | TBX21 |
| PIP | PIP |
| ELAC2 | ELAC2 |
| MC1R | MC1R |
| MIR885 | MIR885 |
| B9D2 | B9D2 |
| IGFBP4 | IGFBP4 |
| FLT3LG | FLT3LG |
| SRSF1 | SRSF1 |
| MIR500A | MIR500A |
| BRS3 | BRS3 |
| CHD8 | CHD8 |
| HAS2 | HAS2 |
| ZNF350 | ZNF350 |
| COPS2 | COPS2 |
| KRT17 | KRT17 |
| CD5 | CD5 |
| ARID1B | ARID1B |
| SRF | SRF |
| TAC1 | TAC1 |
| IL13RA1 | IL13RA1 |
| PEX5 | PEX5 |
| YWHAZ | YWHAZ |
| MIR129-1 | MIR129-1 |
| ZCCHC8 | ZCCHC8 |
| BBS2 | BBS2 |
| ABCG8 | ABCG8 |
| LAMP1 | LAMP1 |
| GALK1 | GALK1 |
| AVPR2 | AVPR2 |
| EIF4EBP1 | EIF4EBP1 |
| TEP1 | TEP1 |
| PRKCG | PRKCG |
| PTPN1 | PTPN1 |
| FBP1 | FBP1 |
| AP3D1 | AP3D1 |
| STAG2 | STAG2 |
| TUBA1A | TUBA1A |

| | |
|---|---|
| FMO3 | FMO3 |
| PTPN3 | PTPN3 |
| CSK | CSK |
| MRTFA | MRTFA |
| LINC00941 | LINC00941 |
| MMAA | MMAA |
| F13A1 | F13A1 |
| MADD | MADD |
| PLA2G7 | PLA2G7 |
| MIR181A2 | MIR181A2 |
| DIS3L2 | DIS3L2 |
| NARS1 | NARS1 |
| ADCY10 | ADCY10 |
| CERS1 | CERS1 |
| C5 | C5 |
| SRPK1 | SRPK1 |
| CDC73 | CDC73 |
| TPM2 | TPM2 |
| MYL2 | MYL2 |
| AMACR | AMACR |
| CDC6 | CDC6 |
| SIX3 | SIX3 |
| IL25 | IL25 |
| MIR628 | MIR628 |
| MIR506 | MIR506 |
| PEPD | PEPD |
| WWTR1 | WWTR1 |
| IFT81 | IFT81 |
| PIEZO1 | PIEZO1 |
| ETS2 | ETS2 |
| MRC1 | MRC1 |
| HSPA9 | HSPA9 |
| MIR367 | MIR367 |
| IGH | IGH |
| MIR1207 | MIR1207 |
| LYVE1 | LYVE1 |
| BBS9 | BBS9 |
| TNKS | TNKS |
| AHCY | AHCY |
| HIC1 | HIC1 |
| MIR381 | MIR381 |
| PRDM1 | PRDM1 |
| RBPJ | RBPJ |
| MIR519D | MIR519D |
| USF2 | USF2 |

| | |
|---|---|
| SMARCD2 | SMARCD2 |
| FOXC1 | FOXC1 |
| IFNL3 | IFNL3 |
| KIR2DL1 | KIR2DL1 |
| HGSNAT | HGSNAT |
| RPS14 | RPS14 |
| GHR | GHR |
| CXCR6 | CXCR6 |
| MID1 | MID1 |
| INSR | INSR |
| DYSF | DYSF |
| NOP2 | NOP2 |
| IFNAR2 | IFNAR2 |
| HNRNPA1 | HNRNPA1 |
| S1PR1 | S1PR1 |
| MMP19 | MMP19 |
| TBX3 | TBX3 |
| AICDA | AICDA |
| HNMT | HNMT |
| TPI1 | TPI1 |
| KAT6B | KAT6B |
| RPS24 | RPS24 |
| ERN1 | ERN1 |
| RBCK1 | RBCK1 |
| BBS4 | BBS4 |
| KRT10 | KRT10 |
| KLK3 | KLK3 |
| LDHA | LDHA |
| JUNB | JUNB |
| LINC01133 | LINC01133 |
| ATP5F1A | ATP5F1A |
| MDM4 | MDM4 |
| BCKDHB | BCKDHB |
| SNORD44 | SNORD44 |
| LOXL1 | LOXL1 |
| HAVCR1 | HAVCR1 |
| SP3 | SP3 |
| REST | REST |
| F9 | F9 |
| ABCG5 | ABCG5 |
| TFAP2B | TFAP2B |
| IGHMBP2 | IGHMBP2 |
| CTSC | CTSC |
| DSTYK | DSTYK |
| PTH1R | PTH1R |

| | |
|---|---|
| RPS19 | RPS19 |
| SCARB2 | SCARB2 |
| GLS | GLS |
| ATP13A3 | ATP13A3 |
| CD70 | CD70 |
| PAK6 | PAK6 |
| MIR22HG | MIR22HG |
| ZNF469 | ZNF469 |
| ODAD3 | ODAD3 |
| INTS6 | INTS6 |
| RASA2 | RASA2 |
| GJA5 | GJA5 |
| GPI | GPI |
| MED12 | MED12 |
| BRDT | BRDT |
| TMEM237 | TMEM237 |
| LOC107133510 | LOC107133510 |
| UBE2I | UBE2I |
| HOXA10 | HOXA10 |
| FUCA1 | FUCA1 |
| MIR520A | MIR520A |
| TRRAP | TRRAP |
| MIR502 | MIR502 |
| FKBP1A | FKBP1A |
| RFX5 | RFX5 |
| SLC7A11 | SLC7A11 |
| SLC16A1 | SLC16A1 |
| SELPLG | SELPLG |
| TPM3 | TPM3 |
| DZIP1L | DZIP1L |
| MIR505 | MIR505 |
| BRMS1 | BRMS1 |
| ZRSR2 | ZRSR2 |
| ACTG1 | ACTG1 |
| F10 | F10 |
| TMEM231 | TMEM231 |
| SALL4 | SALL4 |
| IRAK4 | IRAK4 |
| TNFRSF13C | TNFRSF13C |
| NEB | NEB |
| MIR877 | MIR877 |
| DEFB1 | DEFB1 |
| SOX18 | SOX18 |
| PMM2 | PMM2 |
| PBRM1 | PBRM1 |

| | |
|---|---|
| FCGR2B | FCGR2B |
| PPARGC1A | PPARGC1A |
| RELN | RELN |
| IQSEC1 | IQSEC1 |
| SERPINF2 | SERPINF2 |
| PAX9 | PAX9 |
| SHROOM3 | SHROOM3 |
| RFC2 | RFC2 |
| SERPINE2 | SERPINE2 |
| B3GAT1 | B3GAT1 |
| SFN | SFN |
| IL12RB2 | IL12RB2 |
| LOC106099062 | LOC106099062 |
| TFPI | TFPI |
| CYP11A1 | CYP11A1 |
| CDH23 | CDH23 |
| SNCA | SNCA |
| MIR411 | MIR411 |
| RELB | RELB |
| NRP2 | NRP2 |
| MEIS2 | MEIS2 |
| SF3A3 | SF3A3 |
| CA8 | CA8 |
| PLXND1 | PLXND1 |
| MLH3 | MLH3 |
| IGF2BP2 | IGF2BP2 |
| BBS10 | BBS10 |
| FANCI | FANCI |
| TGFBR3 | TGFBR3 |
| SCARB1 | SCARB1 |
| SIX2 | SIX2 |
| ARHGAP24 | ARHGAP24 |
| MAP3K1 | MAP3K1 |
| ZBTB7A | ZBTB7A |
| ACKR1 | ACKR1 |
| KCNH2 | KCNH2 |
| VEGFB | VEGFB |
| ABT1 | ABT1 |
| DSC2 | DSC2 |
| AATF | AATF |
| LINC00963 | LINC00963 |
| MIR873 | MIR873 |
| C1QB | C1QB |
| RNF43 | RNF43 |
| AHSG | AHSG |

| | |
|---|---|
| ARHGDIA | ARHGDIA |
| MIR671 | MIR671 |
| CUL5 | CUL5 |
| ANKS6 | ANKS6 |
| EPRS1 | EPRS1 |
| HMBS | HMBS |
| DGUOK | DGUOK |
| NUP85 | NUP85 |
| HLA-DRB3 | HLA-DRB3 |
| HTATIP2 | HTATIP2 |
| MUC2 | MUC2 |
| LPP | LPP |
| ATF6 | ATF6 |
| HOXA1 | HOXA1 |
| IL13RA2 | IL13RA2 |
| PCCA | PCCA |
| PDGFRL | PDGFRL |
| PKP2 | PKP2 |
| S100A13 | S100A13 |
| SOCS2 | SOCS2 |
| HSPA1L | HSPA1L |
| IRAK3 | IRAK3 |
| ICOSLG | ICOSLG |
| SPINK1 | SPINK1 |
| MUC3A | MUC3A |
| MIR627 | MIR627 |
| CPLANE1 | CPLANE1 |
| SLC39A7 | SLC39A7 |
| MIR493 | MIR493 |
| IL23A | IL23A |
| NR3C2 | NR3C2 |
| PLA2R1 | PLA2R1 |
| HOXB2 | HOXB2 |
| MDC1 | MDC1 |
| MAP2 | MAP2 |
| RRAS | RRAS |
| POLH | POLH |
| KRT13 | KRT13 |
| DUOX2 | DUOX2 |
| SPRY4 | SPRY4 |
| DNASE1 | DNASE1 |
| CD58 | CD58 |
| GAS1 | GAS1 |
| TCTN1 | TCTN1 |
| ELAVL2 | ELAVL2 |

| | |
|---|---|
| IFT80 | IFT80 |
| APOH | APOH |
| IRF2BP2 | IRF2BP2 |
| GH1 | GH1 |
| GNPTAB | GNPTAB |
| MIR605 | MIR605 |
| PARK7 | PARK7 |
| PRDM14 | PRDM14 |
| RPL36A-HNRNPH2 | RPL36A-HNRNPH2 |
| MYO18A | MYO18A |
| SGCB | SGCB |
| PRMT1 | PRMT1 |
| SIX5 | SIX5 |
| ATF3 | ATF3 |
| NOVA1 | NOVA1 |
| NPY | NPY |
| HDAC3 | HDAC3 |
| CHRND | CHRND |
| TOB1 | TOB1 |
| DPAGT1 | DPAGT1 |
| MIR551B | MIR551B |
| CEP120 | CEP120 |
| NFKBIL1 | NFKBIL1 |
| TAFAZZIN | TAFAZZIN |
| ADORA2B | ADORA2B |
| GNS | GNS |
| HSD3B7 | HSD3B7 |
| GRK2 | GRK2 |
| PLS3 | PLS3 |
| APLN | APLN |
| CDK8 | CDK8 |
| TPBG | TPBG |
| CAPNS1 | CAPNS1 |
| POLR2A | POLR2A |
| MATR3 | MATR3 |
| MT-CYB | MT-CYB |
| WNT2B | WNT2B |
| CDH3 | CDH3 |
| KHSRP | KHSRP |
| MBP | MBP |
| EFNB2 | EFNB2 |
| LIPC | LIPC |
| LTBP2 | LTBP2 |
| MX1 | MX1 |

| | |
|---|---|
| PAFAH1B1 | PAFAH1B1 |
| BDKRB2 | BDKRB2 |
| FMR1 | FMR1 |
| FUBP1 | FUBP1 |
| METTL13 | METTL13 |
| MIR206 | MIR206 |
| MYO1E | MYO1E |
| ELAVL3 | ELAVL3 |
| AGA | AGA |
| CALM3 | CALM3 |
| IQGAP1 | IQGAP1 |
| APRT | APRT |
| MIR187 | MIR187 |
| NUP133 | NUP133 |
| CEBPE | CEBPE |
| AIRE | AIRE |
| ID3 | ID3 |
| SETD1A | SETD1A |
| ACD | ACD |
| BBS7 | BBS7 |
| LRPPRC | LRPPRC |
| CCL26 | CCL26 |
| SUZ12 | SUZ12 |
| MIR92B | MIR92B |
| RHEB | RHEB |
| MAPK10 | MAPK10 |
| CTDSPL | CTDSPL |
| SRP54 | SRP54 |
| A2M | A2M |
| RAB5A | RAB5A |
| LMNB1 | LMNB1 |
| HOXB5 | HOXB5 |
| ID2 | ID2 |
| PMS1 | PMS1 |
| JMJD6 | JMJD6 |
| MIR1228 | MIR1228 |
| TMPO | TMPO |
| MPV17 | MPV17 |
| NHEJ1 | NHEJ1 |
| NUP98 | NUP98 |
| CRIPTO | CRIPTO |
| PAH | PAH |
| NECTIN4 | NECTIN4 |
| PIN1 | PIN1 |
| CTTN | CTTN |

| | |
|---|---|
| CFL1 | CFL1 |
| NSD3 | NSD3 |
| CHST14 | CHST14 |
| MIR16-2 | MIR16-2 |
| CX3CL1 | CX3CL1 |
| MIR498 | MIR498 |
| SGSH | SGSH |
| BCL2A1 | BCL2A1 |
| CLCN1 | CLCN1 |
| B9D1 | B9D1 |
| HAX1 | HAX1 |
| SPON2 | SPON2 |
| MIR489 | MIR489 |
| SLC1A5 | SLC1A5 |
| MSN | MSN |
| NOTCH4 | NOTCH4 |
| PPBP | PPBP |
| NOX1 | NOX1 |
| AIMP1 | AIMP1 |
| NANOG | NANOG |
| DAB2 | DAB2 |
| TRAF3IP1 | TRAF3IP1 |
| MIR515-1 | MIR515-1 |
| DDAH2 | DDAH2 |
| SPHK1 | SPHK1 |
| SHOC2 | SHOC2 |
| MIR508 | MIR508 |
| TTK | TTK |
| CISH | CISH |
| C4BPA | C4BPA |
| CD99 | CD99 |
| MIR654 | MIR654 |
| MMP21 | MMP21 |
| LINC01082 | LINC01082 |
| TSHR | TSHR |
| MIR1224 | MIR1224 |
| CLDN5 | CLDN5 |
| SFRP1 | SFRP1 |
| LRP2 | LRP2 |
| BCS1L | BCS1L |
| GATA1 | GATA1 |
| WNT11 | WNT11 |
| GSS | GSS |
| LMX1B | LMX1B |
| SKI | SKI |

| | |
|---|---|
| MAPK7 | MAPK7 |
| MYOCD | MYOCD |
| TAGLN | TAGLN |
| ATIC | ATIC |
| CTSH | CTSH |
| NAT10 | NAT10 |
| CEP164 | CEP164 |
| NR2F2 | NR2F2 |
| ACTN1 | ACTN1 |
| PYCR1 | PYCR1 |
| DVL3 | DVL3 |
| VANGL1 | VANGL1 |
| ACADVL | ACADVL |
| SETDB1 | SETDB1 |
| FBL | FBL |
| RPL3 | RPL3 |
| SELENBP1 | SELENBP1 |
| IGF2BP1 | IGF2BP1 |
| MIR512-1 | MIR512-1 |
| TPSAB1 | TPSAB1 |
| GNE | GNE |
| SLC6A4 | SLC6A4 |
| LTBR | LTBR |
| ALG9 | ALG9 |
| HOXB13 | HOXB13 |
| MIR744 | MIR744 |
| ANXA3 | ANXA3 |
| HMMR | HMMR |
| CLIC1 | CLIC1 |
| TRIM65 | TRIM65 |
| PSTPIP1 | PSTPIP1 |
| ANKRD26 | ANKRD26 |
| MIR326 | MIR326 |
| MIR371A | MIR371A |
| IL15RA | IL15RA |
| DTNBP1 | DTNBP1 |
| PEX6 | PEX6 |
| IQGAP3 | IQGAP3 |
| PTPRO | PTPRO |
| NBAS | NBAS |
| BHLHE40 | BHLHE40 |
| SERPING1 | SERPING1 |
| EDN2 | EDN2 |
| HOXA11 | HOXA11 |
| TAB2 | TAB2 |

| | |
|---|---|
| NUP214 | NUP214 |
| SMC3 | SMC3 |
| AMBP | AMBP |
| SPDEF | SPDEF |
| GLIS2 | GLIS2 |
| TRIM32 | TRIM32 |
| MIR383 | MIR383 |
| KAT6A | KAT6A |
| GRK5 | GRK5 |
| IHH | IHH |
| MIR454 | MIR454 |
| GPC4 | GPC4 |
| SCT | SCT |
| HPS3 | HPS3 |
| LRRC37A2 | LRRC37A2 |
| ADAM10 | ADAM10 |
| IKBKE | IKBKE |
| PXDNL | PXDNL |
| RLBP1 | RLBP1 |
| GRN | GRN |
| GLUL | GLUL |
| TBL1XR1 | TBL1XR1 |
| COL6A3 | COL6A3 |
| SS18 | SS18 |
| SMN1 | SMN1 |
| TAPBP | TAPBP |
| PSAT1 | PSAT1 |
| AXDND1 | AXDND1 |
| RHO | RHO |
| TNS1 | TNS1 |
| WNT9B | WNT9B |
| TJP2 | TJP2 |
| EGLN1 | EGLN1 |
| NUP160 | NUP160 |
| PTPA | PTPA |
| HOXD13 | HOXD13 |
| ATP6V0A2 | ATP6V0A2 |
| CFC1 | CFC1 |
| MED1 | MED1 |
| IL1RAP | IL1RAP |
| ESS2 | ESS2 |
| LRP1 | LRP1 |
| CSPP1 | CSPP1 |
| GTF2I | GTF2I |
| MCAM | MCAM |

| | |
|---|---|
| PEX13 | PEX13 |
| ATRIP | ATRIP |
| FSTL1 | FSTL1 |
| LONP1 | LONP1 |
| NPPC | NPPC |
| SOX10 | SOX10 |
| TIGIT | TIGIT |
| BLOC1S6 | BLOC1S6 |
| DUOX1 | DUOX1 |
| KIF7 | KIF7 |
| TMEM43 | TMEM43 |
| CASK | CASK |
| IFT74 | IFT74 |
| DNAJB11 | DNAJB11 |
| DEFA1 | DEFA1 |
| CYP2C9 | CYP2C9 |
| ZBTB16 | ZBTB16 |
| DNM1L | DNM1L |
| GABRA3 | GABRA3 |
| GGH | GGH |
| SKIL | SKIL |
| RNASEH2B | RNASEH2B |
| UTP4 | UTP4 |
| LOC111674477 | LOC111674477 |
| PSMD3 | PSMD3 |
| ROCK1 | ROCK1 |
| RRAS2 | RRAS2 |
| MIR487A | MIR487A |
| BICC1 | BICC1 |
| FZD7 | FZD7 |
| SRCAP | SRCAP |
| NKX2-6 | NKX2-6 |
| NUP107 | NUP107 |
| KCNH1 | KCNH1 |
| TK2 | TK2 |
| SULT1A3 | SULT1A3 |
| TNFRSF4 | TNFRSF4 |
| REL | REL |
| MAP1B | MAP1B |
| CASP2 | CASP2 |
| MIR432 | MIR432 |
| IFT122 | IFT122 |
| CXCL17 | CXCL17 |
| CARD9 | CARD9 |
| WSPAR | WSPAR |

| | |
|---|---|
| SLCO1B3 | SLCO1B3 |
| FGF5 | FGF5 |
| MAGI2 | MAGI2 |
| RIGI | RIGI |
| RAD21 | RAD21 |
| NTF3 | NTF3 |
| DEK | DEK |
| ANO1 | ANO1 |
| ALDOB | ALDOB |
| NDRG1 | NDRG1 |
| RPLP0 | RPLP0 |
| APOC3 | APOC3 |
| ANG | ANG |
| CYB5R3 | CYB5R3 |
| HCFC1 | HCFC1 |
| GREB1L | GREB1L |
| MIR874 | MIR874 |
| MFN2 | MFN2 |
| TPO | TPO |
| NAB2 | NAB2 |
| MUS81 | MUS81 |
| BBS12 | BBS12 |
| NCL | NCL |
| MIR1247 | MIR1247 |
| SNRPA | SNRPA |
| G6PC3 | G6PC3 |
| FTO | FTO |
| KLRD1 | KLRD1 |
| EIF4A1 | EIF4A1 |
| BBS5 | BBS5 |
| RRM2B | RRM2B |
| SF3B4 | SF3B4 |
| ALCAM | ALCAM |
| OLR1 | OLR1 |
| PTAFR | PTAFR |
| CYP7A1 | CYP7A1 |
| IL32 | IL32 |
| CYP51A1 | CYP51A1 |
| MGA | MGA |
| BCL11B | BCL11B |
| VCAN | VCAN |
| KLF5 | KLF5 |
| DDC | DDC |
| RPL26 | RPL26 |
| EHMT2 | EHMT2 |

| | |
|---|---|
| HEXA | HEXA |
| SRP72 | SRP72 |
| BRD2 | BRD2 |
| PHF6 | PHF6 |
| NEUROD1 | NEUROD1 |
| PPOX | PPOX |
| BTG1 | BTG1 |
| VKORC1 | VKORC1 |
| LIMK1 | LIMK1 |
| SLC12A6 | SLC12A6 |
| CCL7 | CCL7 |
| NID1 | NID1 |
| TRIM21 | TRIM21 |
| RAD18 | RAD18 |
| SDC2 | SDC2 |
| TRIM24 | TRIM24 |
| TOP2B | TOP2B |
| CAMKK2 | CAMKK2 |
| SPECC1L | SPECC1L |
| GFI1 | GFI1 |
| ERAP1 | ERAP1 |
| TNNC1 | TNNC1 |
| TMSB4X | TMSB4X |
| CPT2 | CPT2 |
| PCGF2 | PCGF2 |
| MYSM1 | MYSM1 |
| SYNE2 | SYNE2 |
| ISG15 | ISG15 |
| CAMP | CAMP |
| CCL21 | CCL21 |
| PAK4 | PAK4 |
| ETV5 | ETV5 |
| SSTR1 | SSTR1 |
| MIR660 | MIR660 |
| MLPH | MLPH |
| S100A6 | S100A6 |
| BACH1 | BACH1 |
| CD79B | CD79B |
| NCOA2 | NCOA2 |
| FZD1 | FZD1 |
| SLX4 | SLX4 |
| RPS26 | RPS26 |
| CUL3 | CUL3 |
| GDPD3 | GDPD3 |
| YTHDF1 | YTHDF1 |

| | |
|---|---|
| U2AF2 | U2AF2 |
| SLC34A1 | SLC34A1 |
| BCL11A | BCL11A |
| ORAI1 | ORAI1 |
| CELF2 | CELF2 |
| SIRT6 | SIRT6 |
| TACSTD2 | TACSTD2 |
| IL1R2 | IL1R2 |
| NTN1 | NTN1 |
| MAPT | MAPT |
| TLR10 | TLR10 |
| HSPA1B | HSPA1B |
| COL4A6 | COL4A6 |
| KDM3B | KDM3B |
| GCLM | GCLM |
| HNRNPC | HNRNPC |
| NOD1 | NOD1 |
| FREM1 | FREM1 |
| PFN1 | PFN1 |
| ARVCF | ARVCF |
| YWHAQ | YWHAQ |
| CDC5L | CDC5L |
| ZIC2 | ZIC2 |
| ASCC1 | ASCC1 |
| SLCO2B1 | SLCO2B1 |
| ARL13B | ARL13B |
| BGN | BGN |
| MFF-DT | MFF-DT |
| MIR876 | MIR876 |
| ITPA | ITPA |
| NR4A2 | NR4A2 |
| MIR589 | MIR589 |
| PRKCZ | PRKCZ |
| RBM20 | RBM20 |
| ERCC6L2 | ERCC6L2 |
| STAT2 | STAT2 |
| COLQ | COLQ |
| POLR1G | POLR1G |
| MAPKBP1 | MAPKBP1 |
| SLCO2A1 | SLCO2A1 |
| CALM2 | CALM2 |
| LOC113633877 | LOC113633877 |
| MIR769 | MIR769 |
| HNRNPK | HNRNPK |
| ADORA2A | ADORA2A |

| | |
|---|---|
| ADNP | ADNP |
| MUC6 | MUC6 |
| ITPR1 | ITPR1 |
| ADCYAP1 | ADCYAP1 |
| SEMA4D | SEMA4D |
| MPI | MPI |
| AARS1 | AARS1 |
| CARD10 | CARD10 |
| LAMP3 | LAMP3 |
| TFAM | TFAM |
| TMEM138 | TMEM138 |
| KIR2DS4 | KIR2DS4 |
| BCKDHA | BCKDHA |
| TNFRSF25 | TNFRSF25 |
| PLP1 | PLP1 |
| CTHRC1 | CTHRC1 |
| IQCB1 | IQCB1 |
| FANCM | FANCM |
| FLII | FLII |
| SLK | SLK |
| SUMF1 | SUMF1 |
| DCDC2 | DCDC2 |
| PHB1 | PHB1 |
| DAG1 | DAG1 |
| EIF2S1 | EIF2S1 |
| CRH | CRH |
| RPS6 | RPS6 |
| PRPF8 | PRPF8 |
| COL7A1 | COL7A1 |
| NUMA1 | NUMA1 |
| CLCN5 | CLCN5 |
| BAZ1B | BAZ1B |
| IRS2 | IRS2 |
| C3AR1 | C3AR1 |
| PYROXD1 | PYROXD1 |
| SMCHD1 | SMCHD1 |
| OXA1L | OXA1L |
| PCSK1 | PCSK1 |
| SEPTIN9 | SEPTIN9 |
| ADAMTSL1 | ADAMTSL1 |
| CTBP1 | CTBP1 |
| YTHDC2 | YTHDC2 |
| RFXAP | RFXAP |
| ATF1 | ATF1 |
| VPS33B | VPS33B |

| | |
|---|---|
| KDM6B | KDM6B |
| LINC00922 | LINC00922 |
| LPAR3 | LPAR3 |
| MDK | MDK |
| NSUN2 | NSUN2 |
| UHRF1 | UHRF1 |
| CAST | CAST |
| PSEN1 | PSEN1 |
| TNFSF4 | TNFSF4 |
| ZFP36 | ZFP36 |
| GSTO1 | GSTO1 |
| WDR1 | WDR1 |
| DAXX | DAXX |
| GORAB | GORAB |
| DNAJA1 | DNAJA1 |
| SLC25A3 | SLC25A3 |
| ACTR3C | ACTR3C |
| PLA2G10 | PLA2G10 |
| SLC39A8 | SLC39A8 |
| EEF1A1 | EEF1A1 |
| ERF | ERF |
| PDE4D | PDE4D |
| PEX26 | PEX26 |
| UBE2T | UBE2T |
| PUF60 | PUF60 |
| HOXB4 | HOXB4 |
| NFATC1 | NFATC1 |
| FGF19 | FGF19 |
| GAS6 | GAS6 |
| HUS1 | HUS1 |
| EIF4G1 | EIF4G1 |
| UGT2B17 | UGT2B17 |
| CD83 | CD83 |
| KCNMA1 | KCNMA1 |
| ADSL | ADSL |
| CEP89 | CEP89 |
| MANBA | MANBA |
| KRT3 | KRT3 |
| ZNF423 | ZNF423 |
| RIN3 | RIN3 |
| FAM111B | FAM111B |
| CORO1C | CORO1C |
| GLDC | GLDC |
| LTA4H | LTA4H |
| PLEK2 | PLEK2 |

| | |
|---|---|
| MARCKS | MARCKS |
| BLVRB | BLVRB |
| GOLPH3 | GOLPH3 |
| NOP53 | NOP53 |
| EPHB2 | EPHB2 |
| MIR520D | MIR520D |
| USP53 | USP53 |
| KCNJ6 | KCNJ6 |
| ALOX15 | ALOX15 |
| CD209 | CD209 |
| EPX | EPX |
| PTBP1 | PTBP1 |
| GNLY | GNLY |
| MIR524 | MIR524 |
| ALOX15B | ALOX15B |
| LOC113664106 | LOC113664106 |
| MAP3K7 | MAP3K7 |
| MIR504 | MIR504 |
| CDON | CDON |
| CYP3A7 | CYP3A7 |
| ROCK2 | ROCK2 |
| GCK | GCK |
| NEK2 | NEK2 |
| CD244 | CD244 |
| ATF4 | ATF4 |
| PRDM16 | PRDM16 |
| PPT1 | PPT1 |
| FOSL2 | FOSL2 |
| DHX16 | DHX16 |
| INHBA | INHBA |
| TRIM33 | TRIM33 |
| IFT27 | IFT27 |
| SLC26A3 | SLC26A3 |
| ABCB10 | ABCB10 |
| USP9X | USP9X |
| LMOD1 | LMOD1 |
| DDX1 | DDX1 |
| MAGEC2 | MAGEC2 |
| NFIX | NFIX |
| NR0B1 | NR0B1 |
| SEC63 | SEC63 |
| VPS13B | VPS13B |
| TFDP1 | TFDP1 |
| LILRB4 | LILRB4 |
| PEX11B | PEX11B |

| | |
|---|---|
| HELLS | HELLS |
| HTR2A | HTR2A |
| PSEN2 | PSEN2 |
| PRRC2A | PRRC2A |
| SIRPA | SIRPA |
| ZNF667-AS1 | ZNF667-AS1 |
| ZNRF3 | ZNRF3 |
| PHEX | PHEX |
| CXCL16 | CXCL16 |
| RPL19 | RPL19 |
| MAP3K20 | MAP3K20 |
| HPS5 | HPS5 |
| MIR509-1 | MIR509-1 |
| FGF17 | FGF17 |
| SMARCC2 | SMARCC2 |
| GFRA1 | GFRA1 |
| PNPLA3 | PNPLA3 |
| NUP93 | NUP93 |
| PCCB | PCCB |
| LMNB2 | LMNB2 |
| PHLDA2 | PHLDA2 |
| LPAR2 | LPAR2 |
| TSG101 | TSG101 |
| ING4 | ING4 |
| NCOA1 | NCOA1 |
| SATB1 | SATB1 |
| ARID2 | ARID2 |
| FGG | FGG |
| MHRT | MHRT |
| CPQ | CPQ |
| KIF11 | KIF11 |
| MIR217 | MIR217 |
| IGKC | IGKC |
| SCN4A | SCN4A |
| AQP2 | AQP2 |
| CUL4A | CUL4A |
| CDIPT | CDIPT |
| P2RY2 | P2RY2 |
| ACTN2 | ACTN2 |
| SOST | SOST |
| CTSF | CTSF |
| KDM5D | KDM5D |
| PRSS2 | PRSS2 |
| NDUFAF6 | NDUFAF6 |
| ACP1 | ACP1 |

| | |
|---|---|
| ERGIC3 | ERGIC3 |
| HK1 | HK1 |
| SPARCL1 | SPARCL1 |
| TACR1 | TACR1 |
| HOXA13 | HOXA13 |
| DYNC1H1 | DYNC1H1 |
| L1CAM | L1CAM |
| FCGRT | FCGRT |
| PRDX2 | PRDX2 |
| ARX | ARX |
| REV3L | REV3L |
| SEMA3C | SEMA3C |
| FZD4 | FZD4 |
| FER | FER |
| SPN | SPN |
| AGK | AGK |
| SCD | SCD |
| PROX1 | PROX1 |
| WNK1 | WNK1 |
| CBFB | CBFB |
| PTPRU | PTPRU |
| TWNK | TWNK |
| ROBO2 | ROBO2 |
| EXOSC3 | EXOSC3 |
| DDAH1 | DDAH1 |
| TREM1 | TREM1 |
| SYNPO | SYNPO |
| AQP4 | AQP4 |
| TRAPPC11 | TRAPPC11 |
| RPS6KA1 | RPS6KA1 |
| SETX | SETX |
| HJV | HJV |
| KIF1B | KIF1B |
| SNRNP70 | SNRNP70 |
| VIRMA | VIRMA |
| PAX3 | PAX3 |
| RNASEH2A | RNASEH2A |
| H6PD | H6PD |
| CARMIL2 | CARMIL2 |
| FMO2 | FMO2 |
| CANX | CANX |
| CD33 | CD33 |
| SCO2 | SCO2 |
| ACLY | ACLY |
| MIR518A1 | MIR518A1 |

| | |
|---|---|
| ROR1 | ROR1 |
| SEC16A | SEC16A |
| CALCRL | CALCRL |
| MIR767 | MIR767 |
| TFF3 | TFF3 |
| PMAIP1 | PMAIP1 |
| JMJD1C | JMJD1C |
| BCL3 | BCL3 |
| MCC | MCC |
| ST3GAL4 | ST3GAL4 |
| KANSL1 | KANSL1 |
| PTK7 | PTK7 |
| SLC11A2 | SLC11A2 |
| MRI1 | MRI1 |
| CGAS | CGAS |
| ACVR1B | ACVR1B |
| GART | GART |
| MIR548B | MIR548B |
| TAF15 | TAF15 |
| PEX16 | PEX16 |
| SLC22A4 | SLC22A4 |
| RPS10 | RPS10 |
| TFR2 | TFR2 |
| ALDH18A1 | ALDH18A1 |
| DNAJB1 | DNAJB1 |
| NGFR | NGFR |
| PSMC3 | PSMC3 |
| CTSA | CTSA |
| CDC20 | CDC20 |
| OCRL | OCRL |
| ULK1 | ULK1 |
| FGF22 | FGF22 |
| RPL18 | RPL18 |
| YTHDF2 | YTHDF2 |
| BSND | BSND |
| GLUD1 | GLUD1 |
| DHX8 | DHX8 |
| PARD3 | PARD3 |
| LEPQTL1 | LEPQTL1 |
| CALML5 | CALML5 |
| PEX3 | PEX3 |
| FZD6 | FZD6 |
| RPL35A | RPL35A |
| KDM5C | KDM5C |
| MAPK6 | MAPK6 |

| | |
|---|---|
| CDK9 | CDK9 |
| NLRP1 | NLRP1 |
| VPS33A | VPS33A |
| DNAJB6 | DNAJB6 |
| IGLL1 | IGLL1 |
| ADIPOR1 | ADIPOR1 |
| GPX2 | GPX2 |
| MAOA | MAOA |
| MEIS1 | MEIS1 |
| SAA4 | SAA4 |
| FOXD2-AS1 | FOXD2-AS1 |
| CLCN7 | CLCN7 |
| PTGIR | PTGIR |
| POGZ | POGZ |
| RPS11 | RPS11 |
| IL19 | IL19 |
| DDX39B | DDX39B |
| DVL2 | DVL2 |
| RBP1 | RBP1 |
| GFPT1 | GFPT1 |
| HOXB3 | HOXB3 |
| VASP | VASP |
| MYL9 | MYL9 |
| ITGA7 | ITGA7 |
| CD7 | CD7 |
| EFNA1 | EFNA1 |
| HLA-DMA | HLA-DMA |
| MYL3 | MYL3 |
| POLA2 | POLA2 |
| SGCG | SGCG |
| ZMPSTE24 | ZMPSTE24 |
| JAM3 | JAM3 |
| HNRNPUL1 | HNRNPUL1 |
| MIR541 | MIR541 |
| IPO8 | IPO8 |
| SLC29A3 | SLC29A3 |
| UBAP2 | UBAP2 |
| HTRA1 | HTRA1 |
| SSX2 | SSX2 |
| NHERF1 | NHERF1 |
| PTN | PTN |
| PAX7 | PAX7 |
| HTR4 | HTR4 |
| MIR450B | MIR450B |
| DCTN1 | DCTN1 |

| | |
|---|---|
| PLA2G1B | PLA2G1B |
| DNAI7 | DNAI7 |
| FERMT3 | FERMT3 |
| SERPINB9 | SERPINB9 |
| MTM1 | MTM1 |
| ARG2 | ARG2 |
| MASP1 | MASP1 |
| H3-3A | H3-3A |
| UGT1A8 | UGT1A8 |
| HOGA1 | HOGA1 |
| CLEC12A | CLEC12A |
| CD3G | CD3G |
| TARBP2 | TARBP2 |
| PDHA1 | PDHA1 |
| THRA | THRA |
| CNTF | CNTF |
| CSNK2A1 | CSNK2A1 |
| TFF1 | TFF1 |
| DBH | DBH |
| SP110 | SP110 |
| TDG | TDG |
| NUMB | NUMB |
| FLG | FLG |
| CYP21A2 | CYP21A2 |
| SKIC3 | SKIC3 |
| KCNJ11 | KCNJ11 |
| ZNF276 | ZNF276 |
| E2F4 | E2F4 |
| TNFSF13 | TNFSF13 |
| HRG | HRG |
| PRSS1 | PRSS1 |
| UBC | UBC |
| TNFRSF14 | TNFRSF14 |
| HYAL3 | HYAL3 |
| MIR329-1 | MIR329-1 |
| GLO1 | GLO1 |
| MCPH1 | MCPH1 |
| HSD3B2 | HSD3B2 |
| MAPK9 | MAPK9 |
| SLC31A1 | SLC31A1 |
| FGB | FGB |
| TNIP1 | TNIP1 |
| CALML3 | CALML3 |
| MIR642A | MIR642A |
| FLOT1 | FLOT1 |

| | |
|---|---|
| NFKBIE | NFKBIE |
| CCT8 | CCT8 |
| HSPB8 | HSPB8 |
| RAP1A | RAP1A |
| TCAP | TCAP |
| NDUFS4 | NDUFS4 |
| DCBLD2 | DCBLD2 |
| FOXE1 | FOXE1 |
| PEX14 | PEX14 |
| RBM8A | RBM8A |
| MIR488 | MIR488 |
| MIR539 | MIR539 |
| PEX19 | PEX19 |
| LDB3 | LDB3 |
| PARVA | PARVA |
| MAP1LC3A | MAP1LC3A |
| ADAMTS8 | ADAMTS8 |
| SULT2B1 | SULT2B1 |
| ZC3H4 | ZC3H4 |
| EFL1 | EFL1 |
| TNFRSF18 | TNFRSF18 |
| RAB8A | RAB8A |
| TIAM1 | TIAM1 |
| KRT16 | KRT16 |
| ABCF1 | ABCF1 |
| SPEN | SPEN |
| MACC1 | MACC1 |
| CEP55 | CEP55 |
| PDHX | PDHX |
| PAK1 | PAK1 |
| EFTUD2 | EFTUD2 |
| EPHA4 | EPHA4 |
| BMAL1 | BMAL1 |
| TXNRD2 | TXNRD2 |
| SLC10A1 | SLC10A1 |
| CA2 | CA2 |
| MCOLN1 | MCOLN1 |
| CAPN2 | CAPN2 |
| JPH2 | JPH2 |
| MYOG | MYOG |
| DPM1 | DPM1 |
| PSMA7 | PSMA7 |
| RFC3 | RFC3 |
| RIPK4 | RIPK4 |
| TAB1 | TAB1 |

| | |
|---|---|
| TCN2 | TCN2 |
| SLC22A3 | SLC22A3 |
| PPP2R5C | PPP2R5C |
| LRP6 | LRP6 |
| RPN1 | RPN1 |
| TET1 | TET1 |
| HHAT | HHAT |
| ADK | ADK |
| BBIP1 | BBIP1 |
| ITGAE | ITGAE |
| ILF3 | ILF3 |
| TRIM27 | TRIM27 |
| FMOD | FMOD |
| APCS | APCS |
| USP22 | USP22 |
| FGF13 | FGF13 |
| CCNK | CCNK |
| CYSLTR2 | CYSLTR2 |
| FUT8 | FUT8 |
| GCH1 | GCH1 |
| CDC42BPA | CDC42BPA |
| SULF2 | SULF2 |
| TPP1 | TPP1 |
| ENPP1 | ENPP1 |
| OPA1 | OPA1 |
| RFX1 | RFX1 |
| EMILIN1 | EMILIN1 |
| MIR513A1 | MIR513A1 |
| BCORL1 | BCORL1 |
| DOCK6 | DOCK6 |
| RHD | RHD |
| STC1 | STC1 |
| JUND | JUND |
| ASAH2 | ASAH2 |
| SPG7 | SPG7 |
| KCNJ1 | KCNJ1 |
| RFC4 | RFC4 |
| MAP1LC3B | MAP1LC3B |
| PTENP1 | PTENP1 |
| KDM4A | KDM4A |
| THRB | THRB |
| CRAT | CRAT |
| RBBP4 | RBBP4 |
| CSNK2B | CSNK2B |
| MIR410 | MIR410 |

| | |
|---|---|
| LPA | LPA |
| COQ2 | COQ2 |
| YWHAG | YWHAG |
| GSTO2 | GSTO2 |
| SART1 | SART1 |
| NEUROG3 | NEUROG3 |
| COQ8A | COQ8A |
| PDX1 | PDX1 |
| GZMA | GZMA |
| PPP1R12A | PPP1R12A |
| NEXN | NEXN |
| AFDN | AFDN |
| ADD3 | ADD3 |
| ARTN | ARTN |
| HUWE1 | HUWE1 |
| SLC25A4 | SLC25A4 |
| PRG2 | PRG2 |
| MYH14 | MYH14 |
| TRPM6 | TRPM6 |
| GYPA | GYPA |
| PLOD2 | PLOD2 |
| MCM7 | MCM7 |
| CHTOP | CHTOP |
| IGF2-AS | IGF2-AS |
| MYLK2 | MYLK2 |
| USH2A | USH2A |
| GRWD1 | GRWD1 |
| MED13L | MED13L |
| SEMA3A | SEMA3A |
| BCAR1 | BCAR1 |
| BUB3 | BUB3 |
| TRIM37 | TRIM37 |
| MAP3K5 | MAP3K5 |
| MEST | MEST |
| LOC126861898 | LOC126861898 |
| JARID2 | JARID2 |
| PPP1R10 | PPP1R10 |
| PEX10 | PEX10 |
| UTS2 | UTS2 |
| CD93 | CD93 |
| HEY2 | HEY2 |
| TRIB2 | TRIB2 |
| MIR3613 | MIR3613 |
| TLE3 | TLE3 |
| SLC4A1 | SLC4A1 |

| | |
|---|---|
| MLXIPL | MLXIPL |
| C1QA | C1QA |
| POLD3 | POLD3 |
| RPS17 | RPS17 |
| IL37 | IL37 |
| VPS45 | VPS45 |
| SCAP | SCAP |
| GATM | GATM |
| TNK2 | TNK2 |
| LAT | LAT |
| TM4SF1 | TM4SF1 |
| PTGER3 | PTGER3 |
| NUDT6 | NUDT6 |
| PRKAA2 | PRKAA2 |
| DDX39A | DDX39A |
| TFEB | TFEB |
| RFC1 | RFC1 |
| RGS6 | RGS6 |
| CXCL3 | CXCL3 |
| CD200 | CD200 |
| GPC6 | GPC6 |
| SLC4A2 | SLC4A2 |
| PLD1 | PLD1 |
| B3GAT3 | B3GAT3 |
| DLD | DLD |
| RPL34 | RPL34 |
| DUT | DUT |
| AARS2 | AARS2 |
| ABCC9 | ABCC9 |
| MCM5 | MCM5 |
| CTSE | CTSE |
| SIN3A | SIN3A |
| TPD52 | TPD52 |
| NCOA6 | NCOA6 |
| RPLP2 | RPLP2 |
| COMP | COMP |
| AQP9 | AQP9 |
| SCTR | SCTR |
| CHERP | CHERP |
| RTKN2 | RTKN2 |
| CKB | CKB |
| PC | PC |
| DDB1 | DDB1 |
| CTAG2 | CTAG2 |
| UBA2 | UBA2 |

MAF
ADAMTS10
POLR1A
INPP5D
RPS15A
EEF2
ETFA
UCP2
CD2
IL21R
MIR3187
KRT4
RAPGEF5
VIPAS39
EHHADH
LTB
PEX2
SULF1
MTUS1
ARPC2
ADGRE5
LTB4R
GNRH1
RPTOR
HPX
CXCL6
TAGLN2
HIF3A
EPHB3
LRRC8A
ALG8
ANKRD1
ANGPTL4
PIGR
PURA
PSMA6
ERC1
BLK
VSIR
PAF1
PABPC1
HGS
CDT1
MIR103A2
SSTR4

MAF
ADAMTS10
POLR1A
INPP5D
RPS15A
EEF2
ETFA
UCP2
CD2
IL21R
MIR3187
KRT4
RAPGEF5
VIPAS39
EHHADH
LTB
PEX2
SULF1
MTUS1
ARPC2
ADGRE5
LTB4R
GNRH1
RPTOR
HPX
CXCL6
TAGLN2
HIF3A
EPHB3
LRRC8A
ALG8
ANKRD1
ANGPTL4
PIGR
PURA
PSMA6
ERC1
BLK
VSIR
PAF1
PABPC1
HGS
CDT1
MIR103A2
SSTR4

| | |
|---|---|
| ADIPOR2 | ADIPOR2 |
| LRMDA | LRMDA |
| PSMC5 | PSMC5 |
| KIR2DS2 | KIR2DS2 |
| PSMD12 | PSMD12 |
| TRIB1 | TRIB1 |
| TCF7 | TCF7 |
| ADPRH | ADPRH |
| CDC45 | CDC45 |
| ADGRF5 | ADGRF5 |
| MBNL1 | MBNL1 |
| GJB1 | GJB1 |
| SFPQ | SFPQ |
| NEBL | NEBL |
| TOE1 | TOE1 |
| GAL | GAL |
| GPR87 | GPR87 |
| RICTOR | RICTOR |
| NUAK1 | NUAK1 |
| FPR2 | FPR2 |
| TRAF3IP2 | TRAF3IP2 |
| CCL19 | CCL19 |
| PEX7 | PEX7 |
| DSG1 | DSG1 |
| LAPTM4B | LAPTM4B |
| ACTG2 | ACTG2 |
| KHDRBS1 | KHDRBS1 |
| GARS1 | GARS1 |
| MYBL2 | MYBL2 |
| ZBTB24 | ZBTB24 |
| LZTFL1 | LZTFL1 |
| RAB11A | RAB11A |
| TBCD | TBCD |
| RARS2 | RARS2 |
| PLK2 | PLK2 |
| VTCN1 | VTCN1 |
| PPP2CA | PPP2CA |
| SLC3A2 | SLC3A2 |
| EFS | EFS |
| CYB5A | CYB5A |
| TSPO | TSPO |
| ARHGEF7 | ARHGEF7 |
| RPL15 | RPL15 |
| CCT3 | CCT3 |
| TNS4 | TNS4 |

| | |
|---|---|
| MMADHC | MMADHC |
| DDX18 | DDX18 |
| DNAJC21 | DNAJC21 |
| COL6A2 | COL6A2 |
| NONO | NONO |
| ARHGAP26 | ARHGAP26 |
| CYP2C8 | CYP2C8 |
| FLRT3 | FLRT3 |
| TRIB3 | TRIB3 |
| ANK1 | ANK1 |
| COQ7 | COQ7 |
| FERMT1 | FERMT1 |
| SLC9A1 | SLC9A1 |
| KIR3DL2 | KIR3DL2 |
| ADGRG1 | ADGRG1 |
| EGFL6 | EGFL6 |
| IFT43 | IFT43 |
| PITX1 | PITX1 |
| POMT2 | POMT2 |
| MACF1 | MACF1 |
| FKTN | FKTN |
| ACKR3 | ACKR3 |
| SHBG | SHBG |
| G3BP1 | G3BP1 |
| CDC37 | CDC37 |
| DYRK2 | DYRK2 |
| MUC7 | MUC7 |
| EXOC4 | EXOC4 |
| DHX9 | DHX9 |
| IQANK1 | IQANK1 |
| CETP | CETP |
| DOT1L | DOT1L |
| COX5A | COX5A |
| PAG1 | PAG1 |
| CUL7 | CUL7 |
| CRK | CRK |
| GLP1R | GLP1R |
| GLCCI1 | GLCCI1 |
| ZNF384 | ZNF384 |
| MT-CO1 | MT-CO1 |
| PROS1 | PROS1 |
| GGA3 | GGA3 |
| RNASEH2C | RNASEH2C |
| DDIT4 | DDIT4 |
| RBM14 | RBM14 |

| | |
|---|---|
| ELF3 | ELF3 |
| RPL27 | RPL27 |
| CPOX | CPOX |
| CPNE1 | CPNE1 |
| CFP | CFP |
| PCNT | PCNT |
| LPAR6 | LPAR6 |
| RHOC | RHOC |
| NUDC | NUDC |
| TONSL | TONSL |
| FGF16 | FGF16 |
| BIN1 | BIN1 |
| CYP26A1 | CYP26A1 |
| QARS1 | QARS1 |
| LILRB1 | LILRB1 |
| RPLP1 | RPLP1 |
| SSTR3 | SSTR3 |
| LSM2 | LSM2 |
| MIR939 | MIR939 |
| IL17RC | IL17RC |
| IRX1 | IRX1 |
| TALDO1 | TALDO1 |
| LILRB2 | LILRB2 |
| TUBB6 | TUBB6 |
| LTK | LTK |
| UNC45A | UNC45A |
| LIMA1 | LIMA1 |
| IMMT | IMMT |
| PCBP1 | PCBP1 |
| SIAH1 | SIAH1 |
| EGLN3 | EGLN3 |
| SON | SON |
| SATB2 | SATB2 |
| ABCC11 | ABCC11 |
| MMP16 | MMP16 |
| ROBO4 | ROBO4 |
| PCSK2 | PCSK2 |
| ACAT1 | ACAT1 |
| ACTR3B | ACTR3B |
| PEX12 | PEX12 |
| SMAD1 | SMAD1 |
| RLF | RLF |
| CYP2F1 | CYP2F1 |
| DDX17 | DDX17 |
| PKLR | PKLR |

| | |
|---|---|
| IKZF2 | IKZF2 |
| SLC12A3 | SLC12A3 |
| CDC27 | CDC27 |
| ACKR2 | ACKR2 |
| ATG5 | ATG5 |
| SMURF2 | SMURF2 |
| MIR616 | MIR616 |
| HMSD | HMSD |
| FIG4 | FIG4 |
| ZNFX1 | ZNFX1 |
| FGF6 | FGF6 |
| EIF3A | EIF3A |
| RPL10 | RPL10 |
| FPR1 | FPR1 |
| CNP | CNP |
| YME1L1 | YME1L1 |
| GAD2 | GAD2 |
| TBL2 | TBL2 |
| BTG3 | BTG3 |
| KIR2DS5 | KIR2DS5 |
| GNB3 | GNB3 |
| IAPP | IAPP |
| GSE1 | GSE1 |
| RPS15 | RPS15 |
| MSC-AS1 | MSC-AS1 |
| GCG | GCG |
| BRWD1 | BRWD1 |
| PON2 | PON2 |
| PVR | PVR |
| SLC25A1 | SLC25A1 |
| ATF6B | ATF6B |
| SPRY1 | SPRY1 |
| RTN4 | RTN4 |
| TAL1 | TAL1 |
| HLA-H | HLA-H |
| IKZF3 | IKZF3 |
| CCN6 | CCN6 |
| FCHO1 | FCHO1 |
| RHBDF2 | RHBDF2 |
| IVL | IVL |
| NCR3 | NCR3 |
| HNRNPL | HNRNPL |
| CGB3 | CGB3 |
| DPEP1 | DPEP1 |
| ETHE1 | ETHE1 |

| | |
|---|---|
| SUN2 | SUN2 |
| H3-4 | H3-4 |
| NUMBL | NUMBL |
| TBX18 | TBX18 |
| SYVN1 | SYVN1 |
| TBX6 | TBX6 |
| RPS27 | RPS27 |
| GLIS3 | GLIS3 |
| CFHR1 | CFHR1 |
| PSMB4 | PSMB4 |
| SENP1 | SENP1 |
| AAAS | AAAS |
| CLDN3 | CLDN3 |
| EXOSC10 | EXOSC10 |
| SRSF3 | SRSF3 |
| HNRNPDL | HNRNPDL |
| PDLIM1 | PDLIM1 |
| NTN4 | NTN4 |
| PFKP | PFKP |
| GSTM2 | GSTM2 |
| DDX3Y | DDX3Y |
| KRIT1 | KRIT1 |
| SLC22A2 | SLC22A2 |
| NREP | NREP |
| CSNK1D | CSNK1D |
| BLNK | BLNK |
| CPB2 | CPB2 |
| RBBP8 | RBBP8 |
| RPL31 | RPL31 |
| SGCA | SGCA |
| FBXO11 | FBXO11 |
| MIR449A | MIR449A |
| POLR2B | POLR2B |
| SCRIB | SCRIB |
| ACSL4 | ACSL4 |
| SMPD2 | SMPD2 |
| ISL1 | ISL1 |
| KPNB1 | KPNB1 |
| B4GAT1 | B4GAT1 |
| ANAPC1 | ANAPC1 |
| NOS1AP | NOS1AP |
| RBBP6 | RBBP6 |
| STYK1 | STYK1 |
| JAG2 | JAG2 |
| FLNC-AS1 | FLNC-AS1 |

| | |
|---|---|
| SPTBN1 | SPTBN1 |
| RPS7 | RPS7 |
| KLRC2 | KLRC2 |
| RECQL | RECQL |
| PAK5 | PAK5 |
| DBT | DBT |
| WDR5 | WDR5 |
| RBM26 | RBM26 |
| AHSP | AHSP |
| CCN3 | CCN3 |
| FUT2 | FUT2 |
| PFKFB3 | PFKFB3 |
| USP11 | USP11 |
| TNFSF14 | TNFSF14 |
| FUT3 | FUT3 |
| NR5A2 | NR5A2 |
| MLLT3 | MLLT3 |
| LPO | LPO |
| HSD3B1 | HSD3B1 |
| PRG4 | PRG4 |
| FERMT2 | FERMT2 |
| CDH11 | CDH11 |
| SYNM | SYNM |
| TIE1 | TIE1 |
| NR2C2 | NR2C2 |
| PFAS | PFAS |
| CD248 | CD248 |
| SSTR5 | SSTR5 |
| RBX1 | RBX1 |
| ASH2L | ASH2L |
| MGAT5 | MGAT5 |
| SHOX2 | SHOX2 |
| REG3A | REG3A |
| DST | DST |
| EGFL7 | EGFL7 |
| ESPL1 | ESPL1 |
| ALKBH5 | ALKBH5 |
| RAD17 | RAD17 |
| HMGN2 | HMGN2 |
| FNIP1 | FNIP1 |
| RNASEL | RNASEL |
| ORM1 | ORM1 |
| CELSR1 | CELSR1 |
| IARS1 | IARS1 |
| MRAS | MRAS |

| | |
|---|---|
| MSMB | MSMB |
| PLTP | PLTP |
| UBTF | UBTF |
| ELF5 | ELF5 |
| NUBP2 | NUBP2 |
| BCL2L2 | BCL2L2 |
| EIF4B | EIF4B |
| SIPA1 | SIPA1 |
| BNC2 | BNC2 |
| GNB1 | GNB1 |
| GINS2 | GINS2 |
| SP7 | SP7 |
| XK | XK |
| KIR2DS1 | KIR2DS1 |
| MSX2 | MSX2 |
| CALB1 | CALB1 |
| CRTC2 | CRTC2 |
| PPP1CA | PPP1CA |
| TNPO3 | TNPO3 |
| EIF1AX | EIF1AX |
| CERK | CERK |
| CCR8 | CCR8 |
| STK40 | STK40 |
| GNL3 | GNL3 |
| SPAAR | SPAAR |
| NAGA | NAGA |
| STX3 | STX3 |
| DDX27 | DDX27 |
| RAMP2 | RAMP2 |
| HTR3A | HTR3A |
| FCGR1A | FCGR1A |
| SMPD3 | SMPD3 |
| MYO1B | MYO1B |
| MAPKAPK2 | MAPKAPK2 |
| CAD | CAD |
| CGA | CGA |
| RTRAF | RTRAF |
| ZNF875 | ZNF875 |
| MFAP5 | MFAP5 |
| PRODH | PRODH |
| BTLA | BTLA |
| CLEC6A | CLEC6A |
| UGT1A | UGT1A |
| SCP2 | SCP2 |
| ASB16-AS1 | ASB16-AS1 |

| | |
|---|---|
| NELFA | NELFA |
| USB1 | USB1 |
| RGCC | RGCC |
| CCL1 | CCL1 |
| AOC1 | AOC1 |
| IL34 | IL34 |
| CEBPD | CEBPD |
| ADAM8 | ADAM8 |
| TOPBP1 | TOPBP1 |
| TPM4 | TPM4 |
| MARK2 | MARK2 |
| PDGFC | PDGFC |
| ALOX12B | ALOX12B |
| RPL22 | RPL22 |
| C19orf48P | C19orf48P |
| CARMN | CARMN |
| LCT | LCT |
| PDHB | PDHB |
| LMO2 | LMO2 |
| SEC23B | SEC23B |
| MIR518D | MIR518D |
| MYO5B | MYO5B |
| TFE3 | TFE3 |
| RDX | RDX |
| MIR100HG | MIR100HG |
| SIRT2 | SIRT2 |
| VAPA | VAPA |
| POGLUT1 | POGLUT1 |
| SASH1 | SASH1 |
| ATAD3B | ATAD3B |
| HMGCL | HMGCL |
| MTA2 | MTA2 |
| PADI2 | PADI2 |
| KDM4C | KDM4C |
| ALAS2 | ALAS2 |
| LUM | LUM |
| PELP1 | PELP1 |
| ARCN1 | ARCN1 |
| ZKSCAN3 | ZKSCAN3 |
| ANXA4 | ANXA4 |
| LBX1 | LBX1 |
| SMARCD1 | SMARCD1 |
| HLA-DRB4 | HLA-DRB4 |
| DDX59 | DDX59 |
| MDH2 | MDH2 |

| | |
|---|---|
| LEF1 | LEF1 |
| MAP3K11 | MAP3K11 |
| MAP3K3 | MAP3K3 |
| WWP2 | WWP2 |
| RUVBL1 | RUVBL1 |
| PBK | PBK |
| RHOD | RHOD |
| DCAF8 | DCAF8 |
| FABP4 | FABP4 |
| SF3A1 | SF3A1 |
| SLCO1A2 | SLCO1A2 |
| ARHGAP31 | ARHGAP31 |
| GATAD1 | GATAD1 |
| BCLAF1 | BCLAF1 |
| DNTT | DNTT |
| CRMP1 | CRMP1 |
| ATP1A1 | ATP1A1 |
| RBSN | RBSN |
| CLDN16 | CLDN16 |
| ADD1 | ADD1 |
| SLC4A4 | SLC4A4 |
| RPS16 | RPS16 |
| USP4 | USP4 |
| CPM | CPM |
| ETV1 | ETV1 |
| P4HA2 | P4HA2 |
| FIP1L1 | FIP1L1 |
| SLC5A1 | SLC5A1 |
| PSMB1 | PSMB1 |
| WRNIP1 | WRNIP1 |
| TLL1 | TLL1 |
| BAMBI | BAMBI |
| TNFSF8 | TNFSF8 |
| OSMR | OSMR |
| ABO | ABO |
| CIRBP | CIRBP |
| CTBP1-DT | CTBP1-DT |
| UBR5 | UBR5 |
| NKX2-8 | NKX2-8 |
| RBM15 | RBM15 |
| RBM39 | RBM39 |
| NCR1 | NCR1 |
| KIR3DS1 | KIR3DS1 |
| PIAS1 | PIAS1 |
| KMT2B | KMT2B |

| | |
|---|---|
| GPR68 | GPR68 |
| UGCG | UGCG |
| VSIG4 | VSIG4 |
| ATP2B4 | ATP2B4 |
| IGES | IGES |
| MAFB | MAFB |
| TACC3 | TACC3 |
| AGPS | AGPS |
| ADAM9 | ADAM9 |
| ACTR2 | ACTR2 |
| AOPEP | AOPEP |
| HOXA-AS3 | HOXA-AS3 |
| RPS3 | RPS3 |
| CCR9 | CCR9 |
| AFF1 | AFF1 |
| PUM3 | PUM3 |
| NR1H3 | NR1H3 |
| PSMB10 | PSMB10 |
| ITGA1 | ITGA1 |
| ADAMTS1 | ADAMTS1 |
| MEPCE | MEPCE |
| RECQL5 | RECQL5 |
| TXNIP | TXNIP |
| RAN | RAN |
| BMP5 | BMP5 |
| NCAPD2 | NCAPD2 |
| MIR101-2 | MIR101-2 |
| CREBZF | CREBZF |
| ADAMTS17 | ADAMTS17 |
| TNFSF9 | TNFSF9 |
| CD1D | CD1D |
| HOXB7 | HOXB7 |
| AKR1A1 | AKR1A1 |
| CALU | CALU |
| ZMYND8 | ZMYND8 |
| ANGPTL2 | ANGPTL2 |
| GALT | GALT |
| ANKRD11 | ANKRD11 |
| SF1 | SF1 |
| SIK1 | SIK1 |
| MYEOV | MYEOV |
| PARP2 | PARP2 |
| NUP205 | NUP205 |
| RARG | RARG |
| DMPK | DMPK |

| | |
|---|---|
| TNFAIP8 | TNFAIP8 |
| KRT1 | KRT1 |
| GAK | GAK |
| GEMIN4 | GEMIN4 |
| MSI2 | MSI2 |
| EEF1G | EEF1G |
| PDIA3 | PDIA3 |
| CCNI | CCNI |
| RNLS | RNLS |
| KDM2B | KDM2B |
| ZNF143 | ZNF143 |
| SCYL1 | SCYL1 |
| PLCB4 | PLCB4 |
| PRDM2 | PRDM2 |
| SEPSECS | SEPSECS |
| MEF2D | MEF2D |
| RPS28 | RPS28 |
| ADCY6 | ADCY6 |
| NAA15 | NAA15 |
| S1PR3 | S1PR3 |
| SLC12A2 | SLC12A2 |
| CD27-AS1 | CD27-AS1 |
| KNSTRN | KNSTRN |
| NFAT5 | NFAT5 |
| SEPTIN7 | SEPTIN7 |
| KPNA2 | KPNA2 |
| CALM1 | CALM1 |
| NRIP1 | NRIP1 |
| RPS20 | RPS20 |
| DDX47 | DDX47 |
| LRIG2 | LRIG2 |
| NELFE | NELFE |
| M6PR | M6PR |
| TEAD1 | TEAD1 |
| MT-CO2 | MT-CO2 |
| UFD1 | UFD1 |
| RPS18 | RPS18 |
| MERTK | MERTK |
| TCF20 | TCF20 |
| ALDH3A1 | ALDH3A1 |
| SLC2A2 | SLC2A2 |
| TAX1BP1 | TAX1BP1 |
| ATP5F1B | ATP5F1B |
| RNF40 | RNF40 |
| NIPAL4 | NIPAL4 |

| | |
|---|---|
| PER1 | PER1 |
| DYRK1A | DYRK1A |
| TTF2 | TTF2 |
| CEP104 | CEP104 |
| VANGL2 | VANGL2 |
| CDK5RAP3 | CDK5RAP3 |
| CCN5 | CCN5 |
| CCL8 | CCL8 |
| VDAC2 | VDAC2 |
| EEF2K | EEF2K |
| KAT8 | KAT8 |
| LGR4 | LGR4 |
| GAP43 | GAP43 |
| MIR26A2 | MIR26A2 |
| KAT2A | KAT2A |
| PCBP2 | PCBP2 |
| AKAP1 | AKAP1 |
| PLEK | PLEK |
| SPTB | SPTB |
| CEMIP | CEMIP |
| MFAP2 | MFAP2 |
| CSNK1E | CSNK1E |
| | DBR1 |
| | SLC67A1 |
| | CHRNA3 |
| | SARS2 |

**Table S3. Barbaloin Predicted Target Genes from Four Databases (PharmMapper, SEA, SuperPred, SwissTargetPrediction).**

| PharmMapper | SEA | SuperPred | SwissTargetPrediction | Union |
|---|---|---|---|---|
| NUDT9 | SLC5A2 | TDP1 | EPHX2 | NUDT9 |
| CTSV | NRAS | HSD17B10 | TYR | CTSV |
| MAPK8 | SLC28A3 | APEX1 | CA2 | MAPK8 |
| CHEK1 | CDA | CTSD | CA7 | CHEK1 |
| STS | SLC5A1 | NR1I2 | CA1 | STS |
| MAPK10 | SLC5A11 | ADORA1 | CA12 | MAPK10 |
| TGFBR2 | TOP1 | TRIM24 | CA14 | TGFBR2 |
| EPHB4 | UMPS | TOP2A | CA9 | EPHB4 |
| CA2 | TYR | CLK4 | CA4 | CA2 |
| APOA2 | HRAS | NFKB1 | CA13 | APOA2 |
| CA1 | CA12 | THRA | CA5A | CA1 |
| CLPP | SLC5A4 | DUSP3 | SLC5A2 | CLPP |
| EGFR | CA14 | HDAC5 | CA6 | EGFR |
| | CA9 | | | |

| | | | | |
|---|---|---|---|---|
| GSTP1 | IL2 | CNR1 | HRAS | GSTP1 |
| CTSD | ALDH1B1 | NTRK3 | ADORA3 | CTSD |
| PNP | CA1 | KDM1A | IGFBP3 | PNP |
| ALB | ALDH1A2 | HDAC8 | LGALS3 | ALB |
| MMP3 | IL6 | KLF5 | LGALS9 | MMP3 |
| BCHE | SLC37A4 | HSP90AA1 | ADORA2A | BCHE |
| PPIA | FGF1 | SLC6A5 | CA3 | PPIA |
| BACE1 | TNNC1 | F13A1 | MMP13 | BACE1 |
| DDX6 | TNNI3 | GLRA1 | MMP1 | DDX6 |
| HSP90AA1 | TNNT2 | CHUK | MMP7 | HSP90AA1 |
| MTAP | CA2 | GRIN1 | MMP12 | MTAP |
| FAP | FGF2 | ABCC1 | MMP8 | FAP |
| CFB | CA13 | CSNK2B | CDA | CFB |
| RTN4R | P4HB | CDK1 | ADA | RTN4R |
| CMA1 | ERAP1 | GRIA2 | ADK | CMA1 |
| F2 | PYGM | CDK5 | SLC29A1 | F2 |
| ESR1 | HKDC1 | ALOX12 | NOX4 | ESR1 |
| PGR | YARS1 | KIF11 | PNP | PGR |
| CA12 | | DPP9 | CAMKK2 | CA12 |
| PIM1 | | NR3C2 | IGFBP5 | PIM1 |
| QPCT | | TTR | HSP90AA1 | QPCT |
| CTSS | | HDAC10 | ADORA2B | CTSS |
| FCAR | | GRK5 | CDK2 | FCAR |
| ICAM2 | | SCD | FUCA1 | ICAM2 |
| NR1H2 | | CCNE1 | GAA | NR1H2 |
| AKR1B1 | | NFE2L2 | MANBA | AKR1B1 |
| MAOB | | KLK1 | AMPD3 | MAOB |
| CDK2 | | C5AR1 | TYMP | CDK2 |
| TREM1 | | CNR2 | TOP1 | TREM1 |
| HK1 | | SCN3A | MGAM | HK1 |
| CSNK1G2 | | METAP2 | SLC5A4 | CSNK1G2 |
| CTSB | | S1PR4 | ERN1 | CTSB |
| TTR | | CDC25C | SLC28A3 | TTR |
| DHFR | | NTSR2 | MMP3 | DHFR |
| CDK5R1 | | PTPN7 | MMP9 | CDK5R1 |
| AMD1 | | PSMB1 | ADAM17 | AMD1 |
| HSPA8 | | ACHE | GBA | HSPA8 |
| GSR | | PDGFRA | PYGL | GSR |
| ADH1B | | EGLN1 | GART | ADH1B |
| CASP7 | | CAPN1 | HK2 | CASP7 |
| PDE3B | | ADAM10 | HK1 | PDE3B |
| TGFBR1 | | MDM4 | GPR35 | TGFBR1 |
| KIF11 | | SLC2A1 | POLB | KIF11 |
| DPP4 | | TFPI | TP53 | DPP4 |
| ANG | | CACNA1B | AMY2A | ANG |

| | | | |
|---|---|---|---|
| BCAT2 | BLM | SELE | BCAT2 |
| DCK | GCK | KDM4C | DCK |
| ESRRG | TBXA2R | MMP2 | ESRRG |
| ADH5 | SCN4A | FOLH1 | ADH5 |
| NPR3 | MAOA | MCL1 | NPR3 |
| GC | P2RX4 | MME | GC |
| IMPA1 | STING1 | SI | IMPA1 |
| FGFR1 | MAPK1 | P2RX3 | FGFR1 |
| SRC | IDO1 | YARS | SRC |
| BAG1 | TDO2 | HPRT1 | BAG1 |
| AMY1A | ERAP1 | OGA | AMY1A |
| AMY1B | BMP2K | PFKFB3 | AMY1B |
| AMY1C | GABRA1 | BCL2A1 | AMY1C |
| PDE4B | QRFPR | EIF4H | PDE4B |
| TYMS | ITK | PABPC1 | TYMS |
| IGLV2-8 | CHRM5 | LGALS4 | IGLV2-8 |
| Esr2 | CDC25B | TYMS | Esr2 |
| REN | TACR2 | LGALS8 | REN |
| BMP7 | PKN1 | RNASEH1 | BMP7 |
| NQO1 | TLR8 | F2 | NQO1 |
| PLA2G10 | CYP3A4 | AGTR1 | PLA2G10 |
| PYGL | TOP1 | ATIC | PYGL |
| KDR | GLS | ALOX12 | KDR |
| DUSP6 | AOC3 | DAO | DUSP6 |
| MIF | PIN1 | KDM3A | MIF |
| CFD | PRCP | MAP2K1 | CFD |
| PDE4D | LDHB | KDM5B | PDE4D |
| HADH | MAP2K2 | KDM4D | HADH |
| NOS3 | SLC1A3 | CDK2 | NOS3 |
| DAPK1 | TACR1 | CCNA1 | DAPK1 |
| TYMP | CTSS | CCNA2 | TYMP |
| IGF1R | GPBAR1 | HSPA8 | IGF1R |
| CCNA2 | PLAT | HSPA5 | CCNA2 |
| AKR1C1 | MARK4 | PTPN2 | AKR1C1 |
| CTSK | GPR17 | MKNK2 | CTSK |
| PAH | PROC | CHEK1 | PAH |
| MAPK14 | AVPR1B | IMPDH1 | MAPK14 |
| CBR1 | CBX4 | IMPDH2 | CBR1 |
| SHBG | P2RX7 | CASP6 | SHBG |
| GSK3B | HDAC11 | CASP7 | GSK3B |
| PPARD | TLR4 | CASP8 | PPARD |
| CDA | ABL1 | METAP2 | CDA |
| SOD2 | MIF | AMD1 | SOD2 |
| PPARG | ZAP70 | CASP1 | PPARG |
| MMP7 | TYRO3 | | MMP7 |

| | | |
|---|---|---|
| PPP5C | CHRNA1 | PPP5C |
| PLK1 | | PLK1 |
| DHODH | | DHODH |
| FNTA | | FNTA |
| MMP8 | | MMP8 |
| AKR1C3 | | AKR1C3 |
| ADH1C | | ADH1C |
| HDAC8 | | HDAC8 |
| REG1A | | REG1A |
| LCK | | LCK |
| B3GAT1 | | B3GAT1 |
| AMY2A | | AMY2A |
| AKT1 | | AKT1 |
| SORD | | SORD |
| EPHA2 | | EPHA2 |
| IMPDH2 | | IMPDH2 |
| MAN1B1 | | MAN1B1 |
| CES1 | | CES1 |
| F10 | | F10 |
| ISG20 | | ISG20 |
| SULT2A1 | | SULT2A1 |
| HEXB | | HEXB |
| LGALS7 | | LGALS7 |
| LGALS7B | | LGALS7B |
| AR | | AR |
| ADK | | ADK |
| HSD17B1 | | HSD17B1 |
| YARS1 | | YARS1 |
| HSD11B1 | | HSD11B1 |
| TPH1 | | TPH1 |
| EPHX2 | | EPHX2 |
| AHCY | | AHCY |
| PDPK1 | | PDPK1 |
| ATOX1 | | ATOX1 |
| CTSG | | CTSG |
| LGALS2 | | LGALS2 |
| PLAU | | PLAU |
| RAC2 | | RAC2 |
| SEC14L2 | | SEC14L2 |
| PTPN1 | | PTPN1 |
| JAK3 | | JAK3 |
| PRKACA | | PRKACA |
| ADAM17 | | ADAM17 |
| BST1 | | BST1 |
| HPN | | HPN |

| | |
|---|---|
| TNK2 | TNK2 |
| RHOA | RHOA |
| ACP3 | ACP3 |
| FABP4 | FABP4 |
| ALDH2 | ALDH2 |
| ELANE | ELANE |
| PARP1 | PARP1 |
| MMP12 | MMP12 |
| CSNK2A1 | CSNK2A1 |
| CDK6 | CDK6 |
| HMGCR | HMGCR |
| SYK | SYK |
| UCK2 | UCK2 |
| GPI | GPI |
| LDHB | LDHB |
| GNPDA1 | GNPDA1 |
| NR1I2 | NR1I2 |
| NOS2 | NOS2 |
| PDHB | PDHB |
| RNASE4 | RNASE4 |
| PGF | PGF |
| IL2 | IL2 |
| CASP3 | CASP3 |
| FHIT | FHIT |
| NR3C2 | NR3C2 |
| AZGP1 | AZGP1 |
| C1R | C1R |
| JAK2 | JAK2 |
| HCK | HCK |
| ITK | ITK |
| F7 | F7 |
| RXRA | RXRA |
| RHEB | RHEB |
| S100A9 | S100A9 |
| NCS1 | NCS1 |
| PCK1 | PCK1 |
| RAB11A | RAB11A |
| ABO | ABO |
| LGALS3 | LGALS3 |
| ARG2 | ARG2 |
| MMP13 | MMP13 |
| MET | MET |
| ABO | TGM3 |
| TGM3 | CYP2C9 |
| CYP2C9 | IGF1 |

IGF1
FKBP1A
THRB
CTNNA1
NQO2
SELP
ALDOA
RNASE3
DTYMK
TK1
DPEP1
SSE1
MAP-
KAPK2
ADAM17
TPI1
ZAP70
PTPN11
MTHFD1
UMPS
CAT
SHMT1
CCNT1
GSTA1
CCL5
ARHGAP1
HINT1
GSTT2B
AKR1C2
PDE5A
APRT
BIRC7
C1S
PDK2
SERPINA1
LYZ
KYAT1
FECH
CHIT1
RAB5A
ST14
GALE
ABL1
NR3C1
ERBB4

FKBP1A
THRB
CTNNA1
NQO2
SELP
ALDOA
RNASE3
DTYMK
TK1
DPEP1
SSE1
MAP-
KAPK2
TPI1
ZAP70
PTPN11
MTHFD1
UMPS
CAT
SHMT1
CCNT1
GSTA1
CCL5
ARHGAP1
HINT1
GSTT2B
AKR1C2
PDE5A
APRT
BIRC7
C1S
PDK2
SERPINA1
LYZ
KYAT1
FECH
CHIT1
RAB5A
ST14
GALE
ABL1
NR3C1
ERBB4
CSK
FDPS

CSK
FDPS
WARS1
ATIC
CLK1
IMPDH1
CD1A
CD209
FABP6
STAT1
THRB
INSR
SRM
MMP9
SETD7
THRA
PADI4
FABP3
XIAP
PIK3R1
ITPKA
CYP2C8
KIT
NMNAT1
ARG1
MMP16
PLEKHA4
PAK6
PAPSS1
ARSA
MMP2
TRAPPC3
PRKCQ
KAT2B
HRAS
HAGH
SDS
PLA2G2A
GP1BA
PITPNA
GSTA3
ARL5A
NT5M
AKT2
SULT2B1

WARS1
ATIC
CLK1
IMPDH1
CD1A
CD209
FABP6
STAT1
INSR
SRM
MMP9
SETD7
THRA
PADI4
FABP3
XIAP
PIK3R1
ITPKA
CYP2C8
KIT
NMNAT1
ARG1
MMP16
PLEKHA4
PAK6
PAPSS1
ARSA
MMP2
TRAPPC3
PRKCQ
KAT2B
HRAS
HAGH
SDS
PLA2G2A
GP1BA
PITPNA
GSTA3
ARL5A
NT5M
AKT2
SULT2B1
SULT1E1
ACADM
GSTM2

SULT1E1
ACADM
GSTM2
GCK
PPCDC
MAPK1
CBS
PTK2

GCK
PPCDC
MAPK1
CBS
PTK2
SLC5A2
NRAS
SLC28A3
SLC5A1
SLC5A11
TOP1
TYR
SLC5A4
CA14
CA9
ALDH1B1
ALDH1A2
IL6
SLC37A4
FGF1
TNNC1
TNNI3
TNNT2
FGF2
CA13
P4HB
ERAP1
PYGM
HKDC1
TDP1
HSD17B10
APEX1
ADORA1
TRIM24
TOP2A
CLK4
NFKB1
DUSP3
HDAC5
CNR1
NTRK3
KDM1A
KLF5
SLC6A5
F13A1

GLRA1
CHUK
GRIN1
ABCC1
CSNK2B
CDK1
GRIA2
CDK5
ALOX12
DPP9
HDAC10
GRK5
SCD
CCNE1
NFE2L2
KLK1
C5AR1
CNR2
SCN3A
METAP2
S1PR4
CDC25C
NTSR2
PTPN7
PSMB1
ACHE
PDGFRA
EGLN1
CAPN1
ADAM10
MDM4
SLC2A1
TFPI
CACNA1B
BLM
TBXA2R
SCN4A
MAOA
P2RX4
STING1
IDO1
TDO2
BMP2K
GABRA1
QRFPR

CHRM5
CDC25B
TACR2
PKN1
TLR8
CYP3A4
GLS
AOC3
PIN1
PRCP
MAP2K2
SLC1A3
TACR1
GPBAR1
PLAT
MARK4
GPR17
PROC
AVPR1B
CBX4
P2RX7
HDAC11
TLR4
TYRO3
CHRNA1
CA7
CA4
CA5A
CA6
ADORA3
IGFBP3
LGALS9
ADORA2A
CA3
MMP1
ADA
SLC29A1
NOX4
CAMKK2
IGFBP5
ADORA2B
FUCA1
GAA
MANBA
AMPD3

| | |
|---|---|
| | MGAM |
| | ERN1 |
| | GBA |
| | GART |
| | HK2 |
| | GPR35 |
| | POLB |
| | TP53 |
| | SELE |
| | KDM4C |
| | FOLH1 |
| | MCL1 |
| | MME |
| | SI |
| | P2RX3 |
| | YARS |
| | HPRT1 |
| | OGA |
| | PFKFB3 |
| | BCL2A1 |
| | EIF4H |
| | PABPC1 |
| | LGALS4 |
| | LGALS8 |
| | RNASEH1 |
| | AGTR1 |
| | DAO |
| | KDM3A |
| | MAP2K1 |
| | KDM5B |
| | KDM4D |
| | CCNA1 |
| | HSPA5 |
| | PTPN2 |
| | MKNK2 |
| | CASP6 |
| | CASP8 |
| | CASP1 |

**Table S4. Antibodies used for western blot and immunofluorescence.**

| Target Protein | Host Species | Modification | Source (Catalog No.) | Dilution |
|---|---|---|---|---|
| Phospho-NF-κB p65 | Rabbit | Ser536 | Cell Signaling Technology (3033) | 1:1000 |
| NF-κB p65 | Rabbit | Total | Cell Signaling Technology (3034) | 1:1000 |
| NLRP3 | Rabbit | - | Immunoway (YT5382) | 1:1000 |

| | | | | |
|---|---|---|---|---|
| IL-1β | Rabbit | - | Abclonal (A20527) | 1:1000 |
| Cleaved caspase-1 | Rabbit | - | Proteintech (22915-1-AP) | 1:1000 |
| GAPDH | Mouse | - | Proteintech (60004-1-Ig) | 1:10000 |
| IL-1β (for IHC) | Rabbit | - | Servicebio (GB11113) | 1:800 |
| IL-6 (for IHC) | Rabbit | - | Servicebio (GB11117) | 1:400 |

## Table S5. Primers Used for qRT-PCR.

| Gene | Species | Sequence | |
|---|---|---|---|
| | | Forward ( 5'→3') | Reverse ( 5'→3') |
| Gapdh | Mouse | CTCGCTCCTGGAA-GATGGTGATGG | TCCAGTATGACTCCACTCAC-GGCA |
| Il-6 | Mouse | CCTTCTTGG-GACTGATGCTGGTGA | TGGGAGTGG-TATCCTCTGTGAAGT |
| Pnp | Mouse | TCCCAGAAGTTATCGTCG-CAAGGC | CCGG-CATCCAGAACTTCCATGTGA |
| Il-1β | Mouse | TGACCCTGAGCGACCTGTCTT-GGC | TCCCCAGGG-CATGTTAAGGAGCTC |
| Gapdh | Human | CCTGCCGTCTAGAAAAAC-CTGCCA | GTGGGTGTCGCTGTT-GAAGTCAGA |
| Il-6 | Human | CTTCGGCAAATGTAGCATGGG-CAC | TTCTGTGCCCAGTG-GACAGGTTTC |
| Pnp | Human | TTATGGCCAGCATTCCAC-TCCCTG | ACTTGGGTCTTGTGTGGGA-GATGC |
