# Supplementary figures and images for: Barbaloin Alleviates Lung Ischemia-Reperfusion Injury by Dual-Targeting IL-6 and PNP
Source: Int J Mol Sci. 2026 Jun 10;27(12):5276. doi: 10.3390/ijms27125276 (PMC13300021; doi:10.3390/ijms27125276)

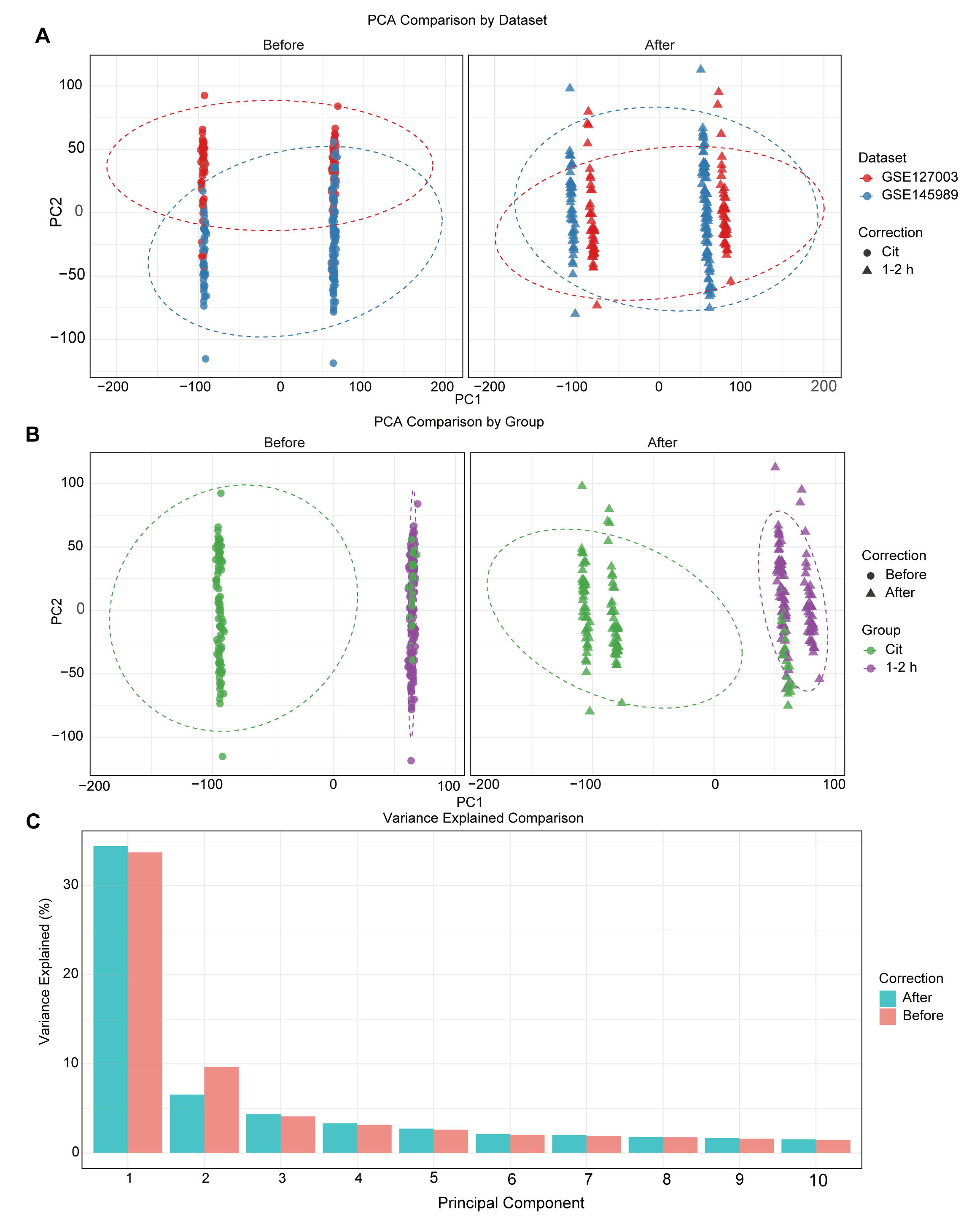

Supplement: Supplementary file 1 [file ijms-27-05276-s001.zip › Figure S1.tif]

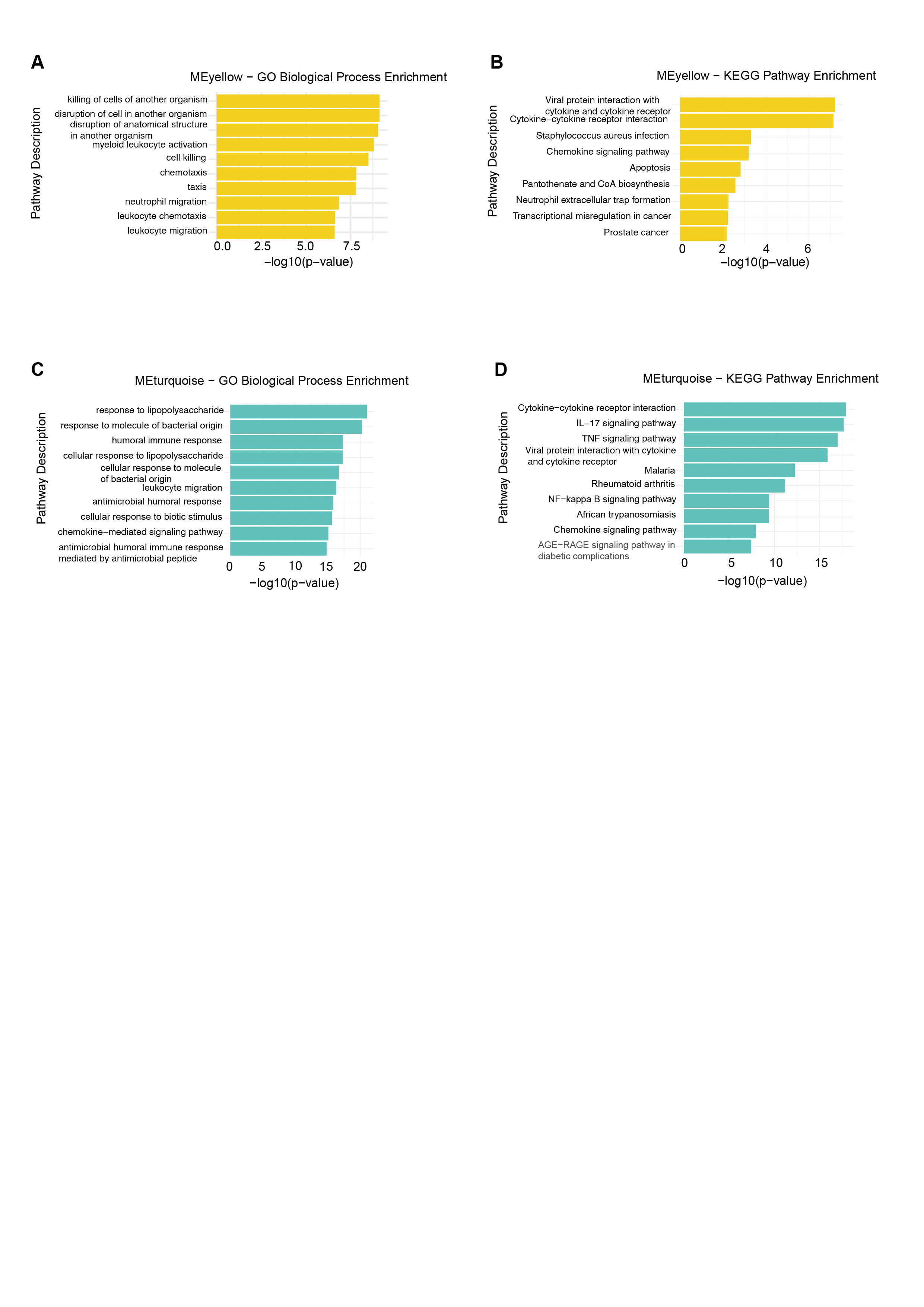

Supplement: Supplementary file 1 [file ijms-27-05276-s001.zip › Figure S2.tif]

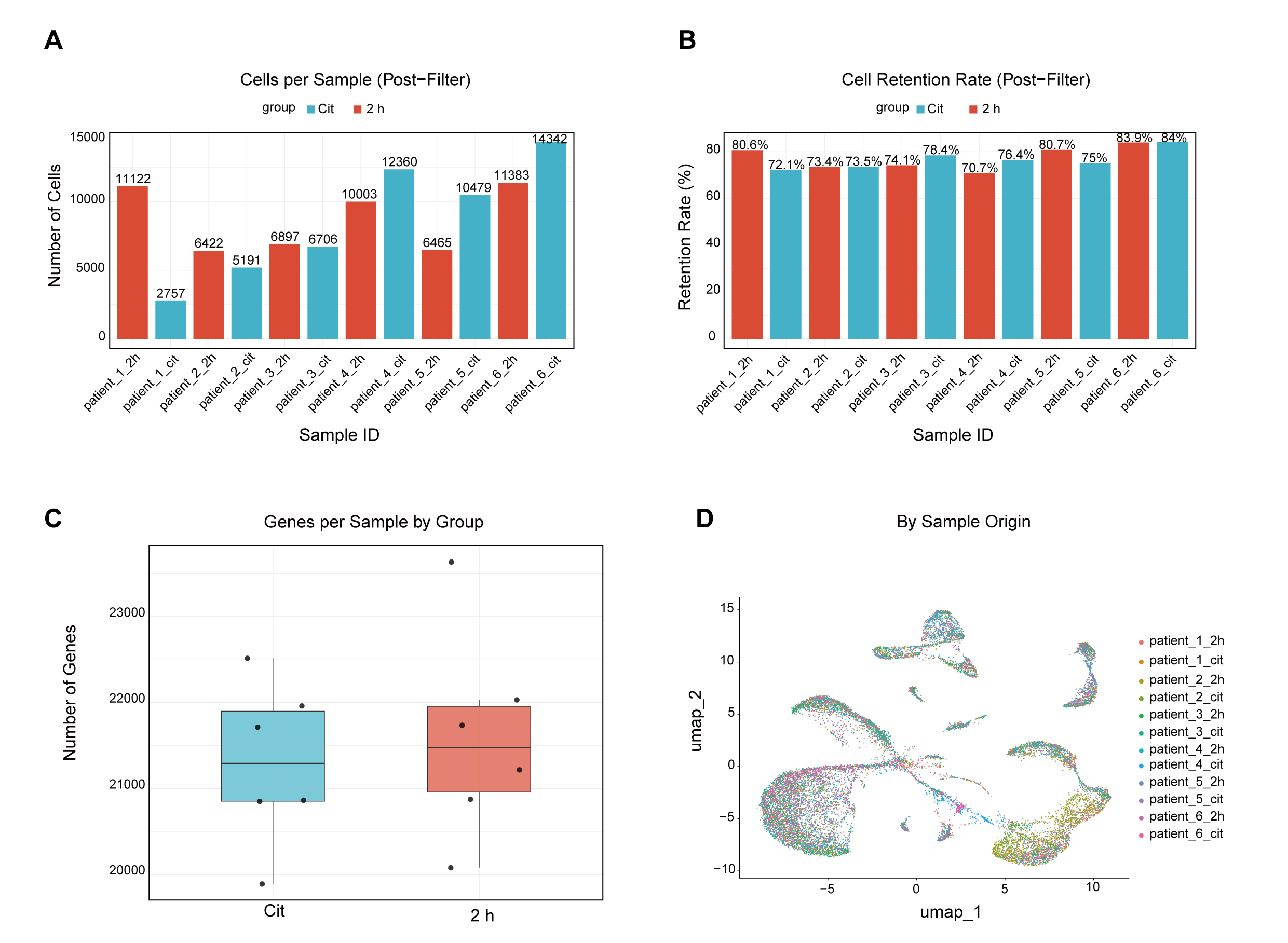

Supplement: Supplementary file 1 [file ijms-27-05276-s001.zip › Figure S3.tif]
